# Synthesis and evaluation of 6-arylaminobenzamides as positron emission tomography imaging ligands for the sphingosine-1-phosphate-5 receptor†

**DOI:** 10.1039/d4md00929k

**Published:** 2025-01-03

**Authors:** Timaeus E. F. Morgan, Emma K. Grant, Robert C. Shaw, Lachlan J. N. Waddell, Martyn C. Henry, Holly McErlain, Carlos J. Alcaide-Corral, Sally L. Pimlott, Adriana A. S. Tavares, Andrew Sutherland

**Affiliations:** a School of Chemistry, University of Glasgow, University Avenue Glasgow G12 8QQ UK Andrew.Sutherland@glasgow.ac.uk; b Edinburgh Imaging, University of Edinburgh 47 Little France Crescent Edinburgh EH16 4TJ UK; c University/BHF Centre for Cardiovascular Sciences, University of Edinburgh 47 Little France Crescent Edinburgh EH16 4TJ UK; d West of Scotland PET Centre, Greater Glasgow and Clyde NHS Trust Glasgow G12 OYN UK

## Abstract

The sphingosine-1-phosphate-5 (S1P_5_) receptor is one of the five membrane G protein-coupled receptors that are activated by the lysophospholipid, sphingosine-1-phosphate, resulting in regulation of many cellular processes. S1P_5_ receptors are located on oligodendrocytes and are proposed to influence oligodendrocyte physiology. Understanding S1P_5_ modulation during processes such as remyelination could have potential applications for demyelinating CNS disorders such as multiple sclerosis (MS). Herein, we report the synthesis and preliminary evaluation of a series of fluorinated 6-arylaminobenzamides as positron emission tomography (PET) ligands of S1P_5_. Pharmacokinetic screening and binding evaluation using a [^35^S]GTPγS assay led to the discovery of TEFM78, a selective and high affinity agonist of S1P_5_. Radiosynthesis of [^18^F]TEFM78 allowed pilot PET imaging studies in an animal model, which showed that [^18^F]TEFM78 can cross the blood brain barrier with good uptake in rat brain and spinal cord.

## Abstract 2

A series of fluorinated 6-arylaminobenzamides have been shown to be selective and potent agonists of the sphingosine-1-phosphate-5 receptor. Radiofluorination and PET imaging with the lead compound showed fast uptake in rat brain and spinal cord.

## Graphical abstract

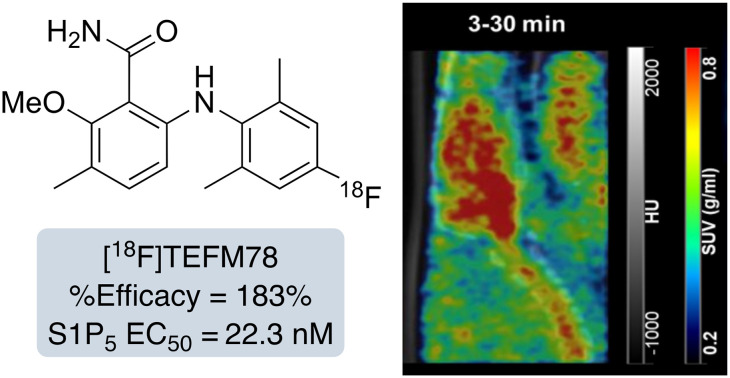

## Introduction

**Fig. 1:**
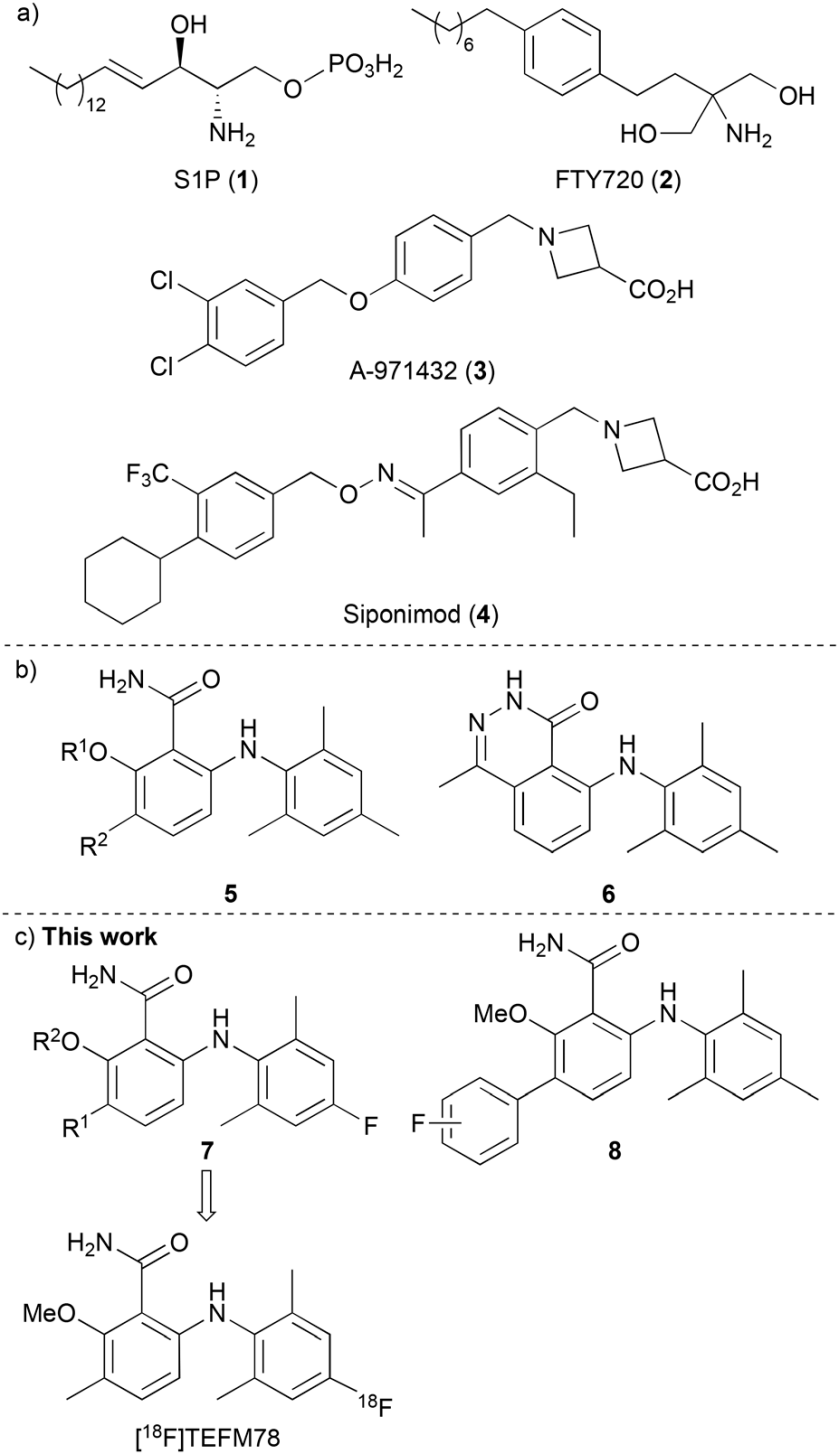
a) Agonists of S1P receptors. b) Selective agonists of S1P_5_. c) This work: assessment of fluorinated 6-arylaminobenzamides.

The lysophospholipid sphingosine-1-phosphate (S1P) (1) is an important signaling molecule, which plays a key role as a cellular mediator (Fig. 1a).^1,2^ This activity occurs through interaction with five highly specific, membrane protein-coupled receptors, S1P_1–5_, which are involved in cell growth and apoptosis in cells of the immune, cardiovascular and central nervous systems.^3,4^ Interest in the S1P_1–5_ receptors increased following the U.S. Food and Drug Administration approval of the S1P analogue FTY720 (Fingolimod, 2), developed for the treatment of patients with relapsing-remitting multiple sclerosis (MS).^5,6^ On phosphorylation, FTY720 engages with four of the S1P receptors (all except S1P_2_), and crucially, acts as a functional antagonist to S1P_1_. This results in receptor internalisation and degradation, leading to lymphocyte sequestration in lymph nodes, preventing entry into the CNS and a relapse of MS.

The S1P_5_ receptor is mainly expressed in white matter tracts of the CNS, on oligodendrocytes, brain cells that are involved in the regulation of the myelination process.^7,8^ Studies have shown that S1P activation of S1P_5_ receptors promotes the survival of myelin-forming mature oligodendrocytes,^9^ and this has led to the proposal that selective agonists of this receptor could also have potential applications in various demyelinating CNS disorders such as MS. In fact, selective agonists (*e.g.*3, Fig. 1a)^10^ and antagonists^11^ of S1P_5_ have been reported. This includes Siponimod (4), a selective S1P_1_ and S1P_5_ modulator that has been recently approved for the treatment of MS, especially for the active secondary progressive form of the disease.^12^ However, the exact mechanism of action of compounds such as siponimod is still unclear and thus, tool compounds are required to fully understand the effects of S1P_5_ modulation and how this relates to neurodegenerative disease.

The radionuclide imaging technique of positron emission tomography (PET) is widely used in the biological and medical sciences for the study of biochemical processes in the living body.^13^ The use of fluorine-18 labelled compounds is of particular interest due to the relatively long half-life (*t*_1/2_ = 109.7 min) and low emission energy of this radioisotope, which results in high resolution images.^14^ In recent years, various PET radioligands have been reported as imaging tools for the S1P receptors, with particular focus on the development of carbon-11 and fluorine-18 labelled compounds for the study of S1P_1_.^15^ As far as we are aware, no fluorine-18 radioligands with S1P_5_ specificity have yet been reported. In 2010, Mattes and co-workers reported a series of selective and potent S1P_5_ agonists that included 6-arylaminobenzamides 5 and 6-arylaminophthalazinone 6 (Fig. 1b).^16^ Based on the high receptor selectivity and bioavailability properties of the 6-arylaminobenzamides, we proposed that these could serve as suitable scaffolds for a structure activity relationship study for the discovery of a S1P_5_ PET radioligand. It was believed that fluorine atom incorporation at one of the existing aromatic rings (*e.g.*7) or the addition of a fluoro-(hetero)aryl group (8) would generate potential PET imaging agents for S1P_5_ (Fig. 1c). Here, we report the design, synthesis and pharmacokinetic properties of a series of fluorinated 6-arylaminobenzamides as selective agonists of the S1P_5_ receptor. We also describe the preliminary evaluation of lead compound [^18^F]TEFM78, which showed good brain and spinal cord uptake in rodents.

## Results and discussion

### Design and chemistry

**Scheme 1:**
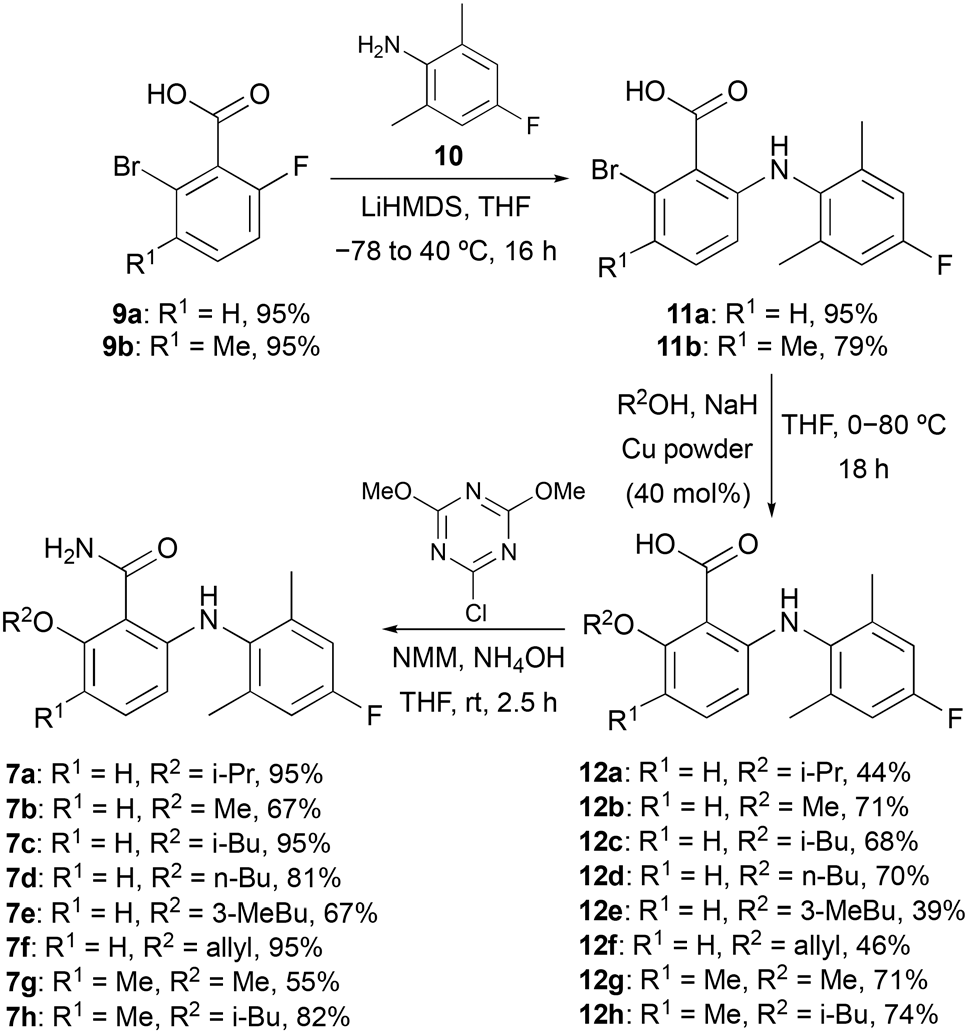
Three-step synthesis of fluorinated 6-arylaminobenzamides 7a–7h.

Docking studies performed by Mattes and co-workers using homology models of bovine rhodopsin and a 6-arylaminobenzamide suggested these compounds adopt a twisted conformation with the benzamide ring residing in a large hydrophobic pocket.^16^ In addition, smaller cavities near the *ortho*- and *meta*-positions of the benzamide ring indicated positions to develop receptor selectivity. Using this insight, our first series of fluorinated 6-arylaminobenzamides explored various alkoxy groups at the *ortho*-position of the benzamide, while incorporating the key fluorine atom within the aniline ring (Scheme 1). These compounds were prepared in three steps. Nucleophilic aromatic substitution of 2-bromo-6-fluorobenzoic acid (9a) with 2,6-dimethyl-4-fluoroaniline (10) in the presence of LiHMDS,^17^ gave 2-arylaminobenzamide 11a in 95% yield. Various alkoxy groups were then introduced at the *ortho*-position using a copper-catalysed Ullmann coupling of bromide 11a with a range of alcohols.^18^ The synthesis of the first series of targets 7a–7f was then completed by amidation using 2-chloro-4,6-dimethoxy-1,3,5-triazine, *N*-methylmorpholine (NMM) and ammonia.^19^ Further SAR studies by the Novartis group demonstrated that high S1P_5_ selectivity could be achieved by the incorporation of substituents at the *meta*-position of the benzamide ring.^16^ Based on this, two further compounds (7g and 7h) containing a 3-methyl group were prepared using this route from 2-bromo-3-methyl-6-fluorobenzoic acid (9b).

**Scheme 2:**
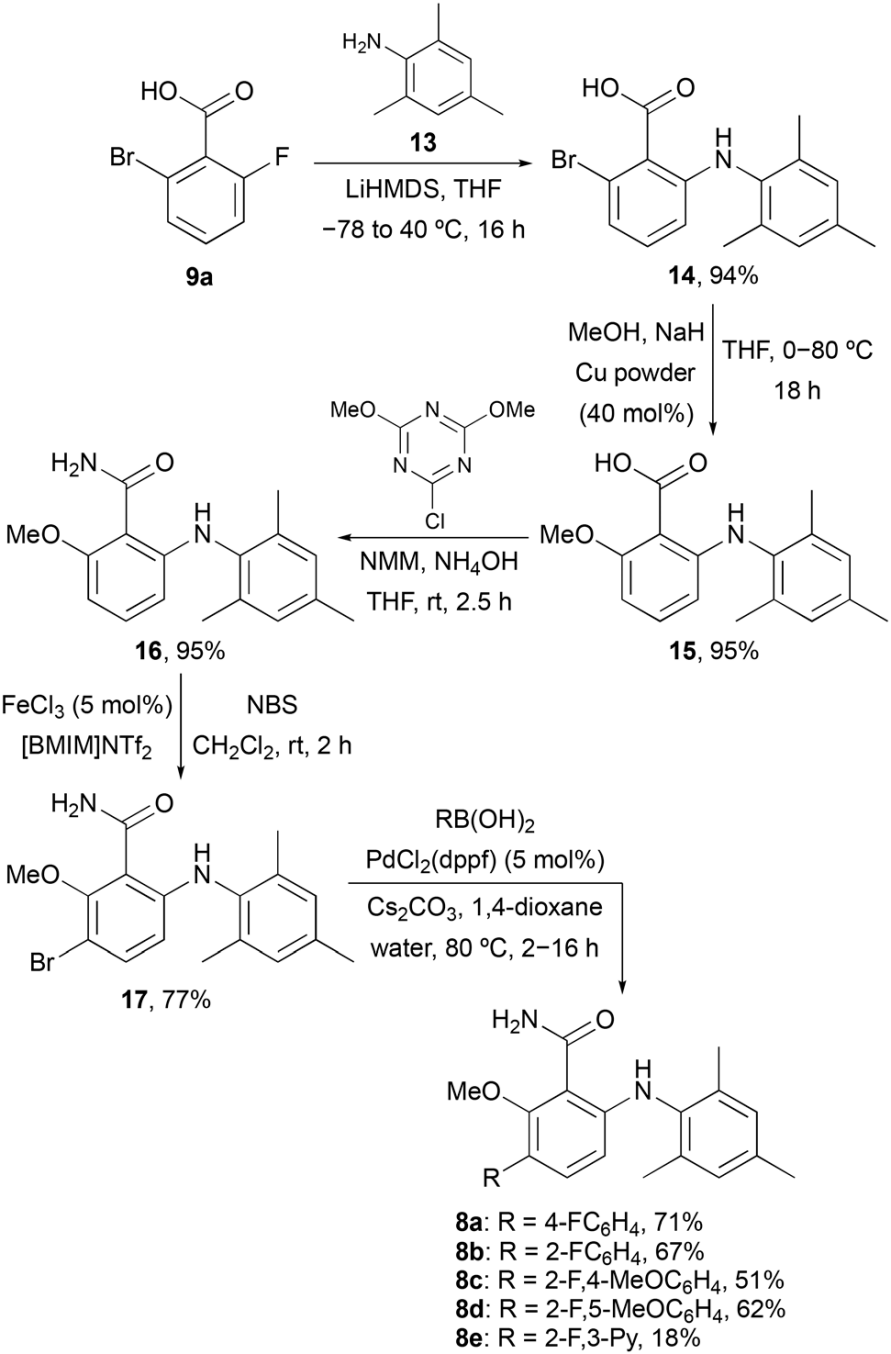
Synthesis of 6-arylaminobenzamides 8a–8e.

To further probe the *meta*-position of the benzamide ring, a second series of agonists of the S1P_5_ receptor was prepared by incorporation of a fluorine-containing (hetero)aryl substituent at this position (Scheme 2). The same series of reactions involving nucleophilic aromatic substitution, copper-mediated methanolysis and amidation gave 6-arylaminobenzamide 16 in 85% overall yield. Substitution of the benzamide ring was then performed using a two-step strategy. The ring was initially brominated using *N*-bromosuccinimide (NBS) and the super Lewis acid catalyst iron(iii) triflimide (generated from FeCl_3_ and the ionic liquid [BMIM]NTf_2_),^20^ which allowed the regioselective synthesis of 17 in 77% yield. Introduction of the fluorinated (hetero)aryl groups was then achieved using the Suzuki–Miyaura cross-coupling reaction with various boronic acids.^21^ This gave compounds 8a–8e in 18–71% yields.

### Physicochemical and binding studies

The physicochemical properties of both sets of compounds was initially performed to assess their potential as brain imaging agents. The calculated partition coefficient (log *P*), the permeability (*P*_m_), the membrane partition coefficient (*K*_m_) and the percentage of plasma protein binding (%PPB) were determined for each compound using established methods (see ESI,† Table S1).^22,23^ As expected, the biaryl amines showed high plasma protein binding, with calculated log *P* values from 3.41–5.57. The permeability and membrane partition coefficient for the majority of compounds were well within the ideal limits for these parameters (*P*_m_ < 0.5; *K*_m_ < 250), which are based on known CNS imaging agents.^23^

A GTPγS assay was then conducted to evaluate receptor agonist activity of each compound at nine concentrations from 100 fM to 10 μM (*n* = 3).^24,25^ Initially, all compounds were tested for agonist activity with S1P_5_ and relative efficacy *versus* S1P (Table 1).^26^ Compounds which showed an EC_50_ value below 40 nM for S1P_5_, were then assessed against S1P_1_, S1P_2_ and S1P_3_. Previous work by the Novartis group described the importance of hydrogen bonding between the carboxamide and 2-substituent, to maintain an in-plane conformation of the amide that was critical for agonist activity against S1P receptors.^16^ Similar results were observed in this study. The use of relatively bulky alkoxy groups (*e.g.*7a, 7c, 7d and 7h) that likely disrupt hydrogen bonding to the amide showed loss of agonist activity with S1P_5_. This was more pronounced for compounds with both a bulky alkoxy group and a 3-substituent, with 6-arylaminobenzamide 7h found to have the weakest activity. From this library, five compounds were found to have strong S1P_5_ activity and were screened against the other receptors. Compound 7f with a 2-allyloxy group was found to have the highest S1P_5_ agonist activity, while 8e with a 2-fluoropyridin-3-yl motif at the 2-position was also found to have good activity. However, both compounds showed high activity for the other receptors, particularly, 8e with a EC_50_ value of 0.336 nM against S1P_1_. Of the other compounds, 7b and 7g were found to possess high activity for S1P_5_, while showing good selectivity against the other receptors. Based on the binding studies and the physicochemical results, 6-arylaminobenzamide 7g, named as TEFM78 was chosen as the lead compound. As well as high activity and good selectivity for S1P_5_, TEFM78 possessed ideal *P*_m_ (0.233) and *K*_m_ (70.5) values and one of the lowest log *P* values of 3.90.

**Table 1:** Binding studies of 7a–7h and 8a–8e with S1P_5_ and other S1P receptors (*n* = 3, errors in parentheses are derived from 95% confidence intervals)

	Efficacy (%)	EC_50_ (nM)			
		S1P_5_	S1P_1_	S1P_2_	S1P_3_
**S1P (1)**	100	10.7 (3.56)	—	—	42.6 (21.8)
7a	105	419 (159)	—	—	—
7b	108	39.8 (9.92)	>10 000	>10 000	>10 000
7c	137	180 (87.7)	—	—	—
7d	140	234 (169)	—	—	—
7e	102	49.0 (97.4)	—	—	—
7f	164	6.66 (3.09)	25.58 (1549)	>10 000	27.64 (106.2)
7g	183	22.3 (5.20)	104 (11 428)	>10 000	2030 (5239)
7h	154	2238 (3589)	—	—	—
8a	129	72.3 (46.3)	—	—	—
8b	136	954 (514)	—	—	—
8c	120	29.8 (24.0)	76.0 (691)	>10 000	>10 000
8d	142	612 (1262)	—	—	—
8e	175	23.4 (21.3)	0.336 (6.458)	>10 000	172 (102)

### Docking studies

**Fig. 2:**
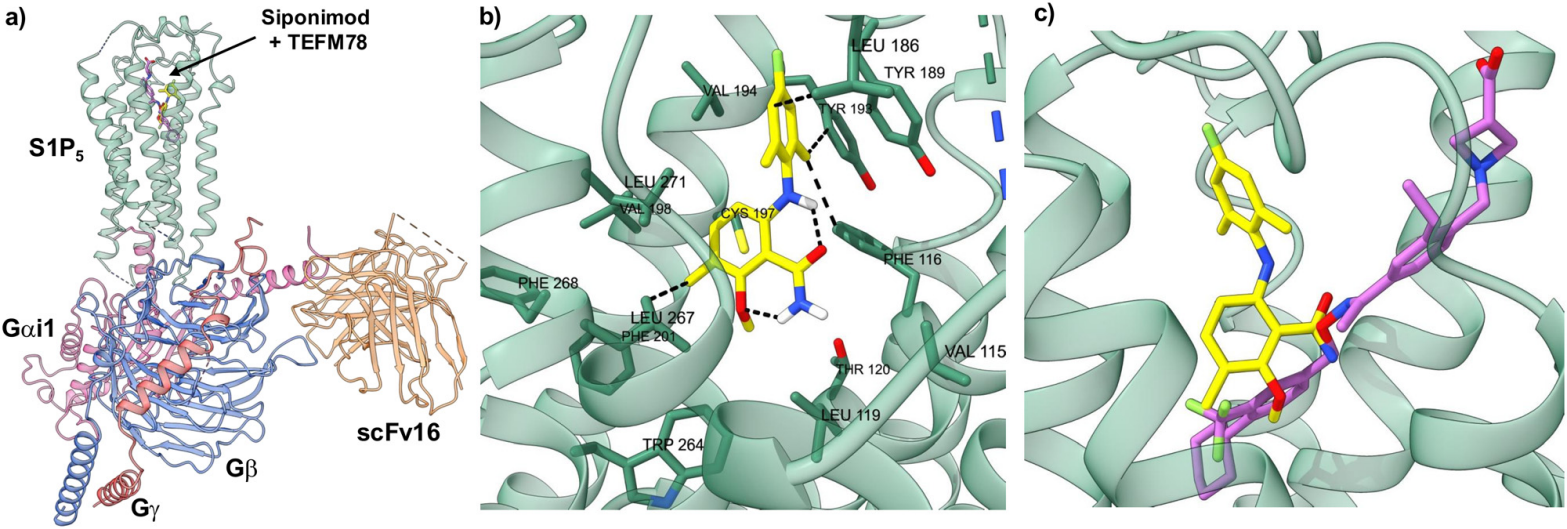
Docking studies of TEFM78 (7g) with the cryo-EM structure of S1P_5_. a) The cryo-EM structure of S1P_5_-Gi heterotrimer-scFv16 complex with Siponimod (purple) and docked TEFM78 (yellow). b) Close up view of docked TEFM78 showing key interactions. c) Close up view of the Siponimod (purple structure) binding site with docked TEFM78 (yellow structure).

To determine how TEFM78 (7g) binds to S1P_5_, docking studies were performed. Previously, Mattes and co-workers used homology models based on bovine rhodopsin for docking studies.^16^ However, in the last few years, structures of S1P_5_ with bound agonists have been reported and these have been used to understand Gi protein coupling, the mechanism of activation and drug recognition.^27–29^ For this study, we chose the cryo-electron microscopy (EM) structure of the S1P_5_-Gi heterotrimer-scFV16 complex with bound agonist, Siponimod (4) (PDB code: 7EW1).^29,30^ Docking studies with this complex showed that TEFM78 binds in the same orthosteric site as S1P and other agonists (Fig. 2a).^27^ This is composed of an upper hydrophilic pocket that binds the phosphonate group of S1P and a lower hydrophobic region, which accommodates the hydrocarbon chain. In their docking and SAR studies, Mattes and co-workers proposed that a twisted 6-arylaminobenzamide structure with hydrogen bonding between the aniline and amide was critical for affinity with S1P_5_. Our docking studies were found to corroborate this view. TEFM78 adopts a twisted conformation with a 72° angle between the two aryl rings and intramolecular hydrogen bonding involving the amide carbonyl and aniline NH moiety (1.95 Å), as well as the 2-methoxy substituent and amide NH_2_ group (1.82 Å) (Fig. 2b). Both aryl rings occupy hydrophobic pockets with multiple Van der Waals contacts. For example, the fluorophenyl moiety has close contacts with Leu186, Tyr193 and Phe116, while the benzamide moiety residues in a pocket composed of various amino acid residues such as leucine, valine and tryptophan. In comparison to other agonists, the benzamide moiety of TEFM78 occupies a similar region as the lipophilic end of Siponimod (4) (Fig. 2c). More specifically, the 2-methoxy and 3-methyl substituents of TEFM78 reside in the same lipophilic sub-pocket as the trifluoromethyl group of Siponimod. The alkoxy groups of other agonists such as Cenerimod and Ozanimod also bind in this sub-pocket.^29^ There is also overlap between the TEFM78 amide and the Siponimod oxime. The main difference between the two structures is caused by the twisting of the 6-arylaminobenzamide, which positions the fluorophenyl moiety in a more preferred lipophilic cavity. These docking studies give an insight into the binding of TEFM78 with S1P_5_ and provide a model for the design of future analogues.

### Radiochemistry

**Scheme 3:**
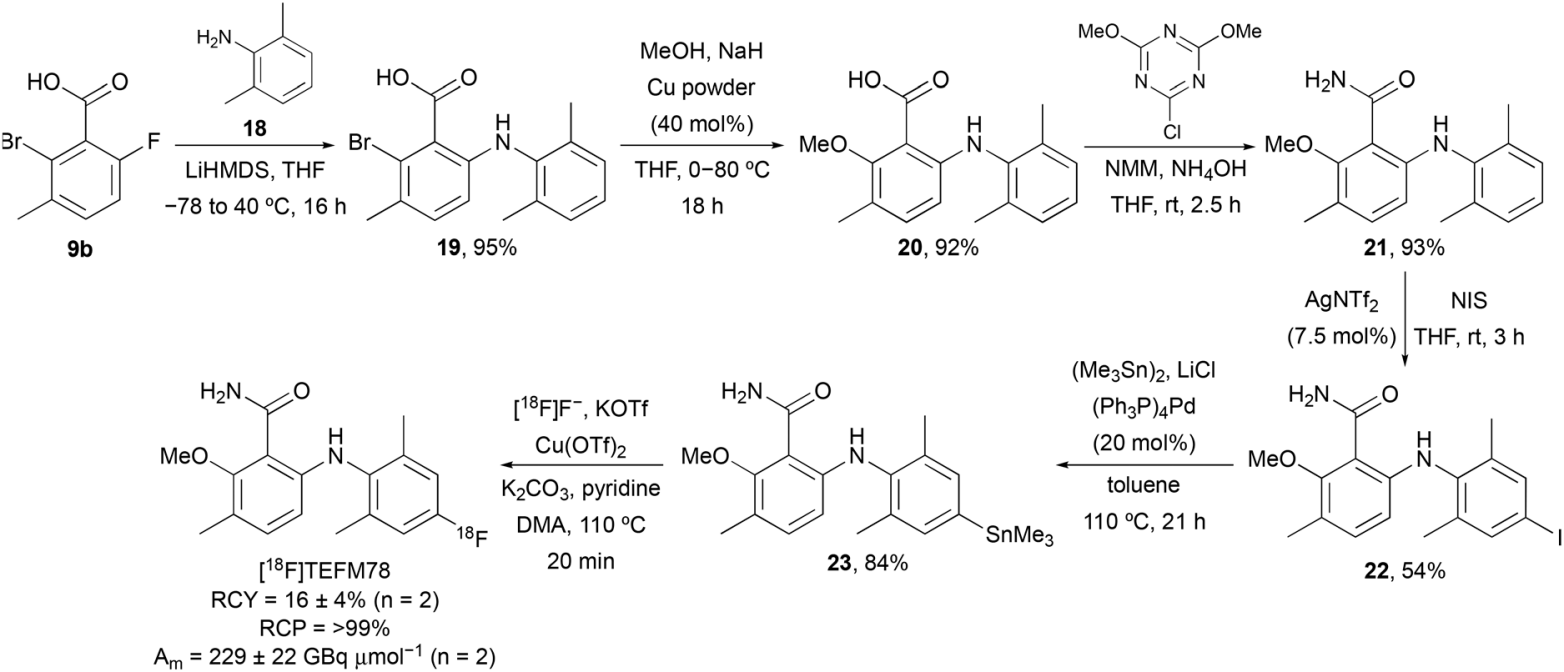
Synthesis of [^18^F]TEFM78.

On identification of TEFM78 as the lead compound, a synthetic route to access a suitable precursor for late stage radiofluorination was developed (Scheme 3). Our approach focused on the synthesis of a 6-arylaminobenzamide that could undergo late-stage functionalisation at the 4′-position for subsequent radiofluorination. Nucleophilic aromatic substitution of 9b with 2,6-dimethylaniline (18), followed by Ullmann coupling and amidation gave benzamide 21 in 81% yield over the three steps. Activation of the electron-rich aniline ring was achieved using silver(i) triflimide-catalysed iodination.^17^ Following optimisation (solvent and reaction time), this gave 22 in 54% yield. Subsequent Pd(0)-catalysed stannylation gave trimethyltin precursor 23 in 84% yield. The radiosynthesis of [^18^F]TEFM78 was then performed using a TRACERlab FX_FN_ automated synthesiser. No-carrier-added [^18^F]fluoride from the cyclotron was trapped on a carbonate-pre-conditioned quaternary methyl ammonium (QMA) cartridge, eluted into the reaction vessel and then azeotropically dried. Radiofluorination studies with 23 and using Cu(OTf)_2_ identified a temperature of 110 °C and a 20 minute reaction time as optimal. Following semi-preparative HPLC purification, [^18^F]TEFM78 was isolated in 16 ± 4% radiochemical yield (RCY), >99% radiochemical purity (RCP) and with a molar activity (*A*_m_) of 229 ± 22 GBq μmol^−1^, starting from 75.3 ± 2.2 GBq of [^18^F]fluoride. The total synthesis time of [^18^F]TEFM78 from delivery of [^18^F]fluoride to extraction of product was 62 minutes. [^18^F]TEFM78 was found to be highly stable by both radio-HPLC and radio-TLC with >99% RCP ten hours after production.

### Pilot PET *in vivo* imaging studies with [^18^F]TEFM78

**Fig. 3:**
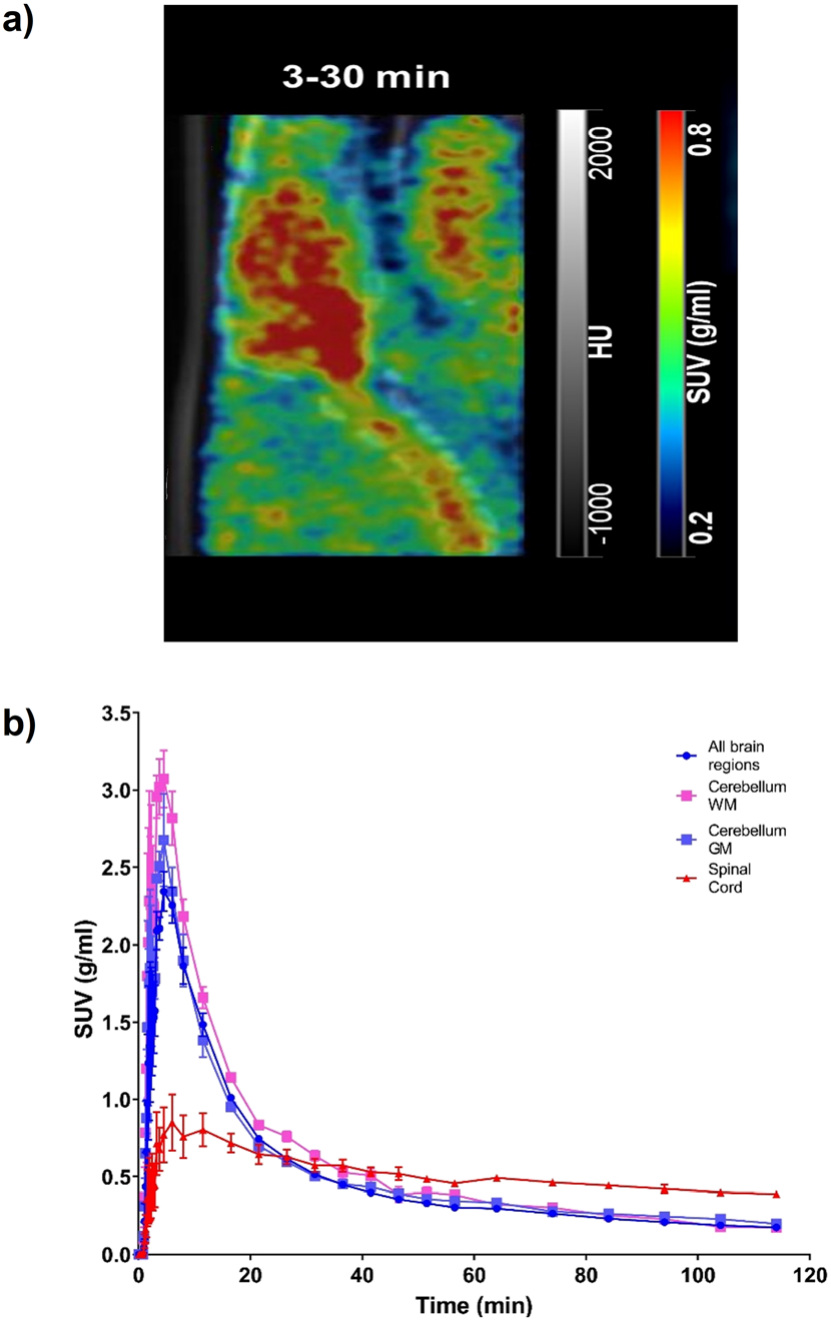
Brain and spinal cord kinetics of [^18^F]TEFM78 in naïve rats. The molar activity of [^18^F]TEFM78 used in this study was 50.2 ± 14 GBq mmol^−1^. a) Representative SUV PET image average between 3 and 30 minutes post-intravenous bolus injection showing good brain penetration and spinal cord uptake. The PET image was overlaid onto the CT scan. b) Average [^18^F]TEFM78 time activity curves for whole brain, whole grey matter, whole white matter and spinal cord in rats showing faster clearance from the brain compared with the spinal cord. Data expressed as SUV and presented as mean ± SEM, *n* = 3.

Following successful preparation of [^18^F]TEFM78, PET imaging with rodents was next conducted. On intravenous bolus administration of [^18^F]TEFM78, there was rapid uptake of the radiotracer into the rat brain (Fig. 3a), followed by clearance within 20 minutes of the scan starting (Fig. 3b). A peak standard uptake value (SUV) of 3 g mL^−1^ was measured in the brain, demonstrating good brain penetration (typical recommended peak SUV for successful brain PET ligands should be >1 g mL^−1^).^31^ The biodistribution and kinetics of the spinal cord were different from the brain with a lower peak radiotracer uptake but slower clearance (Fig. 3b). This initial data showed that [^18^F]TEFM78 can penetrate the mammalian brain and display an *in vivo* kinetics profile compatible with the half-lives of PET imaging radioisotopes. Typically, low free fraction of compounds can limit brain penetration. However, as shown in the PET images presented in Fig. 3, despite low free fraction, brain penetration of [^18^F]TEFM78 is successfully achieved. This demonstrates that, albeit low, the measured radiotracer free fraction does not preclude blood brain barrier penetration and successful brain PET imaging in rats. Notwithstanding, further preclinical studies are needed to investigate radiotracer brain kinetics in more detail and it is possible that higher free fractions would be required for successful human brain imaging.

## Conclusions

In summary, various fluorinated 6-arylaminobenzamides have been prepared and evaluated as potential PET imaging agents of the S1P_5_ receptor. One series with a 4′-fluorophenylamino group examined the SAR of the 3-alkoxy substituent of the benzamide ring, while the second series investigated the incorporation of the fluorine atom through an additional 4-aryl benzamide substituent. Binding evaluation using a [^35^S]GTPγS assay against the main S1P receptors identified TEFM78 (7g) as a selective and high affinity agonist of S1P_5_. Physicochemical analysis of TEFM78 revealed that this compound possessed ideal *P*_m_ and *K*_m_ values and a good log *P* value of 3.90. Based on these results, a synthetic route for the preparation of [^18^F]TEFM78 was developed, with the resulting imaging agent used in pilot PET imaging studies with rodents. These studies showed fast uptake in rat brain with clearance within 20 minutes. Based on these encouraging results, future work will focus on further *in vivo* assessment of [^18^F]TEFM78, including biodistribution studies with disease models.

## Experimental

### General experimental

All reagents and starting materials were obtained from commercial sources and used as received. All reactions were performed under an atmosphere of air unless otherwise stated. All dry solvents were purified using a PureSolv 500 MD solvent purification system. Brine is defined as a saturated solution of aqueous sodium chloride. Flash column chromatography was carried out using Merck Geduran Si 60 (40–63 μm). Merck aluminium-backed plates pre-coated with silica gel 60 (UV_254_) were used for thin layer chromatography and were visualised under ultraviolet light and by staining with KMnO_4_, ninhydrin or vanillin. ^1^H NMR and ^13^C NMR spectra were recorded on a Bruker DPX 400 spectrometer or Bruker 500 spectrometer with chemical shift values in ppm relative to tetramethylsilane (*δ*_H_ 0.00 and *δ*_C_ 0.0) or residual chloroform (*δ*_H_ 7.26 and *δ*_C_ 77.2) as the standard. Assignment of ^1^H and ^13^C NMR signals are based on 2-dimensional COSY, HSQC and DEPT experiments. Infrared spectra were recorded using a Shimadzu FTIR-84005 spectrometer and mass spectra were obtained using a JEOL JMS-700 spectrometer or a Bruker MicroTOFq high resolution mass spectrometer. Melting points were determined on a Gallenkamp melting point apparatus. Purity of all final compounds is >95%, as determined by HPLC.

#### General procedure A: nucleophilic aromatic substitution of 2-fluorobenzoic acids

The benzoic acid (1.0 equiv.) was dissolved in tetrahydrofuran (4.5 mL mmol^−1^) under an atmosphere of argon. The aniline (2.0 equiv.) was added and the mixture cooled to −78 °C. Lithium bis(trimethylsilyl)amide (3.0 equiv.; 1.0 M in tetrahydrofuran) was then slowly added over 0.1 h. The reaction mixture was then allowed to reach room temperature before heating to 40 °C and stirring for 16 h. The reaction was quenched with water (2.5 mL mmol^−1^) and acidified to pH 2 with 10% aqueous hydrochloric acid. The mixture was then extracted with ethyl acetate (3 × 10 mL), dried (MgSO_4_), filtered and concentrated *in vacuo*. The resulting residue was then purified by flash column chromatography, eluting with 20–40% ethyl acetate in petroleum ether (40–60) to afford the benzoic acid derivative.

#### General procedure B: Ullmann condensation of 2-bromobenzoic acids

Sodium hydride (3.0 equiv.; 60% in mineral oil) was added to a dry flask under argon. The sodium hydride was then washed with hexane (2 × 5 mL mmol^−1^) to remove the oil and the flask cooled to 0 °C. The required dry alcohol (3.3 mL mmol^−1^) was added to the reaction vessel carefully over 0.1 h and then stirred for 0.5 h. The benzoic acid derivative (1.0 equiv.) was added followed by the addition of copper powder (0.4 equiv.) and the reaction mixture heated to 80 °C for 18 h. The reaction mixture was cooled to room temperature and filtered through a pad of Celite®. The filtrate was concentrated *in vacuo* and dissolved in water (3.3 mL mmol^−1^). The mixture was acidified to pH 2 using 10% aqueous hydrochloric acid and extracted with dichloromethane (3 × 5.0 mL mmol^−1^). The organic layer was dried (MgSO_4_), filtered and concentrated *in vacuo*. The resulting residue was then purified by flash column chromatography, eluting with 20% ethyl acetate in petroleum ether (40–60) to afford the ether product.

#### General procedure C: amidation of benzoic acid derivatives

The benzoic acid derivative (1.0 equiv.) was dissolved in tetrahydrofuran (3.0 mL mmol^−1^) and 2-chloro-4,6-dimethoxy-1,3,5-triazine (1.2 equiv.) and *N*-methylmorpholine (3.0 equiv.) were added. The mixture was then stirred for 2 h at room temperature. The precipitate was filtered and ammonium hydroxide (9.0 mL mmol^−1^) was added to the filtrate. The reaction mixture was stirred for 0.5 h at room temperature and then filtered. 2 M sodium hydroxide (9.0 mL mmol^−1^) was added to the filtrate and the crude product was extracted with ethyl acetate (3 × 9.0 mL mmol^−1^). The organic layer was dried (MgSO_4_), filtered and concentrated *in vacuo*. The resulting residue was then purified by flash column chromatography, eluting with 10% ethyl acetate in petroleum ether (40–60) to afford the benzamide product.

#### General procedure D: Suzuki–Miyaura reaction of 3-bromobenzamides

To a reaction vessel containing the aryl bromide (1.0 equiv.) in 1,4-dioxane (15 mL mmol^−1^) and water (1.1 mL mmol^−1^) was added [1,1′-bis(diphenylphosphino)ferrocene]dichloropalladium(ii) (1 : 1) (5 mol%), the boronic acid (1.6 equiv.) and caesium carbonate (3.0 equiv.). The reaction mixture was then sealed and degassed under argon for 0.2 h, before heating to 80 °C for 2–16 h. The reaction mixture was cooled, filtered through Celite® and washed with ethyl acetate (50 mL mmol^−1^). The filtrate was washed with water (3 × 50 mL mmol^−1^) and brine (50 mL mmol^−1^) and the organic layer dried (MgSO_4_), filtered and concentrated *in vacuo*. The resulting residue was then purified by flash column chromatography to afford the coupled product.

#### 2-Bromo-6-[(4′-fluoro-2′,6′-dimethylphenyl)amino]benzoic acid (11a)

The reaction was carried out according to general procedure A and gave 2-bromo-6-[(4′-fluoro-2′,6′-dimethylphenyl)amino]benzoic acid (11a) as a brown solid (1.45 g, 95%). Mp 148–150 °C; IR (neat) 2920, 1672, 1595, 1560, 1481, 1439, 1242, 858, 767 cm^−1^; ^1^H NMR (500 MHz, CDCl_3_): *δ* 11.19 (br s, 1H, OH), 7.78 (br s, 1H, NH), 7.00 (dd, 1H, *J* = 7.8, 1.4 Hz, 3-H), 6.97 (t, 1H, *J* = 7.8 Hz, 4-H), 6.85 (d, 2H, *J* = 9.0 Hz, 3′-H and 5′-H), 6.15 (dd, 1H, *J* = 7.8, 1.4 Hz, 5-H), 2.18 (s, 6H, 2′-CH_3_ and 6′-CH_3_); ^13^C{^1^H} NMR (126 MHz, CDCl_3_): *δ* 172.1 (C), 161.0 (C, d, ^1^*J*_CF_ = 245.3 Hz), 150.1 (C), 138.9 (2× C, d, ^3^*J*_CF_ = 8.9 Hz), 133.7 (CH), 132.5 (C, d, ^4^*J*_CF_ = 2.8 Hz), 123.6 (CH), 123.2 (C), 115.1 (2× CH, d, ^2^*J*_CF_ = 21.9 Hz), 113.2 (C), 111.9 (CH), 18.39 (CH_3_), 18.38 (CH_3_); MS (EI) *m*/*z* 337 (M^+^, 84%), 319 (72), 293 (58), 212 (100), 198 (37); HRMS (EI) *m*/*z*: [M]^+^ calcd for C_15_H_13_^79^BrFNO_2_ 337.0114; found 337.0122.

#### 2-Bromo-6-[(4′-fluoro-2′,6′-dimethylphenyl)amino]-3-methylbenzoic acid (11b)

The reaction was carried out according to general procedure A and gave 2-bromo-6-[(4′-fluoro-2′,6′-dimethylphenyl)amino]benzoic acid (11b) as a brown solid (0.120 g, 79%). Mp 130–132 °C; IR (neat) 2924, 1696, 1606, 1491, 1215, 1129, 1019, 862, 757 cm^−1^; ^1^H NMR (400 MHz, CDCl_3_): *δ* 7.02 (d, 1H, *J* = 8.5 Hz, 4-H), 6.83 (d, 2H, *J* = 9.0 Hz, 3′-H and 5′-H), 6.09 (d, 1H, *J* = 8.5 Hz, 5-H), 2.33 (s, 3H, 3-CH_3_), 2.17 (s, 6H, 2′-CH_3_ and 6′-CH_3_); ^13^C{^1^H} NMR (101 MHz, CDCl_3_): *δ* 173.2 (C), 160.7 (C, d, ^1^*J*_CF_ = 245.0 Hz), 146.2 (C), 138.6 (2× C, d, ^3^*J*_CF_ = 8.6 Hz), 134.1 (CH), 133.0 (C, d, ^4^*J*_CF_ = 2.8 Hz), 128.1 (C), 124.2 (C), 116.8 (C), 115.0 (2× CH, d, ^2^*J*_CF_ = 21.8), 112.0 (CH), 23.0 (CH_3_), 18.37 (CH_3_), 18.36 (CH_3_); MS (ESI) *m*/*z* 350 ([M–H]^−^, 100%); HRMS (ESI) *m*/*z*: [M–H]^−^ calcd for C_16_H_14_^79^BrFNO_2_ 350.0197; found 350.0197.

#### 6-[(4′-Fluoro-2′,6′-dimethylphenyl)amino]-2-isopropoxybenzoic acid (12a)

The reaction was carried out according to general procedure B and gave 6-[(4′-fluoro-2′,6′-dimethylphenyl)amino]-2-isopropoxybenzoic acid (12a) as an orange solid (0.062 g, 44%). Mp 99–101 °C; IR (neat) 3250, 2982, 1697, 1578, 1458, 1252 cm^−1^; ^1^H NMR (400 MHz, CDCl_3_): *δ* 12.04 (br s, 1H, OH), 9.89 (br s, 1H, NH), 7.10 (t, 1H, *J* = 8.4 Hz, 4-H), 6.83 (d, *J* = 9.0 Hz, 3′-H and 5′-H), 6.24 (br d, 1H, *J* = 8.4 Hz, 3-H), 5.87 (dd, *J* = 8.4, 0.8 Hz, 5-H), 4.83 (sept, 1H, *J* = 6.1 Hz, OC*H*(CH_3_)_2_), 2.16 (s, 6H, 2′-CH_3_ and 6′-CH_3_), 1.51 (d, 6H, *J* = 6.1 Hz, OCH(C*H*_3_)_2_); ^13^C{^1^H} NMR (101 MHz, CDCl_3_): *δ* 169.3 (C), 160.9 (C, d, ^1^*J*_CF_ = 244.9 Hz), 158.3 (C), 152.7 (C), 139.2 (2× C, d, ^3^*J*_CF_ = 8.7 Hz), 134.2 (CH), 133.0 (C, d, ^4^*J*_CF_ = 2.8 Hz), 114.8 (2× CH, d, ^2^*J*_CF_ = 21.7 Hz), 106.8 (CH), 100.5 (CH), 100.0 (C), 74.3 (CH), 22.1 (2× CH_3_), 18.39 (CH_3_), 18.37 (CH_3_); MS (ESI) *m*/*z* 340 (M + Na^+^, 100%); HRMS (ESI) *m*/*z*: [M + Na]^+^ calcd for C_18_H_20_FNNaO_3_ 340.1319; found 340.1306.

#### 6-[(4′-Fluoro-2′,6′-dimethylphenyl)amino]-2-methoxybenzoic acid (12b)

The reaction was carried out according to general procedure B and gave 6-[(4′-fluoro-2′,6′-dimethylphenyl)amino]-2-methoxybenzoic acid (12b) as a brown solid (0.031 g, 71%). Mp 100–101 °C; IR (neat) 3246, 2953, 1695, 1577, 1464, 1443, 1255, 1084, 806 cm^−1^; ^1^H NMR (500 MHz, CDCl_3_): *δ* 11.50 (br s, 1H, OH), 9.90 (br s, 1H, NH), 7.13 (t, 1H, *J* = 8.4 Hz, 4-H), 6.83 (d, 2H, *J* = 9.0 Hz, 3′-H and 5′-H), 6.25 (dd, 1H, *J* = 8.4, 0.9 Hz, 3-H), 5.91 (dd, 1H, *J* = 8.4, 0.9 Hz, 5-H), 4.05 (s, 3H, OCH_3_), 2.15 (s, 6H, 2′-CH_3_ and 6′-CH_3_); ^13^C{^1^H} NMR (126 MHz, CDCl_3_): *δ* 169.0 (C), 160.9 (C, d, ^1^*J*_CF_ = 245.0 Hz), 159.9 (C), 152.7 (C), 139.2 (2× C, d, ^3^*J*_CF_ = 8.9 Hz), 134.4 (CH), 132.9 (C, d, ^4^*J*_CF_ = 2.7 Hz), 114.8 (2× CH, d, ^2^*J*_CF_ = 21.9 Hz), 107.2 (CH), 98.8 (C), 98.0 (CH), 56.9 (CH_3_), 18.36 (CH_3_), 18.35 (CH_3_); MS (ESI) *m*/*z* 312 (M + Na^+^, 100%); HRMS (ESI) *m*/*z*: [M + Na]^+^ calcd for C_16_H_16_FNNaO_3_ 312.1006; found 312.0994.

#### 6-[(4′-Fluoro-2′,6′-dimethylphenyl)amino]-2-isobutoxybenzoic acid (12c)

The reaction was carried out according to general procedure B and gave 6-[(4′-fluoro-2′,6′-dimethylphenyl)amino]-2-isobutoxybenzoic acid (12c) as an orange solid (0.033 g, 68%). Mp 74–76 °C; IR (neat) 3255, 2966, 1695, 1577, 1456, 1407, 1252, 1057, 756 cm^−1^; ^1^H NMR (500 MHz, CDCl_3_): *δ* 11.77 (br s, 1H, OH), 9.90 (br s, 1H, NH), 7.10 (t, 1H, *J* = 8.3 Hz, 4-H), 6.83 (d, 2H, *J* = 9.1 Hz, 3′-H and 5′-H), 6.22 (dd, 1H, *J* = 8.3, 0.9 Hz, 3-H), 5.89 (dd, 1H, *J* = 8.3, 0.9 Hz, 5-H), 4.00 (d, 2H, *J* = 6.4 Hz, 1′′-H_2_), 2.29–2.19 (m, 1H, 2′′-H), 2.16 (s, 6H, 2′-CH_3_ and 6′-CH_3_), 1.11 (d, 6H, *J* = 6.4 Hz, 2′′-CH_3_ and 3′′-H_3_); ^13^C{^1^H} NMR (126 MHz, CDCl_3_): *δ* 169.1 (C), 160.9 (C, d, ^1^*J*_CF_ = 244.6 Hz), 159.5 (C), 152.6 (C), 139.2 (2× C, d, ^3^*J*_CF_ = 8.7 Hz), 134.4 (CH), 132.9 (C, d, ^4^*J*_CF_ = 2.8 Hz), 114.8 (2× CH, d, ^2^*J*_CF_ = 21.6 Hz), 106.9 (CH), 98.91 (CH), 98.87 (C), 76.7 (CH_2_), 28.1 (CH), 19.2 (2× CH_3_), 18.37 (CH_3_), 18.36 (CH_3_); MS (ESI) *m*/*z* 354 (M + Na^+^, 100%); HRMS (ESI) *m*/*z*: [M + Na]^+^ calcd for C_19_H_22_FNNaO_3_ 354.1476; found 354.1465.

#### 2-*n*-Butoxy-6-[(4′-fluoro-2′,6′-dimethylphenyl)amino]benzoic acid (12d)

The reaction was carried out according to general procedure B and gave 2-*n*-butoxy-6-[(4′-fluoro-2′,6′-dimethylphenyl)amino]benzoic acid (12d) as a brown solid (0.033 g, 70%). Mp 77–79 °C; IR (neat) 3246, 2963, 1694, 1578, 1456, 1408, 1252, 1219, 754 cm^−1^; ^1^H NMR (500 MHz, CDCl_3_): *δ* 11.78 (br s, 1H, OH), 9.90 (br, 1H, NH), 7.10 (t, 1H, *J* = 8.3 Hz, 4-H), 6.83 (d, 2H, *J* = 9.2 Hz, 3′-H and 5′-H), 6.23 (dd, 1H, *J* = 8.3, 0.8 Hz, 3-H), 5.89 (dd, 1H, *J* = 8.3, 0.8 Hz, 5-H), 4.22 (t, 2H, *J* = 6.5 Hz, 1′′-H_2_), 2.15 (s, 6H, 2′-CH_3_ and 6′-CH_3_), 1.95–1.87 (m, 2H, 2′′-H_2_), 1.60–1.49 (m, 2H, 3′′-H_2_), 1.02 (t, 3H, *J* = 7.4 Hz, 4′′-H_3_); ^13^C{^1^H} NMR (126 MHz, CDCl_3_): *δ* 169.1 (C), 160.9 (C, d, ^1^*J*_CF_ = 244.6 Hz), 159.4 (C), 152.6 (C), 139.2 (2× C, d, ^3^*J*_CF_ = 8.9 Hz), 134.4 (CH), 132.9 (C, d, ^4^*J*_CF_ = 2.7 Hz), 114.8 (2× CH, d, ^2^*J*_CF_ = 21.9 Hz), 106.9 (CH), 99.0 (CH and C), 70.2 (CH_2_), 30.9 (CH_2_), 19.2 (CH_2_), 18.37 (CH_3_), 18.36 (CH_3_), 13.7 (CH_3_); MS (ESI) *m*/*z* 354 (M + Na^+^, 100%); HRMS (ESI) *m*/*z*: [M + Na]^+^ calcd for C_19_H_22_FNNaO_3_ 354.1476; found 354.1466.

#### 6-[(4′-Fluoro-2′,6′-dimethylphenyl)amino]-2-(3′′-methyl-*n*-butoxy)benzoic acid (12e)

The reaction was carried out according to general procedure B and gave 6-[(4′-fluoro-2′,6′-dimethylphenyl)amino]-2-(3′′-methyl-*n*-butoxy)benzoic acid (12e) as a brown viscous oil (0.020 g, 39%). IR (neat) 3244, 2959, 1694, 1578, 1458, 1408, 1252, 1217, 1076, 754 cm^−1^; ^1^H NMR (400 MHz, CDCl_3_): *δ* 11.69 (br s, 1H, OH), 9.90 (br s, 1H, NH), 7.11 (t, 1H, *J* = 8.3 Hz, 4-H), 6.83 (d, 2H, *J* = 9.0 Hz, 3′-H and 5′-H), 6.24 (br d, 1H, *J* = 8.3 Hz, 3-H), 5.89 (dd, 1H, *J* = 8.3, 0.8 Hz, 5-H), 4.24 (t, 2H, *J* = 6.5 Hz, 1′′-H_2_), 2.16 (s, 6H, 2′-CH_3_ and 6′-CH_3_), 1.91–1.77 (m, 3H, 2′′-H_2_ and 3′′-H), 1.01 (d, 6H, *J* = 6.4 Hz, 3′′-CH_3_ and 4′′-H_3_); ^13^C{^1^H} NMR (126 MHz, CDCl_3_): *δ* 169.1 (C), 160.9 (C, d, ^1^*J*_CF_ = 244.8 Hz), 159.5 (C), 152.7 (C), 139.2 (2× C, d, ^3^*J*_CF_ = 8.4 Hz), 134.3 (CH), 133.0 (C, d, ^4^*J*_CF_ = 2.7 Hz), 114.8 (2× CH, d, ^2^*J*_CF_ = 21.9 Hz), 107.0 (CH), 99.03 (C), 98.98 (CH), 69.0 (CH_2_), 37.7 (CH_2_), 25.1 (CH), 22.5 (2× CH_3_), 18.37 (CH_3_), 18.36 (CH_3_); MS (ESI) *m*/*z* 368 (M + Na^+^, 100%); HRMS (ESI) *m*/*z*: [M + Na]^+^ calcd for C_20_H_24_FNNaO_3_ 368.1632; found 368.1620.

#### 2-(Allyloxy)-6-[(4′-fluoro-2′,6′-dimethylphenyl)amino]benzoic acid (12f)

The reaction was carried out according to general procedure B and gave 2-(allyloxy)-6-[(4′-fluoro-2′,6′-dimethylphenyl)amino]benzoic acid (12f) as a brown viscous oil (0.0643 g, 46%). IR (neat) 3258, 2920, 1694, 1576, 1456, 1252, 1051, 806 cm^−1^; ^1^H NMR (500 MHz, CDCl_3_): *δ* 11.60 (br s, 1H, OH), 9.89 (br s, 1H, NH), 7.10 (t, 1H, *J* = 8.2 Hz, 4-H), 6.83 (d, 2H, *J* = 9.1 Hz, 3′-H and 5′-H), 6.23 (d, 1H, *J* = 8.2 Hz, 3-H), 6.11 (ddt, 1H, *J* = 17.0, 10.5, 5.8 Hz, 2′′-H), 5.91 (d, 1H, *J* = 8.2 Hz, 5-H), 5.51 (dd, 1H, *J* = 17.0, 0.8 Hz, 3′′-*H*H), 5.45 (dd, 1H, *J* = 10.5, 0.8 Hz, 3′′-H*H*), 4.76 (br d, 2H, *J* = 5.8 Hz, 1′′-H_2_), 2.15 (s, 6H, 2′-CH_3_ and 6′-CH_3_); ^13^C{^1^H} NMR (126 MHz, CDCl_3_): *δ* 169.0 (C), 160.9 (C, d, ^1^*J*_CF_ = 245.0 Hz), 159.1 (C), 152.7 (C), 139.2 (2× C, d, ^3^*J*_CF_ = 8.3 Hz), 134.3 (CH), 132.9 (C, d, ^4^*J*_CF_ = 2.7 Hz), 131.0 (CH), 120.7 (CH_2_), 114.8 (2× CH, ^2^*J*_CF_ = 21.8 Hz), 107.3 (CH), 99.4 (CH), 99.2 (C), 71.2 (CH_2_), 18.4 (2× CH_3_); MS (ESI) *m*/*z* 338 (M + Na^+^, 100%); HRMS (ESI) *m*/*z*: [M + Na]^+^ calcd for C_18_H_18_FNNaO_3_ 338.1163; found 338.1151.

#### 6-[(4′-Fluoro-2′,6′-dimethylphenyl)amino]-2-methoxy-3-methylbenzoic acid (12g)

The reaction was carried out according to general procedure B and gave 6-[(4′-fluoro-2′,6′-dimethylphenyl)amino]-2-methoxy-3-methylbenzoic acid (12g) as an orange viscous oil (0.0307 g, 71%). IR (neat) 3308, 2926, 1700, 1574, 1504, 1216, 757 cm^−1^; ^1^H NMR (500 MHz, CDCl_3_): *δ* 11.93 (br s, 1H, OH), 9.45 (br s, 1H, NH), 7.02 (d, 1H, *J* = 8.7 Hz, 4-H), 6.83 (d, 2H, *J* = 9.1 Hz, 3′-H and 5′-H), 5.98 (d, 1H, *J* = 8.7 Hz, 5-H), 3.93 (s, 3H, OCH_3_), 2.20 (s, 3H, 3-CH_3_), 2.15 (s, 6H, 2′-CH_3_ and 6′-CH_3_); ^13^C{^1^H} NMR (126 MHz, CDCl_3_): *δ* 168.7 (C), 160.9 (C, d, ^1^*J*_CF_ = 244.6 Hz), 158.3 (C), 150.3 (C), 139.2 (2× C, d, ^3^*J*_CF_ = 8.9 Hz), 137.2 (CH), 133.0 (C, d, ^4^*J*_CF_ = 2.7 Hz), 116.5 (C), 114.8 (2× CH, d, ^2^*J*_CF_ = 21.7 Hz), 109.8 (CH), 102.3 (C), 62.3 (CH_3_), 18.38 (CH_3_), 18.37 (CH_3_), 15.1 (CH_3_); MS (ESI) *m*/*z* 326 (M + Na^+^, 100%); HRMS (ESI) *m*/*z*: [M + Na]^+^ calcd for C_17_H_18_FNNaO_3_ 326.1163; found 326.1148.

#### 6-[(4′-Fluoro-2′,6′-dimethylphenyl)amino]-2-isobutoxy-3-methylbenzoic acid (12h)

The reaction was carried out according to general procedure B and gave 6-[(4′-fluoro-2′,6′-dimethylphenyl)amino]-2-isobutoxy-3-methylbenzoic acid (12h) as an orange solid (0.073 g, 74%). Mp 130–132 °C; IR (neat) 3294, 2963, 1705, 1573, 1504, 1404, 1227, 1034, 818 cm^−1^; ^1^H NMR (400 MHz, CDCl_3_): *δ* 12.24 (br s, 1H, OH), 9.44 (br s, 1H, NH), 7.02 (d, 1H, *J* = 8.7 Hz, 4-H), 6.83 (d, 2H, *J* = 9.1 Hz, 3′-H and 5′-H), 5.97 (d, 1H, *J* = 8.7 Hz, 5-H), 3.76 (d, 2H, *J* = 6.7 Hz, 1′′-H_2_), 2.28–2.19 (m, 1H, 2′′-H), 2.18 (s, 3H, 3-CH_3_), 2.15 (s, 6H, 2′-CH_3_ and 6′-CH_3_), 1.12 (d, 6H, *J* = 6.7 Hz, 2′′-CH_3_ and 3′′-H_3_); ^13^C{^1^H} NMR (101 MHz, CDCl_3_): *δ* 168.7 (C), 160.9 (C, d, ^1^*J*_CF_ = 244.8 Hz), 157.2 (C), 150.3 (C), 139.2 (2× C, d, ^3^*J*_CF_ = 8.5 Hz), 137.1 (CH), 133.0 (C, d, ^4^*J*_CF_ = 2.7 Hz), 116.8 (C), 114.9 (2× CH, d, ^2^*J*_CF_ = 21.9 Hz), 109.6 (CH), 102.7 (C), 82.1 (CH_2_), 29.1 (CH), 19.0 (2× CH_3_), 18.36 (CH_3_), 18.35 (CH_3_), 15.2 (CH_3_); MS (ESI) *m*/*z* 368 (M + Na^+^, 100%); HRMS (ESI) *m*/*z*: [M + Na]^+^ calcd for C_20_H_24_FNNaO_3_ 368.1632; found 368.1619.

#### 6-[(4′-Fluoro-2′,6′-dimethylphenyl)amino]-2-isopropoxybenzamide (7a)

The reaction was carried out according to general procedure C and gave 6-[(4′-fluoro-2′,6′-dimethylphenyl)amino]-2-isopropoxybenzamide (7a) as a pale yellow solid (0.0380 g, 95%). Mp 204–206 °C; IR (neat) 3444, 3188, 2982, 2361, 1638, 1584, 1456, 1250, 1111, 1044 cm^−1^; ^1^H NMR (400 MHz, CDCl_3_): *δ* 10.13 (br s, 1H, NH), 8.16 (br s, 1H, NH), 7.01 (t, 1H, *J* = 8.3 Hz, 4-H), 6.82 (d, 2H, *J* = 9.2 Hz, 3′-H and 5′-H), 6.20 (br d, 1H, *J* = 8.3 Hz, 3-H), 5.80 (dd, 1H, *J* = 8.3, 0.9 Hz, 5-H), 5.65 (br s, 1H, NH), 4.70 (sept, 1H, *J* = 6.1 Hz, OC*H*(CH_3_)_2_), 2.18 (s, 6H, 2′-CH_3_ and 6′-CH_3_), 1.44 (d, 6H, *J* = 6.1 Hz, OCH(C*H*_3_)_2_); ^13^C{^1^H} NMR (126 MHz, CDCl_3_): *δ* 171.5 (C), 160.6 (C, d, ^1^*J*_CF_ = 244.0 Hz), 158.3 (C), 152.1 (C), 139.1 (2× C, d, ^3^*J*_CF_ = 8.2 Hz), 134.0 (C, d, ^4^*J*_CF_ = 2.7 Hz), 132.6 (CH), 114.6 (2× CH, d, ^2^*J*_CF_ = 21.8 Hz), 105.9 (CH), 103.2 (C), 100.9 (CH), 72.0 (CH), 22.3 (2× CH_3_), 18.49 (CH_3_), 18.48 (CH_3_); MS (ESI) *m*/*z* 339 (M + Na^+^, 100%); HRMS (ESI) *m*/*z*: [M + Na]^+^ calcd for C_18_H_21_FN_2_NaO_2_ 339.1479; found 339.1465.

#### 6-[(4′-Fluoro-2′,6′-dimethylphenyl)amino]-2-methoxybenzamide (7b)

The reaction was carried out according to general procedure C and gave 6-[(4′-fluoro-2′,6′-dimethylphenyl)amino]-2-methoxybenzamide (7b) as a pale yellow solid (0.0201 g, 67%). Mp 171–173 °C; IR (neat) 3462, 3186, 2924, 2359, 1638, 1580, 1458, 1250, 1128, 752 cm^−1^; ^1^H NMR (400 MHz, CDCl_3_): *δ* 10.17 (br s, 1H, NH), 7.94 (br s, 1H, NH), 7.05 (t, 1H, *J* = 8.4 Hz, 4-H), 6.82 (d, 2H, *J* = 9.0 Hz, 3′-H and 5′-H), 6.22 (dd, 1H, *J* = 8.4, 0.9 Hz, 3-H), 5.84 (dd, 1H, *J* = 8.4, 0.9 Hz, 5-H), 5.77 (br s, 1H, NH), 3.92 (s, 3H, OCH_3_), 2.17 (s, 6H, 2′-CH_3_ and 6′-CH_3_); ^13^C{^1^H} NMR (101 MHz, CDCl_3_): *δ* 171.3 (C), 160.6 (C, d, ^1^*J*_CF_ = 245.0 Hz), 160.0 (C), 152.0 (C), 139.1 (2× C, d, ^3^*J*_CF_ = 8.7 Hz), 133.9 (C, d, ^4^*J*_CF_ = 2.6 Hz), 132.8 (CH), 114.6 (2× CH, d, ^2^*J*_CF_ = 21.8 Hz), 106.4 (CH), 102.1 (C), 98.4 (CH), 56.0 (CH_3_), 18.5 (CH_3_), 18.4 (CH_3_); MS (ESI) *m*/*z* 311 (M + Na^+^, 100%); HRMS (ESI) *m*/*z*: [M + Na]^+^ calcd for C_16_H_17_FN_2_NaO_2_ 311.1166; found 311.1158.

#### 6-[(4′-Fluoro-2′,6′-dimethylphenyl)amino]-2-isobutoxybenzamide (7c)

The reaction was carried out according to general procedure C and gave 6-[(4′-fluoro-2′,6′-dimethylphenyl)amino]-2-isobutoxybenzamide (7c) as an orange solid (0.0285 g, 95%). Mp 152–154 °C; IR (neat) 3455, 3181, 2922, 2363, 1636, 1582, 1493, 1449, 1246, 1061, 864, 754 cm^−1^; ^1^H NMR (500 MHz, CDCl_3_): *δ* 10.14 (br s, 1H, NH), 8.06 (br s, 1H, NH), 7.02 (t, 1H, *J* = 8.5 Hz, 4-H), 6.82 (d, 2H, *J* = 9.1 Hz, 3′-H and 5′-H), 6.19 (br d, 1H, *J* = 8.5 Hz, 3-H), 5.82 (dd, 1H, *J* = 8.5, 0.8 Hz, 5-H), 5.73 (br s, 1H, NH), 3.86 (d, 2H, *J* = 6.4 Hz, 1′′-H_2_), 2.21–2.13 (m, 7H, 2′-CH_3_, 6′-CH_3_ and 2′′-H), 1.08 (d, 6H, *J* = 6.7 Hz, 2′′-CH_3_ and 3′′-H_3_); ^13^C{^1^H} NMR (126 MHz, CDCl_3_): *δ* 171.4 (C), 160.6 (C, d, ^1^*J*_CF_ = 243.6 Hz), 159.5 (C), 152.0 (C), 139.1 (2× C, d, ^3^*J*_CF_ = 8.4 Hz), 133.9 (C, d, ^4^*J*_CF_ = 2.7 Hz), 132.8 (CH), 114.6 (2× CH, d, ^2^*J*_CF_ = 21.4 Hz), 106.1 (CH), 102.1 (C), 99.3 (CH), 75.7 (CH_2_), 28.3 (CH), 19.5 (2× CH_3_), 18.47 (CH_3_), 18.46 (CH_3_); MS (ESI) *m*/*z* 353 (M + Na^+^, 100%); HRMS (ESI) *m*/*z*: [M + Na]^+^ calcd for C_19_H_23_FN_2_NaO_2_ 353.1636; found 353.1623.

#### 2-*n*-Butoxy-6-[(4′-fluoro-2′,6′-dimethylphenyl)amino]benzamide (7d)

The reaction was carried out according to general procedure C and gave 2-*n*-butoxy-6-[(4′-fluoro-2′,6′-dimethylphenyl)amino]benzamide (7d) as a pale orange solid (0.0244 g, 81%). Mp 156–158 °C; IR (neat) 3460, 3181, 2932, 1645, 1570, 1491, 1445, 1248, 752 cm^−1^; ^1^H NMR (500 MHz, CDCl_3_): *δ* 10.16 (br s, 1H, NH), 8.06 (br s, 1H, NH), 7.02 (t, 1H, *J* = 8.5 Hz, 4-H), 6.81 (d, 2H, *J* = 9.1 Hz, 3′-H and 5′-H), 6.20 (dd, 1H, *J* = 8.5, 0.8 Hz, 3-H), 5.82 (dd, 1H, *J* = 8.5, 0.8 Hz, 5-H), 5.70 (br s, 1H, NH), 4.09 (t, 2H, *J* = 6.5 Hz, 1′′-H_2_), 2.17 (s, 6H, 2′-CH_3_ and 6′-CH_3_), 1.90–1.81 (m, 2H, 2′′-H_2_), 1.57–1.47 (m, 2H, 3′′-H_2_), 1.00 (t, 3H, *J* = 7.5 Hz, 4′′-H_3_); ^13^C{^1^H} NMR (126 MHz, CDCl_3_): *δ* 171.5 (C), 160.6 (C, d, ^1^*J*_CF_ = 243.9 Hz), 159.5 (C), 152.0 (C), 139.1 (2× C, d, ^3^*J*_CF_ = 8.4 Hz), 134.0 (C, d, ^4^*J*_CF_ = 2.8 Hz), 132.8 (CH), 114.6 (2× CH, d, ^2^*J*_CF_ = 21.7 Hz), 106.1 (CH), 102.2 (C), 99.3 (CH), 69.0 (CH_2_), 31.3 (CH_2_), 19.5 (CH_2_), 18.45 (CH_3_), 18.44 (CH_3_), 13.8 (CH_3_); MS (ESI) *m*/*z* 353 (M + Na^+^, 100%); HRMS (ESI) *m*/*z*: [M + Na]^+^ calcd for C_19_H_23_FN_2_NaO_2_ 353.1636; found 353.1623.

#### 6-[(4′-Fluoro-2′,6′-dimethylphenyl)amino]-2-(3′′-methyl-*n*-butoxy)benzamide (7e)

The reaction was carried out according to general procedure C and gave 6-[(4′-fluoro-2′,6′-dimethylphenyl)amino]-2-(3′′-methyl-*n*-butoxy)benzamide (7e) as a pale yellow solid (0.014 g, 67%). Mp 143–145 °C; IR (neat) 3467, 3201, 2960, 1641, 1580, 1451, 1245, 1216, 754 cm^−1^; ^1^H NMR (500 MHz, CDCl_3_): *δ* 10.16 (br s, 1H, NH), 8.05 (br s, 1H, NH), 7.02 (t, 1H, *J* = 8.5 Hz, 4-H), 6.81 (d, 2H, *J* = 9.1 Hz, 3′-H and 5′-H), 6.21 (br d, 1H, *J* = 8.5 Hz, 3-H), 5.82 (dd, 1H, *J* = 8.5, 0.8 Hz, 5-H), 5.65 (br s, 1H, NH), 4.11 (t, 2H, *J* = 6.6 Hz, 1′′-H_2_), 2.17 (s, 6H, 2′-CH_3_ and 6′-CH_3_), 1.87–1.73 (m, 3H, 2′′-H_2_ and 3′′-H), 0.99 (d, 6H, *J* = 6.4 Hz, 3′′-CH_3_ and 4′′-H_3_); ^13^C{^1^H} NMR (126 MHz, CDCl_3_): *δ* 171.4 (C), 160.6 (C, d, ^1^*J*_CF_ = 243.7 Hz), 159.5 (C), 152.1 (C), 139.1 (2× C, d, ^3^*J*_CF_ = 8.8 Hz), 133.9 (C, d, ^4^*J*_CF_ = 2.7 Hz), 132.8 (CH), 114.6 (2× CH, d, ^2^*J*_CF_ = 21.4 Hz), 106.2 (CH), 102.2 (C), 99.3 (CH), 67.8 (CH_2_), 38.1 (CH_2_), 25.3 (CH), 22.6 (2× CH_3_), 18.46 (CH_3_), 18.45 (CH_3_); MS (ESI) *m*/*z* 367 (M + Na^+^, 100%); HRMS (ESI) *m*/*z*: [M + Na]^+^ calcd for C_20_H_25_FN_2_NaO_2_ 367.1792; found 367.1774.

#### 2-(Allyloxy)-6-[(4′-fluoro-2′,6′-dimethylphenyl)amino]benzamide (7f)

The reaction was carried out according to general procedure C and gave 2-(allyloxy)-6-[(4′-fluoro-2′,6′-dimethylphenyl)amino]benzamide (7f) as a pale yellow solid (0.0445 g, 95%). Mp 176–178 °C; IR (neat) 3443, 3181, 2920, 1647, 1605, 1582, 1451, 1238, 1053, 810 cm^−1^; ^1^H NMR (500 MHz, CDCl_3_): *δ* 10.12 (br s, 1H, NH), 7.99 (br s, 1H, NH), 7.02 (t, 1H, *J* = 8.2 Hz, 4-H), 6.82 (d, 2H, *J* = 9.1 Hz, 3′-H and 5′-H), 6.20 (br d, 1H, *J* = 8.2 Hz, 3-H), 6.11 (ddt, 1H, *J* = 17.2, 10.5, 5.6 Hz, 2′′-H), 5.84 (dd, 1H, *J* = 8.2, 0.8 Hz, 5-H), 5.75 (br s, 1H, NH), 5.46 (dd, 1H, *J* = 17.2, 1.2 Hz, 3′′-*H*H), 5.36 (dd, 1H, *J* = 10.5, 1.2 Hz, 3′′-H*H*), 4.63 (br d, 2H, *J* = 5.6 Hz, 1′′-H_2_), 2.17 (s, 6H, 2′-CH_3_ and 6′-CH_3_); ^13^C{^1^H} NMR (126 MHz, CDCl_3_): *δ* 171.3 (C), 160.6 (C, d, ^1^*J*_CF_ = 244.1 Hz), 159.0 (C), 152.0 (C), 139.1 (2× C, d, ^3^*J*_CF_ = 8.4 Hz), 133.9 (C, d, ^4^*J*_CF_ = 2.7 Hz), 132.7 (CH), 132.4 (CH), 119.2 (CH_2_), 114.6 (2× CH, ^2^*J*_CF_ = 21.4 Hz), 106.5 (CH), 102.5 (C), 99.7 (CH), 70.1 (CH_2_), 18.46 (CH_3_), 18.44 (CH_3_); MS (ESI) *m*/*z* 337 (M + Na^+^, 100%); HRMS (ESI) *m*/*z*: [M + Na]^+^ calcd for C_18_H_19_FN_2_NaO_2_ 337.1323; found 337.1313.

#### 6-[(4′-Fluoro-2′,6′-dimethylphenyl)amino]-2-methoxy-3-methylbenzamide (7g)

The reaction was carried out according to general procedure C and gave 6-[(4′-fluoro-2′,6′-dimethylphenyl)amino]-2-methoxy-3-methylbenzamide (7g) as a pale yellow solid (0.016 g, 55%). Mp 136–138 °C; IR (neat) 3464, 3226, 2923, 1623, 1569, 1496, 1404, 1257, 1130, 1046, 865 cm^−1^; ^1^H NMR (500 MHz, CDCl_3_): *δ* 9.36 (br s, 1H, NH), 7.95 (br s, 1H, NH), 6.94 (d, 1H, *J* = 8.6 Hz, 4-H), 6.81 (d, 2H, *J* = 9.1 Hz, 3′-H and 5′-H), 5.91 (d, 1H, *J* = 8.6 Hz, 5-H), 5.74 (br s, 1H, NH), 3.78 (s, 3H, OCH_3_), 2.17 (s, 6H, 2′-CH_3_ and 6′-CH_3_), 2.17 (s, 3H, 3-CH_3_); ^13^C{^1^H} NMR (126 MHz, CDCl_3_): *δ* 170.8 (C), 160.5 (C, d, ^1^*J*_CF_ = 243.7 Hz), 158.2 (C), 149.2 (C), 139.0 (2× C, d, ^3^*J*_CF_ = 8.5 Hz), 135.1 (CH), 134.0 (C, d, ^4^*J*_CF_ = 2.7 Hz), 117.7 (C), 114.6 (2× CH, d, ^2^*J*_CF_ = 21.8 Hz), 109.0 (CH), 106.9 (C), 61.2 (CH_3_), 18.48 (CH_3_), 18.47 (CH_3_), 15.2 (CH_3_); MS (ESI) *m*/*z* 325 (M + Na^+^, 100%); HRMS (ESI) *m*/*z*: [M + Na]^+^ calcd for C_17_H_19_FN_2_NaO_2_ 325.1323; found 325.1315.

#### 6-[(4′-Fluoro-2′,6′-dimethylphenyl)amino]-2-isobutoxy-3-methylbenzamide (7h)

The reaction was carried out according to general procedure C and gave 6-[(4′-fluoro-2′,6′-dimethylphenyl)amino]-2-isobutoxy-3-methylbenzamide (7h) as a white solid (0.059 g, 82%). Mp 125–127 °C; IR (neat) 3449, 3233, 2963, 1651, 1574, 1497, 1258, 1042 cm^−1^; ^1^H NMR (400 MHz, CDCl_3_): *δ* 9.23 (br s, 1H, NH), 7.93 (br s, 1H, NH), 6.93 (d, 1H, *J* = 8.6 Hz, 4-H), 6.81 (d, 2H, *J* = 9.1 Hz, 3′-H and 5′-H), 5.92 (br s, 1H, NH), 5.90 (d, 1H, *J* = 8.6 Hz, 5-H), 3.62 (d, 2H, *J* = 6.7 Hz, 1′′-H_2_), 2.22–2.06 (m, 10H, 3-CH_3_, 2′-CH_3_, 6′-CH_3_ and 2′′-H), 1.06 (d, 6H, *J* = 6.7 Hz, 2′′-CH_3_ and 3′′-H_3_); ^13^C{^1^H} NMR (101 MHz, CDCl_3_): *δ* 171.0 (C), 160.5 (C, d, ^1^*J*_CF_ = 243.7 Hz), 157.1 (C), 148.9 (C), 139.0 (2× C, d, ^3^*J*_CF_ = 8.6 Hz), 135.0 (CH), 134.0 (C, d, ^4^*J*_CF_ = 2.8 Hz), 118.0 (C), 114.6 (2× CH, d, ^2^*J*_CF_ = 21.7 Hz), 108.8 (CH), 107.7 (C), 80.7 (CH_2_), 29.3 (CH), 19.3 (2× CH_3_), 18.48 (CH_3_), 18.47 (CH_3_), 15.4 (CH_3_); MS (ESI) *m*/*z* 367 (M + Na^+^, 100%); HRMS (ESI) *m*/*z*: [M + Na]^+^ calcd for C_20_H_25_FN_2_NaO_2_ 367.1792; found 367.1781.

#### 2-Bromo-6-(mesitylamino)benzoic acid (14)^16^

The reaction was carried out according to general procedure A and gave 2-bromo-6-(mesitylamino)benzoic acid (14) as a brown solid (1.44 g, 94%). The spectroscopic data were consistent with the literature.^16^ Mp 169–171 °C; ^1^H NMR (400 MHz, CDCl_3_): *δ* 7.00–6.90 (m, 4H, 3-H, 4-H, 3′-H and 5′-H), 6.18 (dd, 1H, *J* = 7.8, 1.8 Hz, 5-H), 2.31 (s, 3H, 4′-CH_3_), 2.15 (s, 6H, 2′-CH_3_ and 6′-CH_3_); ^13^C{^1^H} NMR (101 MHz, CDCl_3_): *δ* 172.5 (C), 150.3 (C), 136.5 (C), 136.2 (2× C), 134.0 (C), 133.5 (CH), 129.3 (2× CH), 123.4 (C), 122.8 (CH), 113.1 (C), 112.2 (CH), 21.0 (CH_3_), 18.1 (2× CH_3_); MS (EI) *m*/*z* 333 (M^+^, 100%), 315 (55), 300 (64), 208 (25), 135 (21).

#### 6-(Mesitylamino)-2-methoxybenzoic acid (15)

The reaction was carried out according to general procedure B and gave 6-(mesitylamino)-2-methoxybenzoic acid (15) as an orange solid (1.62 g, 95%). Mp 83–85 °C; IR (neat) 3277, 3176, 2918, 1676, 1573, 1435, 1397, 1356, 1254, 1190, 1061, 771 cm^−1^; ^1^H NMR (500 MHz, CDCl_3_): *δ* 11.50 (br s, 1H, OH), 9.93 (s, 1H, NH), 7.10 (t, 1H, *J* = 8.3 Hz, 4-H), 6.95 (s, 2H, 3′-H and 5′-H), 6.22 (br d, 1H, *J* = 8.3 Hz, 3-H), 5.96 (dd, 1H, *J* = 8.3, 0.9 Hz, 5-H), 4.04 (s, 3H, OCH_3_), 2.31 (s, 3H, 4′-CH_3_), 2.14 (s, 6H, 2′-CH_3_ and 6′-CH_3_); ^13^C{^1^H} NMR (126 MHz, CDCl_3_): *δ* 169.1 (C), 159.9 (C), 152.9 (C), 136.5 (2× C), 136.4 (C), 134.4 (C), 134.3 (CH), 129.2 (2× CH), 107.5 (CH), 98.6 (C), 97.7 (CH), 56.8 (CH_3_), 21.0 (CH_3_), 18.1 (2× CH_3_); MS (ESI) *m*/*z* 284 ([M–H]^−^, 100%); HRMS (ESI) *m*/*z*: [M–H]^−^ calcd for C_17_H_18_NO_3_ 284.1292; found 284.1292.

#### 6-(Mesitylamino)-2-methoxybenzamide (16)

The reaction was carried out according to general procedure C and gave 6-(mesitylamino)-2-methoxybenzamide (16) as a white solid (1.53 g, 95%). Mp 180–182 °C; IR (neat) 3439, 3171, 2917, 1646, 1590, 1458, 1406, 1365, 1254, 1084, 806 cm^−1^; ^1^H NMR (500 MHz, CDCl_3_): *δ* 10.18 (br s, 1H, NH), 7.90 (br s, 1H, NH), 7.01 (t, 1H, *J* = 8.3 Hz, 4-H), 6.92 (s, 2H, 3′-H and 5′-H), 6.17 (dd, 1H, *J* = 8.3, 0.9 Hz, 3-H), 6.09 (br s, 1H, NH), 5.88 (dd, 1H, *J* = 8.3, 0.9 Hz, 5-H), 3.89 (s, 3H, OCH_3_), 2.29 (s, 3H, 4′-CH_3_), 2.15 (s, 6H, 2′-CH_3_ and 6′-CH_3_); ^13^C{^1^H} NMR (126 MHz, CDCl_3_): *δ* 171.5 (C), 159.9 (C), 152.1 (C), 136.5 (2× C), 135.6 (C), 135.4 (C), 132.6 (CH), 129.0 (2× CH), 106.6 (CH), 102.0 (C), 98.0 (CH), 55.9 (CH_3_), 20.9 (CH_3_), 18.2 (2× CH_3_); MS (ESI) *m*/*z* 285 (M + H^+^, 100%); HRMS (ESI) *m*/*z*: [M + H]^+^ calcd for C_17_H_21_N_2_O_2_ 285.1598; found 285.1600.

#### 3-Bromo-6-(mesitylamino)-2-methoxybenzamide (17)

To a dry flask under argon was added iron(iii) chloride (0.0450 g, 0.275 mmol, 5 mol%) and 1-butyl-3-methylimidazolium bis(trifluoromethylsulfonyl)imide ([BMIM]NTf_2_) (2.30 mL, 11.0 mmol) and the mixture stirred for 0.5 h. 6-(Mesitylamino)-2-methoxybenzamide (16) (1.56 g, 5.49 mmol) in dichloromethane (14 mL) was added along with *N*-bromosuccinimide (0.784 g, 4.72 mmol, 0.8 equiv.). After 1 h of stirring at room temperature, *N*-bromosuccinimide (0.196 g, 1.10 mmol, 0.2 equiv.) was added and the reaction stirred for a further 1 h. The mixture was diluted with ethyl acetate (20 mL) and filtered through a short pad of silica. The organic layer was then washed with 1 M sodium thiosulfate (2 × 50 mL), brine (50 mL) and then dried (MgSO_4_), filtered and concentrated *in vacuo*. Purification by flash column chromatography, eluting with 20% ethyl acetate in petroleum ether (40–60) gave 3-bromo-6-(mesitylamino)-2-methoxybenzamide (17) as a white solid (1.54 g, 77%). Mp 184–186 °C; IR (neat) 3456, 3256, 2924, 1651, 1566, 1489, 1404, 1258, 1042, 818 cm^−1^; ^1^H NMR (500 MHz, CDCl_3_): *δ* 9.56 (br s, 1H, NH), 7.83 (br s, 1H, NH), 7.21 (d, 1H, *J* = 9.1 Hz, 4-H), 6.94 (s, 2H, 3′-H and 5′-H), 6.08 (br s, 1H, NH), 5.95 (d, 1H, *J* = 9.1, Hz, 5-H), 3.89 (s, 3H, OCH_3_), 2.30 (s, 3H, 4′-CH_3_), 2.14 (s, 6H, 2′-CH_3_ and 6′-CH_3_); ^13^C{^1^H} NMR (126 MHz, CDCl_3_): *δ* 170.0 (C), 156.7 (C), 150.7 (C), 136.42 (CH), 136.39 (2× C), 136.2 (C), 134.7 (C), 129.3 (2× CH), 111.0 (CH), 108.2 (C), 102.0 (C), 61.9 (CH_3_), 21.1 (CH_3_), 18.3 (2× CH_3_); MS (ESI) *m*/*z* 385 (M + Na^+^, 100%); HRMS (ESI) *m*/*z*: [M + Na]^+^ calcd for C_17_H_19_^79^BrN_2_NaO_2_ 385.0522; found 385.0512.

#### 3-(4′′-Fluorophenyl)-6-(mesitylamino)-2-methoxybenzamide (8a)

The reaction was carried out according to general procedure D and gave 3-(4′′-fluorophenyl)-6-(mesitylamino)-2-methoxybenzamide (8a) as a white solid (0.0738 g, 71%). Mp 209–211 °C; IR (neat) 3449, 3225, 2924, 1651, 1489, 1250, 1227, 1042, 818, 756 cm^−1^; ^1^H NMR (400 MHz, CDCl_3_): *δ* 9.73 (br s, 1H, NH), 8.07 (br s, 1H, NH), 7.46 (dd, 2H, *J* = 8.7, 5.5 Hz, 2′′-H and 6′′-H), 7.08 (t, 2H, *J* = 8.7 Hz, 3′′-H and 5′′-H), 7.06 (d, 1H, *J* = 8.8 Hz, 4-H), 6.95 (s, 2H, 3′-H and 5′-H), 6.08 (d, 1H, *J* = 8.8 Hz, 5-H), 5.83 (br s, 1H, NH), 3.45 (s, 3H, OCH_3_), 2.32 (s, 3H, 4′-CH_3_), 2.19 (s, 6H, 2′-CH_3_ and 6′-CH_3_); ^13^C{^1^H} NMR (101 MHz, CDCl_3_): *δ* 171.0 (C), 161.8 (C, d, ^1^*J*_CF_ = 245.6 Hz), 157.8 (C), 150.9 (C), 136.5 (2× C), 136.0 (C), 135.1 (C), 134.7 (CH), 134.5 (C, d, ^4^*J*_CF_ = 3.4 Hz), 130.8 (2× CH, d, ^3^*J*_CF_ = 7.8 Hz), 129.2 (2× CH), 121.4 (C), 115.2 (2× CH, d, ^2^*J*_CF_ = 21.2 Hz), 109.7 (CH), 106.7 (C), 61.4 (CH_3_), 21.1 (CH_3_), 18.4 (2× CH_3_); MS (ESI) *m*/*z* 401 (M + Na^+^, 100%); HRMS (ESI) *m*/*z*: [M + Na]^+^ calcd for C_23_H_23_FN_2_NaO_2_ 401.1636; found 401.1623.

#### 3-(2′′-Fluorophenyl)-6-(mesitylamino)-2-methoxybenzamide (8b)

The reaction was carried out according to general procedure D and gave 3-(2′′-fluorophenyl)-6-(mesitylamino)-2-methoxybenzamide (8b) as a white solid (0.0698 g, 67%). Mp 219–221 °C; IR (neat) 3464, 3264, 2916, 1620, 1481, 1396, 1242, 1034, 748 cm^−1^; ^1^H NMR (400 MHz, CDCl_3_): *δ* 9.77 (br s, 1H, NH), 8.02 (br s, 1H, NH), 7.37 (t, 1H, *J* = 7.5 Hz, ArH), 7.34–7.24 (m, 1H, ArH), 7.20–7.07 (m, 2H, ArH), 7.04 (d, 1H, *J* = 8.7 Hz, 4-H), 6.94 (s, 2H, 3′-H and 5′-H), 6.08 (d, 1H, *J* = 8.7 Hz, 5-H), 5.88 (br s, 1H, NH), 3.47 (s, 3H, OCH_3_), 2.30 (s, 3H, 4′-CH_3_), 2.20 (s, 6H, 2′-CH_3_ and 6′-CH_3_); ^13^C{^1^H} NMR (101 MHz, CDCl_3_): *δ* 170.9 (C), 160.0 (C, d, ^1^*J*_CF_ = 246.4 Hz), 158.3 (C), 151.2 (C), 136.4 (2× C), 135.9 (C), 135.1 (CH, d, ^4^*J*_CF_ = 1.8 Hz), 135.0 (C), 132.1 (CH, d, ^4^*J*_CF_ = 3.1 Hz), 129.1 (2× CH), 128.7 (CH, d, ^3^*J*_CF_ = 8.1 Hz), 126.0 (C, d, ^2^*J*_CF_ = 15.7 Hz), 123.9 (CH, d, ^3^*J*_CF_ = 3.6 Hz), 116.2 (C), 115.7 (CH, d, ^2^*J*_CF_ = 22.8 Hz), 109.2 (CH), 106.4 (C), 61.6 (CH_3_), 20.9 (CH_3_), 18.3 (2× CH_3_); MS (ESI) *m*/*z* 401 (M + Na^+^, 100%); HRMS (ESI) *m*/*z*: [M + Na]^+^ calcd for C_23_H_23_FN_2_NaO_2_ 401.1636; found 401.1621.

#### 3-(2′′-Fluoro-4′′-methoxyphenyl)-6-(mesitylamino)-2-methoxybenzamide (8c)

The reaction was carried out according to general procedure D and gave 3-(2′′-fluoro-4′′-methoxyphenyl)-6-(mesitylamino)-2-methoxybenzamide (8c) as a white solid (0.0573 g, 51%). Mp 212–214 °C; IR (neat) 3466, 2913, 1624, 1489, 1256, 1040, 951, 826 cm^−1^; ^1^H NMR (400 MHz, CDCl_3_): *δ* 9.75 (br s, 1H, NH), 8.04 (br s, 1H, NH), 7.27 (t, 1H, *J* = 8.0 Hz, 6′′-H), 7.02 (d, 1H, *J* = 8.6 Hz, 4-H), 6.94 (s, 2H, 3′-H and 5′-H), 6.73 (dd, 1H, *J* = 8.0, 4.0 Hz, 5′′-H), 6.69 (dd, 1H, *J* = 11.8, 4.0 Hz, 3′′-H), 6.07 (d, 1H, *J* = 8.6 Hz, 5-H), 5.79 (br s, 1H, NH), 3.83 (s, 3H, 4′′-OCH_3_), 3.47 (s, 3H, 2-OCH_3_), 2.30 (s, 3H, 4′-CH_3_), 2.19 (s, 6H, 2′-CH_3_ and 6′-CH_3_); ^13^C{^1^H} NMR (101 MHz, CDCl_3_): *δ* 170.9 (C), 160.5 (C, d, ^1^*J*_CF_ = 247.5 Hz), 160.0 (C, d, ^3^*J*_CF_ = 10.8 Hz), 158.3 (C), 151.0 (C), 136.4 (2× C), 135.8 (C), 135.3 (CH, d, ^4^*J*_CF_ = 1.6 Hz), 135.1 (C), 132.3 (CH, d, ^3^*J*_CF_ = 5.2 Hz), 129.1 (2× CH), 118.1 (C, d, ^2^*J*_CF_ = 16.2 Hz), 116.1 (C), 109.8 (CH, d, ^4^*J*_CF_ = 3.0 Hz), 109.2 (CH), 106.4 (C), 101.7 (CH, d, ^2^*J*_CF_ = 26.6 Hz), 61.5 (CH_3_), 55.6 (CH_3_), 20.9 (CH_3_), 18.3 (2× CH_3_); MS (ESI) *m*/*z* 431 (M + Na^+^, 100%); HRMS (ESI) *m*/*z*: [M + Na]^+^ calcd for C_24_H_25_FN_2_NaO_3_ 431.1741; found 431.1721.

#### 3-(2′′-Fluoro-5′′-methoxyphenyl)-6-(mesitylamino)-2-methoxybenzamide (8d)

The reaction was carried out according to general procedure D and gave 3-(2′′-fluoro-5′′-methoxyphenyl)-6-(mesitylamino)-2-methoxybenzamide (8d) as a white solid (0.0694 g, 62%). Mp 196–198 °C; IR (neat) 3456, 2934, 1647, 1564, 1489, 1404, 1248, 1206, 1038, 752 cm^−1^; ^1^H NMR (400 MHz, CDCl_3_): *δ* 9.78 (br s, 1H, NH), 8.03 (br s, 1H, NH), 7.07–7.00 (m, 2H, 4-H and 3′′-H), 6.94 (s, 2H, 3′-H and 5′-H), 6.90 (dd, 1H, *J* = 6.0, 3.5 Hz, 6′′-H), 6.81 (dt, 1H, *J* = 8.8, 3.5 Hz, 4′′-H), 6.08 (d, 1H, *J* = 8.8 Hz, 5-H), 5.67 (br s, 1H, NH), 3.80 (s, 3H, 5′′-OCH_3_), 3.51 (s, 3H, 2-OCH_3_), 2.31 (s, 3H, 4′-CH_3_), 2.19 (s, 6H, 2′-CH_3_ and 6′-CH_3_); ^13^C{^1^H} NMR (101 MHz, CDCl_3_): *δ* 170.8 (C), 158.3 (C), 155.4 (C, d, ^4^*J*_CF_ = 2.0 Hz), 154.5 (C, d, ^1^*J*_CF_ = 238.9 Hz), 151.3 (C), 136.4 (2× C), 135.9 (C), 135.1 (CH, d, ^4^*J*_CF_ = 1.8 Hz), 135.0 (C), 129.1 (2× CH), 126.6 (C, d, ^2^*J*_CF_ = 17.5 Hz), 116.7 (CH, d, ^3^*J*_CF_ = 3.1 Hz), 116.2 (C), 116.1 (CH, d, ^2^*J*_CF_ = 28.7 Hz), 113.7 (CH, d, ^3^*J*_CF_ = 8.0 Hz), 109.2 (CH), 106.4 (C), 61.7 (CH_3_), 55.8 (CH_3_), 21.0 (CH_3_), 18.3 (2× CH_3_); MS (ESI) *m*/*z* 431 (M + Na^+^, 100%); HRMS (ESI) *m*/*z*: [M + Na]^+^ calcd for C_24_H_25_FN_2_NaO_3_ 431.1741; found 431.1723.

#### 3-(2′′-Fluoropyridin-3′′-yl)-6-(mesitylamino)-2-methoxybenzamide (8e)

The reaction was carried out according to general procedure D and gave 3-(2′′-fluoropyridin-3′′-yl)-6-(mesitylamino)-2-methoxybenzamide (8e) as a white solid (0.0190 g, 18%). Mp 214–216 °C; IR (neat) 3441, 3163, 1667, 1566, 1427, 1258, 1034, 825, 756 cm^−1^; ^1^H NMR (400 MHz, CDCl_3_): *δ* 9.77 (br s, 1H, NH), 8.18 (ddd, 1H, *J* = 4.8, 1.9, 1.1 Hz, 4′′-H), 7.93 (br s, 1H, NH), 7.85 (ddd, 1H, *J* = 9.5, 7.3, 1.9 Hz, 6′′-H), 7.23 (ddd, 1H, *J* = 7.3, 4.8, 1.7 Hz, 5′′-H), 7.06 (dd, 1H, *J* = 8.8, 1.3 Hz, 4-H), 6.95 (s, 2H, 3′-H and 5′-H), 6.10 (d, 1H, *J* = 8.8 Hz, 5-H), 5.76 (br s, 1H, NH), 3.49 (s, 3H, OCH_3_), 2.31 (s, 3H, 4′-CH_3_), 2.19 (s, 6H, 2′-CH_3_ and 6′-CH_3_); ^13^C{^1^H} NMR (101 MHz, CDCl_3_): *δ* 170.4 (C), 160.9 (C, d, ^1^*J*_CF_ = 239.5 Hz), 158.3 (C), 151.6 (C), 146.1 (CH, d, ^3^*J*_CF_ = 14.5 Hz), 142.3 (CH, d, ^3^*J*_CF_ = 4.4 Hz), 136.4 (2× C), 136.1 (C), 134.7 (CH, ^4^*J*_CF_ = 2.1 Hz), 134.7 (C), 129.2 (2× CH), 121.3 (CH, d, ^4^*J*_CF_ = 4.3 Hz), 120.8 (C, d, ^2^*J*_CF_ = 30.4 Hz), 114.3 (C, d, ^3^*J*_CF_ = 4.5 Hz), 109.5 (CH), 106.5 (C), 61.9 (CH_3_), 21.0 (CH_3_), 18.3 (2× CH_3_); MS (ESI) *m*/*z* 402 (M + Na^+^, 100%); HRMS (ESI) *m*/*z*: [M + Na]^+^ calcd for C_22_H_22_FN_3_NaO_2_ 402.1588; found 402.1578.

#### 2-Bromo-6-[(2′,6′-dimethylphenyl)amino]-3-methylbenzoic acid (19)

The reaction was carried out according to general procedure A and gave 2-bromo-6-[(2′,6′-dimethylphenyl)amino]-3-methylbenzoic acid (19) as a brown solid (2.74 g, 95%). Mp 126–128 °C; IR (neat) 3389, 2918, 1690, 1661, 1489, 1234, 773 cm^−1^; ^1^H NMR (500 MHz, CDCl_3_): *δ* 10.29 (br s, 1H, OH), 7.17–7.07 (m, 3H, 3′-H, 4′-H and 5′-H), 7.02 (d, 1H, *J* = 8.5 Hz, 4-H), 6.14 (d, 1H, *J* = 8.5 Hz, 5-H), 2.34 (s, 3H, 3-CH_3_), 2.20 (s, 6H, 2′-CH_3_ and 6′-CH_3_); ^13^C{^1^H} NMR (126 MHz, CDCl_3_): *δ* 173.4 (C), 146.0 (C), 137.1 (C), 136.0 (2× C), 134.0 (CH), 128.6 (2× CH), 128.0 (C), 126.4 (CH), 124.0 (C), 116.9 (C), 112.5 (CH), 23.0 (CH_3_), 18.2 (2× CH_3_); MS (ESI) *m*/*z* 356 (M + Na^+^, 100%); HRMS (ESI) *m*/*z*: [M + Na]^+^ calcd for C_16_H_16_^79^BrNNaO_2_ 356.0257; found 356.0250.

#### 6-[(2′,6′-Dimethylphenyl)amino]-2-methoxy-3-methylbenzoic acid (20)

The reaction was carried out according to general procedure B and gave 6-[(2′,6′-dimethylphenyl)amino]-2-methoxy-3-methylbenzoic acid (20) as a brown solid (1.96 g, 92%). Mp 92–94 °C; IR (neat) 3289, 2945, 1699, 1570, 1501, 1385, 1223, 1040, 818, 770 cm^−1^; ^1^H NMR (500 MHz, CDCl_3_): *δ* 12.15 (br s, 1H, OH), 9.59 (br s, 1H, NH), 7.18–7.06 (m, 3H, 3′-H, 4′-H and 5′-H), 7.01 (d, 1H, *J* = 8.7 Hz, 4-H), 6.03 (d, 1H, *J* = 8.7 Hz, 5-H), 3.93 (s, 3H, OCH_3_), 2.20 (s, 3H, 3-CH_3_), 2.18 (s, 6H, 2′-CH_3_ and 6′-CH_3_); ^13^C{^1^H} NMR (126 MHz, CDCl_3_): *δ* 168.7 (C), 158.2 (C), 150.2 (C), 137.11 (CH), 137.09 (C), 136.7 (2× C), 128.5 (2× CH), 126.7 (CH), 116.3 (C), 110.0 (CH), 102.1 (C), 62.2 (CH_3_), 18.2 (2× CH_3_), 15.1 (CH_3_); MS (ESI) *m*/*z* 308 (M + Na^+^, 100%); HRMS (ESI) *m*/*z*: [M + Na]^+^ calcd for C_17_H_19_NNaO_3_ 308.1257; found 308.1248.

#### 6-[(2′,6′-Dimethylphenyl)amino]-2-methoxy-3-methylbenzamide (21)

The reaction was carried out according to general procedure C and gave 6-[(2′,6′-dimethylphenyl)amino]-2-methoxy-3-methylbenzamide (21) as a white solid (1.30 g, 93%). Mp 116–118 °C; IR (neat) 3443, 3184, 2936, 1645, 1570, 1497, 1366, 1261, 1047, 816, 731 cm^−1^; ^1^H NMR (400 MHz, CDCl_3_): *δ* 9.47 (br s, 1H, NH), 7.93 (br s, 1H, NH), 7.19–7.03 (m, 3H, 3′-H, 4′-H and 5′-H), 6.94 (d, 1H, *J* = 8.6 Hz, 4-H), 6.04 (br s, 1H, NH), 5.96 (d, 1H, *J* = 8.6 Hz, 5-H), 3.79 (s, 3H, OCH_3_), 2.21 (s, 6H, 2′-CH_3_ and 6′-CH_3_), 2.18 (s, 3H, 3-CH_3_); ^13^C{^1^H} NMR (101 MHz, CDCl_3_): *δ* 170.9 (C), 158.1 (C), 148.9 (C), 138.1 (C), 136.6 (2× C), 134.9 (CH), 128.3 (2× CH), 126.0 (CH), 117.5 (C), 109.3 (CH), 107.0 (C), 61.1 (CH_3_), 18.3 (2× CH_3_), 15.2 (CH_3_); MS (ESI) *m*/*z* 307 (M + Na^+^, 100%); HRMS (ESI) *m*/*z*: [M + Na]^+^ calcd for C_17_H_20_N_2_NaO_2_ 307.1417; found 307.1409.

#### 6-[(4′-Iodo-2′,6′-dimethylphenyl)amino]-2-methoxy-3-methylbenzamide (22)

To a solution of 6-[(2′,6′-dimethylphenyl)amino]-2-methoxy-3-methylbenzamide (21) (0.500 g, 1.76 mmol) and silver bis(trifluoromethanesulfonyl)imide (0.0512 g, 0.132 mmol, 7.5 mol%) in tetrahydrofuran (7 mL) under argon was slowly added *N*-iodosuccinimide (0.554 g, 2.46 mmol) in tetrahydrofuran (2.0 mL). The reaction mixture was stirred at room temperature for 3 h in the dark. The reaction mixture was filtered through a short pad of Celite®, washed with ethyl acetate and concentrated *in vacuo*. Purification by flash column chromatography (20–25% diethyl ether in hexane) gave 6-[(4′-iodo-2′,6′-dimethylphenyl)amino]-2-methoxy-3-methylbenzamide (22) as a white solid (0.389 g, 54%). Mp 141–143 °C; IR (neat) 3447, 3202, 2934, 1645, 1572, 1497, 1468, 1398, 1366, 1265, 1215, 1047, 851, 733 cm^−1^; ^1^H NMR (500 MHz, CDCl_3_): *δ* 9.41 (br s, 1H, NH), 7.93 (br s, 1H, NH), 7.45 (s, 2H, 3′-H and 5′-H), 6.95 (d, 1H, *J* = 8.6 Hz, 4-H), 5.93 (d, 1H, *J* = 8.6 Hz, 5-H), 5.69 (br s, 1H, NH), 3.78 (s, 3H, OCH_3_), 2.17 (s, 3H, 3-CH_3_), 2.13 (s, 6H, 2′-CH_3_ and 6′-CH_3_); ^13^C{^1^H} NMR (126 MHz, CDCl_3_): *δ* 170.8 (C), 158.3 (C), 148.5 (C), 139.2 (2× C), 138.3 (C), 137.3 (2× CH), 135.1 (CH), 118.3 (C), 109.4 (CH), 107.4 (C), 90.9 (C), 61.3 (CH_3_), 18.1 (2× CH_3_), 15.4 (CH_3_); MS (ESI) *m*/*z* 433 (M + Na^+^, 100%); HRMS (ESI) *m*/*z*: [M + Na]^+^ calcd for C_17_H_19_IN_2_NaO_2_ 433.0383; found 433.0385.

#### 6-[(4′-(Trimethylstannyl)-2′-6′-dimethylphenyl)amino]-2-methoxy-3-methylbenzamide (23)

An oven-dried microwave vial was flushed with argon and charged with 6-[(4′-iodo-2′,6′-dimethylphenyl)amino]-2-methoxy-3-methylbenzamide (22) (0.0800 g, 0.195 mmol) in toluene (3.0 mL). Lithium chloride (0.0413 g, 0.975 mmol) was added and the mixture was degassed under argon, while stirring for 0.2 h. Tetrakis(triphenylphosphine)palladium(0) (0.0451 g, 0.0390 mmol, 20 mol%) and hexamethylditin (0.0806 mL, 0.390 mmol) were added under argon and the reaction mixture was stirred under reflux for 21 h. After cooling to room temperature, the reaction was quenched by the addition of 30% aqueous solution of potassium fluoride (2.0 mL) and stirred at room temperature for 0.5 h. The mixture was filtered through a short pad of Celite®, washed with ethyl acetate and concentrated *in vacuo*. Purification by neutral alumina (Brockmann grade V) flash column chromatography, eluting with 20% diethyl ether in hexane gave 6-[(4′-(trimethylstannyl)-2′-6′-dimethylphenyl)amino]-2-methoxy-3-methylbenzamide (23) as a colourless oil (0.0734 g, 84%). IR (neat) 3445, 3188, 2918, 1651, 1576, 1497, 1267, 1258, 1049, 765 cm^−1^; ^1^H NMR (500 MHz, CDCl_3_): *δ* 9.46 (br s, 1H, NH), 7.92 (br s, 1H, NH), 7.22 (s, 2H, 3′-H and 5′-H), 6.93 (d, 1H, *J* = 8.6 Hz, 4-H), 5.98 (d, 1H, *J* = 8.6 Hz, 5-H), 5.69 (br s, 1H, NH), 3.78 (s, 3H, OCH_3_), 2.19 (s, 6H, 2′-CH_3_ and 6′-CH_3_), 2.17 (s, 3H, 3-CH_3_), 0.29 (s, 9H, Sn(CH_3_)_3_); ^13^C{^1^H} NMR (126 MHz, CDCl_3_): *δ* 170.9 (C), 158.3 (C), 149.1 (C), 139.5 (C), 138.5 (C), 136.1 (2× C), 136.0 (2× CH), 135.1 (CH), 117.7 (C), 109.6 (CH), 107.1 (C), 61.3 (CH_3_), 18.4 (2× CH_3_), 15.3 (CH_3_), −9.4 (3× CH_3_); MS (ESI) *m*/*z* 471 (M + Na^+^, 100%); HRMS (ESI) *m*/*z*: [M + Na]^+^ calcd for C_20_H_28_N_2_NaO_2_Sn 471.1065; found 471.1056.

#### Procedure for the [^35^S]GTPγS binding assay^25^

The assay buffer was prepared from HEPES (20 mM), NaCl (100 mM), MgCl_2_ (10 mM) and fatty acid-free BSA (0.1%) at pH 7.4. ChemiScreen™ S1P_1–3,5_ lysophospholipid receptor membrane preparations were obtained from Eurofins. The membrane preparation was diluted to 0.4 g mL^−1^ with assay buffer and mixed 1 : 1 (v/v) with a 0.4 mg mL^−1^ solution of saponin in assay buffer. Nine concentrations of the compounds to be tested were prepared (10 μM, 1 μM, 100 nM, 10 nM, 1 nM, 100 pM, 10 pM, 1 pM, 100 fM). Merck™ Multiscreen® HTS 96 well filter plates were pre-wet with 70% aqueous EtOH (50 μL per well) for 30 seconds and then washed twice with assay buffer (200 μL per well). To each well was added membrane preparation (25 μL), GDP in assay buffer (25 μL, 2 μM), [^35^S]GTPγS in assay buffer (25 μL, 1.2 nM) and test compound (25 μL). For total binding, S1P solution in assay buffer (25 μL, 10 μM) was added and for basal, assay buffer (25 μL) was added. The assay plate was incubated at 30 °C for 0.5 h before the addition of cold assay buffer (100 μL per well) to stop the reaction. The plate was filtered using a Millipore vacuum manifold, with a pressure between 600–800 mbar and then dried at 50 °C for 1–2 h. Betaplate Scint scintillation cocktail (30 μL per well) was added and the disintegrations per minute were determined using a Wallac 1450 Microbeta Trilux liquid scintillation and luminescence counter. EC_50_ values were derived from non-linear regression analysis using GraphPad Prism Version 6 (GraphPad Software Inc).

#### Docking studies

All computational experiments were conducted on a Dell Latitude 5420 11th Gen Intel(R) Core(TM) i7-1185G7. The cryo-EM structure of siponimod-bound S1P_5_ in complex with Gi protein was obtained from the Protein Database (PDB code: 7EW1, http://www.rcsb.org).^29^ Siponimod (4) and TEFM78 (7g) were drawn using MarvinSketch 24.3.0. The three-dimensional, energetically minimised conformer of each compound was then obtained using Mercury software from the Cambridge Crystallographic Data Centre (CCDC). Using Hermes GOLD software from the CCDC, a docking was configured with the siponimod-S1P_5_ complex (PDB code: 7EW1) in which the bound siponimod ligand was extracted and hydrogen atoms were added to the protein. No water molecules were present within the cryo-EM structure of the protein and no further changes were made to the protein. The binding site was defined using the previously extracted siponimod ligand with a radius of 10.0 Å. The energetically minimised conformer of Siponimod (4) and TEFM78 (7g) were specified as the ligands for docking and the reference ligand was specified as the previously extracted siponimod ligand. The fitness function used to score the docking solutions was specified as ASP. The ASP scoring function was selected following a screen of all scoring functions available within the GOLD software in which the energetically minimised conformer of Siponimod (4) was redocked into S1P_5_. The ASP scoring function generated the highest percentage of solutions with a root-mean-square deviation (RMSD) of less than 2.0 Å than any other scoring function screened. Additional amendments to default settings were made including the number of genetic algorithm runs was set to 20 for each ligand, early termination was not allowed and search efficient was specified as >200%. The docking solutions were then analysed within Hermes and UCSF ChimeraX 1.8 software.

#### Radiochemistry: general experimental

No-carrier-added aqueous [^18^F]fluoride was produced *via* the ^18^O(p,n)^18^F nuclear reaction by irradiation of ^18^O-enriched water by a GE PETtrace 8 cyclotron. All radiofluorination reactions were carried out on a GE TRACERlab FX_FN_ automated synthesiser. Sep-Pak QMA Carbonate Plus Light cartridges (Waters) were preconditioned with potassium trifluoromethanesulfonate (90 mg mL^−1^; 10 mL) and water (10 mL) prior to use. Oasis HLB Plus Light (Waters) cartridges were preconditioned with ethanol (5 mL) and then with water (10 mL) prior to use. The starting activity for calculating the radiochemical yield was determined from the GM reading taken immediately following delivery of [^18^F]fluoride to the synthesiser from the cyclotron. The final activity readings were recorded using a Capintec CRC-25 PET dose calibrator.

#### Analytical HPLC method

Analytical HPLC was carried out on a Thermo Dionex Ulimate system 3000 equipped with a Berthold FlowStar LB 513 radio flow detector and a DAD-3000 UV detector. An isocratic mobile phase of 70% acetonitrile in water was used with a Phenomenex Gemini 5 μm C18 110 Å 250 × 4.6 mm column at a rate of 1 mL min^−1^. The nonradioactive standards were detected using a UV wavelength of 254 nm.

#### Preparation of [^18^F]TEFM78

Immediately prior to delivering [^18^F]fluoride, 6-[(4′-(trimethylstannyl)-2′-6′-dimethylphenyl)amino]-2-methoxy-3-methylbenzamide (23) (5.0 mg), copper(ii) trifluoromethanesulfonate (8.0 mg) and pyridine (20 μL) were dissolved in *N*,*N*-dimethylacetamide (0.70 mL) and the solution added to a vial on the synthesiser. Cyclotron target water containing [^18^F]fluoride was transferred to and trapped on a Sep-Pak QMA Carbonate Plus Light 46 mg cartridge. The activity was eluted into a reaction vessel using a solution of potassium trifluoromethanesulfonate (4.5 mg) and potassium carbonate (50 μg) in water (0.30 mL) and acetonitrile (0.30 mL). This solution was dried by being stirred at 100 °C under vacuum and a stream of helium gas for 2 min. This process was repeated twice using acetonitrile (2 × 1 mL). The [^18^F]fluoride was then completely dried by applying full vacuum for 1 min. The solution of 6-[(4′-(trimethylstannyl)-2′-6′-dimethylphenyl)amino]-2-methoxy-3-methylbenzamide (23) was added to the reaction vessel, which was sealed, and the mixture heated to 110 °C for 20 min while being stirred. The reaction mixture was cooled to 30 °C and diluted with a 50% aqueous solution of acetonitrile (2.0 mL). The reaction mixture was then transferred into the HPLC injector loop for purification. Purification was performed by semipreparative HPLC with a SYKMN S1122 solvent delivery system using a Phenomenex Luna 5 μm PFP(2) 100 Å 250 × 10 mm column and eluted using a 60% solution of acetonitrile in water at a flow rate of 4.0 mL min^−1^. The product fraction was identified using a gamma detector at a retention time of approximately 13 min and collected into a flask containing water (20 mL). The diluted fraction was then passed onto an Oasis HLB Plus Light cartridge, washed with water (5.0 mL), and eluted from the cartridge with ethanol (0.50 mL) and saline (4.5 mL). [^18^F]TEFM78 was isolated in 16 ± 4% radiochemical yield with a radiochemical purity of >99% and a molar activity of 229 ± 22 GBq μmol^−1^, starting from 75.3 ± 2.2 GBq of [^18^F]fluoride (*n* = 2). The total synthesis time from delivery of [^18^F]fluoride to extraction of the product was 62 min.

#### [^18^F]TEFM78 *in vivo* studies in rats

All animal procedures were performed in accordance with the guidelines for care and use of laboratory animals of Edinburgh University and experiments were approved by the Home Office under the Animals (Scientific Procedures) Act 1986, UK. Naïve adult male Sprague–Dawley rats (age: 12.92 ± 0.34 weeks, weight: 402.67 ± 19.48 g, mean ± SD, *n* = 3) were used for *in vivo* scanning. Anesthesia was induced with the use of an induction chamber with 3% isoflurane (Isoflo, APIECE) delivered in a mixture of 0.5 L min^−1^ of oxygen and 0.5 L min^−1^ nitrous oxide. Once anesthesia was induced, the rat was transferred to the bed of the PET scanner (NanoPET/CT, Mediso Medical Imaging Systems, Budapest, Hungary) ensuring the nose was in the nose cone and anesthesia was maintained with 2–2.5% isoflurane (50/50 oxygen/nitrous oxide, 1 L min^−1^). A rectal thermometer was inserted and a respiration monitor was placed on the chest of the rat. The tail vein was then cannulated with a 26G cannula to allow for the bolus injection of the radiotracer. PET scans were conducted on a Mediso nanoPET/CT (Mediso Medical Imaging Systems, Hungary) using a coincidence mode of 1 : 5. The acquisition time was set to 120 minutes emission scan. Once the PET scan was complete, a CT scan was acquired using the following parameters: a semi-circular trajectory with maximum field of view and 360 projections; a tube voltage of 50 kVp and exposure time of 300 ms were used, with a binning setting of 1 : 4. PET data was reconstructed using Mediso's iterative Teratomo 3D reconstruction algorithm with 4 iterations and 6 subsets, full detector model, spike filter on, normal regularization, a 400 to 600 keV energy window and a 0.4 mm voxel size. All PET data was corrected for randoms, scatter and attenuation. Emission data was re-binned as follows 18 × 10 s, 2 × 30 s, 1 × 60 s, 2 × 2 min, 10 × 5 min and 6 × 10 min. Once the PET scans were reconstructed, data was analysed using PMOD image analysis software (PMOD Technologies LLC, Zürich, Switzerland). Volumes of interest (VOIs) were created by using all regions in the rat Schiffer and SIGMA brain atlas (whole brain), whole grey matter and whole white matter regions or manually drawn in the case of the spinal cord region. Time activity curves (TACs) were created for each region and PET data expressed as standard uptake value (SUV), calculated as radioactive concentration in the VOI divided by the injected dose and animal's weight.

## Data availability

The data supporting this article have been included as part of the ESI.† This includes the physicochemical properties and binding curves of 7a–7h and 8a–8e, selected analytical HPLC traces for target compounds, HPLC traces from the radiosynthesis of [^18^F]TEFM78, ^1^H and ^13^C NMR spectra of all compounds.

## Conflicts of interest

There are no conflicts to declare.

## Supplementary Material

MD-016-D4MD00929K-s001
