# Supplementary material for: Synthesis and evaluation of 6-arylaminobenzamides as positron emission tomography imaging ligands for the sphingosine-1-phosphate-5 receptor
Source: RSC Med Chem. 2025 Jan 3;16(3):1235–49. doi: 10.1039/d4md00929k (PMC11729640; doi:10.1039/d4md00929k)

Supplementary Information (SI) for RSC Medicinal Chemistry.
This journal is © The Royal Society of Chemistry 2025

Supporting Information for:

Synthesis and Evaluation of 6-Arylaminobenzamides as Positron Emission Tomography Imaging Ligands for the Sphingosine-1-Phosphate-5 Receptor

Timaeus E. F. Morgan,[a,b] Emma K. Grant,[a] Robert C. Shaw,[b,c] Lachlan J. N. Waddell,[a] Martyn C. Henry,[a] Holly McErlain,[a] Carlos J. Alcaide-Corral,[b] Sally L. Pimlott,[d] Adriana A. S. Tavares[b,c] and Andrew Sutherland[a]*

[a]School of Chemistry, University of Glasgow, Glasgow, G12 8QQ, U.K. [b]Edinburgh Imaging, University of Edinburgh, 47 Little France Crescent, Edinburgh, EH16 4TJ, U.K. [c]University/BHF Centre for Cardiovascular Sciences, University of Edinburgh, 47 Little France Crescent, Edinburgh, EH16 4TJ, U.K. [d]West of Scotland PET Centre, Greater Glasgow and Clyde NHS Trust, Glasgow, G12 OYN, U.K.

Table of Contents

1. Physiochemical Properties of 6-arylaminobenzamides **7a**–**7h** and **8a**–**8e** S2
2. Binding curves for S1P (**1**), 6-arylaminobenzamides **7a**–**7h** and **8a**–**8e** S3–S10
3. Selected Analytical HPLC Traces for: **7a**, **7d**, **7e**, **7f**, **7g** (TEFM78) and **8a**–**8e** S11–S20
4. HPLC Traces for [^{18}F]TEFM78 S21
5. Other Docked Poses of TEFM78 Generated by GOLD S22
6. References S23
7. ^{1}H and ^{13}C NMR Spectra for all Compounds S24–S87

1. Physiochemical Properties of 6-arylaminobenzamides 7a–7h and 8a–8e.[1]

Table S1

Compound	log P^a	$P_m{}^b$	$K_m{}^b$	%PPB[c]
**7a**	4.07	0.319	101	99%
**7b**	3.41	0.223	64.4	99%
**7c**	4.63	0.487	101	100%
**7d**	4.65	0.509	168	100%
**7e**	4.98	0.646	223	100%
**7f**	4.10	0.285	89.6	100%
**7g**	3.90	0.233	70.5	99%
**7h**	5.12	0.518	178	100%
**8a**	5.57	0.650	246	100%
**8b**	5.57	0.514	195	100%
**8c**	5.45	0.465	190	100%
**8d**	5.45	0.446	182	100%
**8e**	4.70	0.211	79.9	99%

[a]Calculated using ChemDraw. [b]Determined using immobilised artificial membrane (IAM) column. [c]Determined using human serum albumin (HSA) coated column.

2. Binding curves of S1P (1), 6-arylaminobenzamides 7a–7h and 8a–8e.[2]

Sphingosine-1-phosphate:

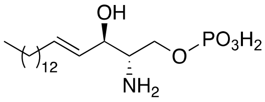

1

S1P3 **S1P5**

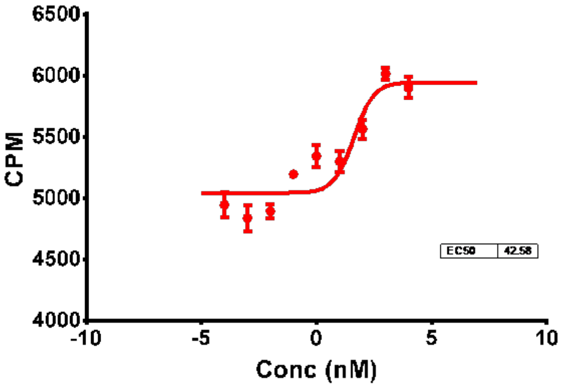

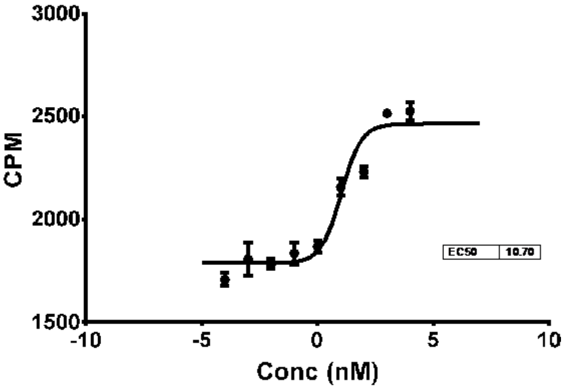

6-[(4′-Fluoro-2′,6′-dimethylphenyl)amino]-2-isopropoxybenzamide:

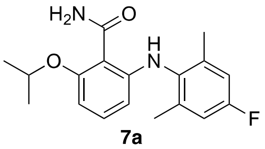

7a

S1P5

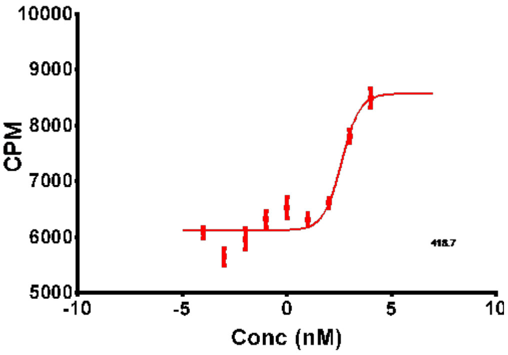

6-[(4′-Fluoro-2′,6′-dimethylphenyl)amino]-2-methoxybenzamide:

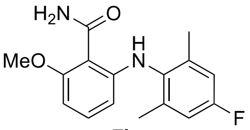

7b

S1P1

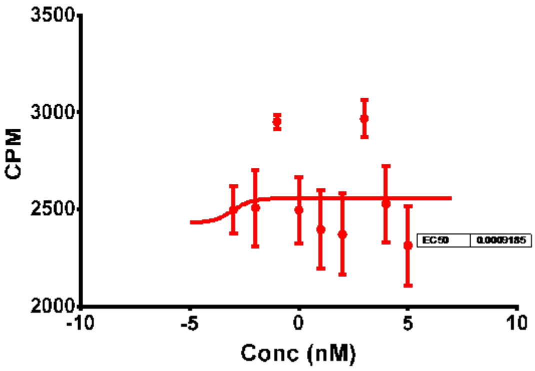

S1P2

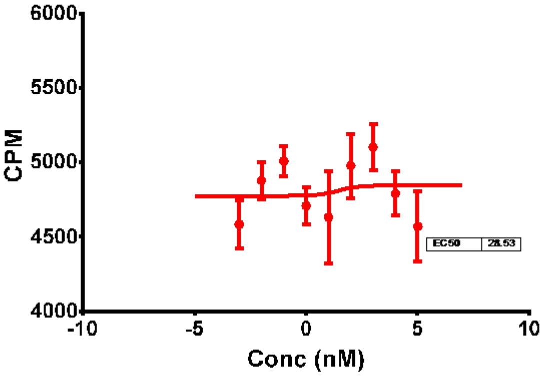

S1P3

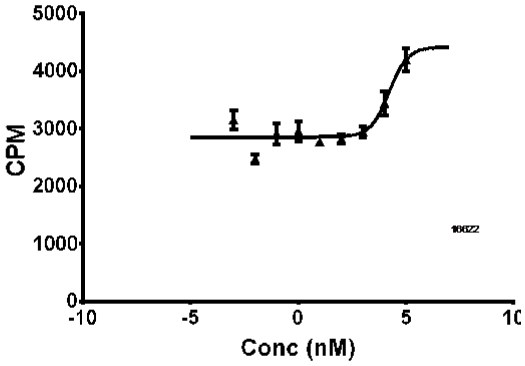

S1P5

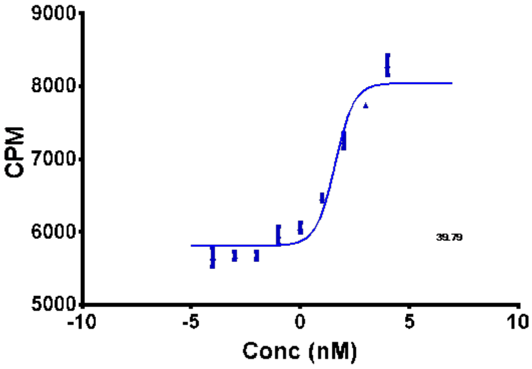

6-[(4′-Fluoro-2′,6′-dimethylphenyl)amino]-2-isobutoxybenzamide:

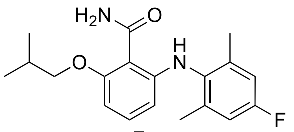

7c

S1P5

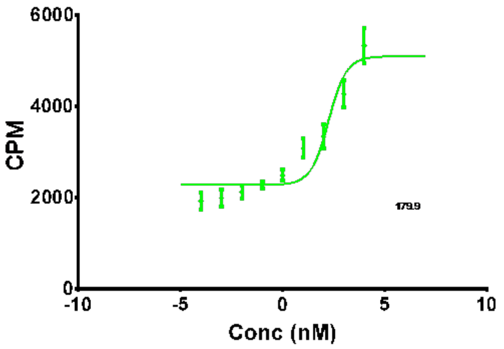

2-*n*-Butoxy-6-[(4′-fluoro-2′,6′-dimethylphenyl)amino]benzamide:

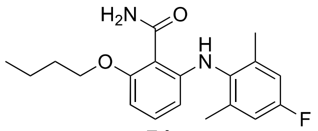

7d

S1P5

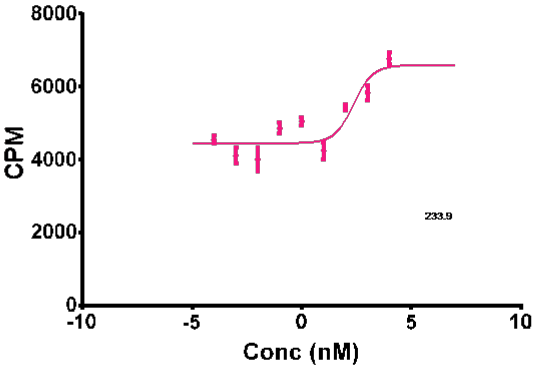

6-[(4′-Fluoro-2′,6′-dimethylphenyl)amino]-2-(3″-methyl-*n*-butoxy)benzamide:

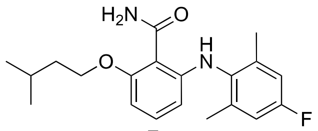

7e

S1P5

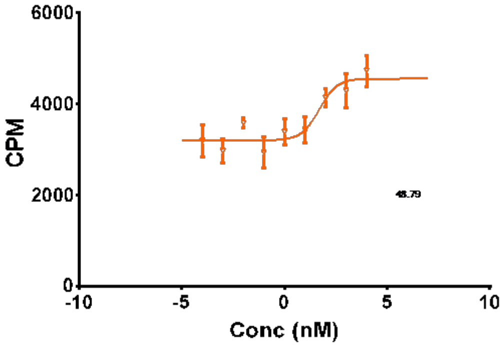

2-(Allyloxy)-6-[(4′-fluoro-2′,6′-dimethylphenyl)amino]benzamide:

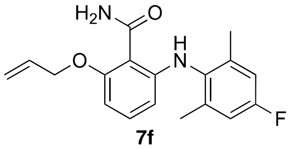

7f

S1P1

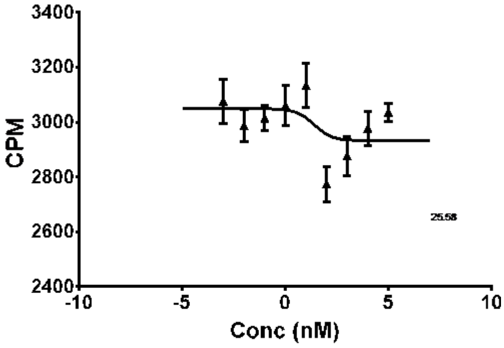

S1P2

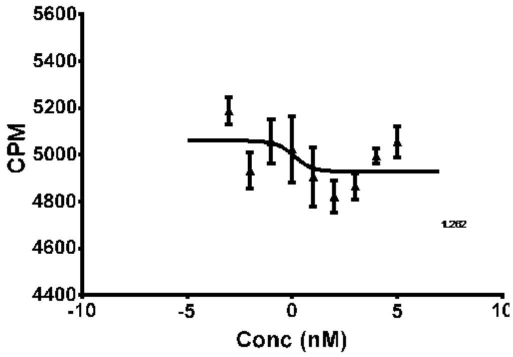

S1P3

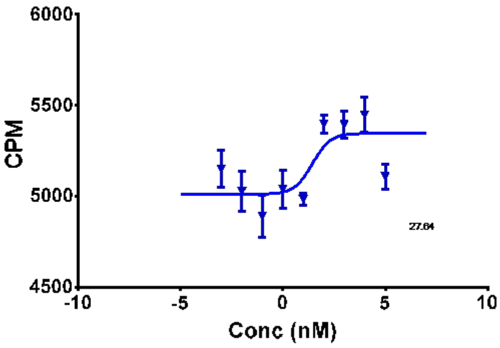

S1P5

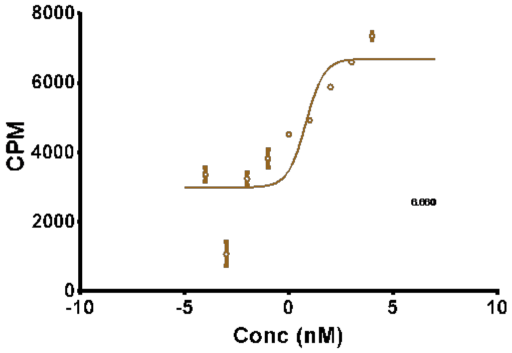

6-[(4′-Fluoro-2′,6′-dimethylphenyl)amino]-2-methoxy-3-methylbenzamide:

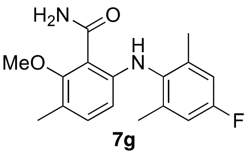

7g

S1P1

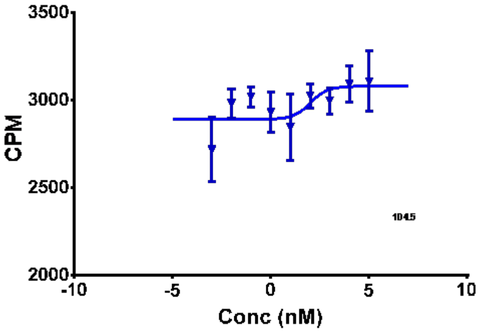

S1P2

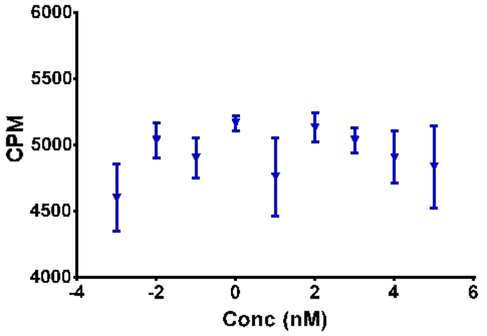

S1P3

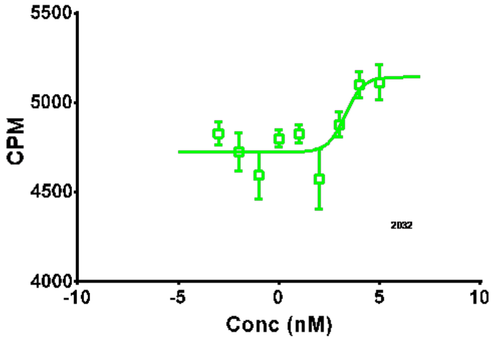

S1P5

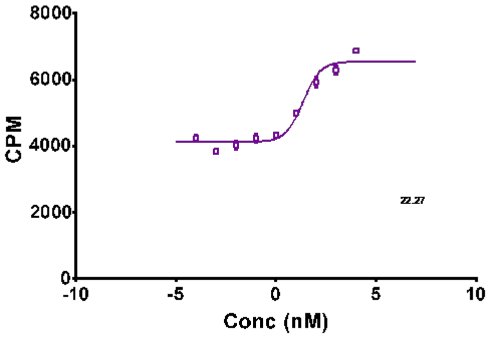

6-[(4′-Fluoro-2′,6′-dimethylphenyl)amino]-2-isobutoxy-3-methylbenzamide:

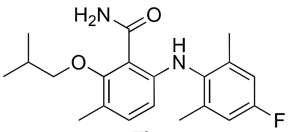

7h

S1P5

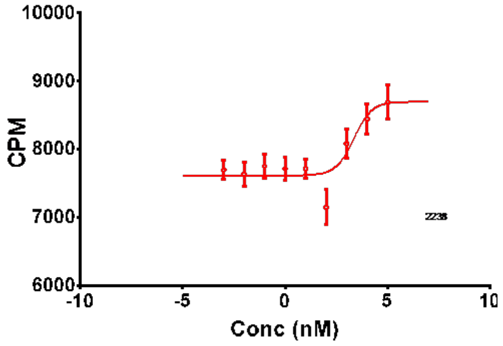

3-(4″-Fluorophenyl)-6-(mesitylamino)-2-methoxybenzamide:

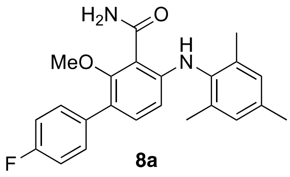

8a

S1P5

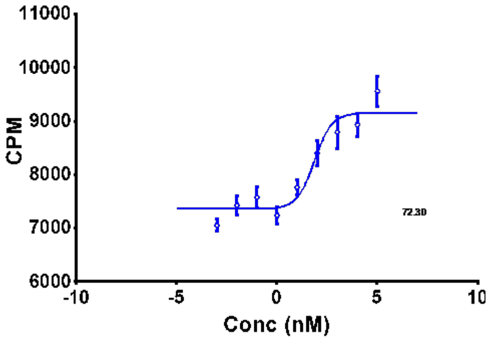

3-(2″-Fluorophenyl)-6-(mesitylamino)-2-methoxybenzamide:

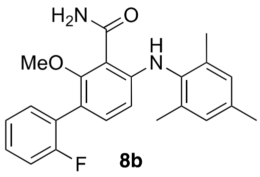

8b

S1P5

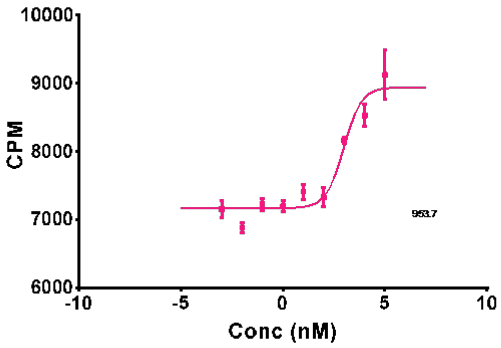

3-(2″-Fluoro-4″-methoxyphenyl)-6-(mesitylamino)-2-methoxybenzamide:

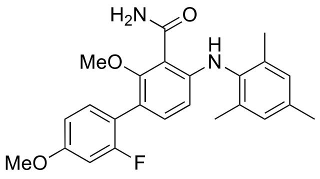

8c

$S1P_1$

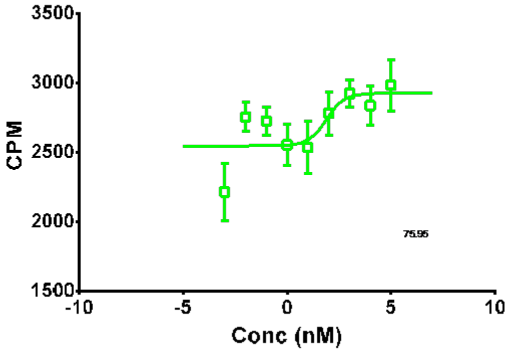

$S1P_2$

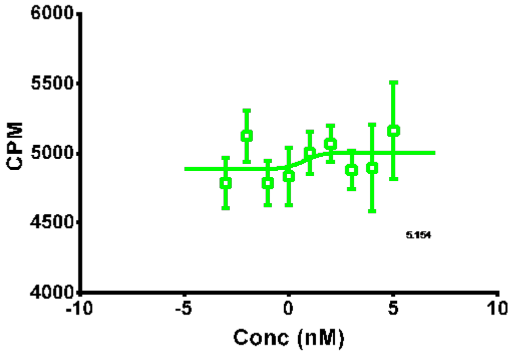

$S1P_3$

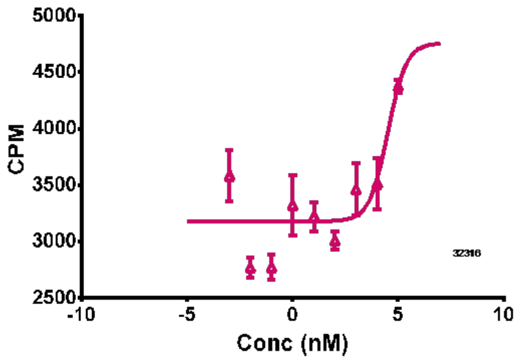

$S1P_5$

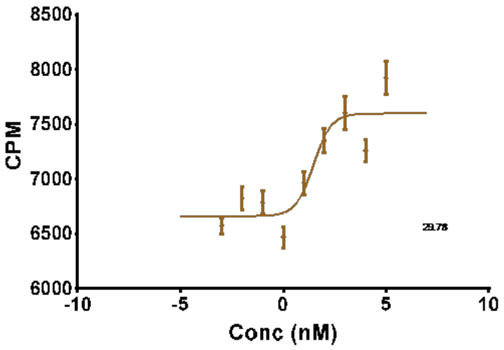

3-(2″-Fluoro-5″-methoxyphenyl)-6-(mesitylamino)-2-methoxybenzamide:

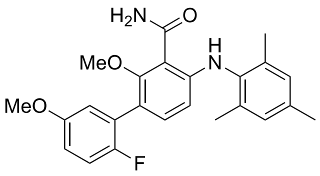

8d

$S1P_5$

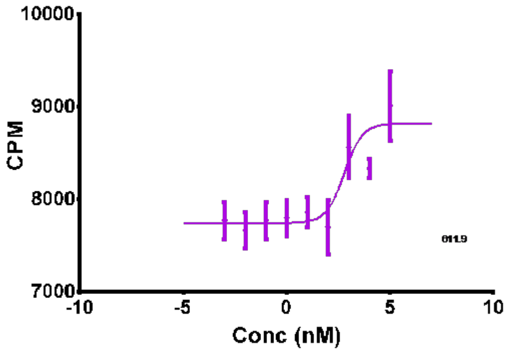

3-(2″-Fluoropyridin-3″-yl)-6-(mesitylamino)-2-methoxybenzamide:

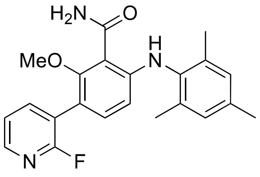

8e

S1P1

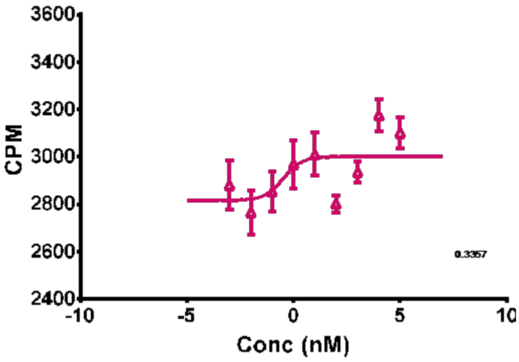

S1P2

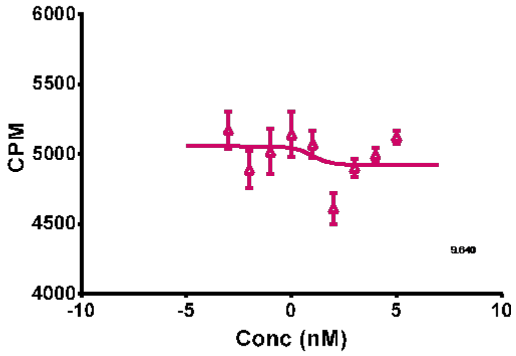

S1P3

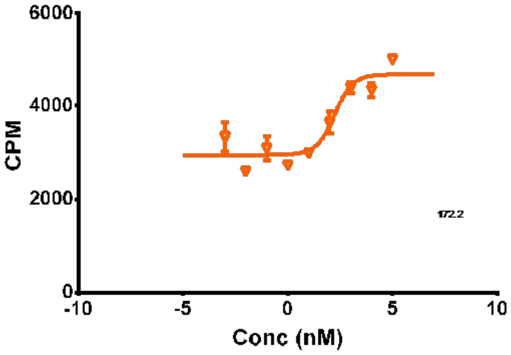

S1P5

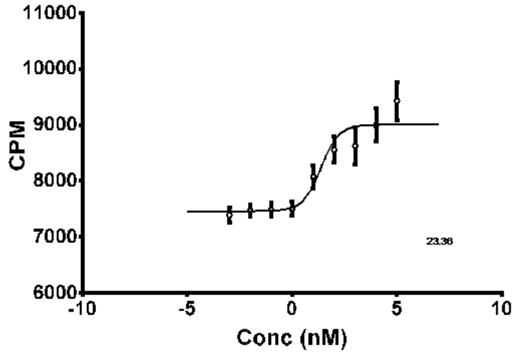

3. Selected Analytical HPLC Traces for: 7a, 7d, 7e, 7f, 7g (TEFM78) and 8a–8e

6-[(4′-Fluoro-2′,6′-dimethylphenyl)amino]-2-isopropoxybenzamide (7a)

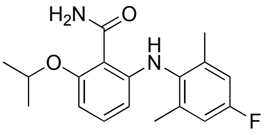

Sample Name	: TEFM01-53_run3		
Sample ID	: TEFM01-53_run3		
Data Filename	: TEFM01-53_run3.lcd		
Method Filename	: EVO_10-90_MeCN_H2O_1mlmin_20min_3.lcm		
Batch Filename	:		
Vial #	: 1-61	Sample Type	: Unknown
Injection Volume	: 20 uL		
Date Acquired	: 21/08/2024 13:09:59	Acquired by	: System Administrator
Date Processed	: 21/08/2024 15:34:09	Processed by	: System Administrator

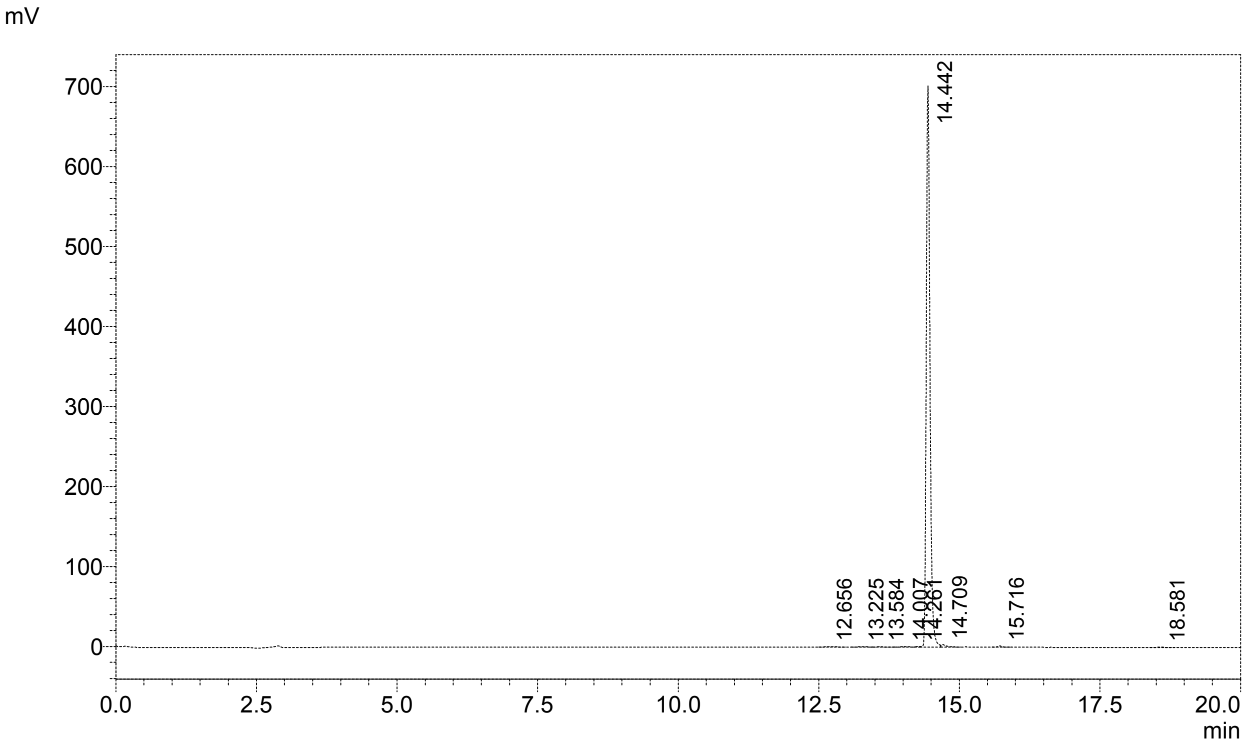

Detector A Channel 2 254nm

Peak#	Ret. Time	Area	Height	Conc.	Unit	Mark	Name
1	12.656	3871	338	0.119		M	
2	13.225	2688	241	0.083		M	
3	13.584	1772	278	0.055		M	
4	14.007	3646	798	0.112		M	
5	14.261	2952	779	0.091		M	
6	14.442	3199877	701279	98.656		V M	
7	14.709	16961	2837	0.523		V M	
8	15.716	9109	1774	0.281		M	
9	18.581	2598	295	0.080		M	
Total		3243473	708619	100.000			

2-*n*-Butoxy-6-[(4′-fluoro-2′,6′-dimethylphenyl)amino]benzamide (7d)

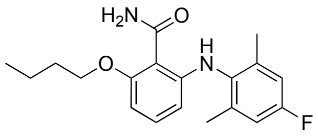

Sample Name	: TEFM01-65_run3		
Sample ID	: TEFM01-65_run3		
Data Filename	: TEFM01-65_run3.lcd		
Method Filename	: EVO_10-90_MeCN_H2O_1mlmin_20min_3.lcm		
Batch Filename	:		
Vial #	: 1-64	Sample Type	: Unknown
Injection Volume	: 10 uL		
Date Acquired	: 22/08/2024 10:38:57	Acquired by	: System Administrator
Date Processed	: 22/08/2024 10:58:58	Processed by	: System Administrator

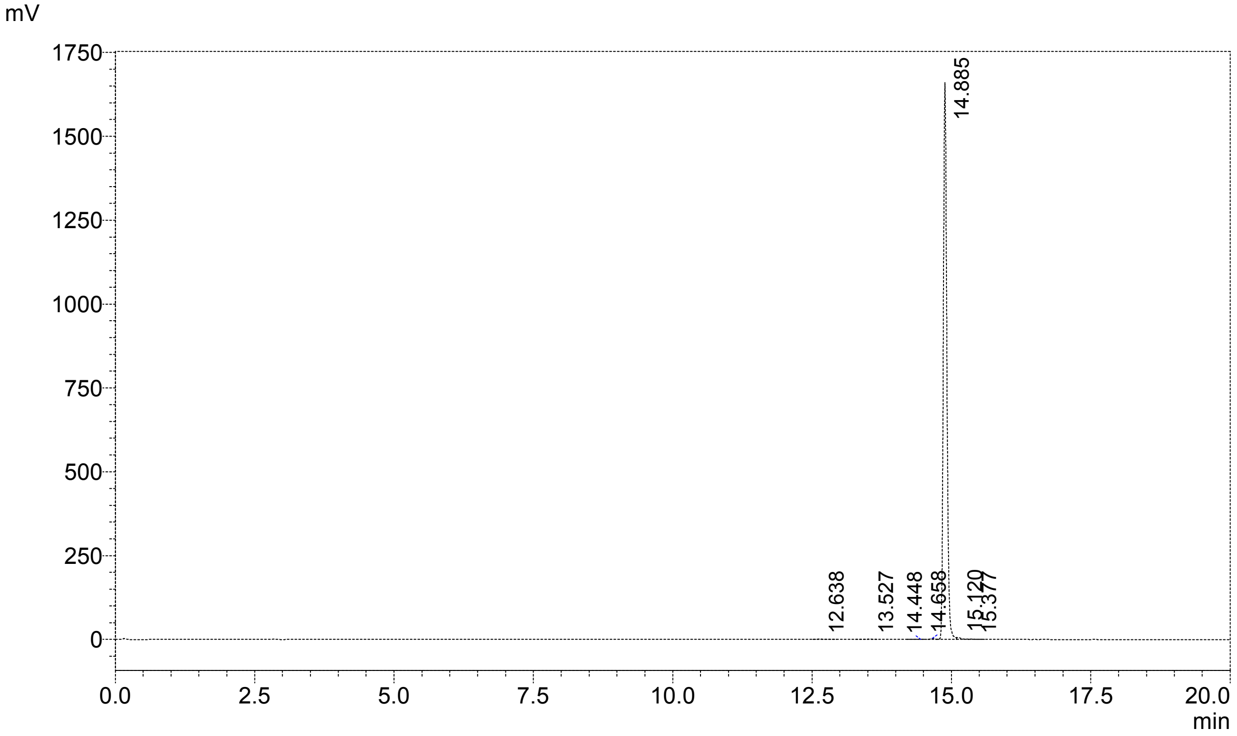

Detector A Channel 2 254nm

Peak#	Ret. Time	Area	Height	Conc.	Unit	Mark	Name
1	12.638	4390	487	0.060		M	
2	13.527	4861	649	0.066		M	
3	14.448	3429	390	0.047		M	
4	14.658	13569	3096	0.185		M	
5	14.885	7273247	1659897	99.117		V M	
6	15.120	32057	5710	0.437		V M	
7	15.377	6523	1098	0.089		V M	
Total		7338076	1671326	100.000			

6-[(4′-Fluoro-2′,6′-dimethylphenyl)amino]-2-(3″-methyl-*n*-butoxy)benzamide (7e)

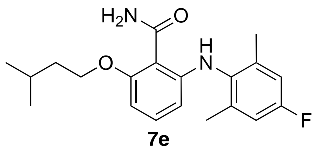

7e

Sample Name	: TEFM01-71		
Sample ID	: TEFM01-71		
Data Filename	: TEFM01-71.lcd		
Method Filename	: EVO_10-90_MeCN_H2O_1mlmin_20min_3.lcm		
Batch Filename	:		
Vial #	: 1-61	Sample Type	: Unknown
Injection Volume	: 20 uL		
Date Acquired	: 22/08/2024 11:28:19	Acquired by	: System Administrator
Date Processed	: 22/08/2024 11:48:20	Processed by	: System Administrator

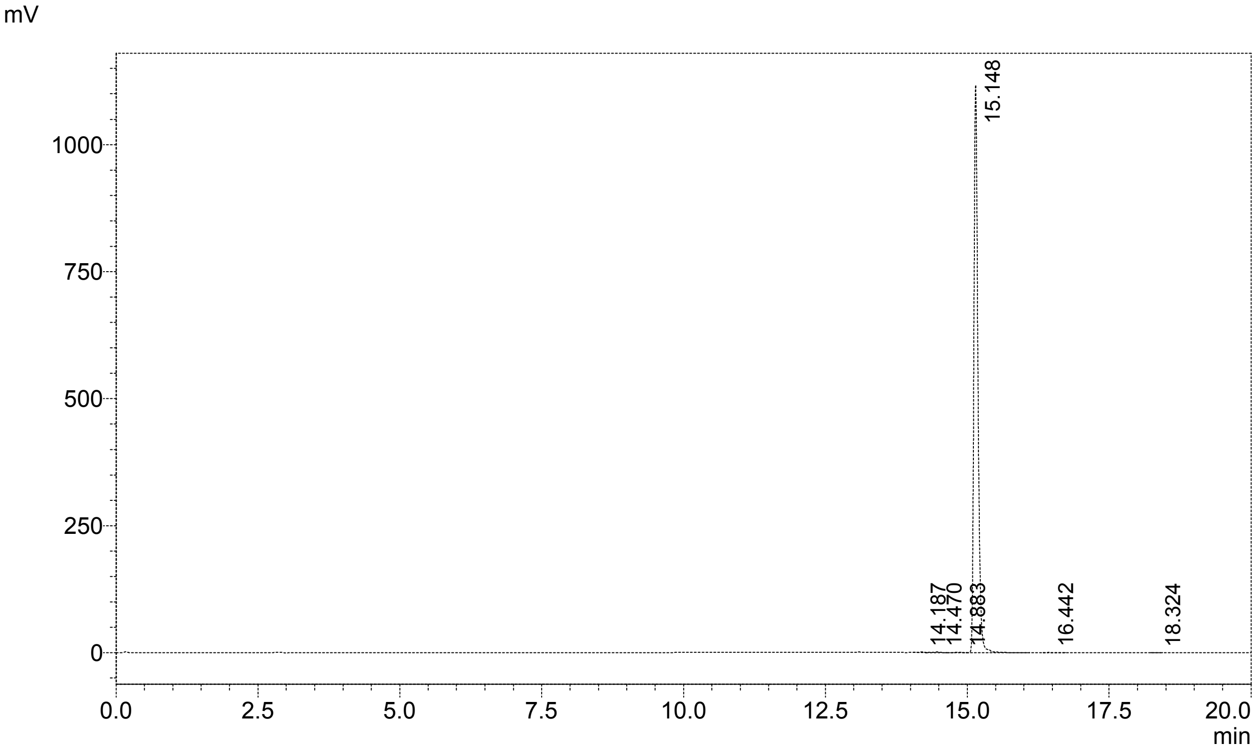

Detector A Channel 2 254nm

Peak#	Ret. Time	Area	Height	Conc.	Unit	Mark	Name
1	14.187	4753	1094	0.085		M	
2	14.470	8463	1158	0.151		M	
3	14.883	4889	546	0.087		M	
4	15.148	5592589	1117320	99.592		M	
5	16.442	2926	531	0.052		M	
6	18.324	1893	364	0.034		M	
Total		5615513	1121013	100.000			

2-(Allyloxy)-6-[(4′-fluoro-2′,6′-dimethylphenyl)amino]benzamide (7f)

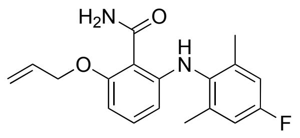

Sample Name	: TEFM01-77		
Sample ID	: TEFM01-77		
Data Filename	: TEFM01-77.lcd		
Method Filename	: EVO_10-90_MeCN_H2O_1mlmin_20min_3.lcm		
Batch Filename	:		
Vial #	: 1-62	Sample Type	: Unknown
Injection Volume	: 30 uL		
Date Acquired	: 21/08/2024 13:32:43	Acquired by	: System Administrator
Date Processed	: 21/08/2024 15:24:35	Processed by	: System Administrator

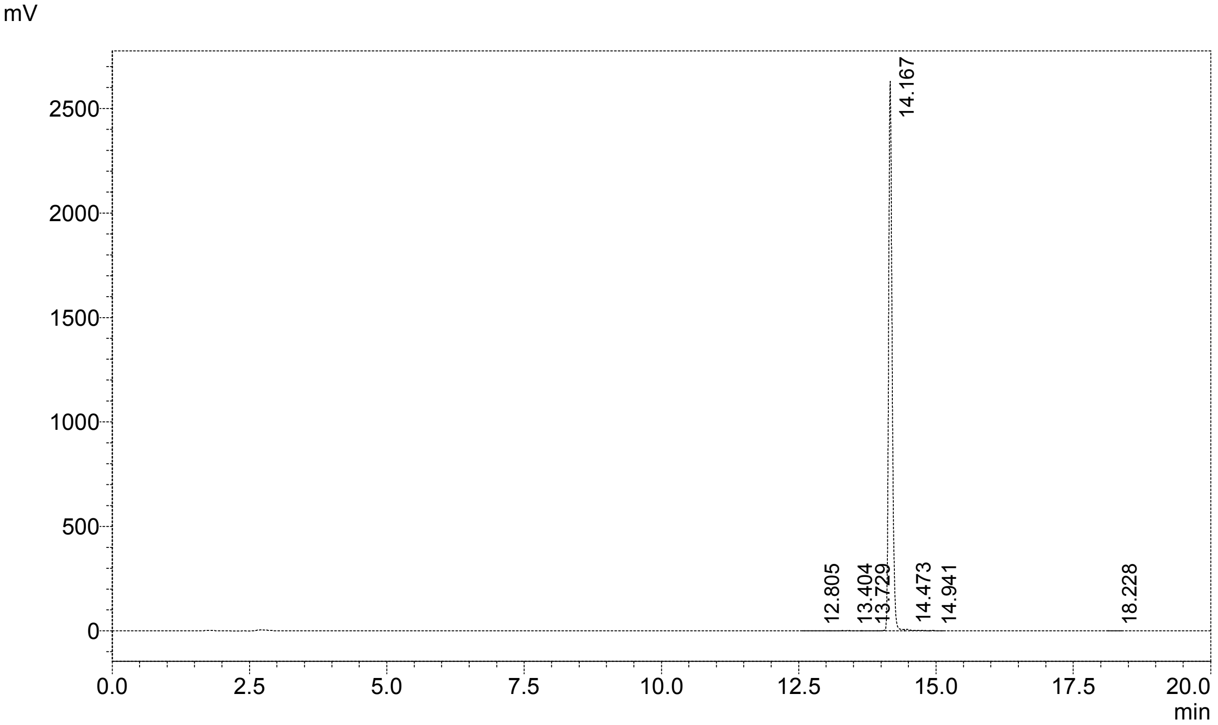

Detector A Channel 2 254nm

Peak#	Ret. Time	Area	Height	Conc.	Unit	Mark	Name
1	12.805	5125	355	0.044		M	
2	13.404	7522	497	0.064		M	
3	13.729	2301	285	0.020		M	
4	14.167	11621032	2628314	99.477		M	
5	14.473	41215	6308	0.353		V M	
6	14.941	2354	608	0.020		M	
7	18.228	2536	451	0.022		M	
Total		11682085	2636818	100.000			

6-[(4′-fluoro-2′,6′-dimethylphenyl)amino]-2-methoxy-3-methylbenzamide (7g, TEFM78)

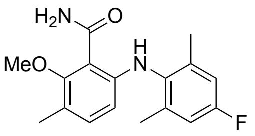

Sample Name	: TEFM01-78_run2		
Sample ID	: TEFM01-78_run2		
Data Filename	: TEFM01-78_run2.lcd		
Method Filename	: EVO_10-90_MeCN_H2O_1mlmin_20min_3.lcm		
Batch Filename	:		
Vial #	: 1-61	Sample Type	: Unknown
Injection Volume	: 5 uL		
Date Acquired	: 22/08/2024 15:20:34	Acquired by	: System Administrator
Date Processed	: 22/08/2024 15:40:34	Processed by	: System Administrator

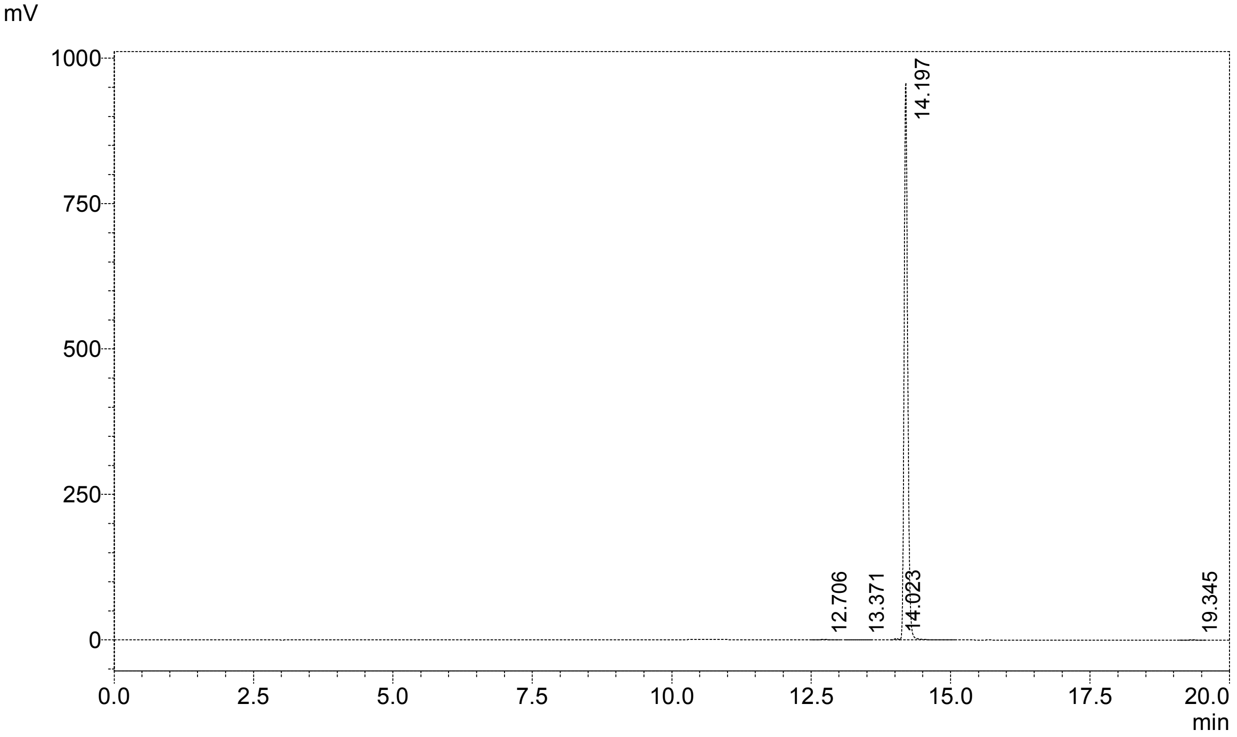

Detector A Channel 2 254nm

Peak#	Ret. Time	Area	Height	Conc.	Unit	Mark	Name
1	12.706	3607	281	0.085		M	
2	13.371	2089	220	0.049		M	
3	14.023	10814	2505	0.256			
4	14.197	4208943	957689	99.553		SV	
5	19.345	2403	302	0.057		M	
Total		4227856	960998	100.000			

3-(4″-Fluorophenyl)-6-(mesitylamino)-2-methoxybenzamide (8a)

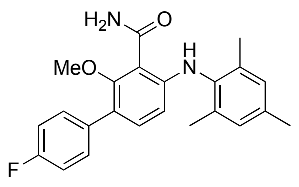

Sample Name	: TEFM01-126
Sample ID	: TEFM01-126
Data Filename	: TEFM01-126.lcd
Method Filename	: EVO_10-90_MeCN_H2O_1mlmin_20min_3.lcm
Batch Filename	:
Vial #	: 1-62
Injection Volume	: 10 uL
Date Acquired	: 22/08/2024 09:46:16
Date Processed	: 22/08/2024 10:29:45

Sample Type	: Unknown
Acquired by	: System Administrator
Processed by	: System Administrator

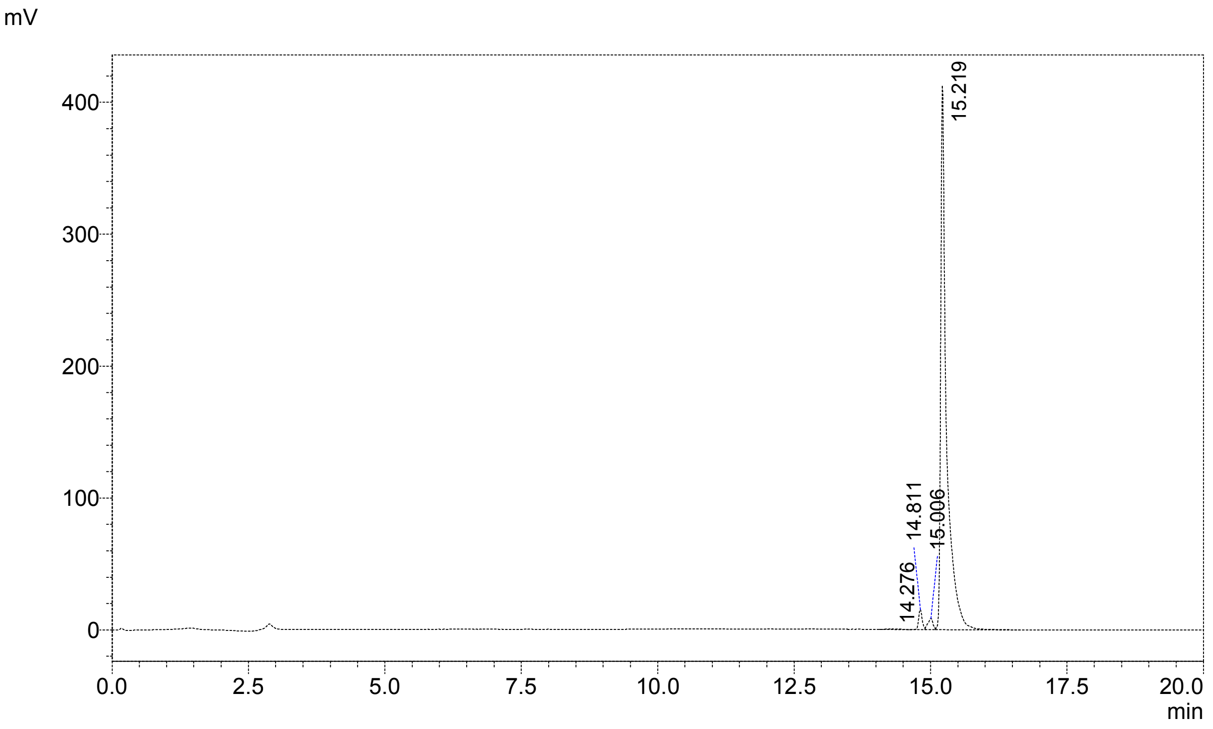

Detector A Channel 2 254nm

Peak#	Ret. Time	Area	Height	Conc.	Unit	Mark	Name
1	14.276	7139	588	0.224		M	
2	14.811	69093	15223	2.166			
3	15.006	53149	8528	1.666		V	
4	15.219	3060052	412611	95.943		V	
Total		3189434	436950	100.000			

3-(2′′-Fluorophenyl)-6-(mesitylamino)-2-methoxybenzamide (8b)

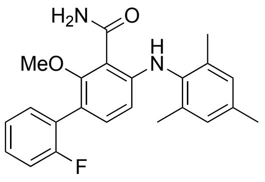

Sample Name	: TEFM01-132		
Sample ID	: TEFM01-132		
Data Filename	: TEFM01-132.lcd		
Method Filename	: EVO_10-90_MeCN_H2O_1mlmin_20min_3.lcm		
Batch Filename	:		
Vial #	: 1-63	Sample Type	: Unknown
Injection Volume	: 10 uL		
Date Acquired	: 22/08/2024 10:12:02	Acquired by	: System Administrator
Date Processed	: 22/08/2024 10:32:03	Processed by	: System Administrator

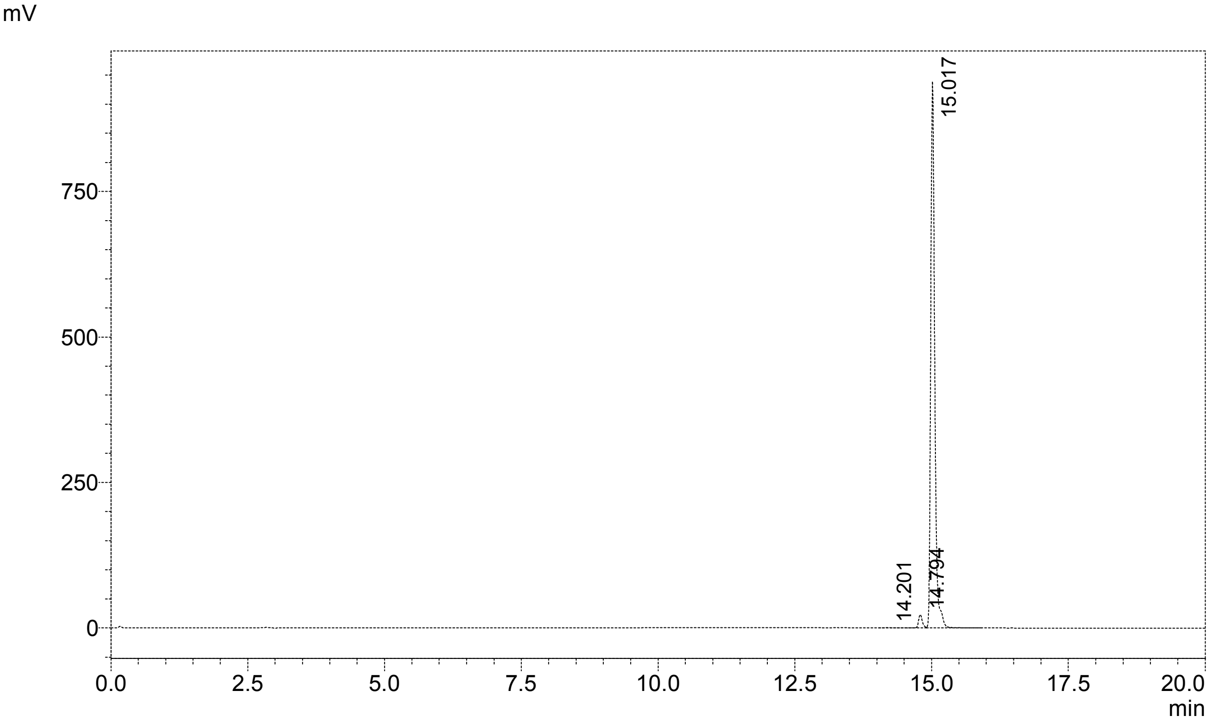

Detector A Channel 2 254nm

Peak#	Ret. Time	Area	Height	Conc.	Unit	Mark	Name
1	14.201	7059	540	0.148		M	
2	14.794	111847	22929	2.340			
3	15.017	4660011	939208	97.512		SV	
Total		4778916	962677	100.000			

3-(2″-Fluoro-4″-methoxyphenyl)-6-(mesitylamino)-2-methoxybenzamide (8c)

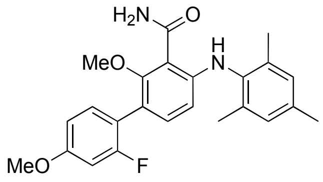

Sample Name	: TEFM01-138_run2
Sample ID	: TEFM01-138_run2
Data Filename	: TEFM01-138_run2.lcd
Method Filename	: EVO_10-90_MeCN_H2O_1mlmin_20min_3.lcm
Batch Filename	:
Vial #	: 1-61
Injection Volume	: 10 uL
Date Acquired	: 22/08/2024 09:23:11
Date Processed	: 22/08/2024 09:53:15
Sample Type	: Unknown
Acquired by	: System Administrator
Processed by	: System Administrator

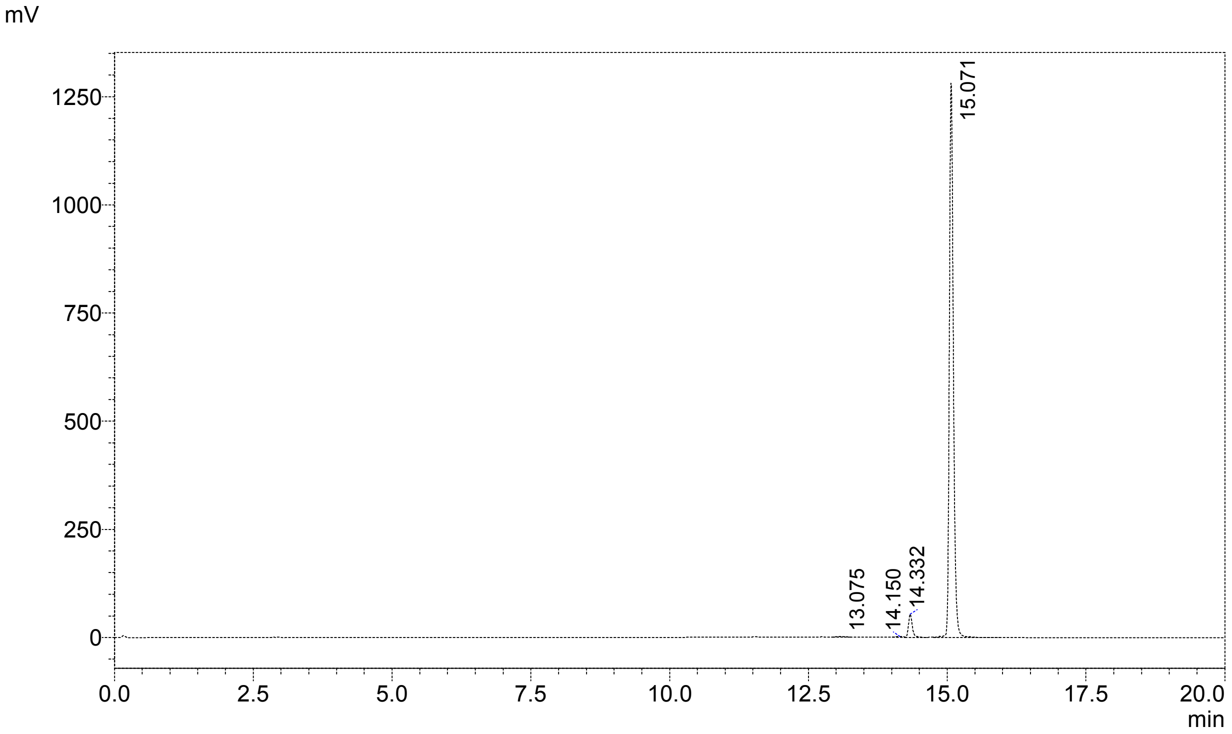

Detector A Channel 2 254nm

Peak#	Ret. Time	Area	Height	Conc.	Unit	Mark	Name
1	13.075	8922	983	0.132		M	
2	14.150	15505	1916	0.230		M	
3	14.332	258264	53505	3.834		M	
4	15.071	6453609	1280642	95.803		M	
Total		6736301	1337045	100.000			

3-(2″-fluoro-5″-methoxyphenyl)-6-(mesitylamino)-2-methoxybenzamide (8d)

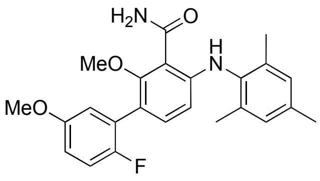

Sample Name	: TEFM01-139		
Sample ID	: TEFM01-139		
Data Filename	: TEFM01-139.lcd		
Method Filename	: EVO_10-90_MeCN_H2O_1mlmin_20min_3.lcm		
Batch Filename	:		
Vial #	: 1-64	Sample Type	: Unknown
Injection Volume	: 30 uL		
Date Acquired	: 21/08/2024 14:18:32	Acquired by	: System Administrator
Date Processed	: 21/08/2024 15:59:54	Processed by	: System Administrator

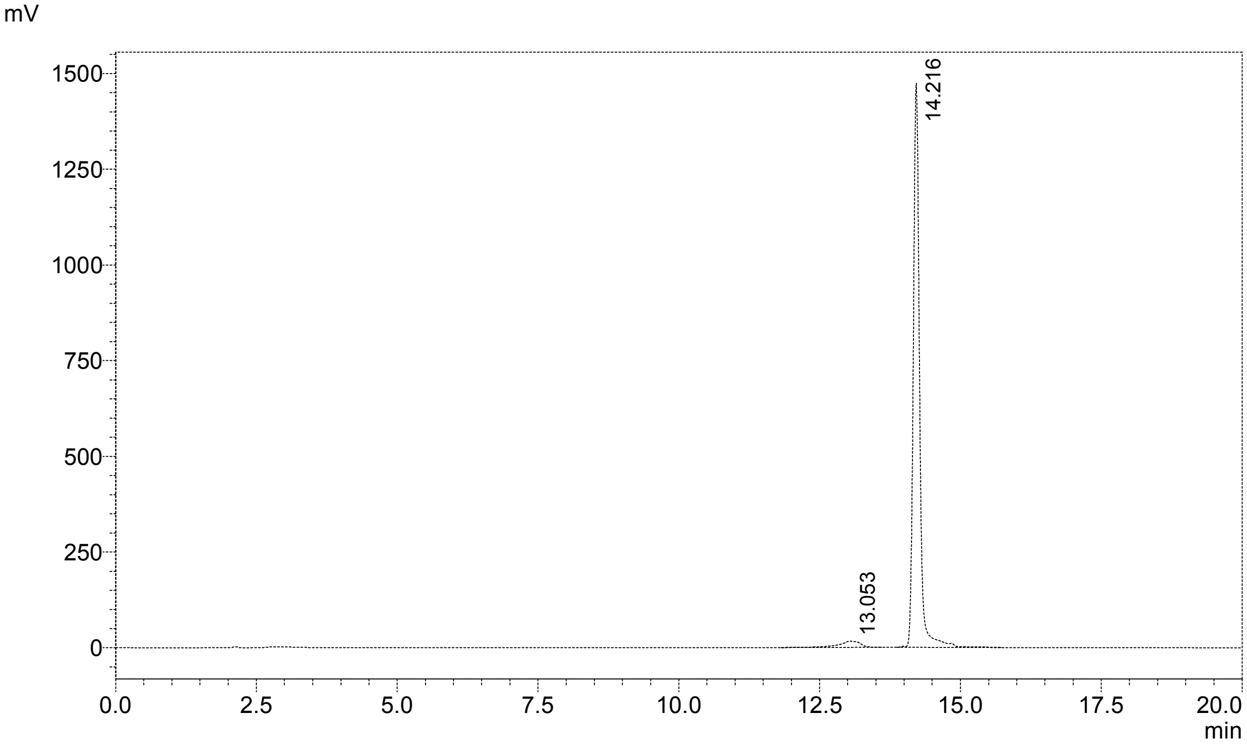

Detector A Channel 2 254nm

Peak#	Ret. Time	Area	Height	Conc.	Unit	Mark	Name
1	13.053	433663	16140	3.702		M	
2	14.216	11279865	1472721	96.298		M	
Total		11713528	1488861	100.000			

3-(2″-fluoropyridin-3″-yl)-6-(mesitylamino)-2-methoxybenzamide (8e)

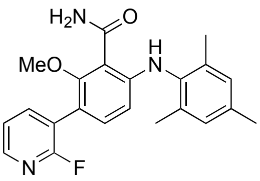

Sample Name	: TEFM01-140_run2		
Sample ID	: TEFM01-140_run2		
Data Filename	: TEFM01-140_run2.lcd		
Method Filename	: EVO_10-90_MeCN_H2O_1mlmin_20min_3.lcm		
Batch Filename	:		
Vial #	: 1-61	Sample Type	: Unknown
Injection Volume	: 20 uL		
Date Acquired	: 22/08/2024 11:01:32	Acquired by	: System Administrator
Date Processed	: 22/08/2024 11:21:33	Processed by	: System Administrator

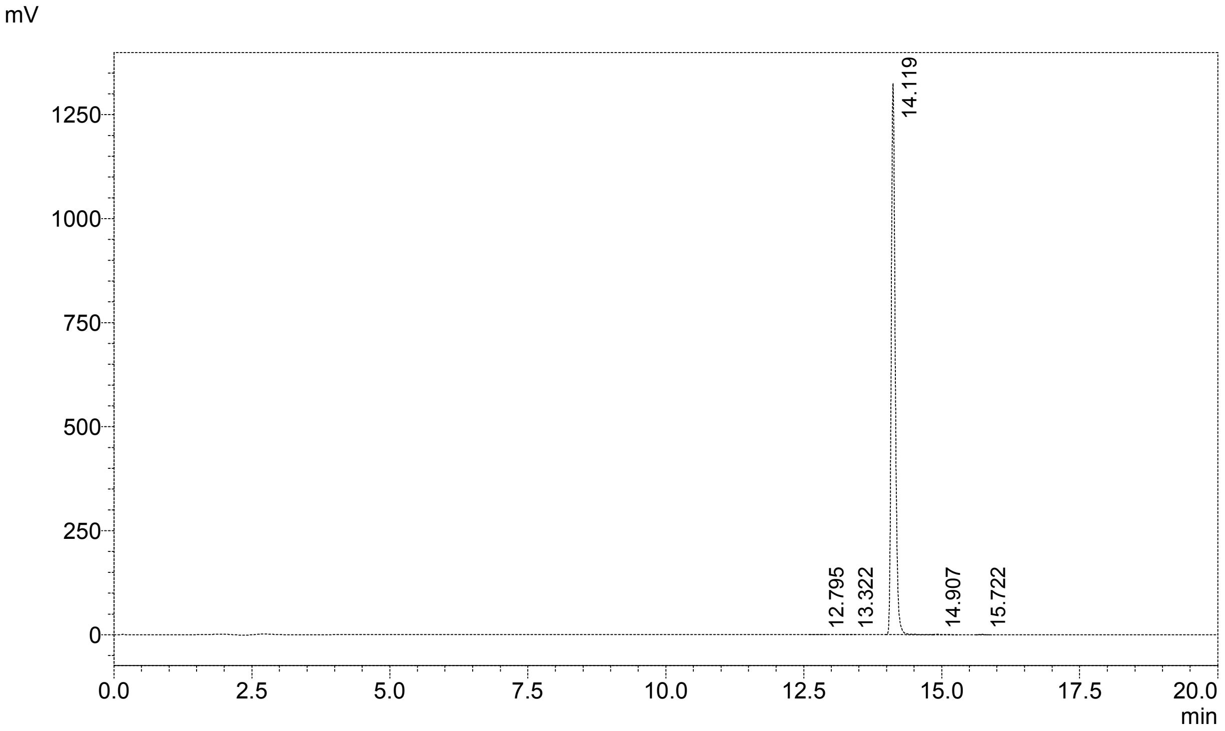

Detector A Channel 2 254nm

Peak#	Ret. Time	Area	Height	Conc.	Unit	Mark	Name
1	12.795	2181	214	0.033		M	
2	13.322	2227	348	0.033		M	
3	14.119	6679441	1325166	99.712		M	
4	14.907	9119	1111	0.136		V M	
5	15.722	5754	1172	0.086		M	
Total		6698721	1328010	100.000			

4. HPLC Traces for [^{18}F]TEFM78

Blue: standard UV chromatogram of TEFM78.

Black: UV chromatogram of [^{18}F]TEFM78 production.

Red: Radio-HPLC chromatogram of [^{18}F]TEFM78 production.

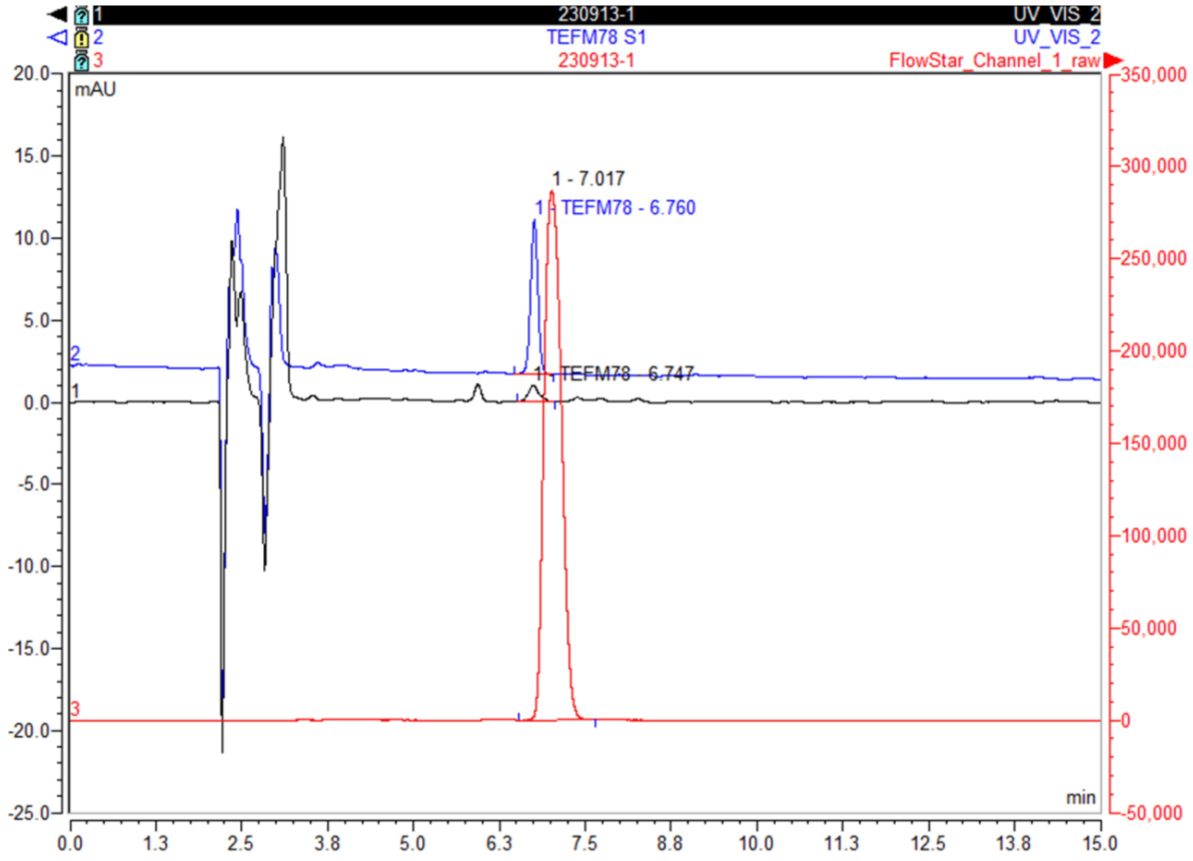

5. Other Docked Poses of TEFM78 Generated by GOLD

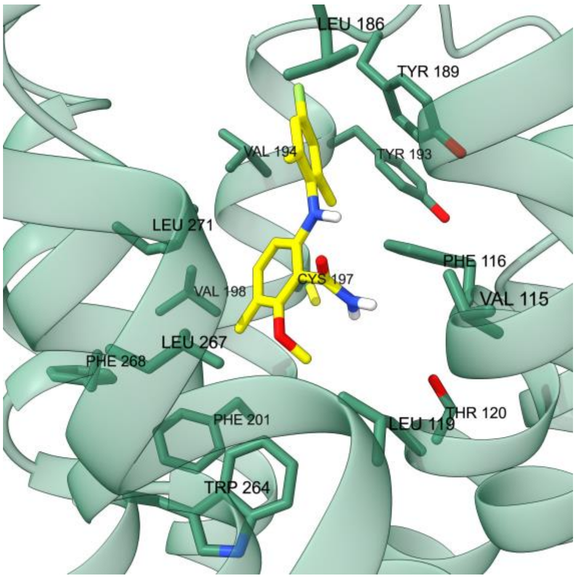

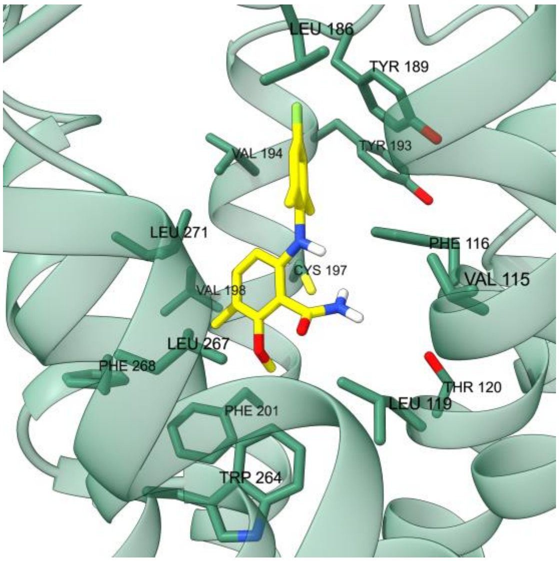

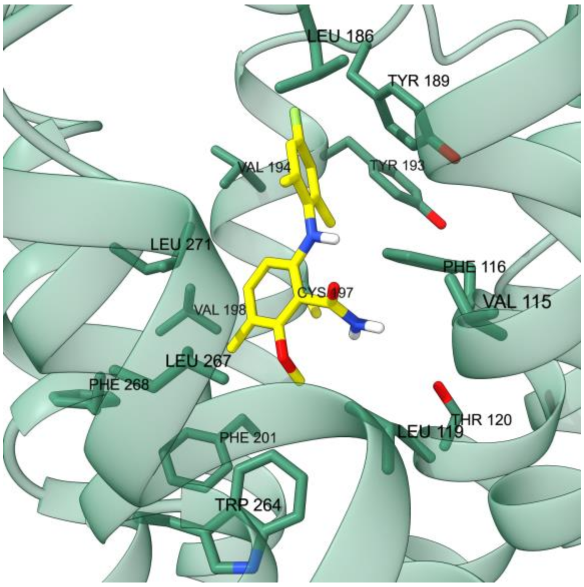

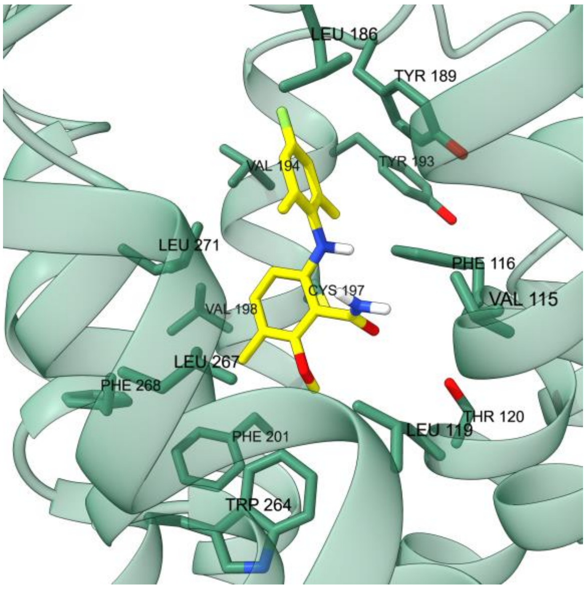

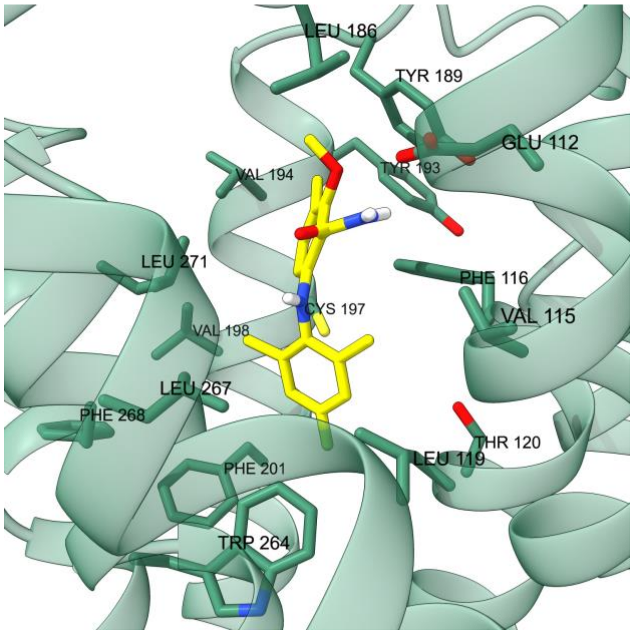

6. References

1. Tavares, A. A. S.; Lewsey, J.; Dewar, D.; Pimlott, S. L. Radiotracer Properties Determined by High Performance Liquid Chromatography: A Potential Tool for Brain Radiotracer Discovery. *Nucl. Med. Biol.* **2012**, *39*, 127–135.
2. Harrison, C.; Traynor, J. R. The [^{35}S]GTPγS Binding Assay: Approaches and Applications in Pharmacology. *Life Sci*. **2003**, *74*, 489–508.

11a: 1H NMR (500 MHz, CDCl3)

7. 1H and 13C NMR Spectra for all Compounds

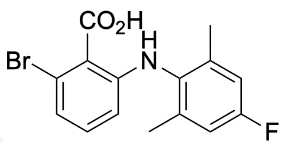

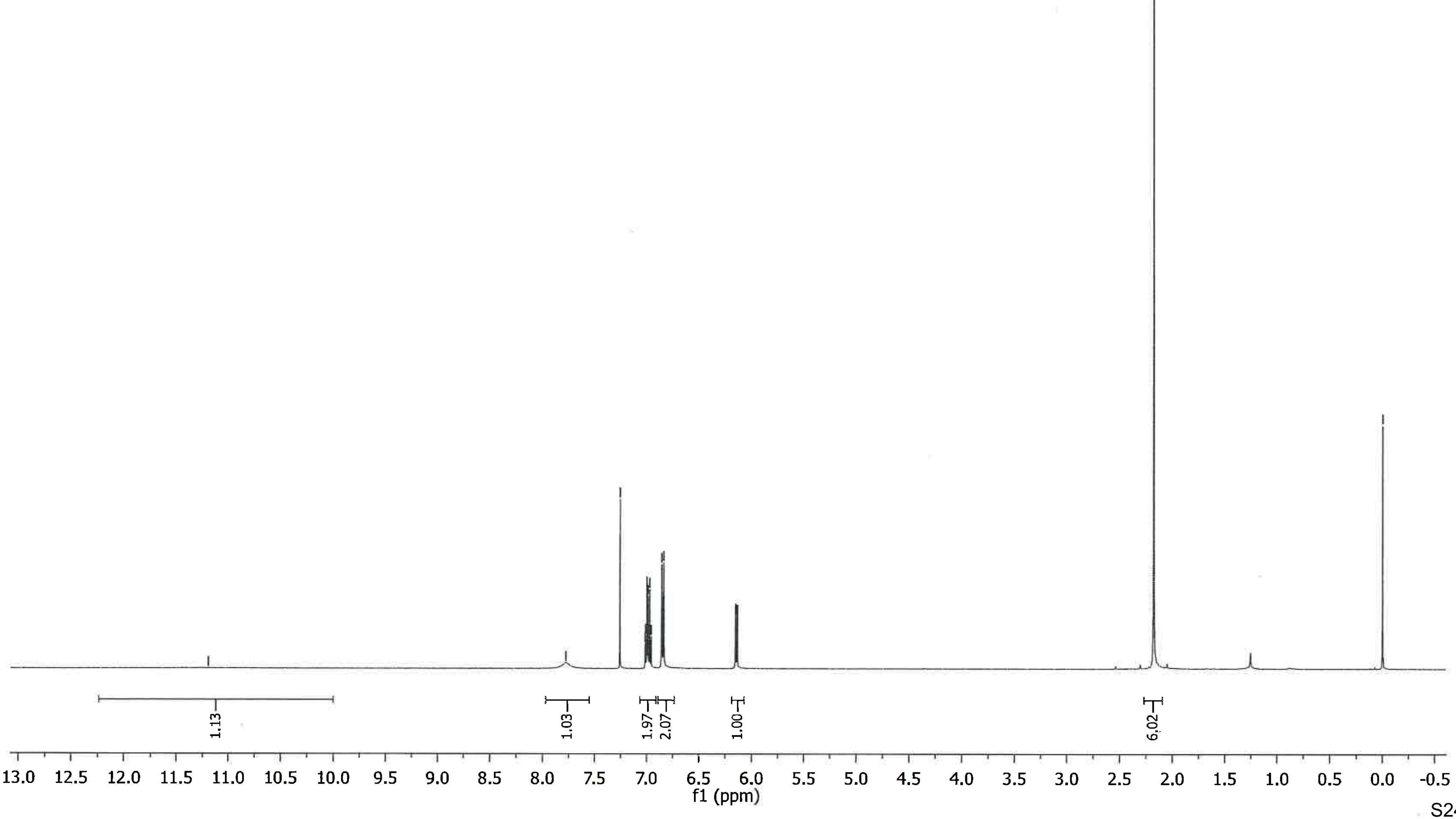

11a: 13C{1H} NMR (126 MHz, CDCl3)

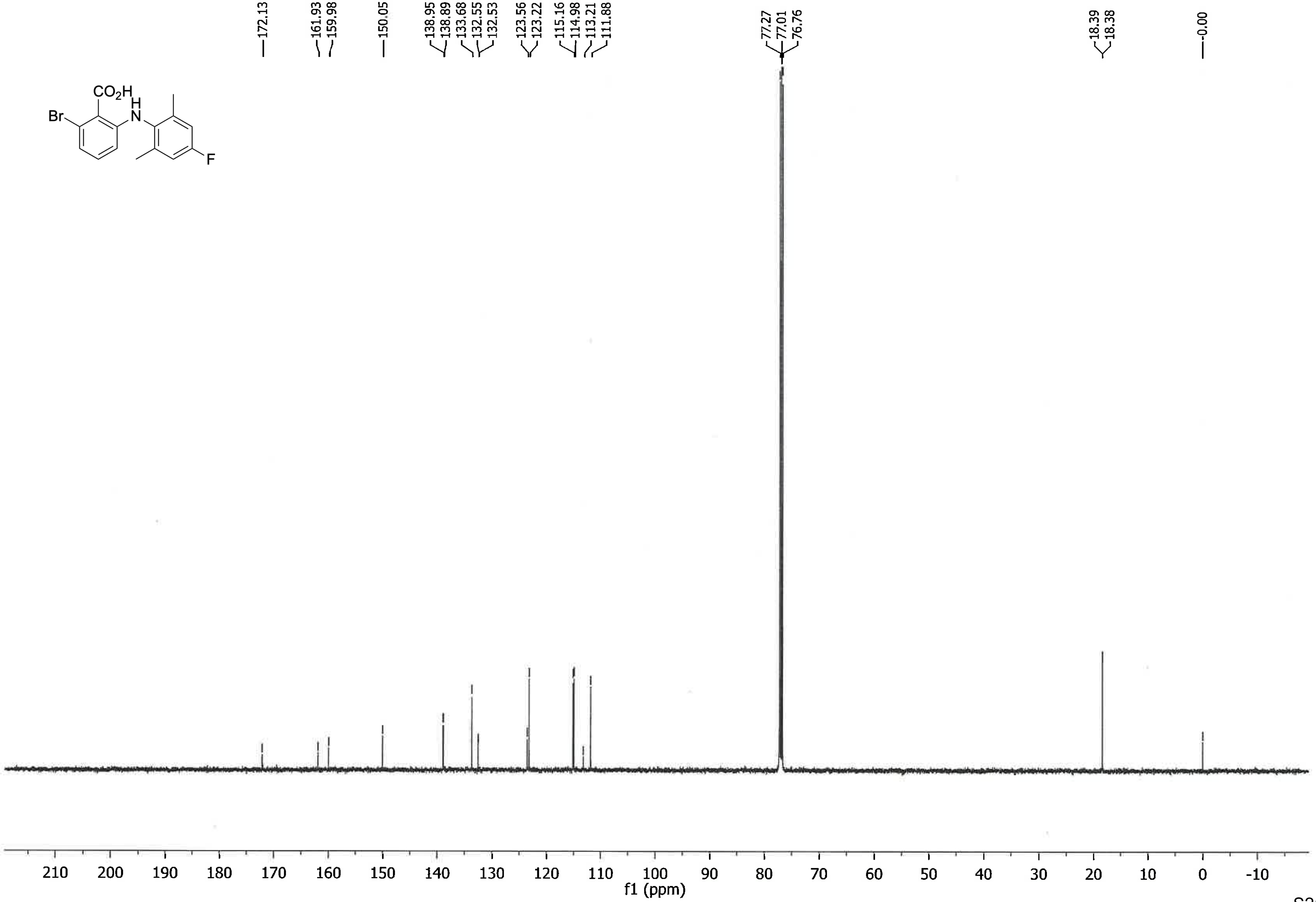

11b: 1H NMR (400 MHz, CDCl3)

Parameter	Value
Spectrometer Frequency	400.13

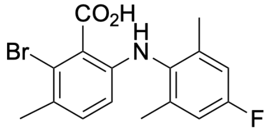

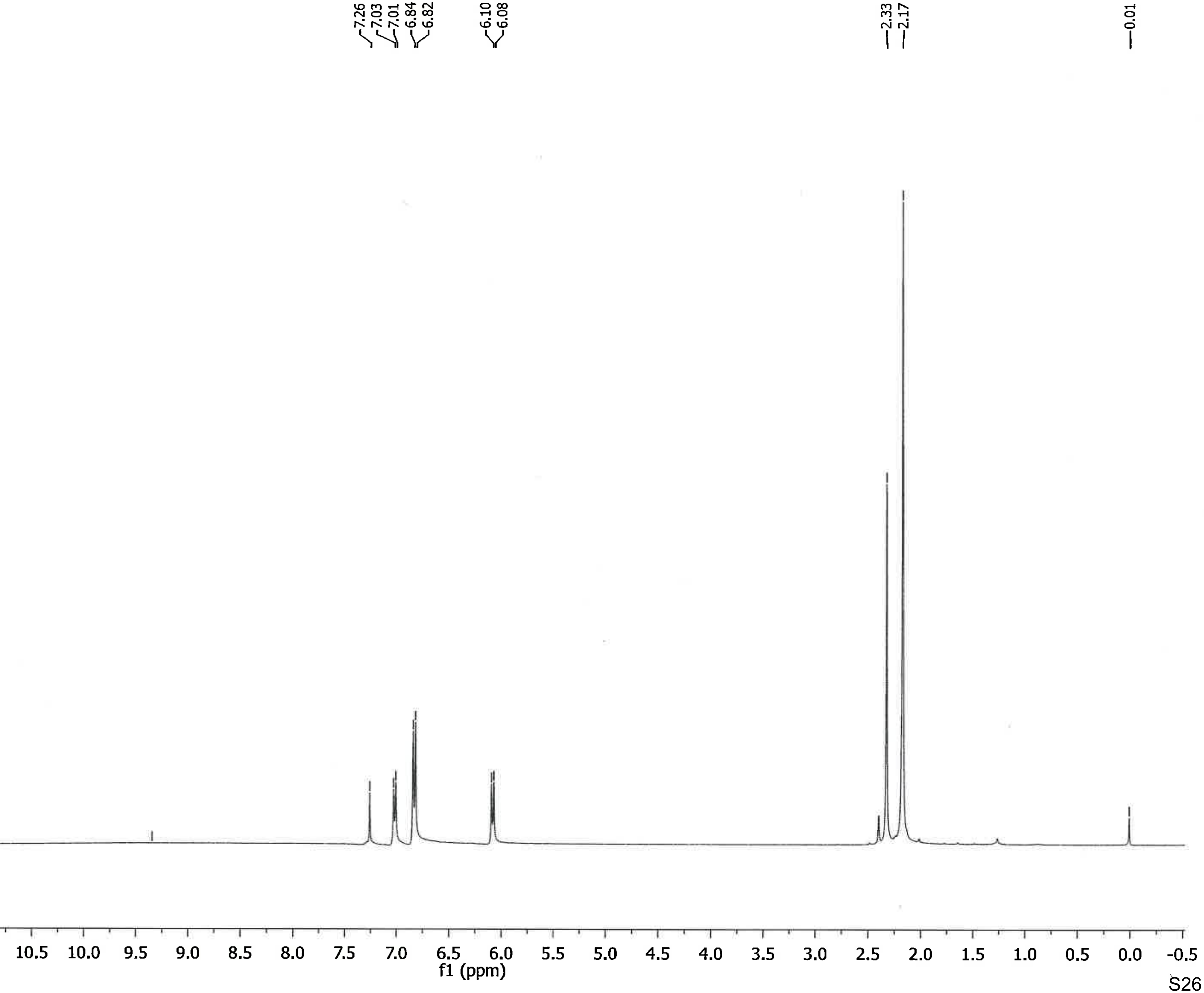

11b: 13C{1H} NMR (101 MHz, CDCl3)

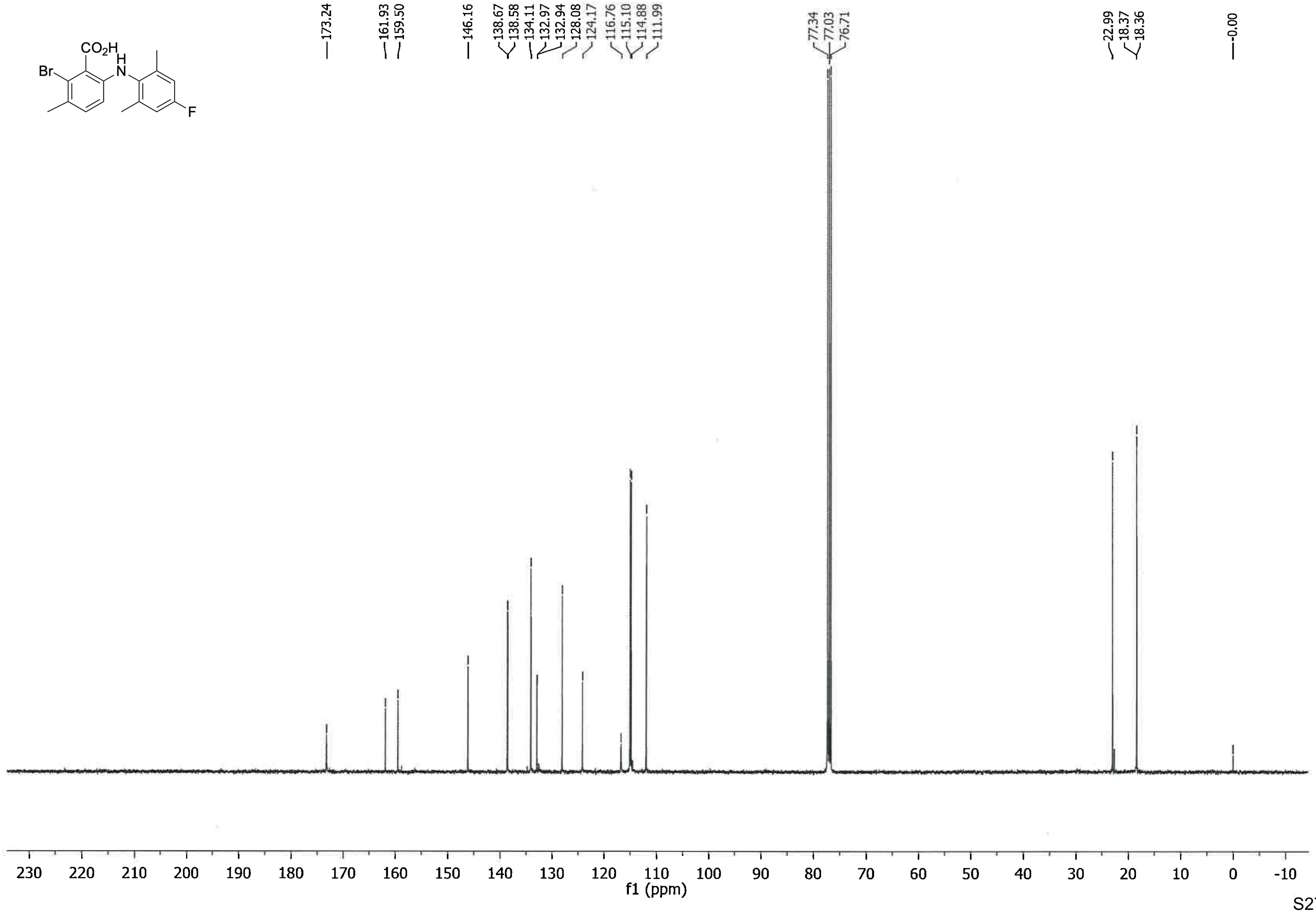

12a: 1H NMR (400 MHz, CDCl3)

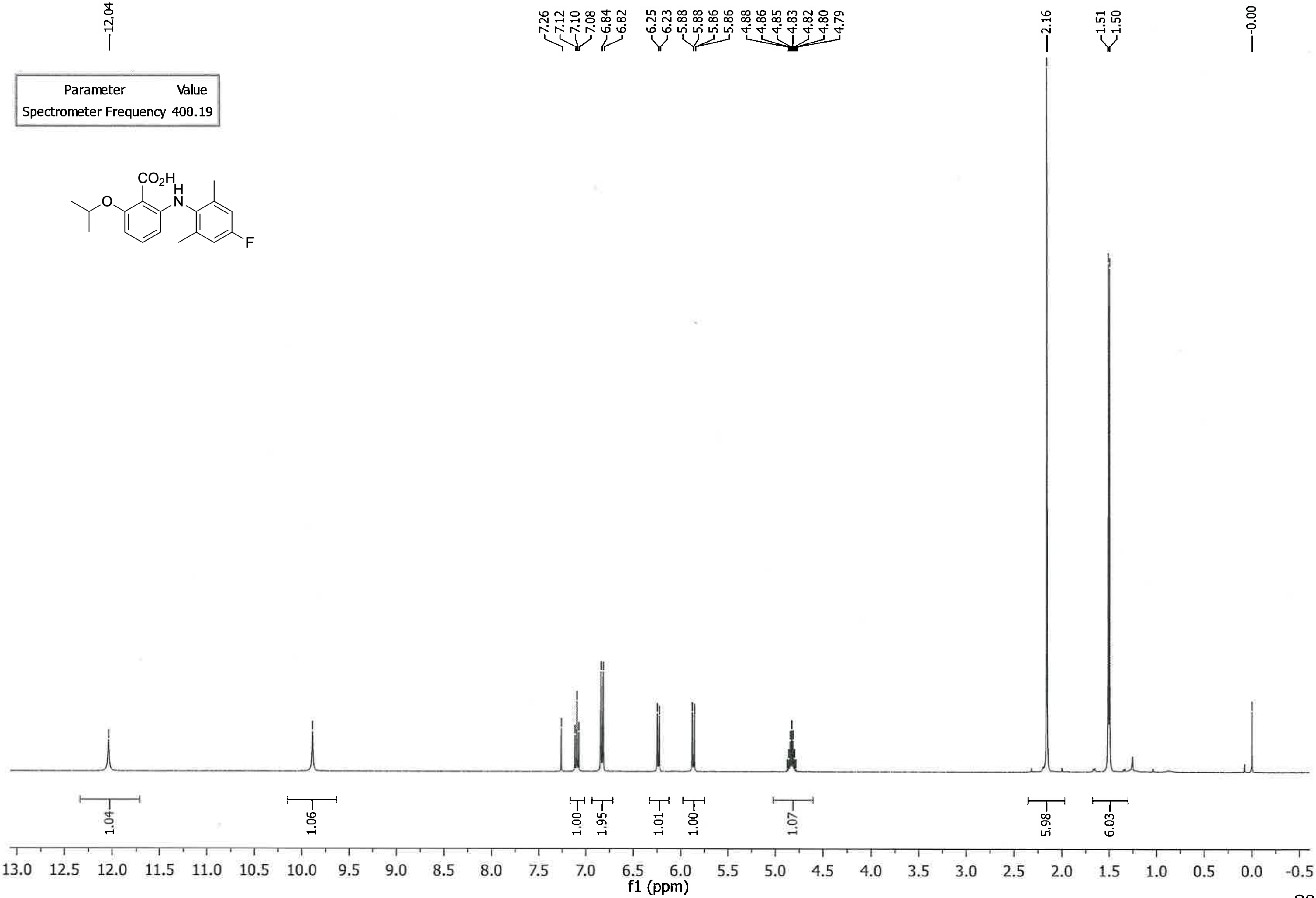

12a: 13C{1H} NMR (101 MHz, CDCl3)

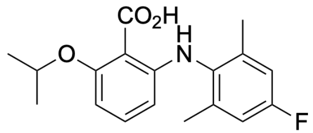

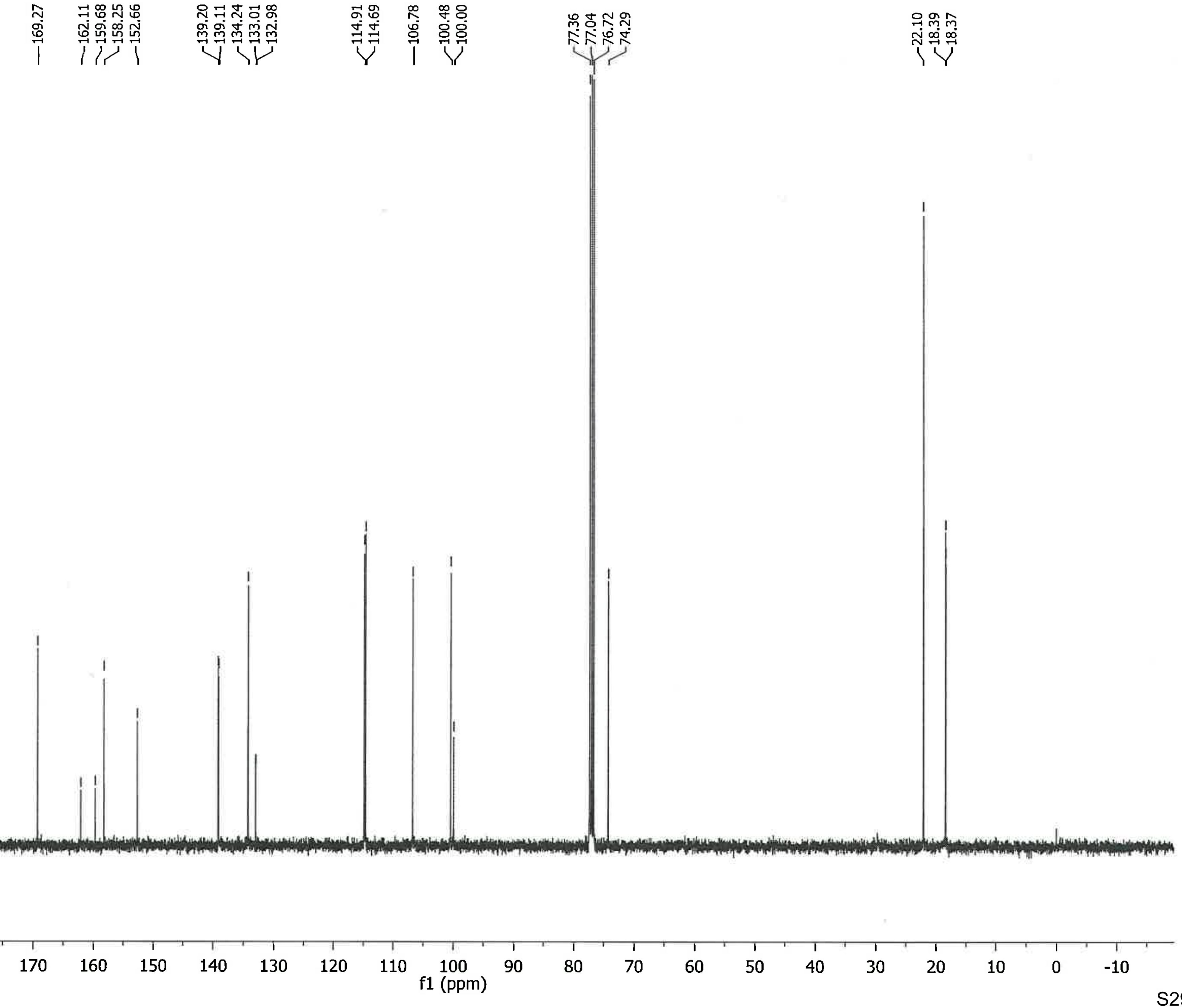

12b: 1H NMR (500 MHz, CDCl3)

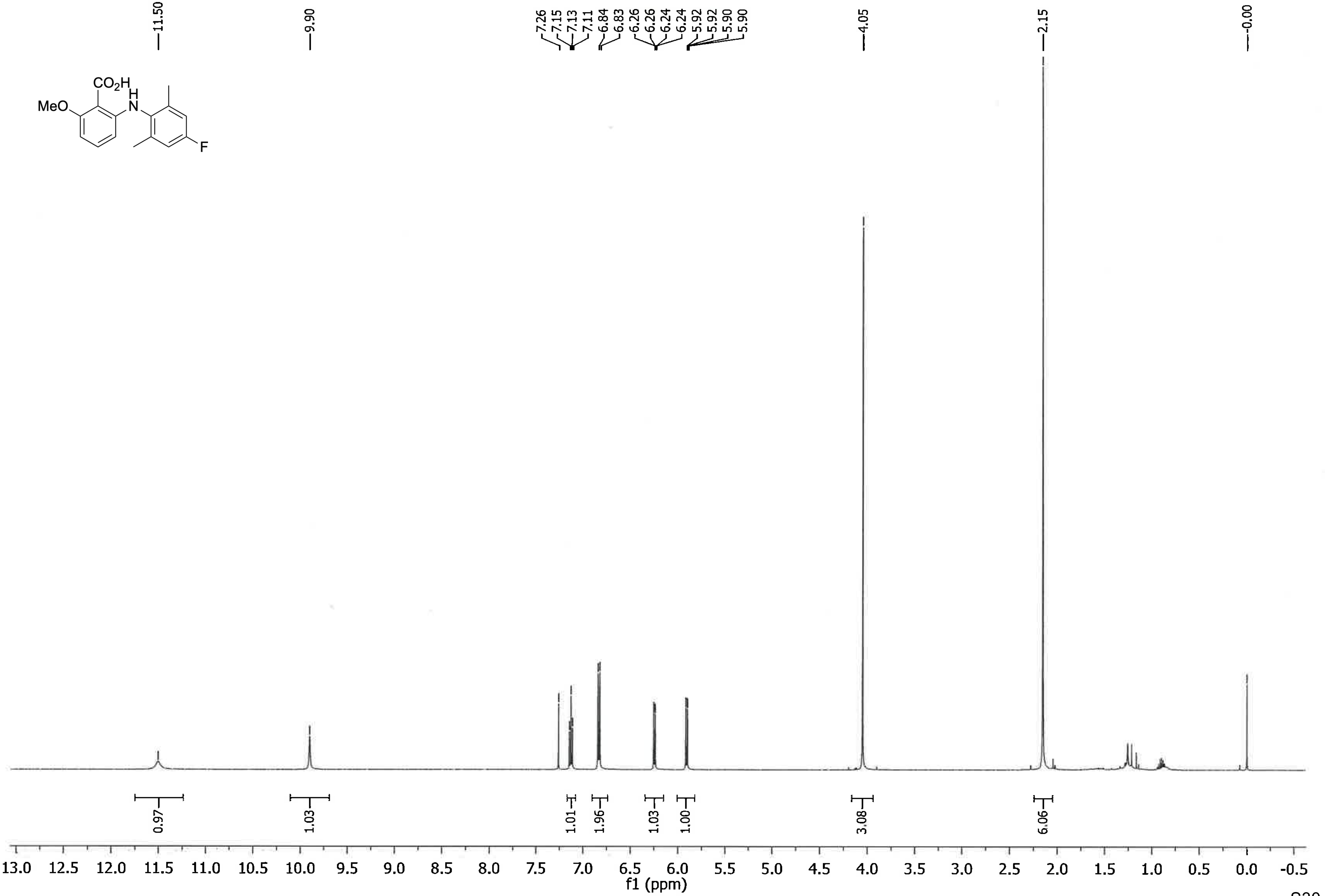

12b: 13C{1H} NMR (126 MHz, CDCl3)

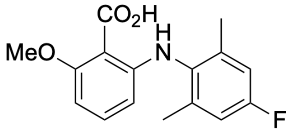

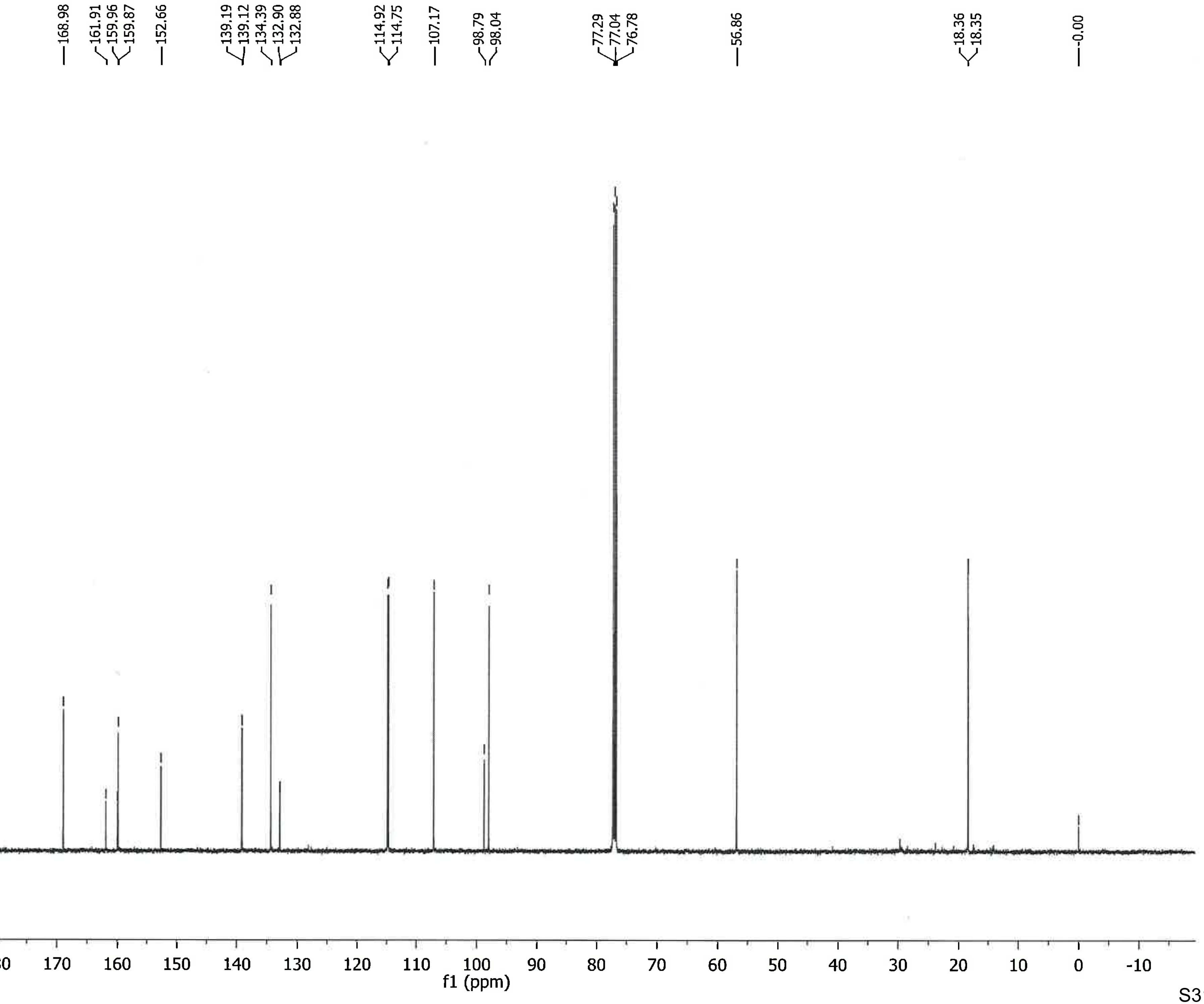

12c: 1H NMR (500 MHz, CDCl3)

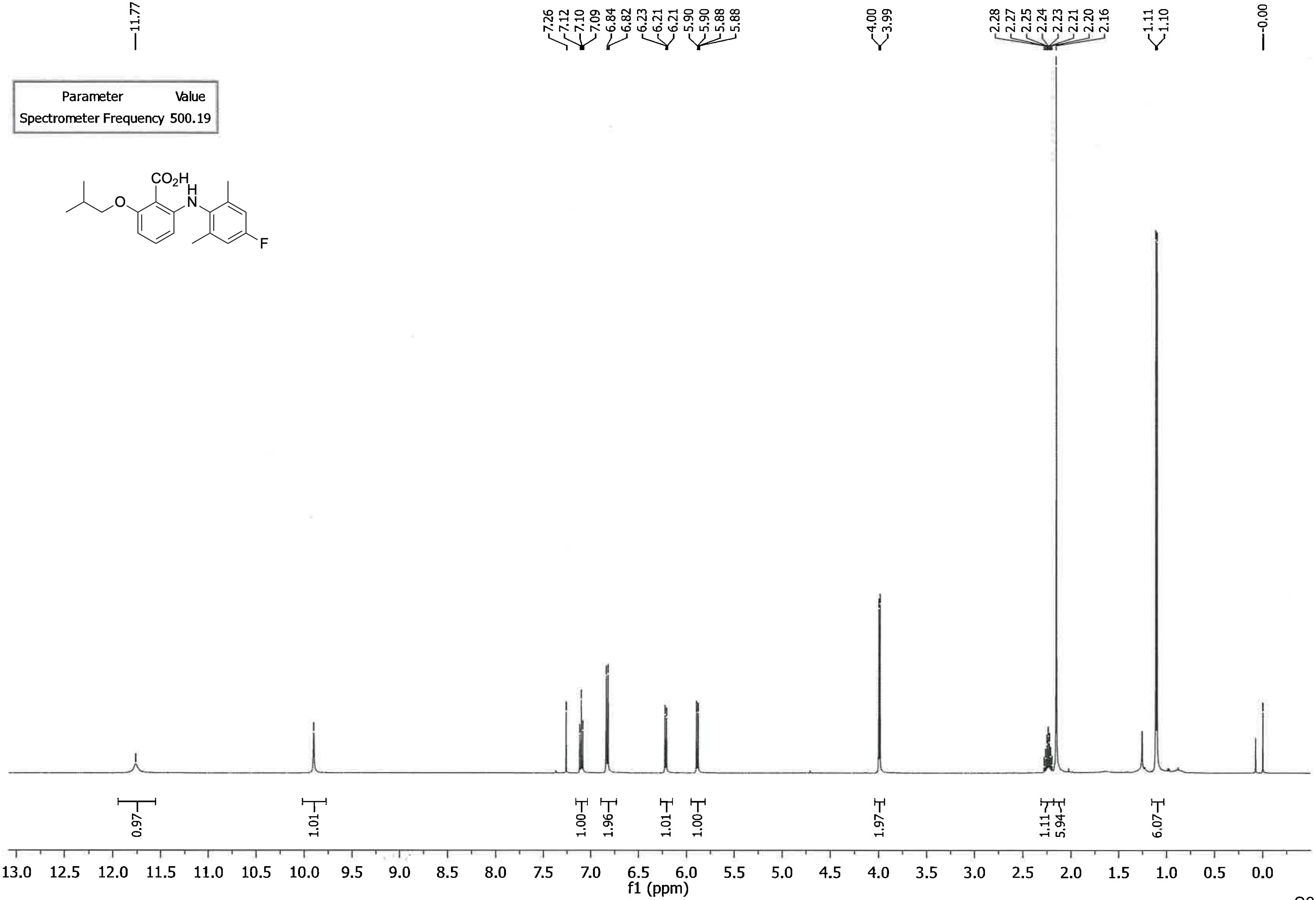

12c: 13C{1H} NMR (126 MHz, CDCl3)

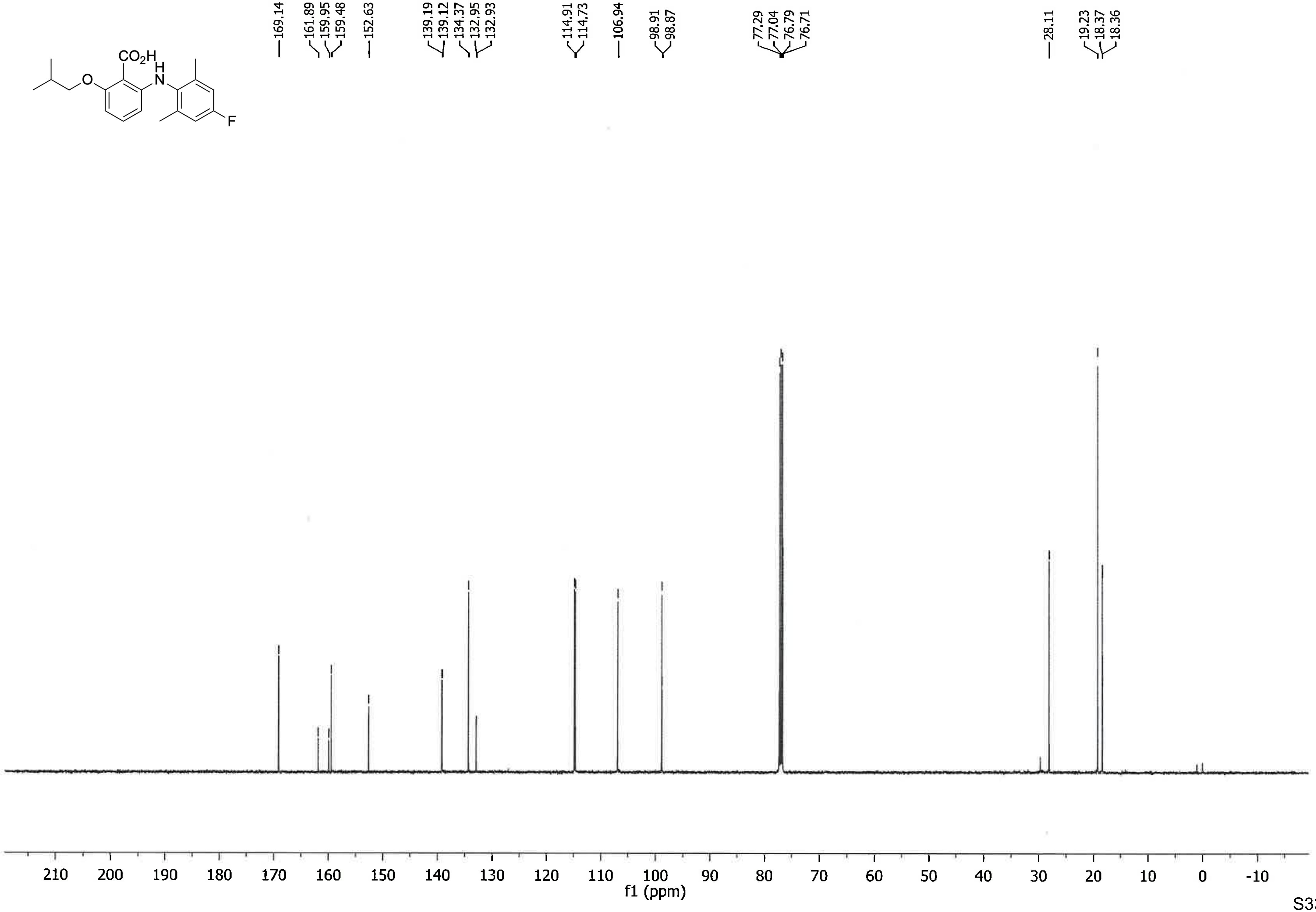

12d: 1H NMR (500 MHz, CDCl3)

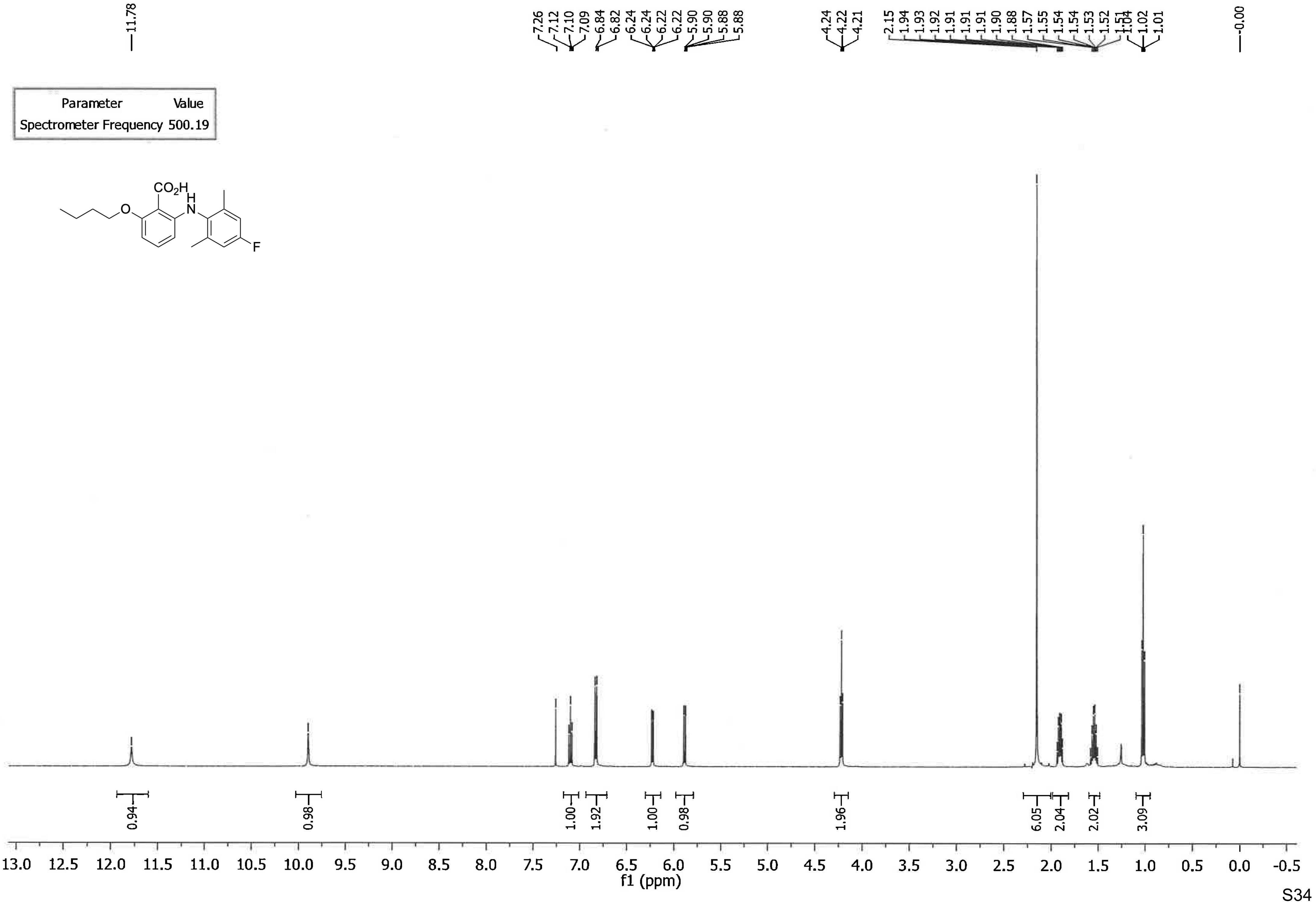

12d: 13C{1H} NMR (126 MHz, CDCl3)

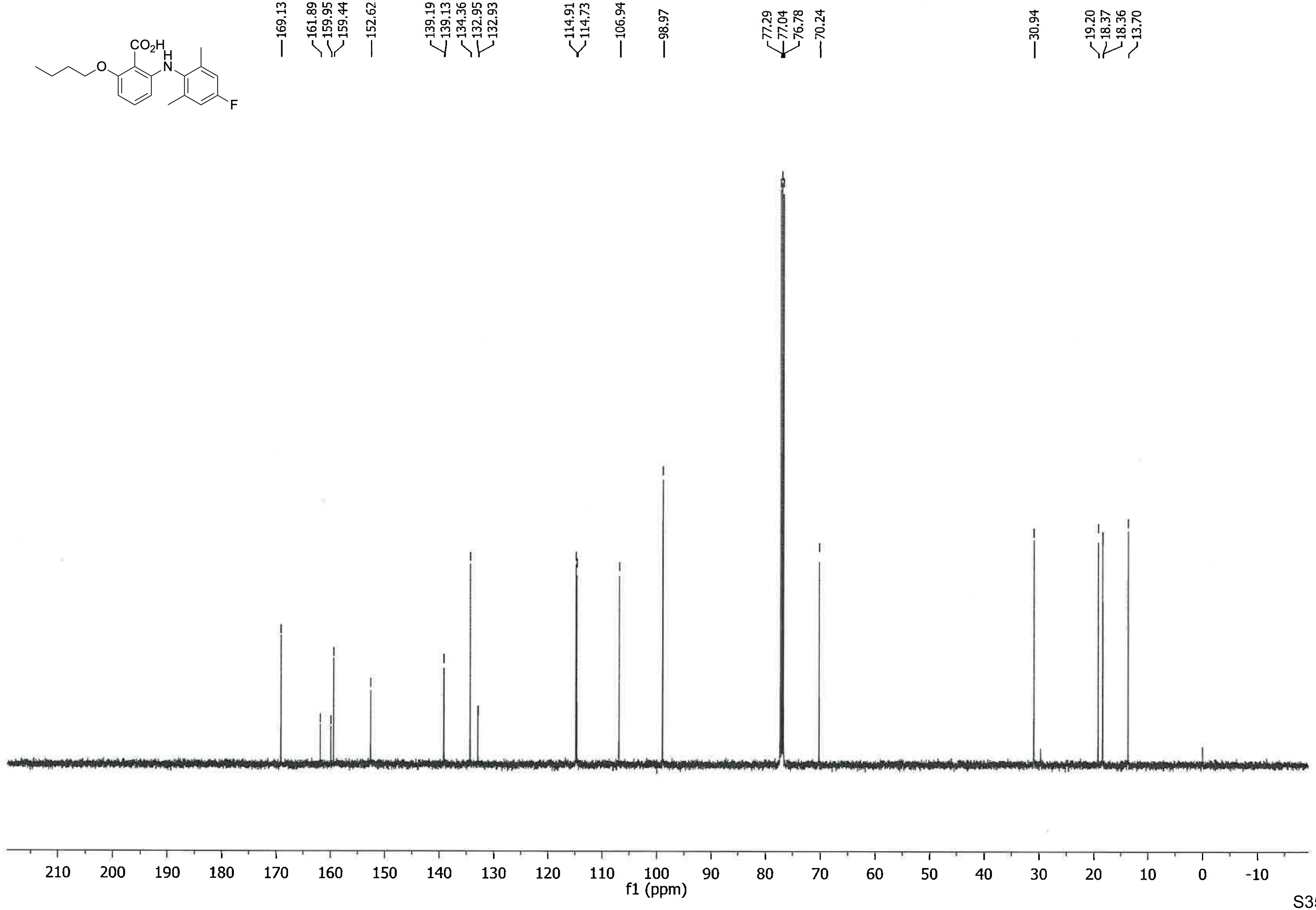

12e: 1H NMR (400 MHz, CDCl3)

Parameter	Value
Spectrometer Frequency	400.19

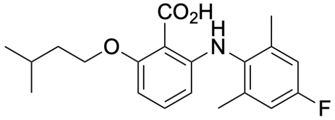

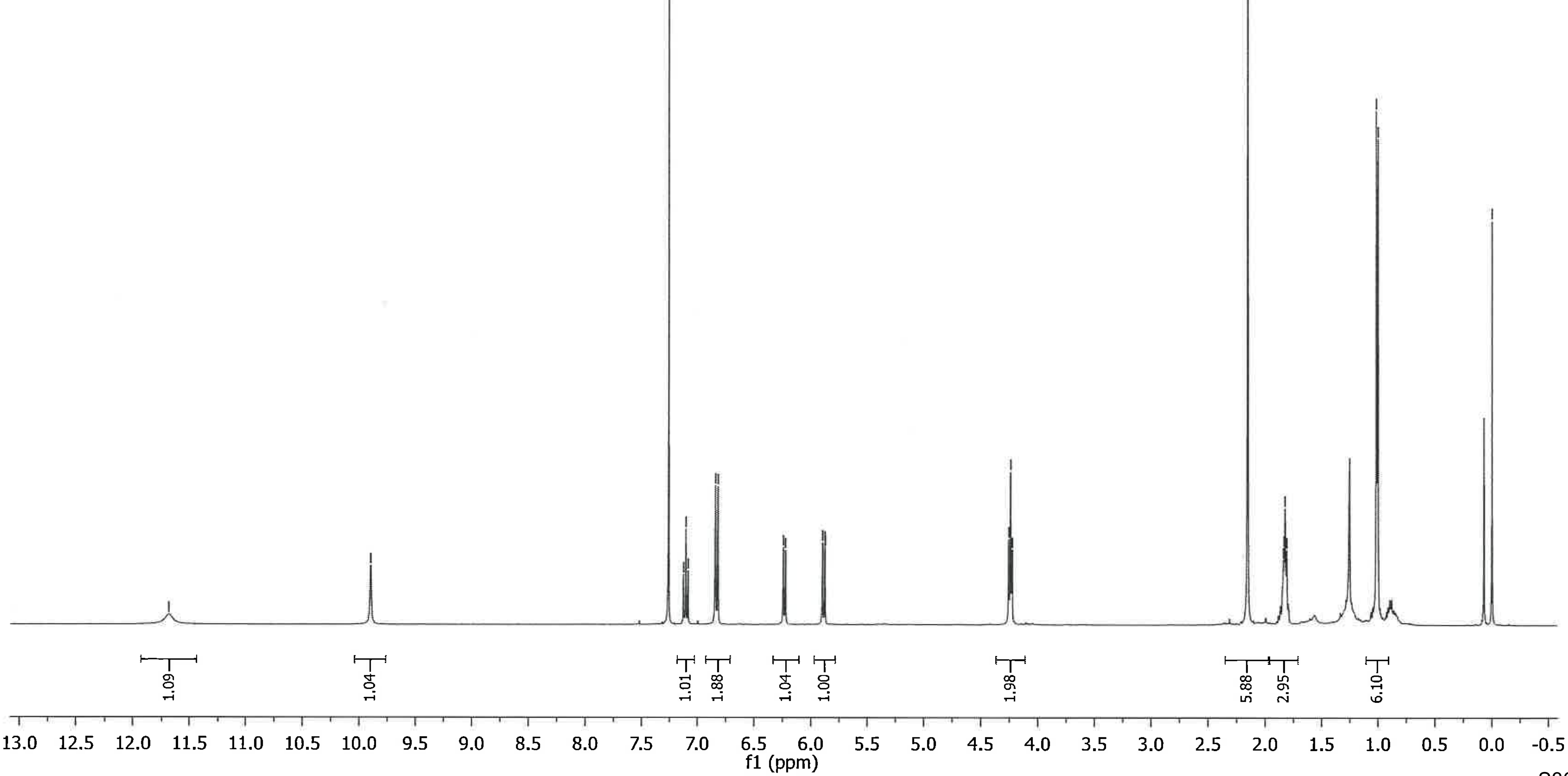

12e: 13C{1H} NMR (126 MHz, CDCl3)

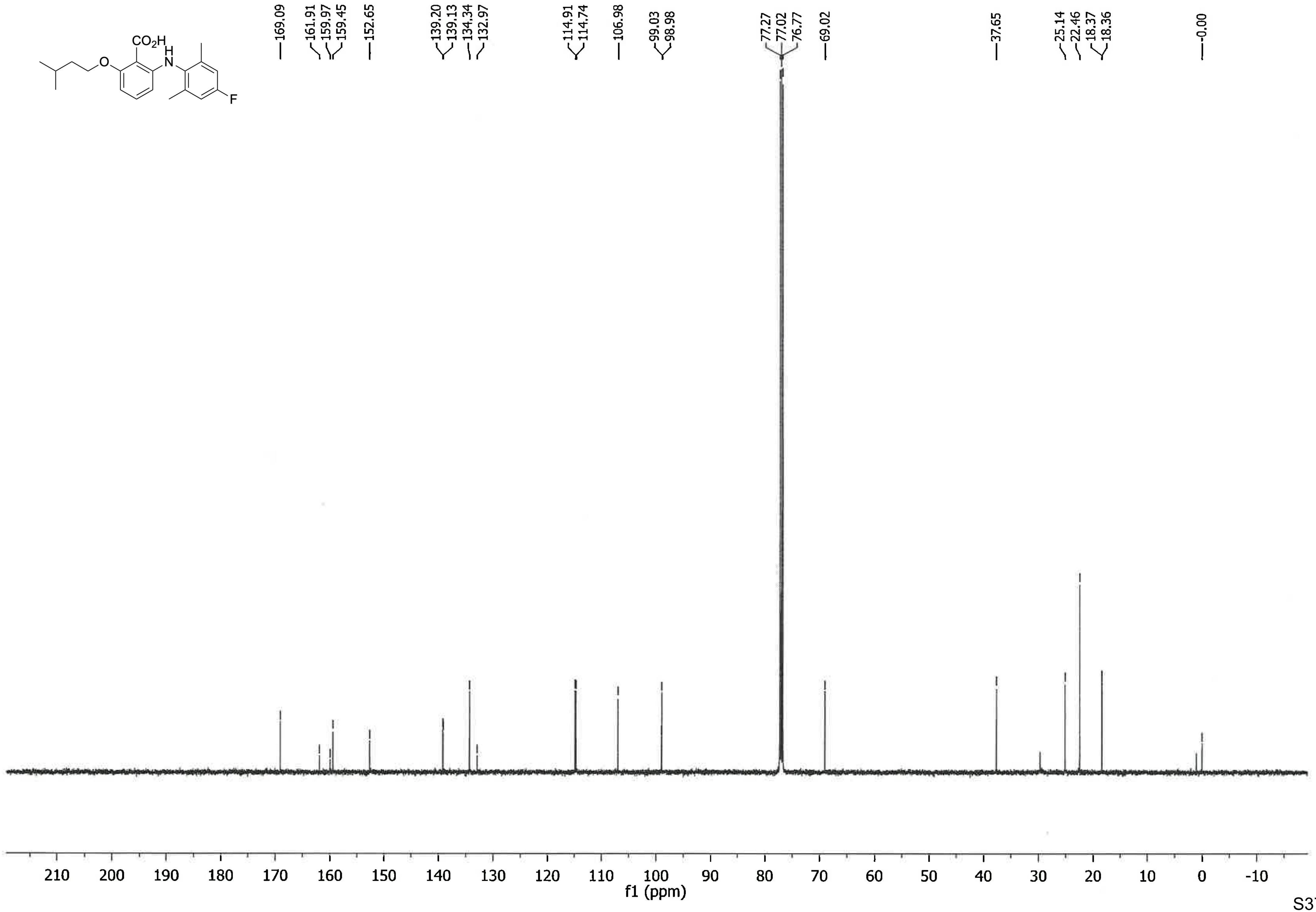

12f: 1H NMR (500 MHz, CDCl3)

Parameter	Value
Spectrometer Frequency	500.19

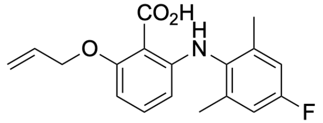

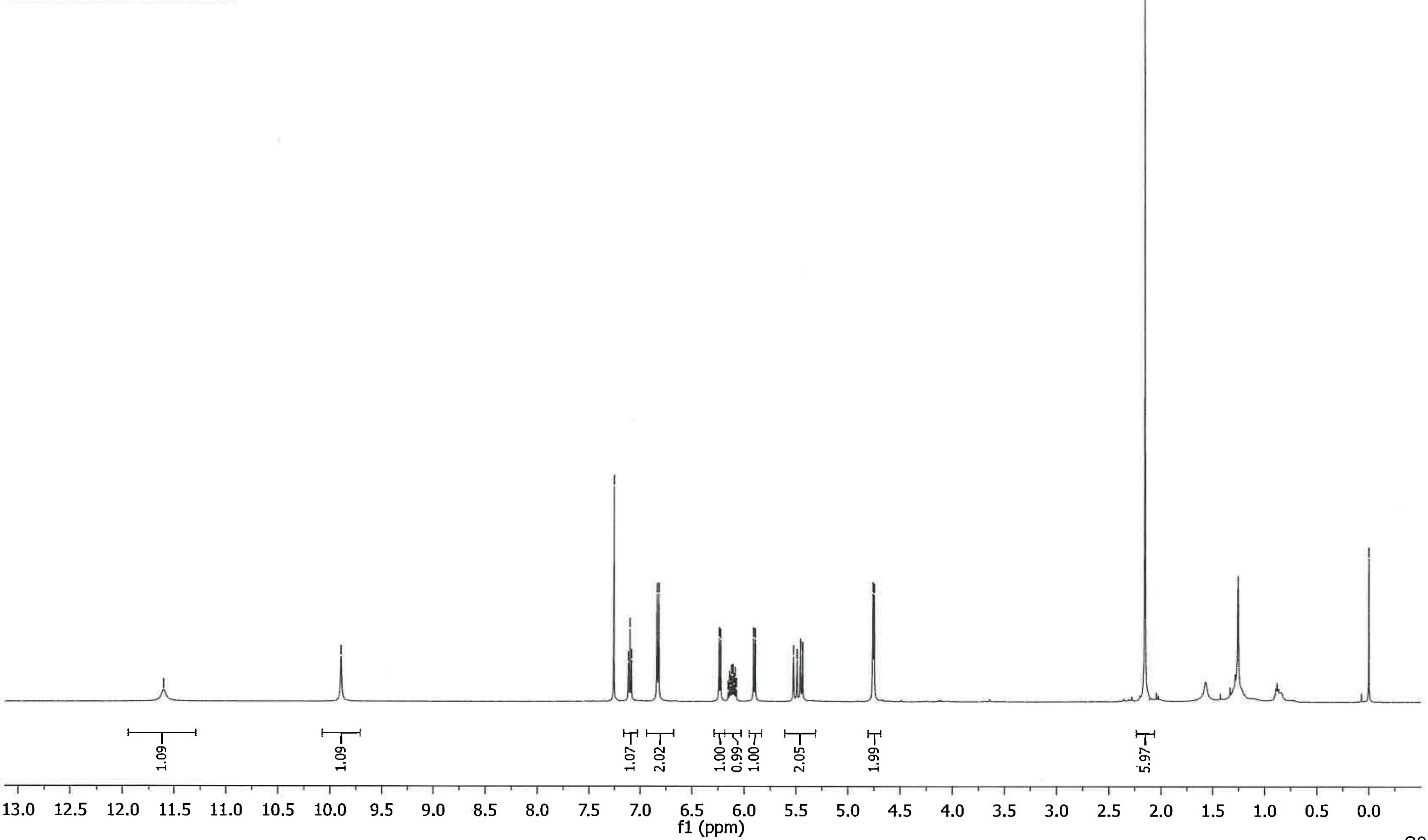

12f: 13C{1H} NMR (126 MHz, CDCl3)

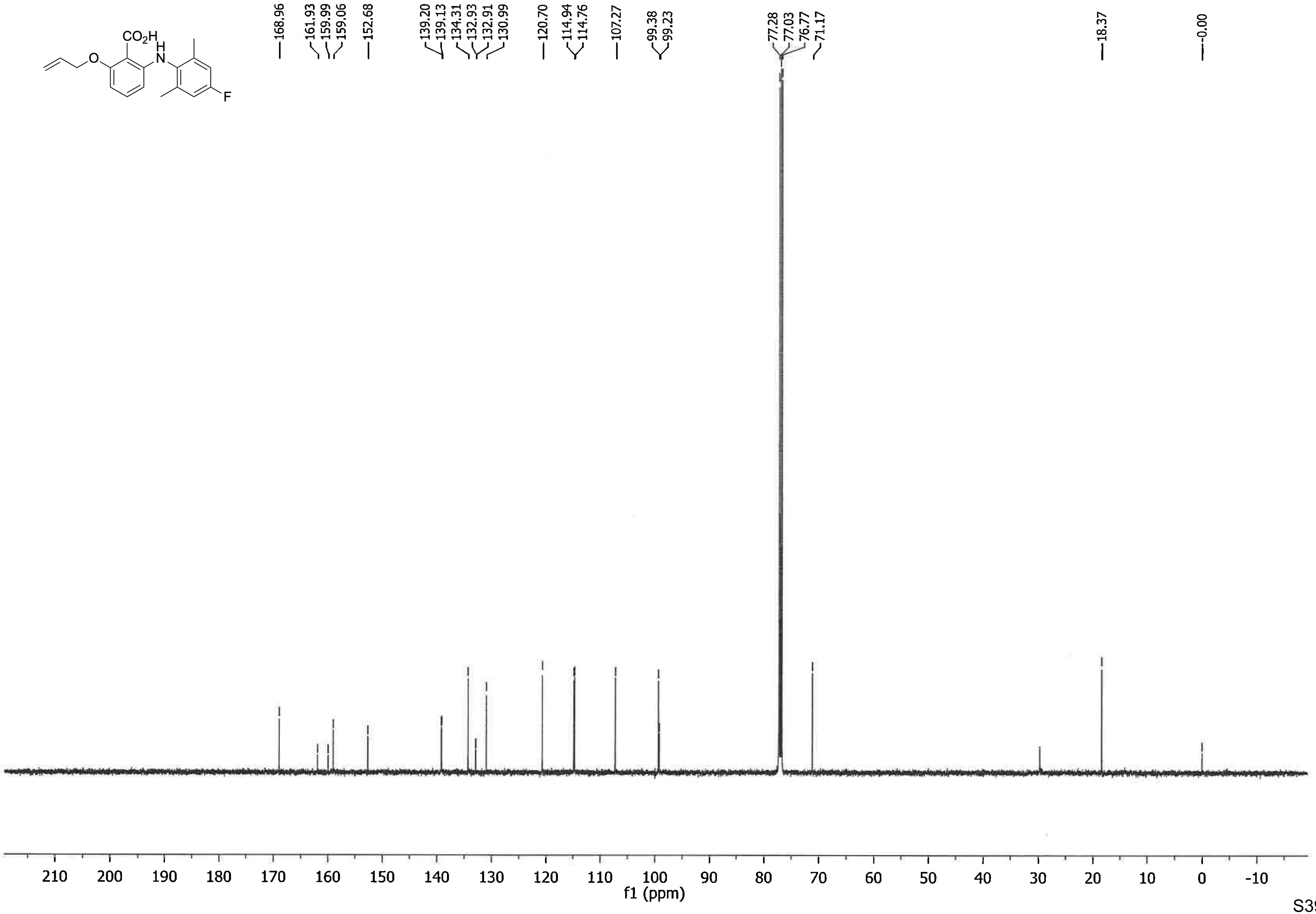

12g: 1H NMR (500 MHz, CDCl3)

Parameter	Value
Spectrometer Frequency	500.19

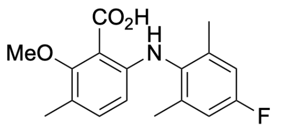

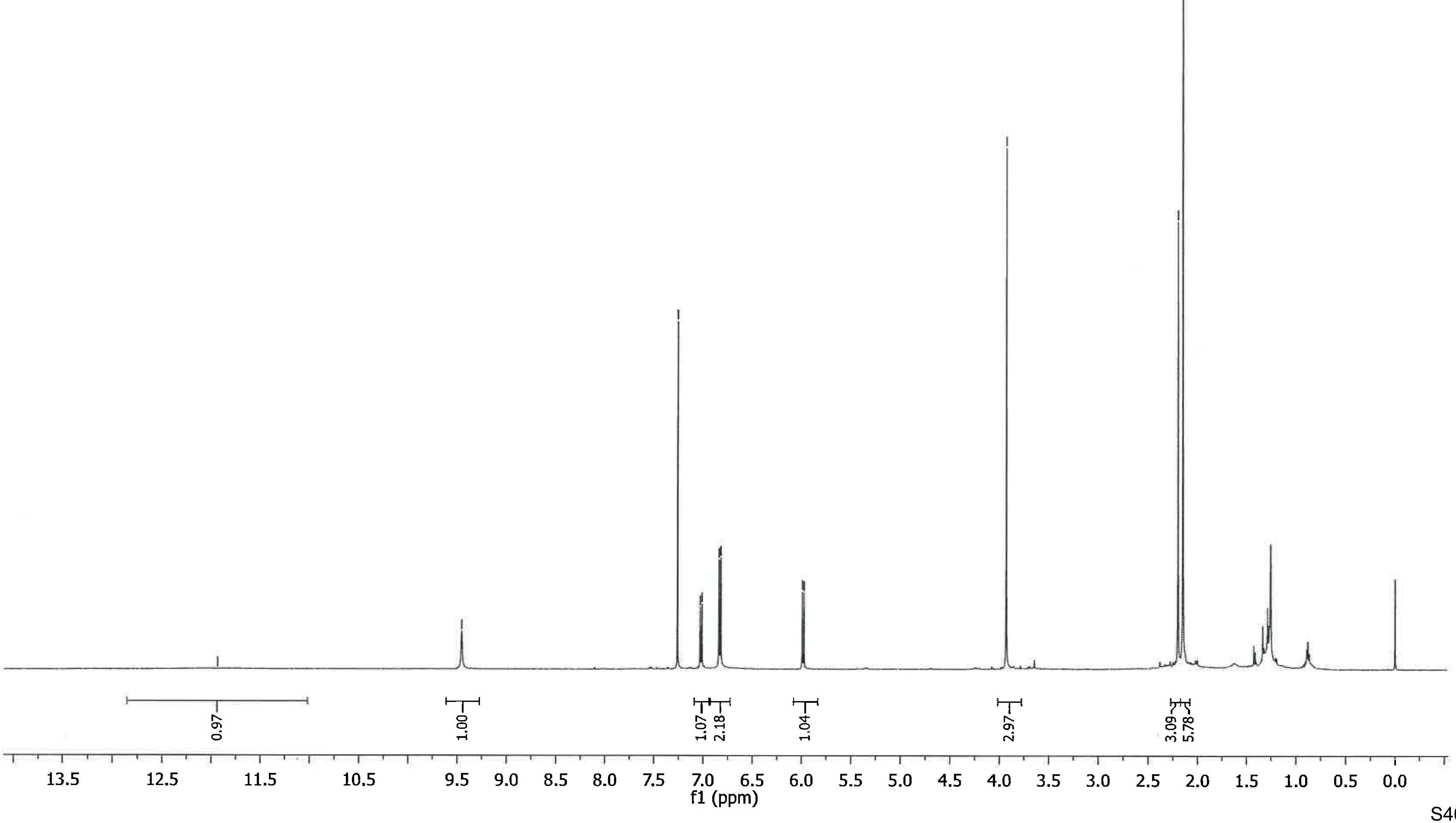

12g: 13C{1H} NMR (126 MHz, CDCl3)

Parameter	Value
Spectrometer Frequency	125.77

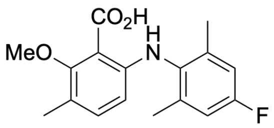

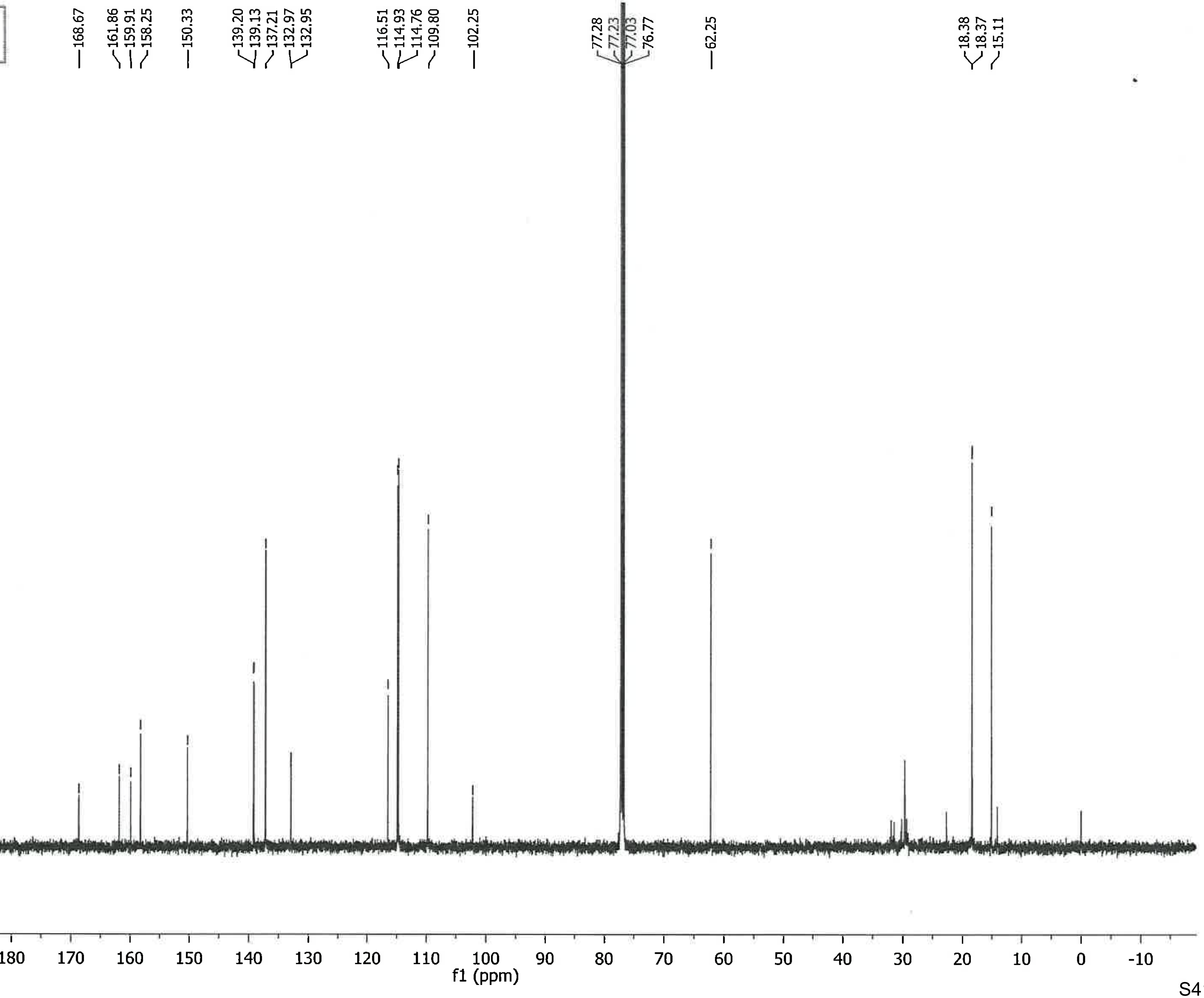

12h: 1H NMR (400 MHz, CDCl3)

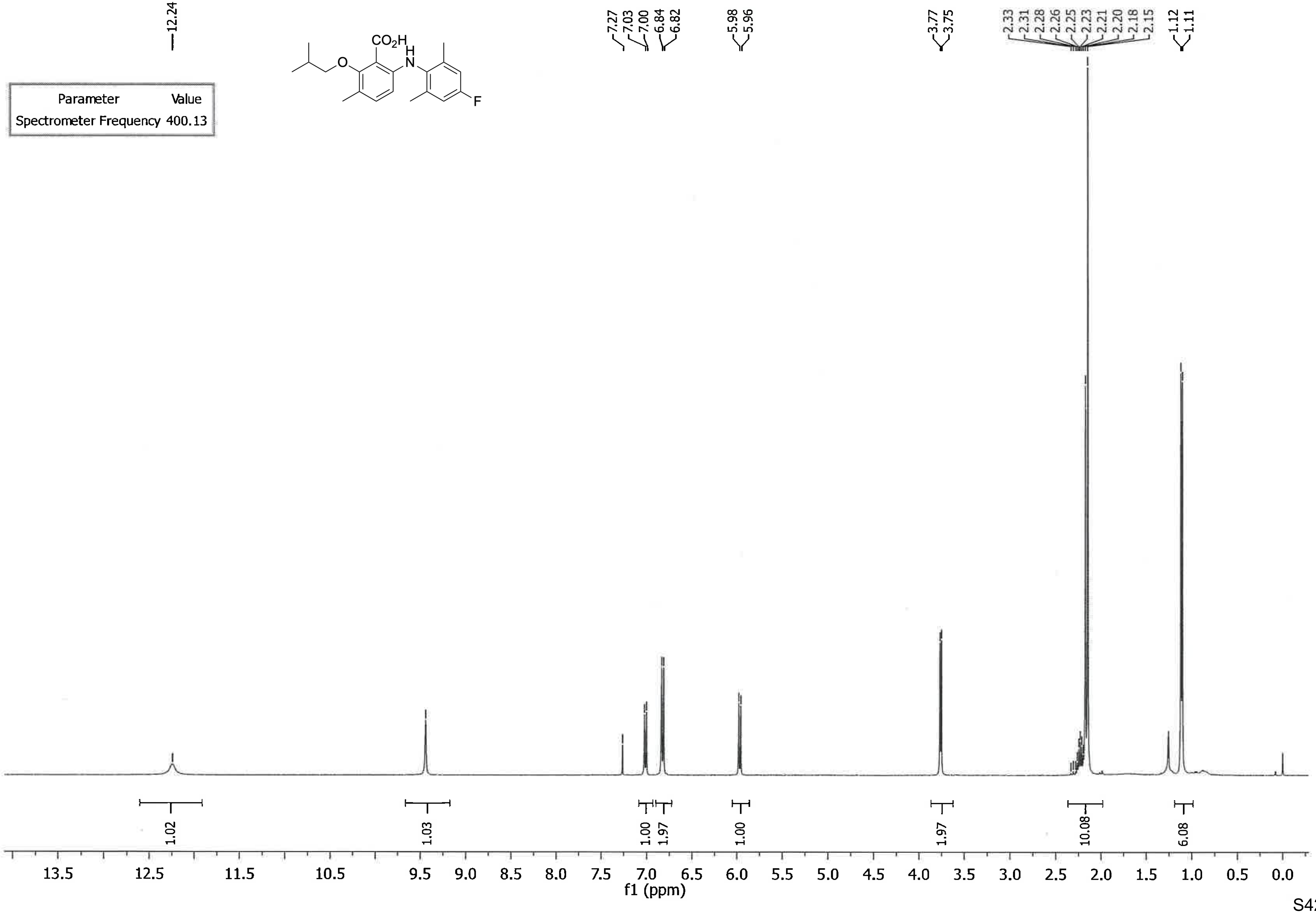

12h: 13C{1H} NMR (101 MHz, CDCl3)

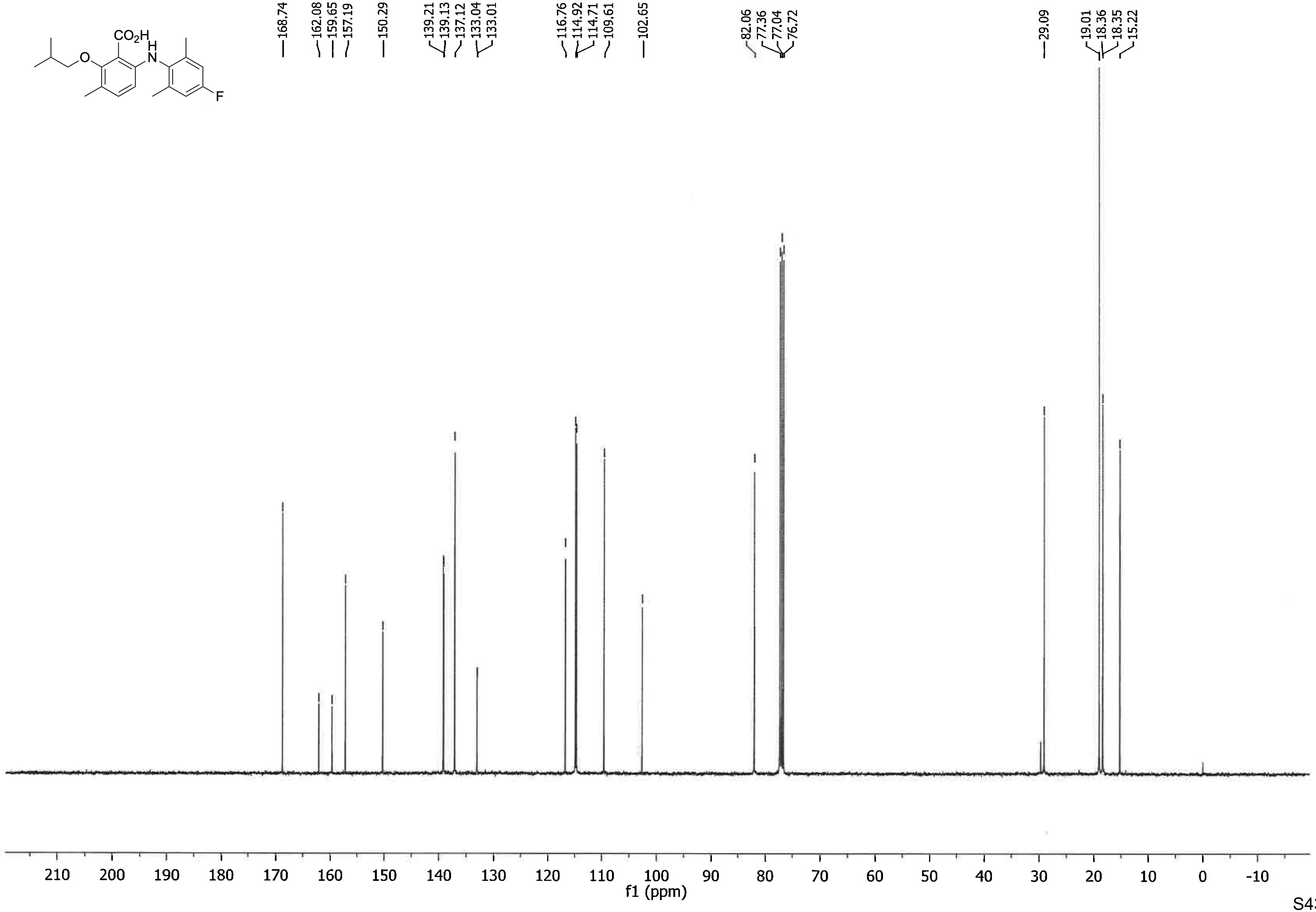

7a: 1H NMR (400 MHz, CDCl3)

Parameter	Value
Spectrometer Frequency	400.19

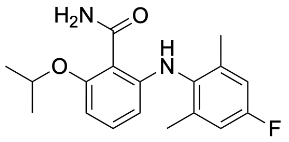

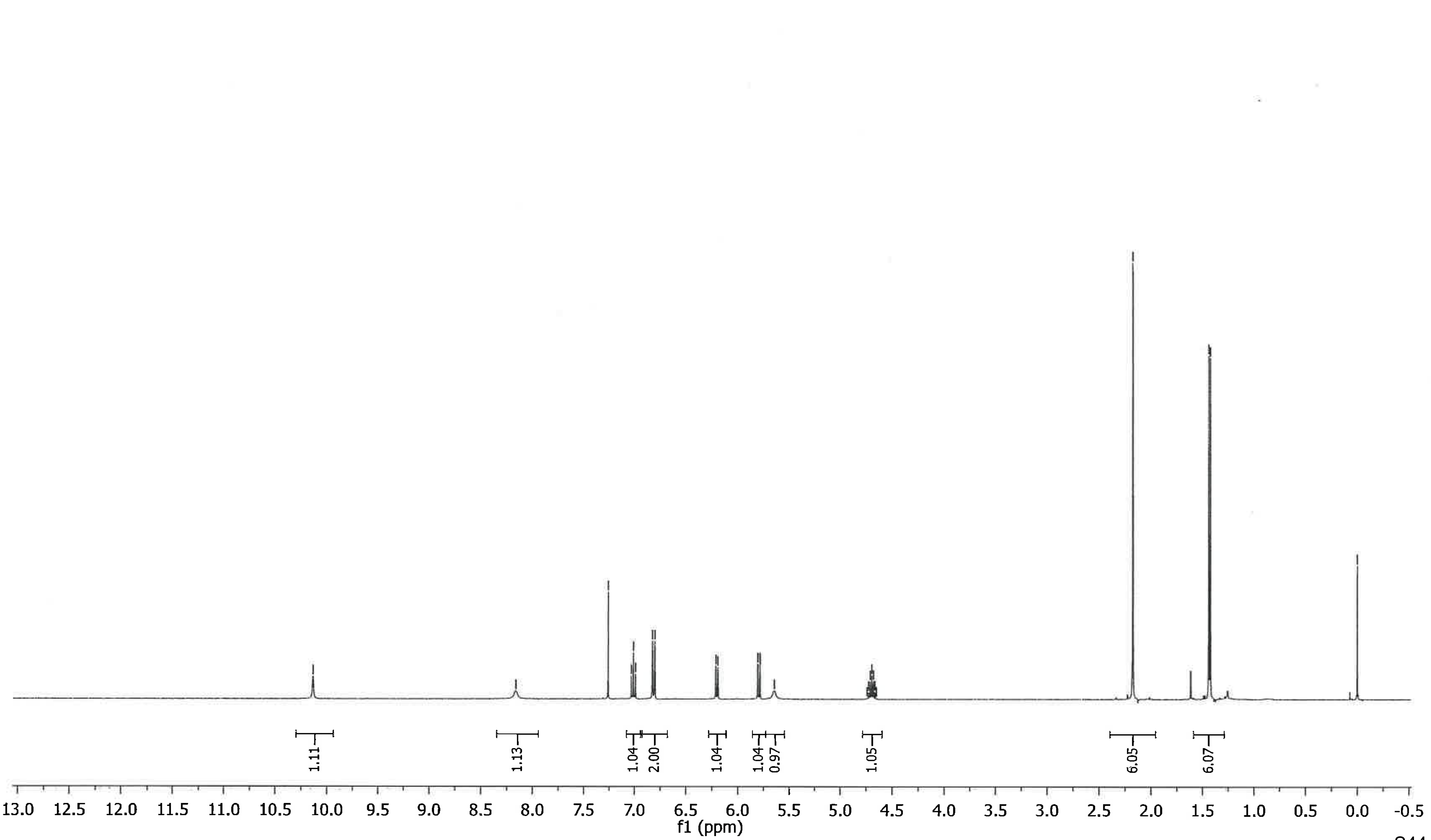

7a: 13C{1H} NMR (126 MHz, CDCl3)

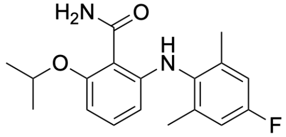

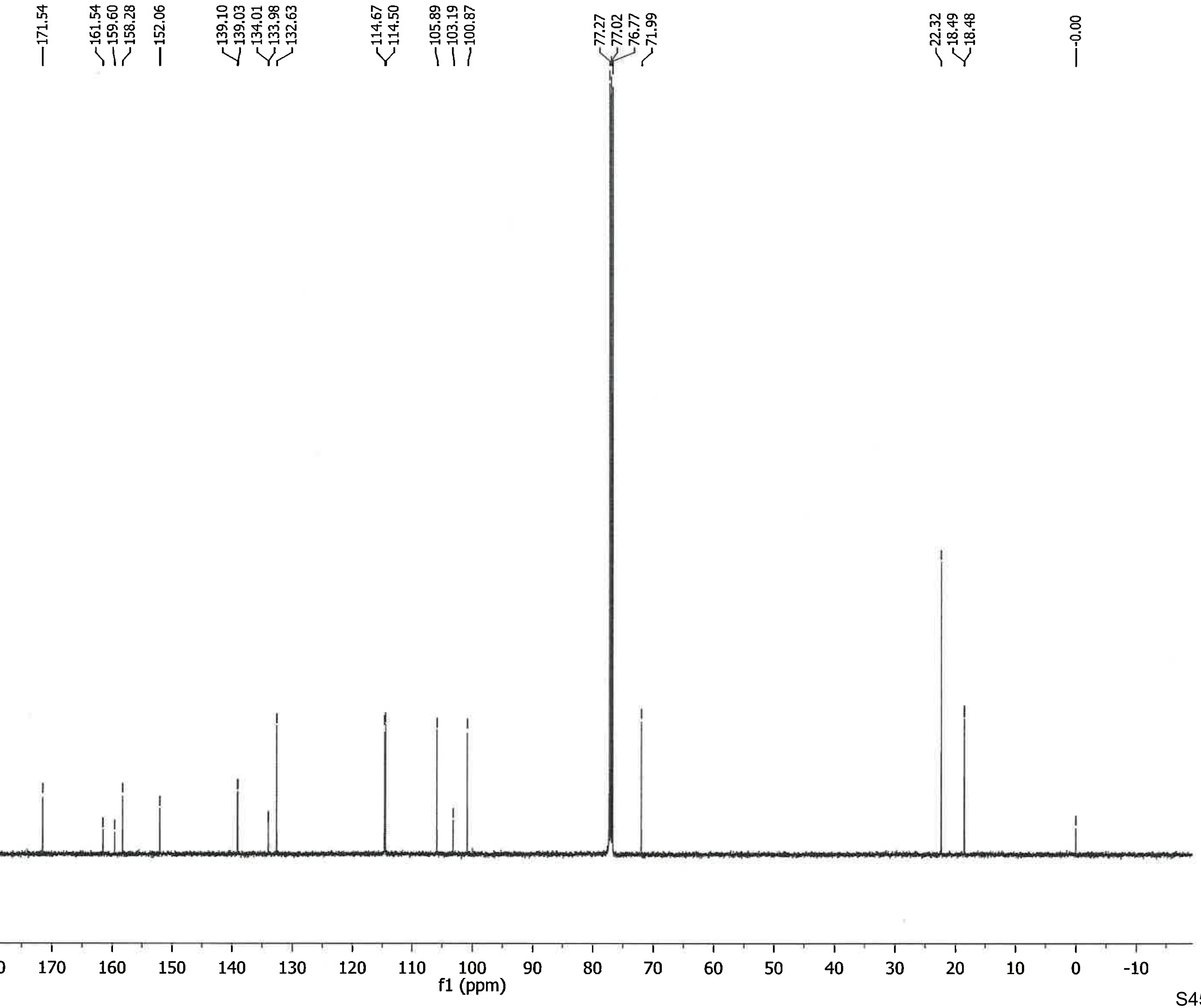

7b: 1H NMR (400 MHz, CDCl3)

Parameter	Value
Spectrometer Frequency	400.19

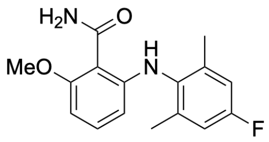

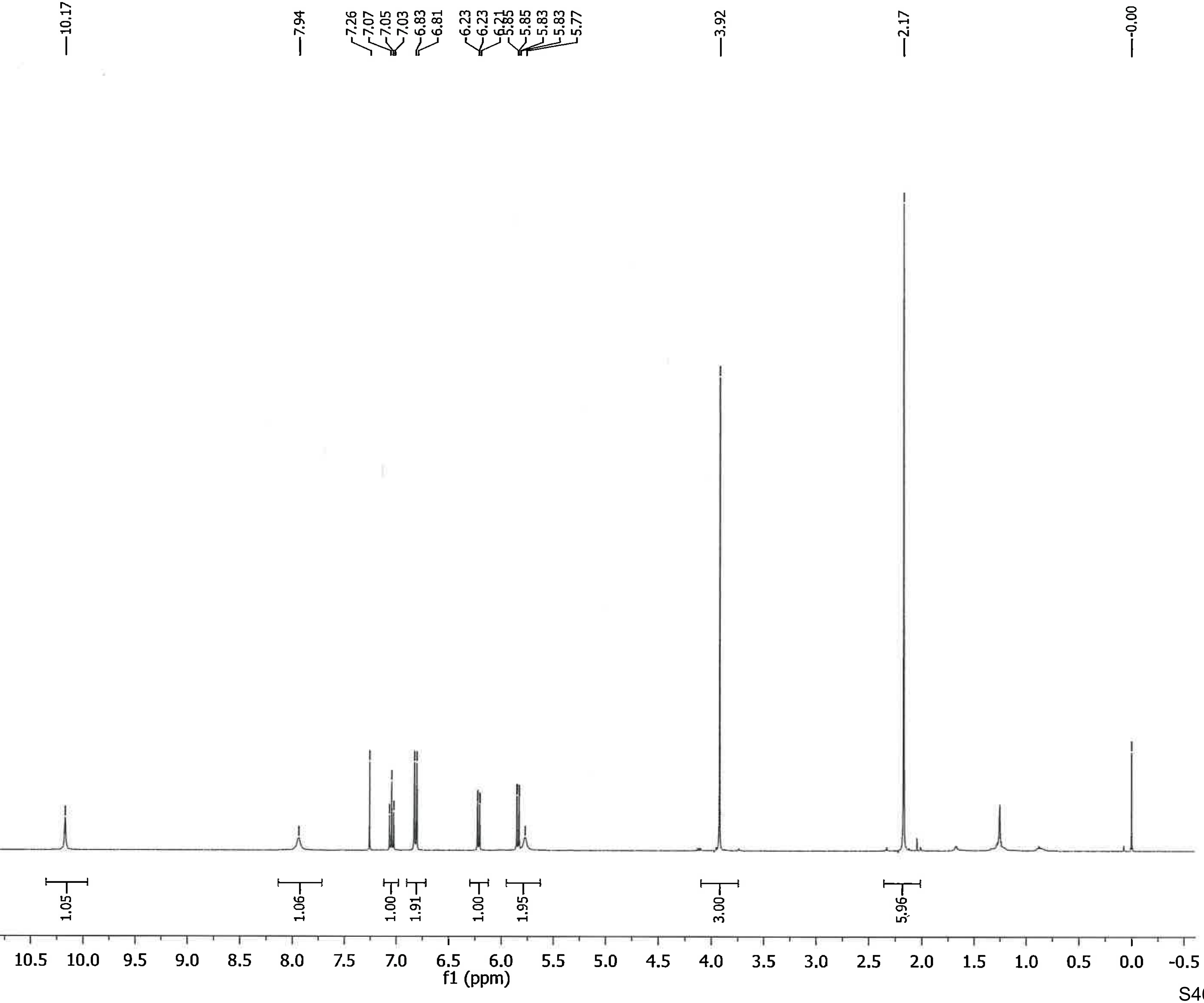

7b: 13C{1H} NMR (101 MHz, CDCl3)

Parameter	Value
Spectrometer Frequency	100.63

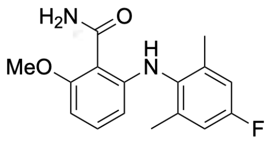

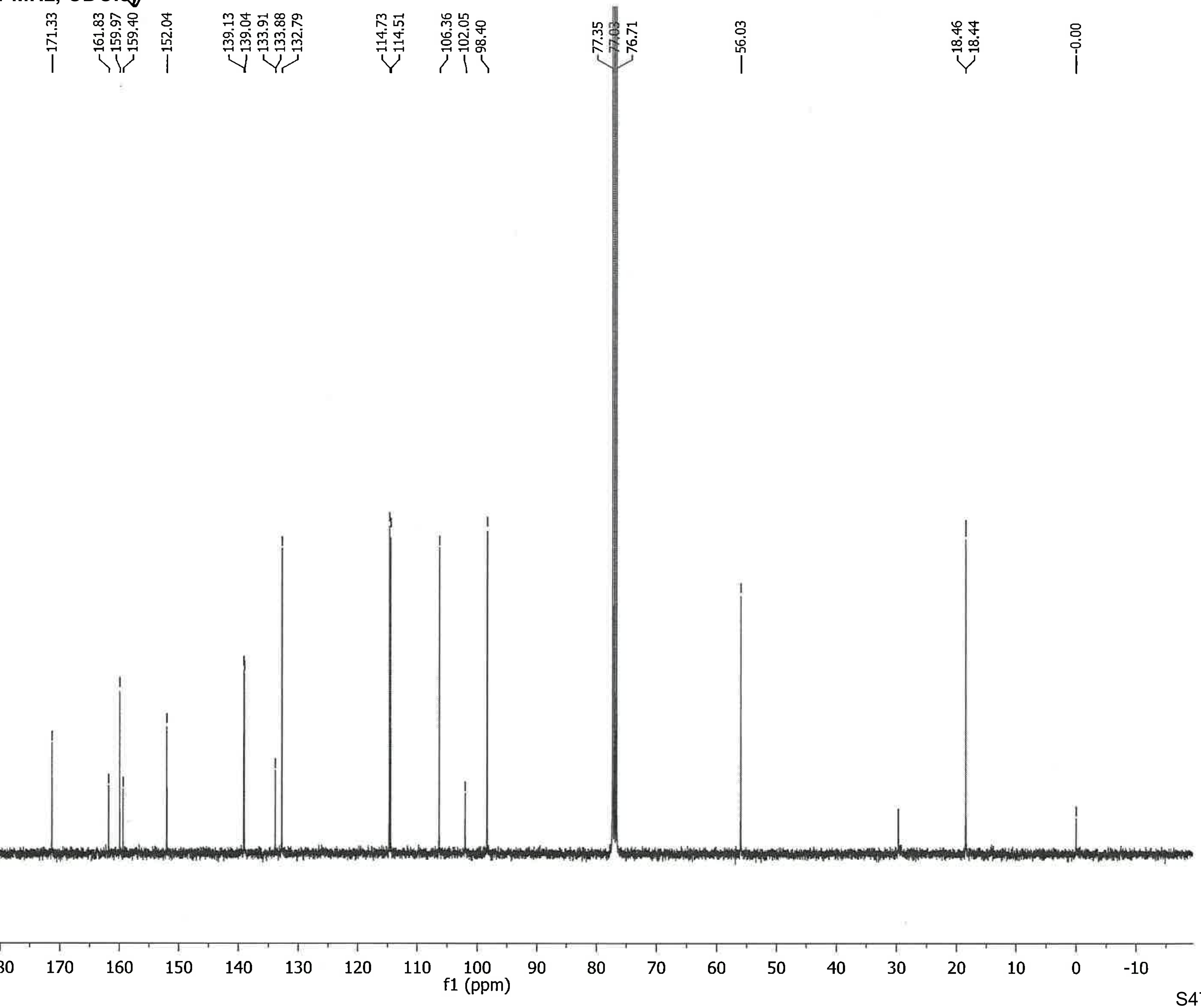

7c: 1H NMR (500 MHz, CDCl3)

Parameter	Value
Spectrometer Frequency	500.19

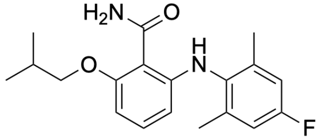

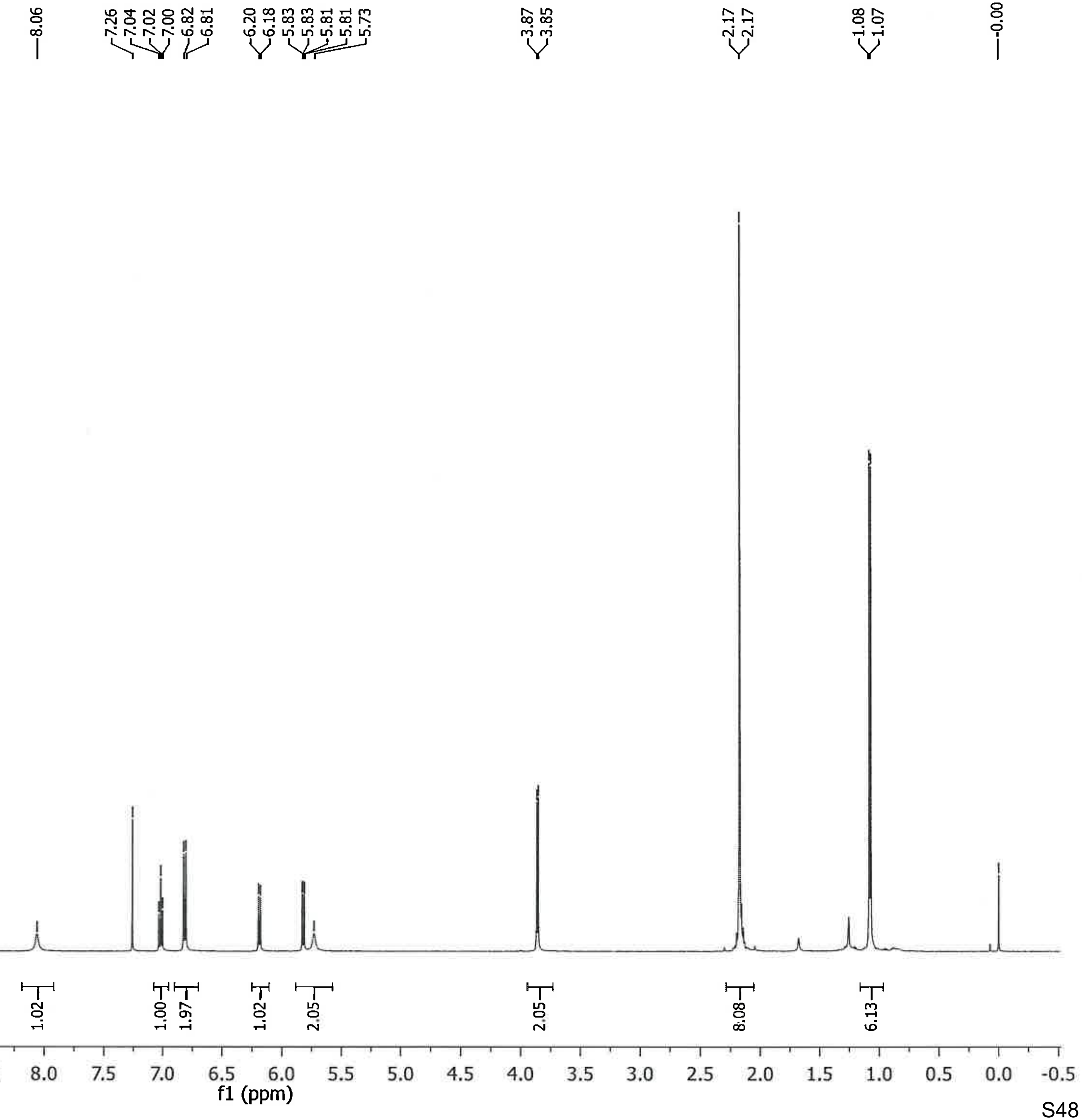

7c: 13C{1H} NMR (126 MHz, CDCl3)

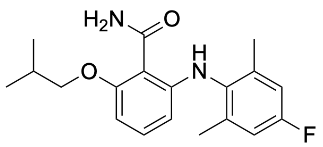

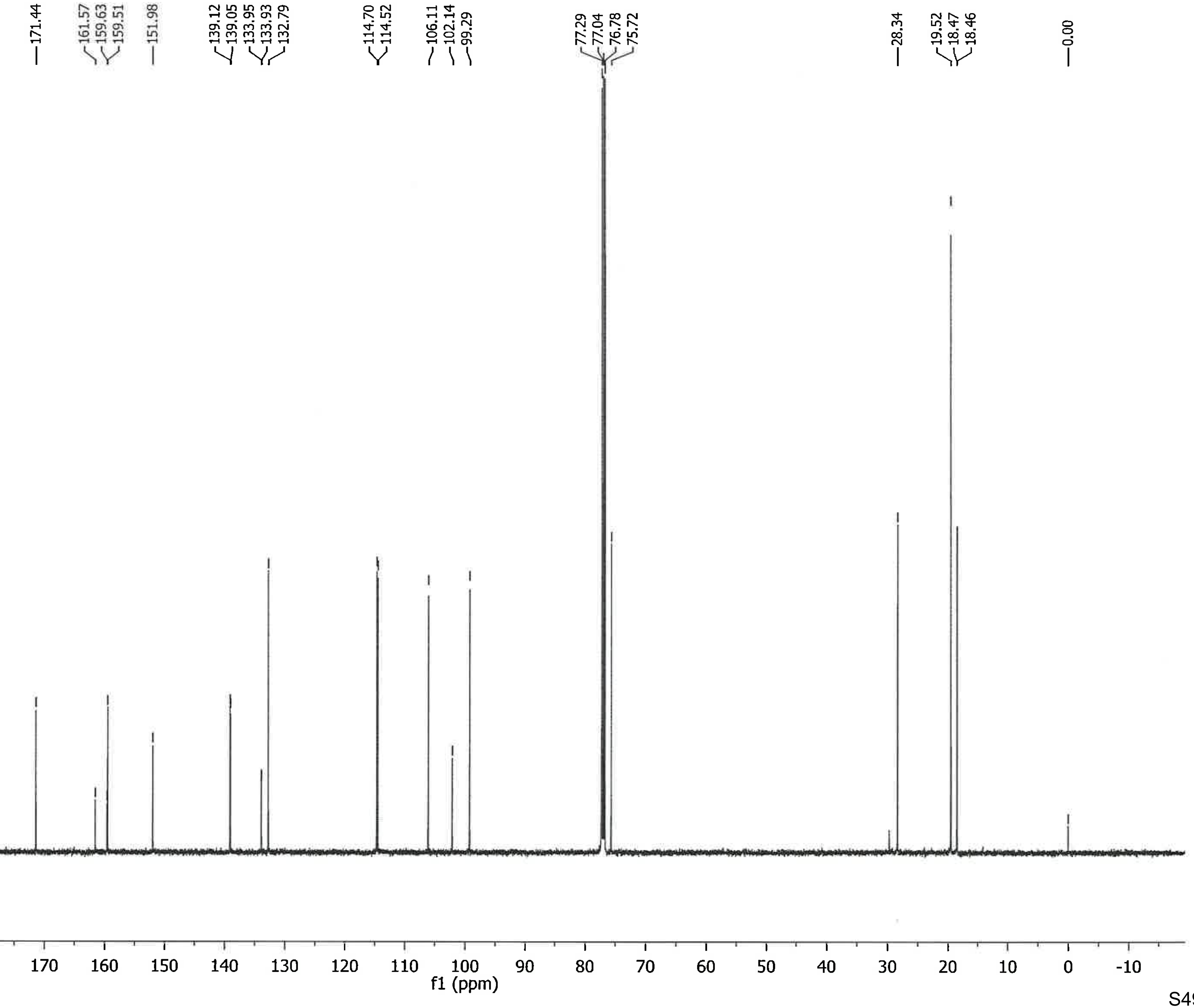

7d: 1H NMR (500 MHz, CDCl3)

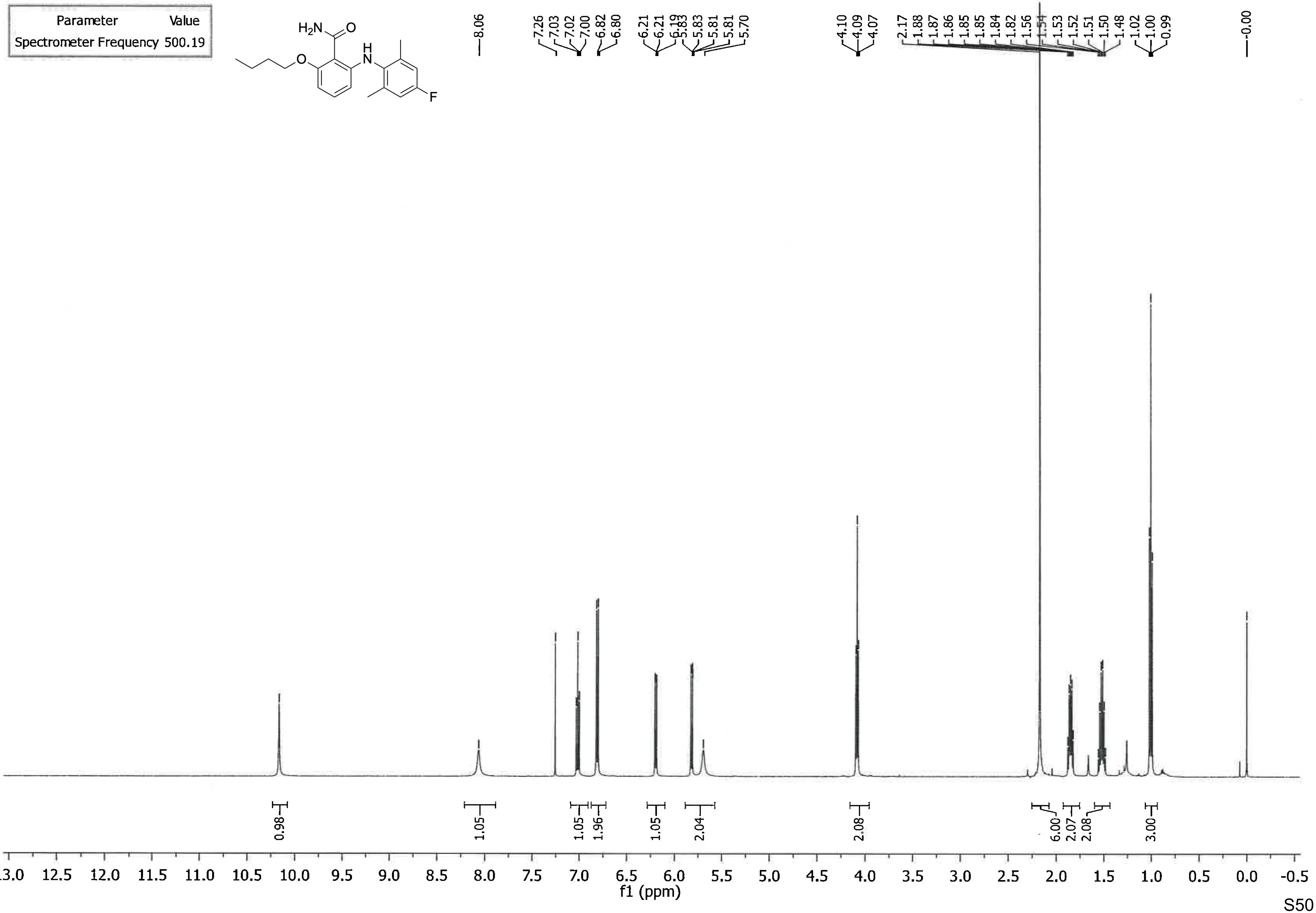

7d: 13C{1H} NMR (126 MHz, CDCl3)

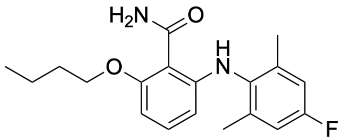

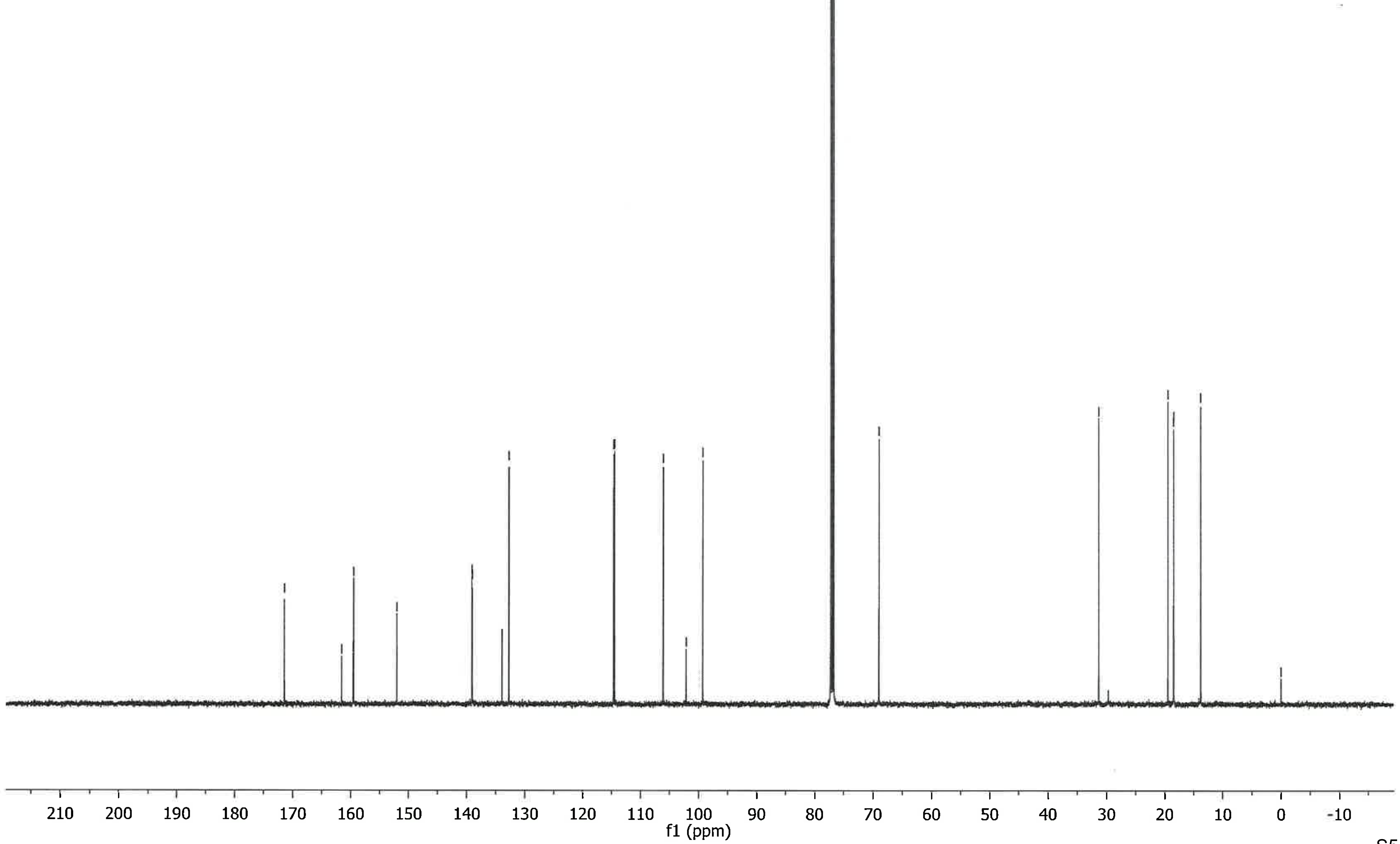

7e: 1H NMR (500 MHz, CDCl3)

Parameter	Value
Spectrometer Frequency	500.19

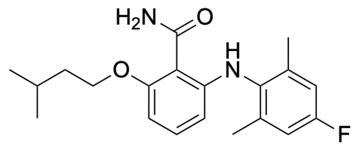

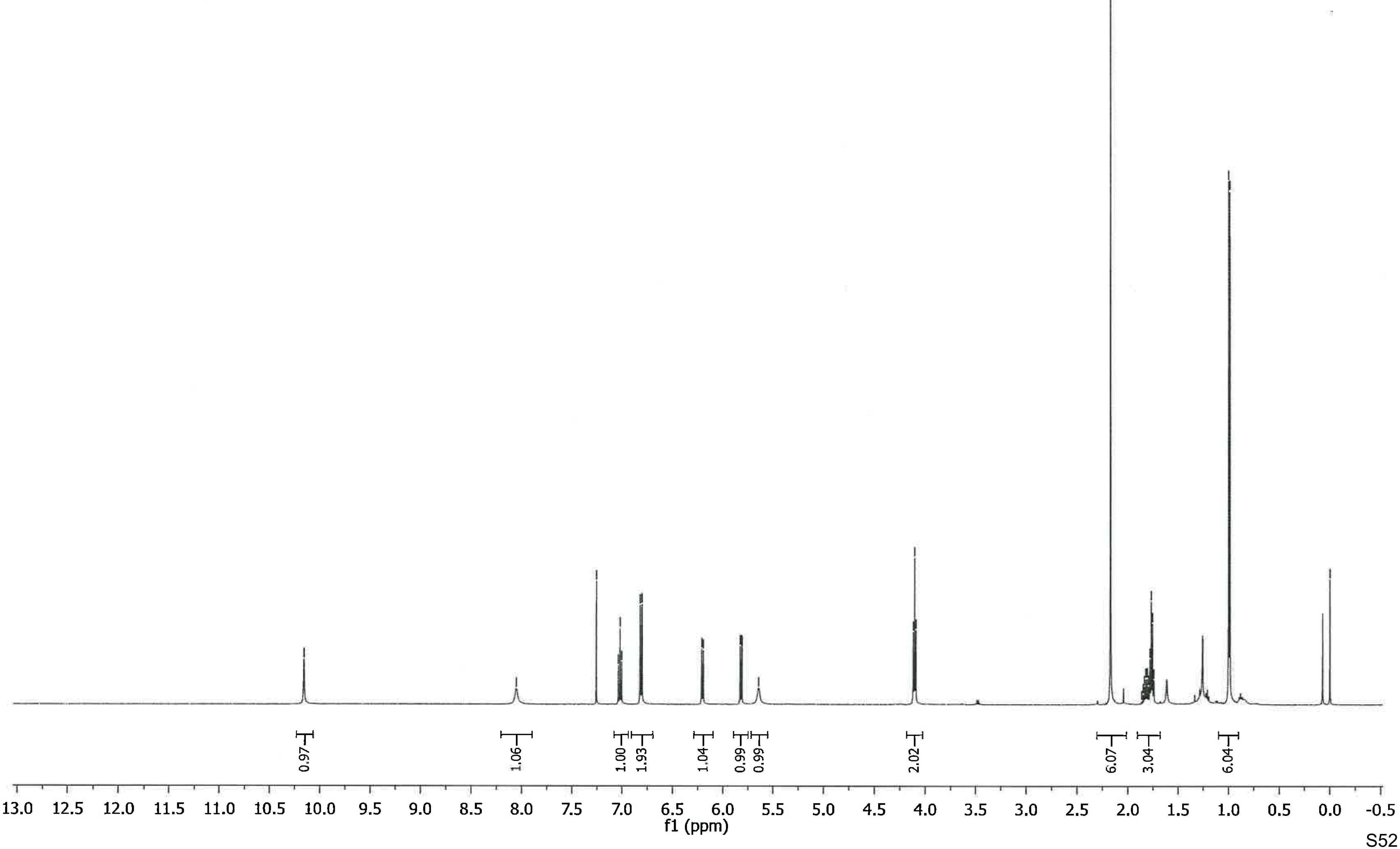

7e: 13C{1H} NMR (126 MHz, CDCl3)

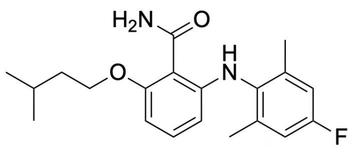

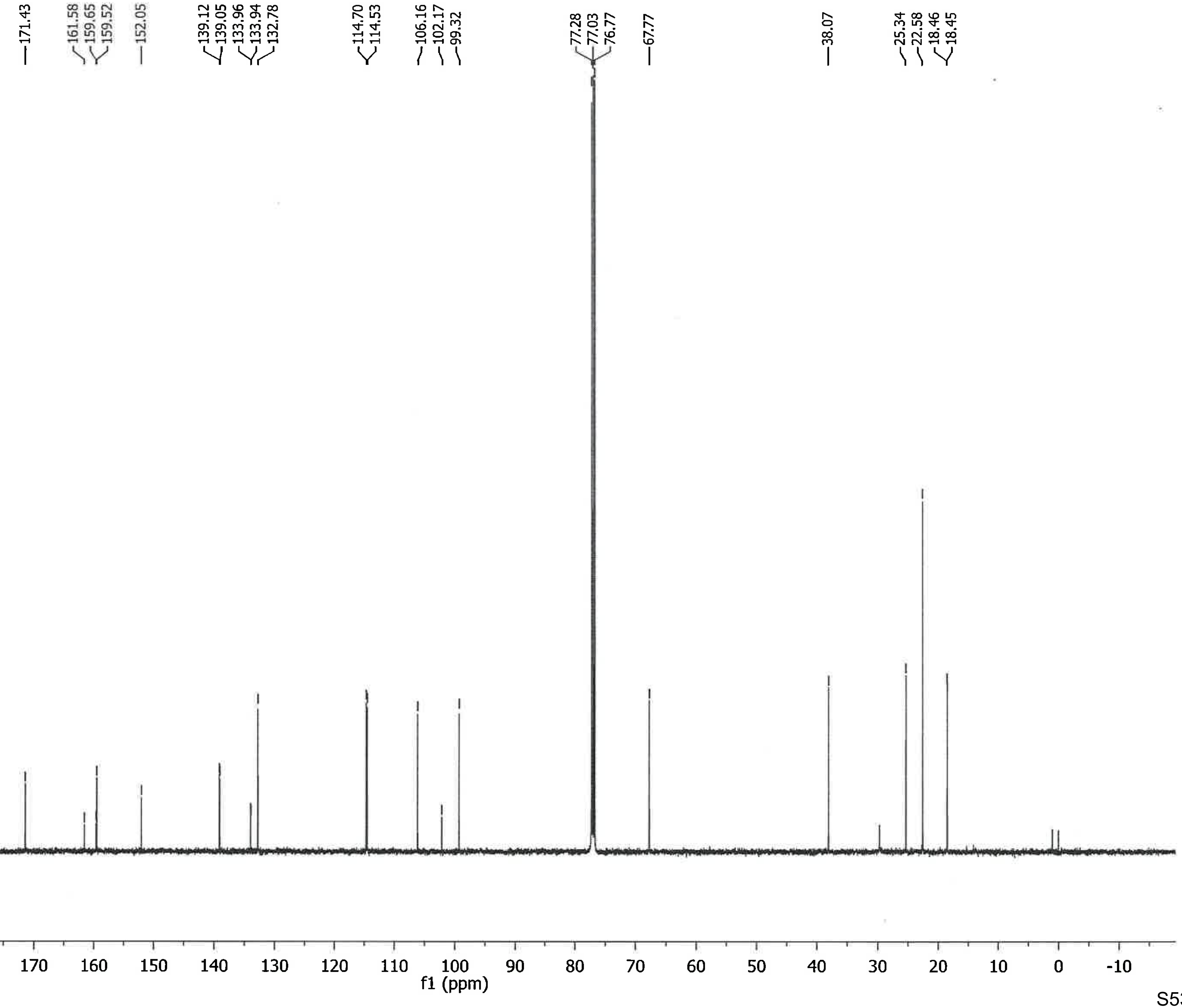

7f: 1H NMR (500 MHz, CDCl3)

Parameter	Value
Spectrometer Frequency	500.19

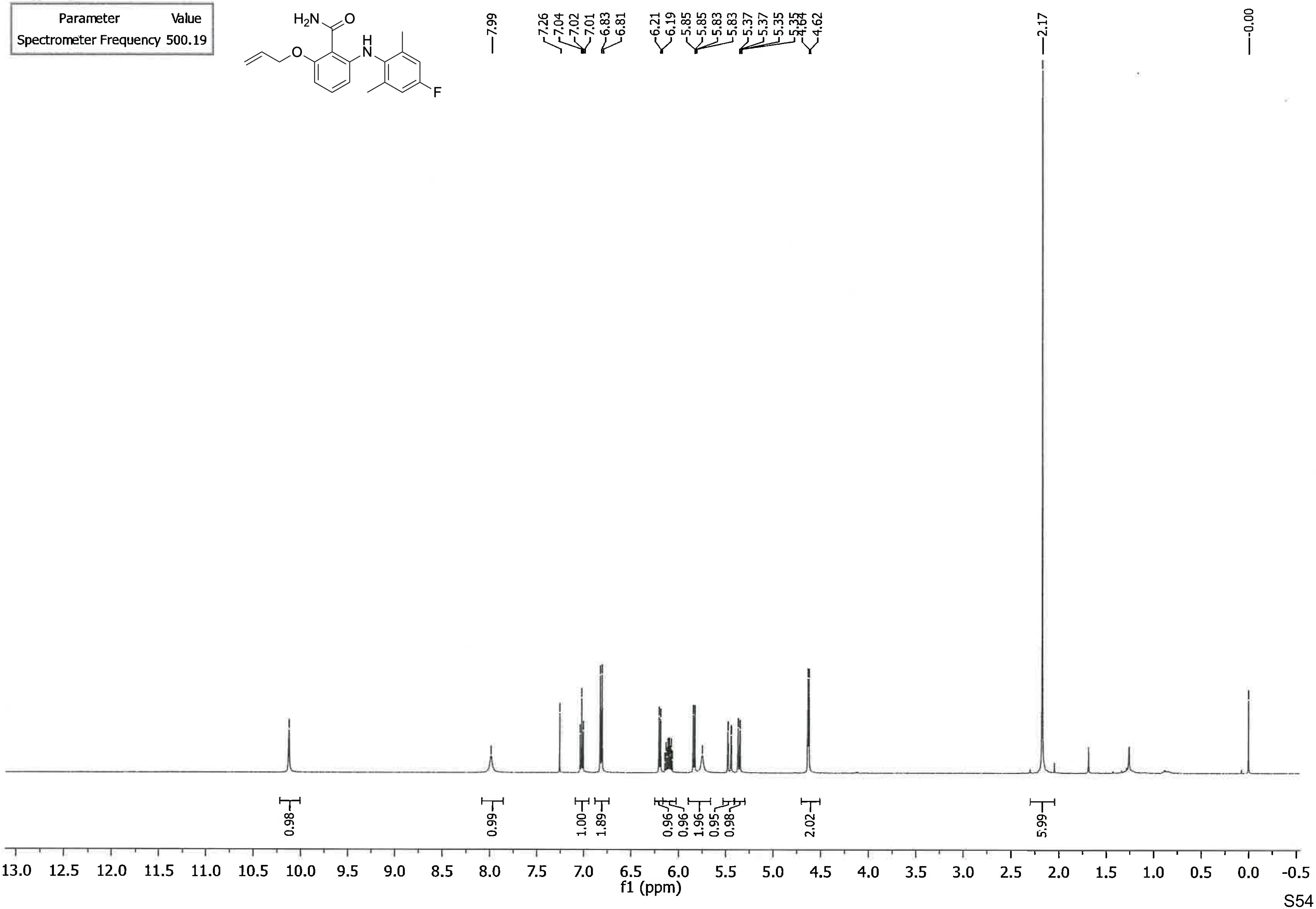

7f: 13C{1H} NMR (126 MHz, CDCl3)

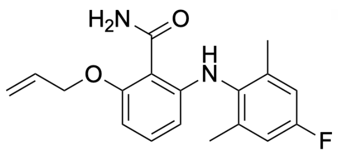

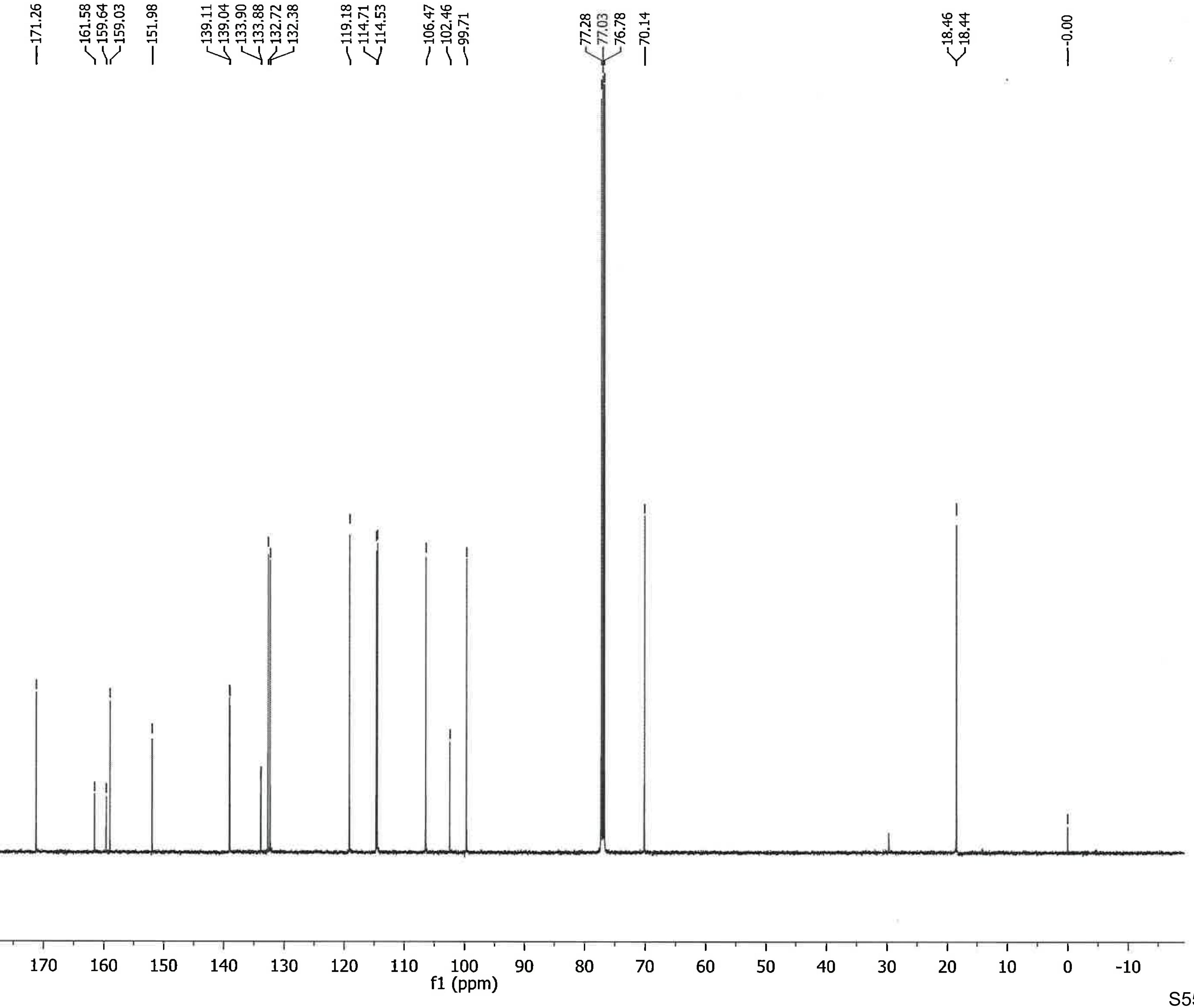

7g: 1H NMR (500 MHz, CDCl3)

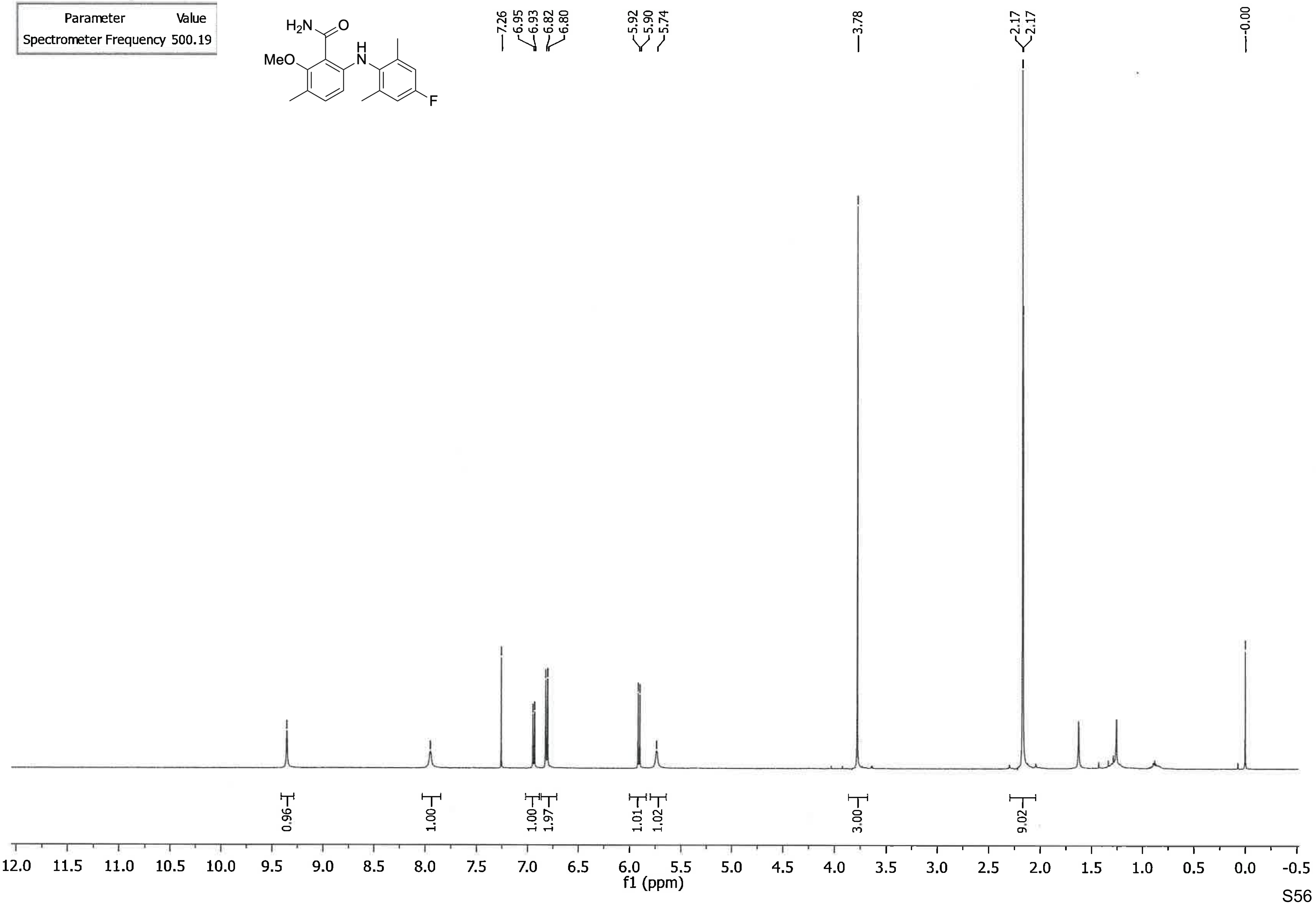

7g: 13C{1H} NMR (126 MHz, CDCl3)

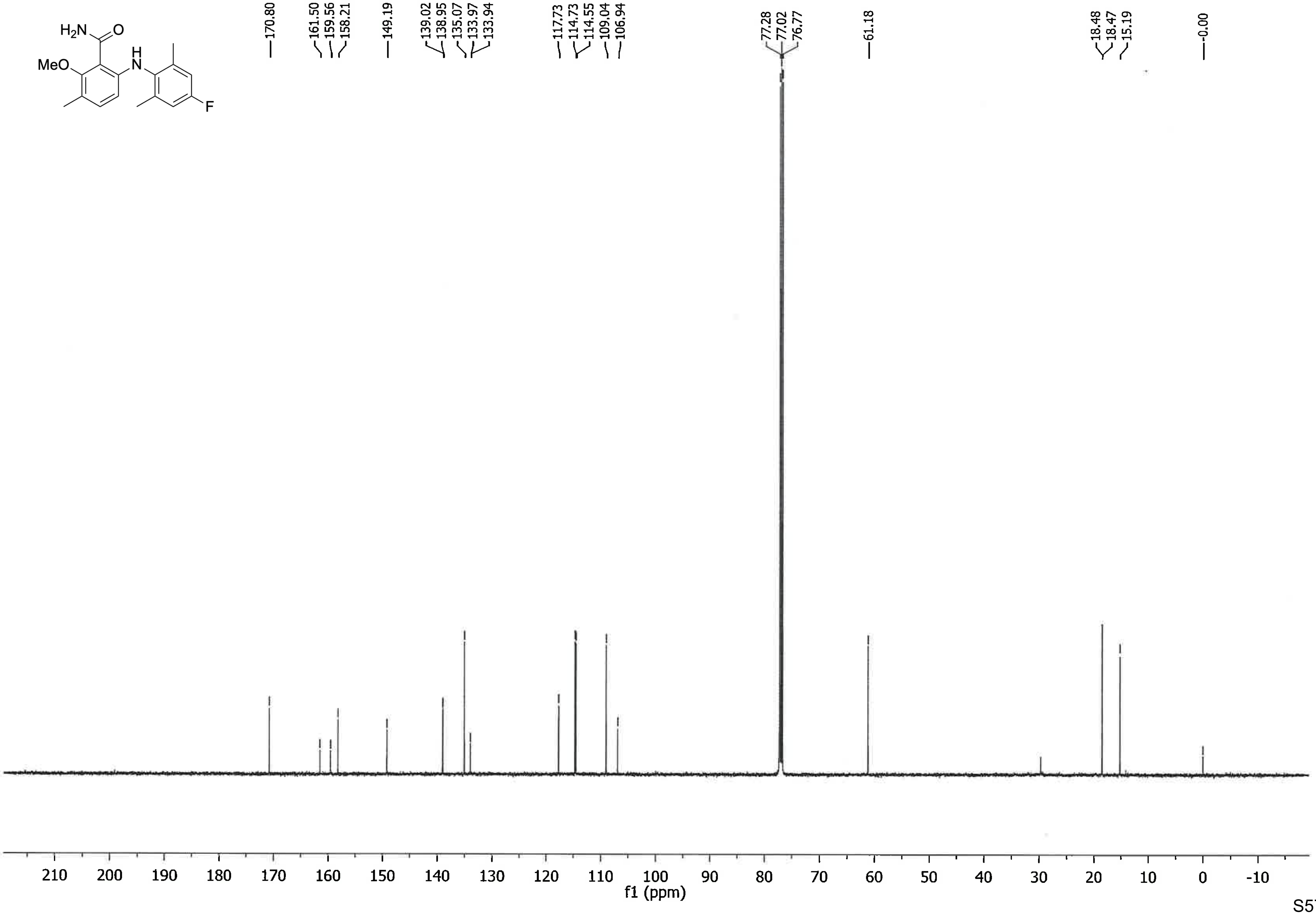

7h: 1H NMR (400 MHz, CDCl3)

Parameter	Value
Spectrometer Frequency	400.13

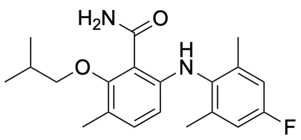

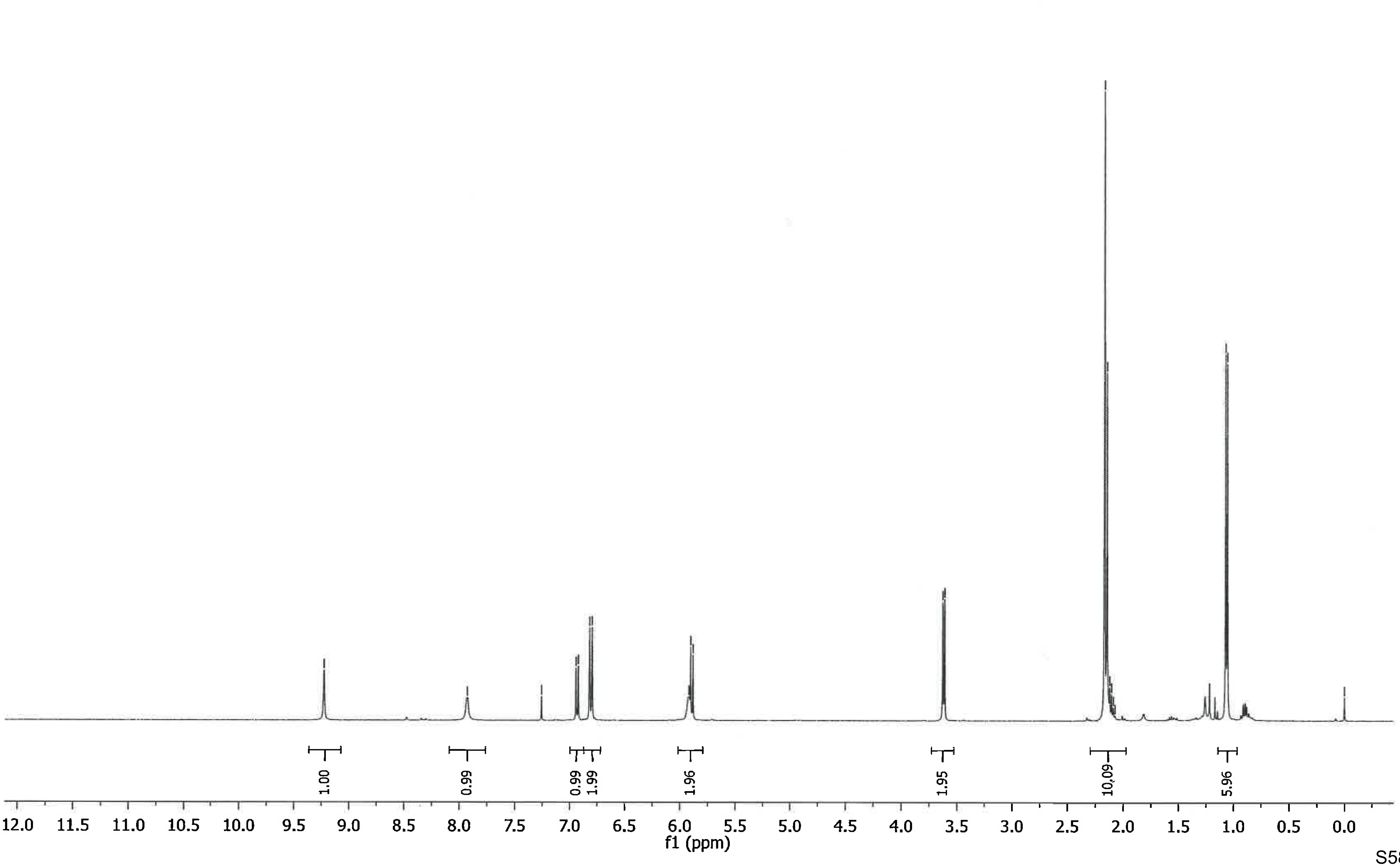

7h: 13C{1H} NMR (101 MHz, CDCl3)

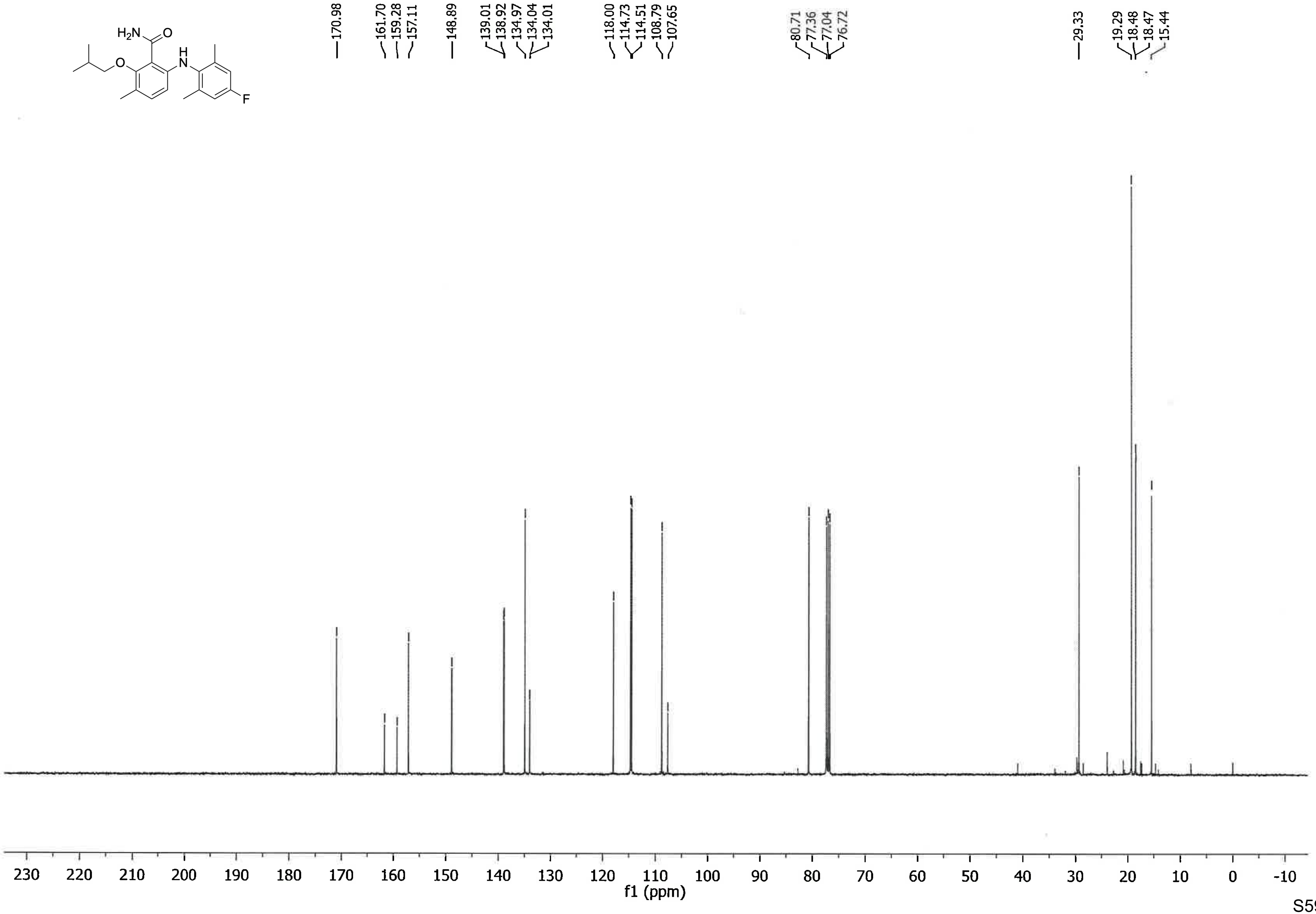

14: 1H NMR (400 MHz, CDCl3)

Parameter	Value
Spectrometer Frequency	400.19

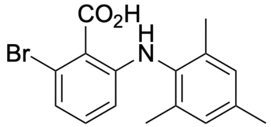

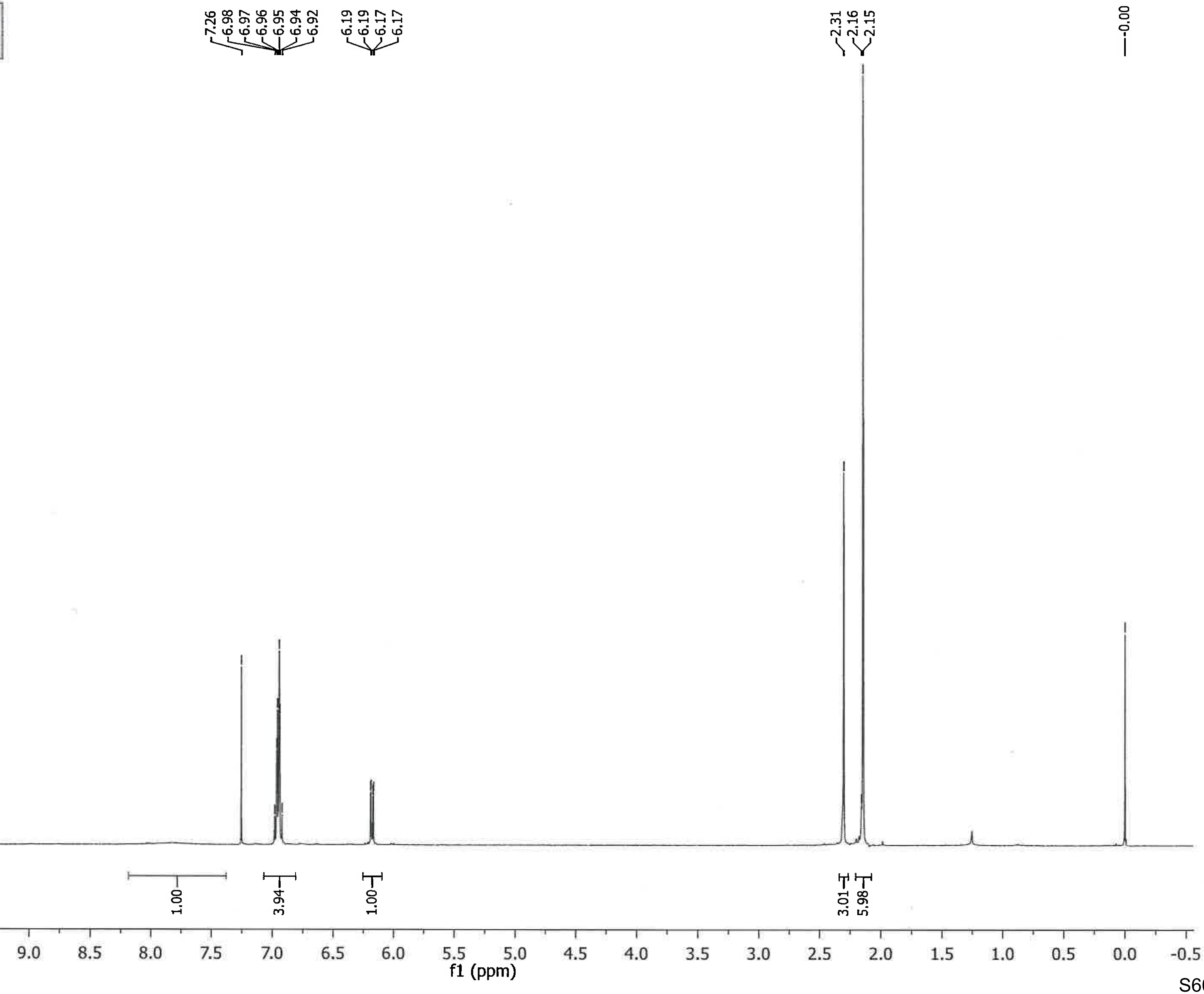

14: 13C{1H} NMR (101 MHz, CDCl3)

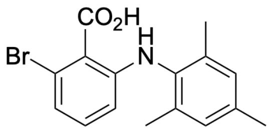

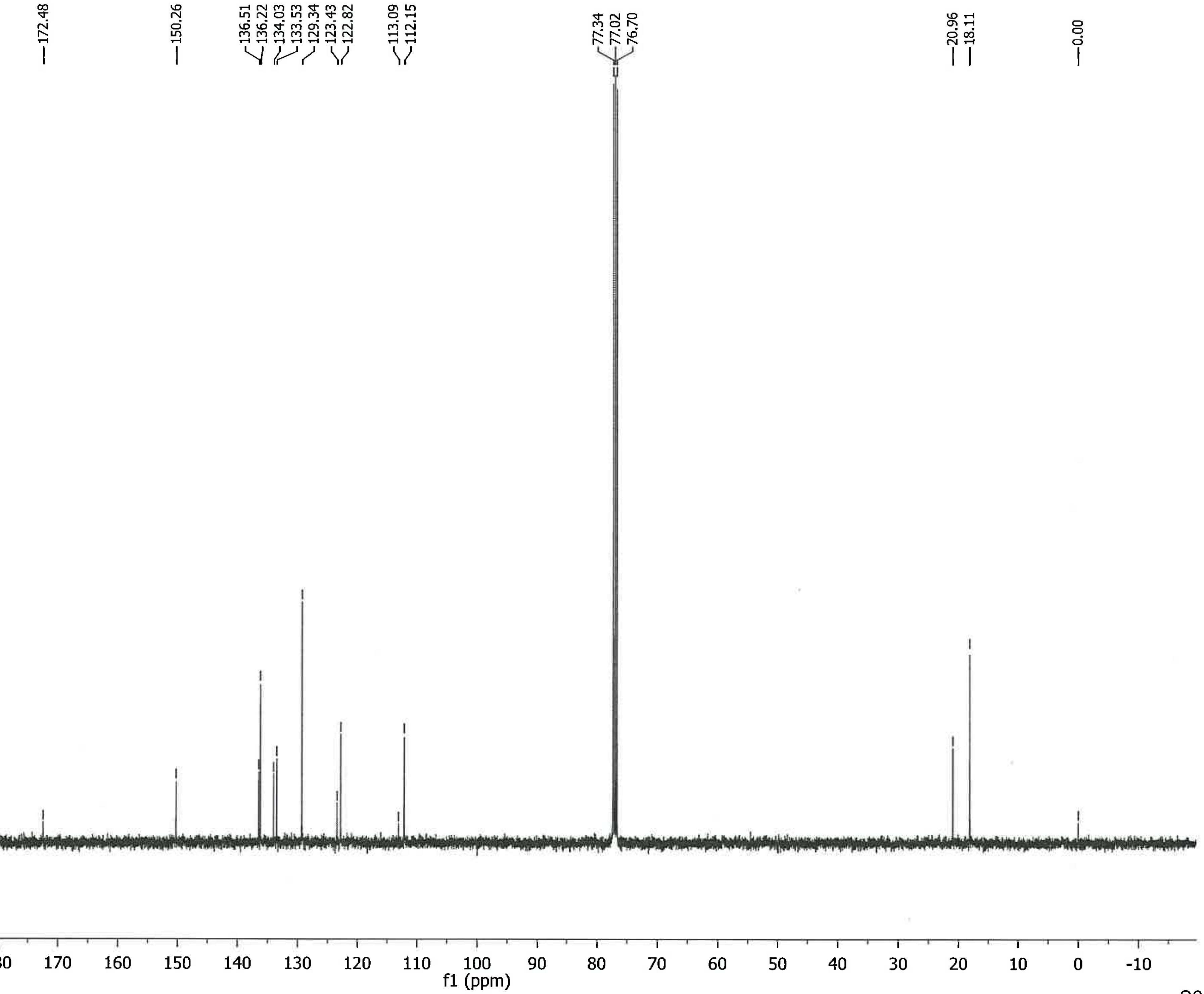

15: 1H NMR (500 MHz, CDCl3)

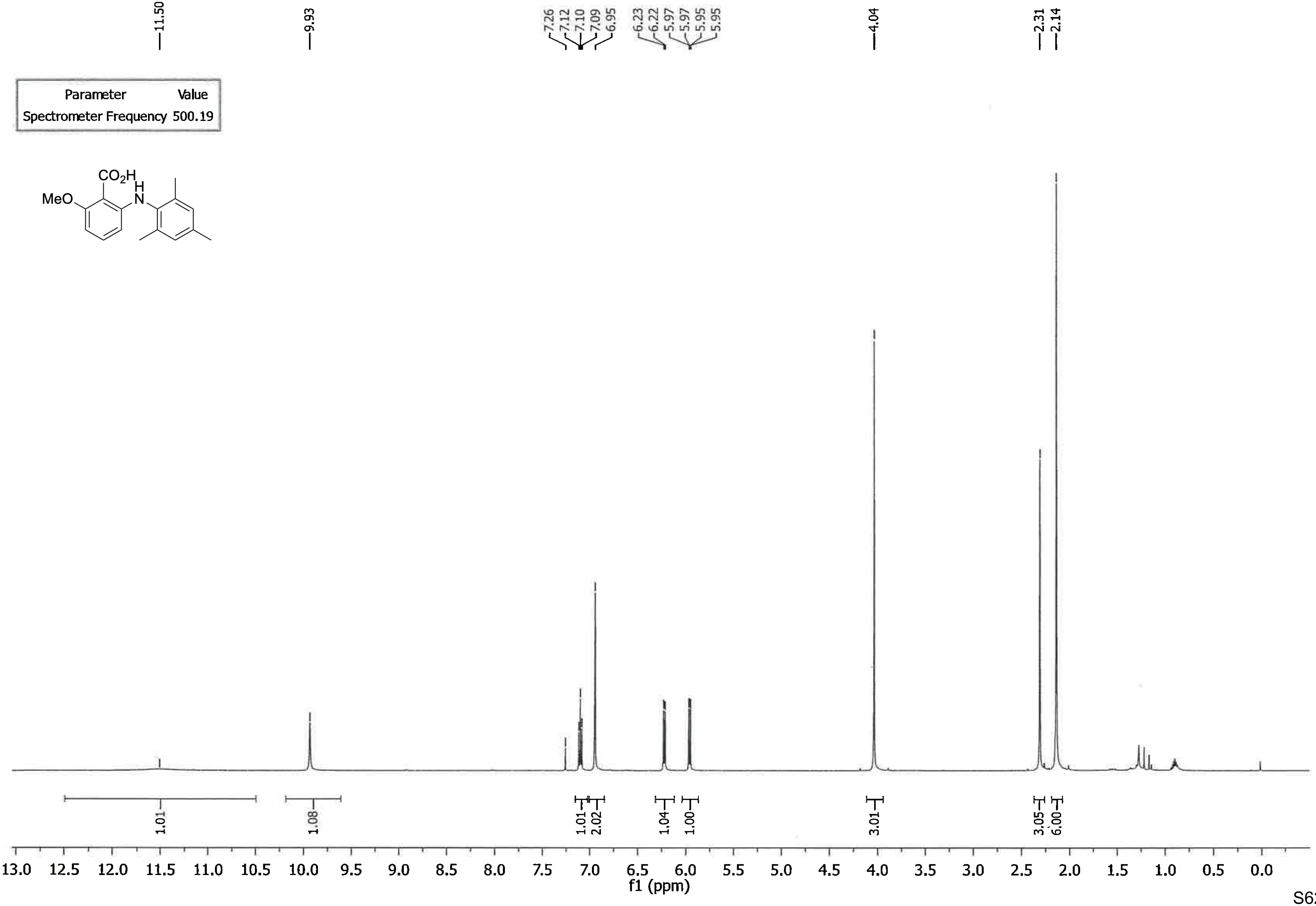

15: 13C{1H} NMR (126 MHz, CDCl3)

Parameter	Value
Spectrometer Frequency	125.77

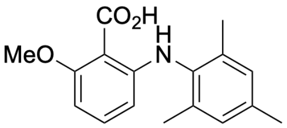

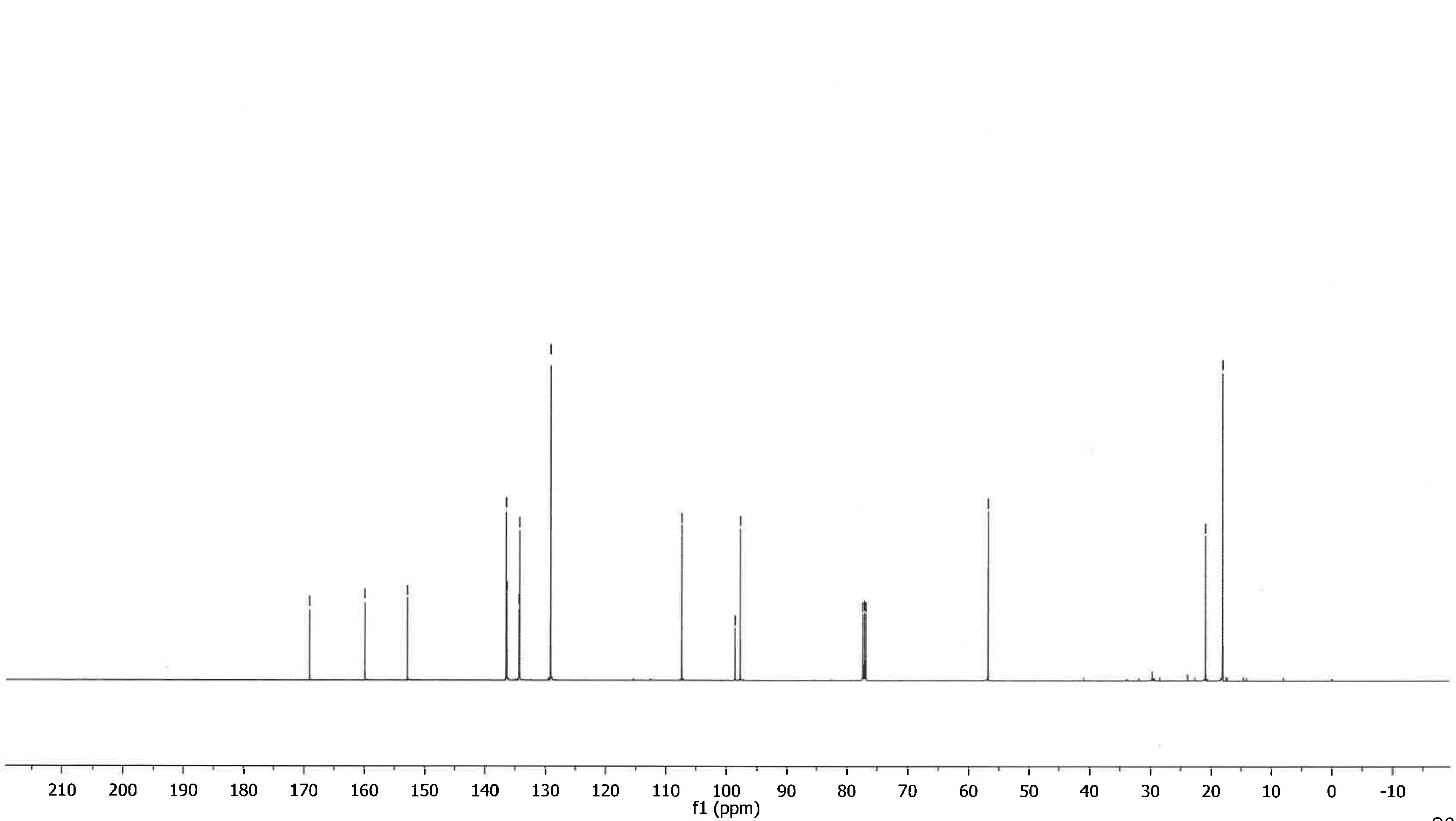

16: 1H NMR (500 MHz, CDCl3)

Parameter	Value
Spectrometer Frequency	500.19

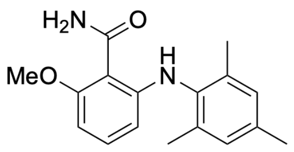

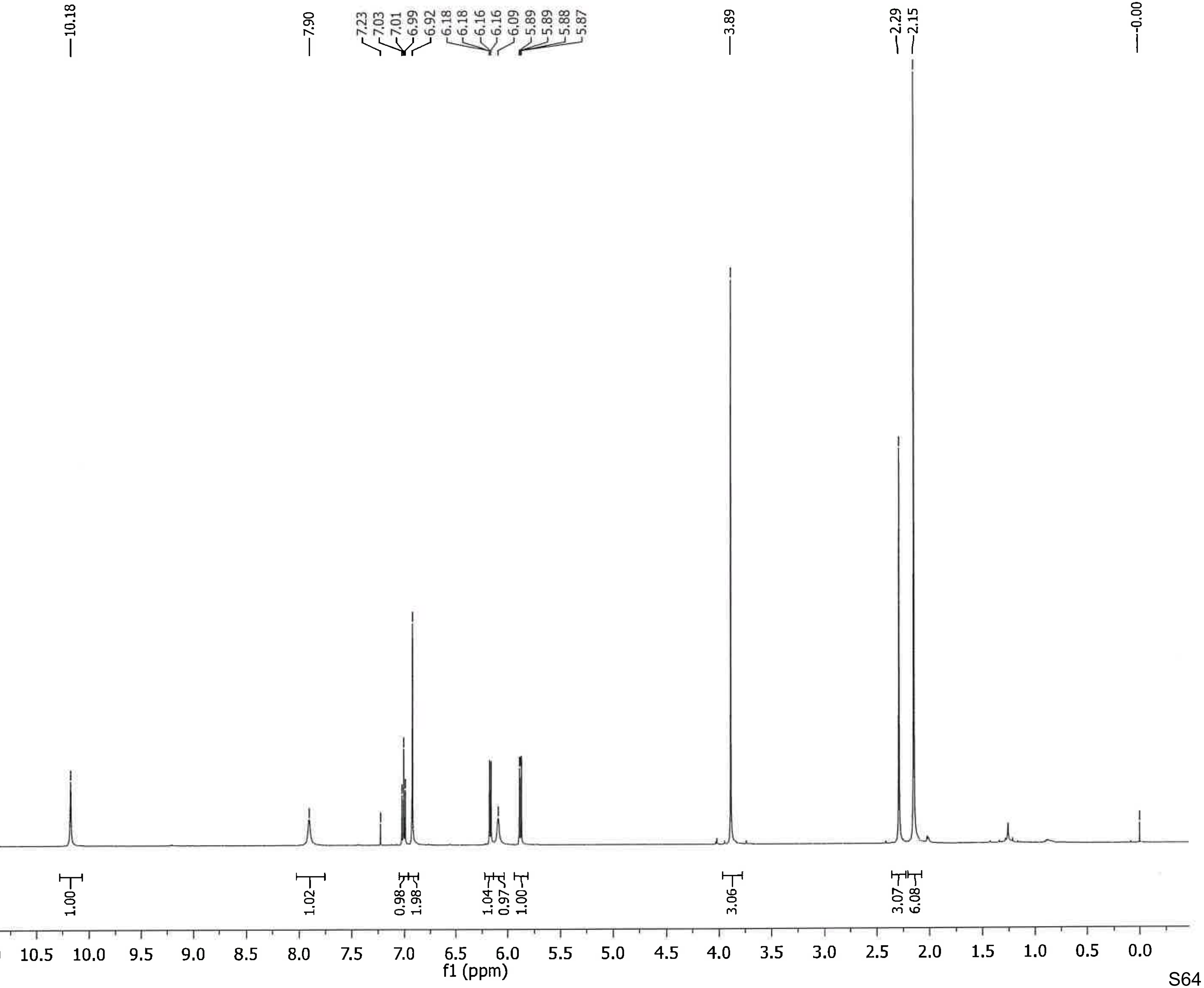

16: 13C{1H} NMR (126 MHz, CDCl3)

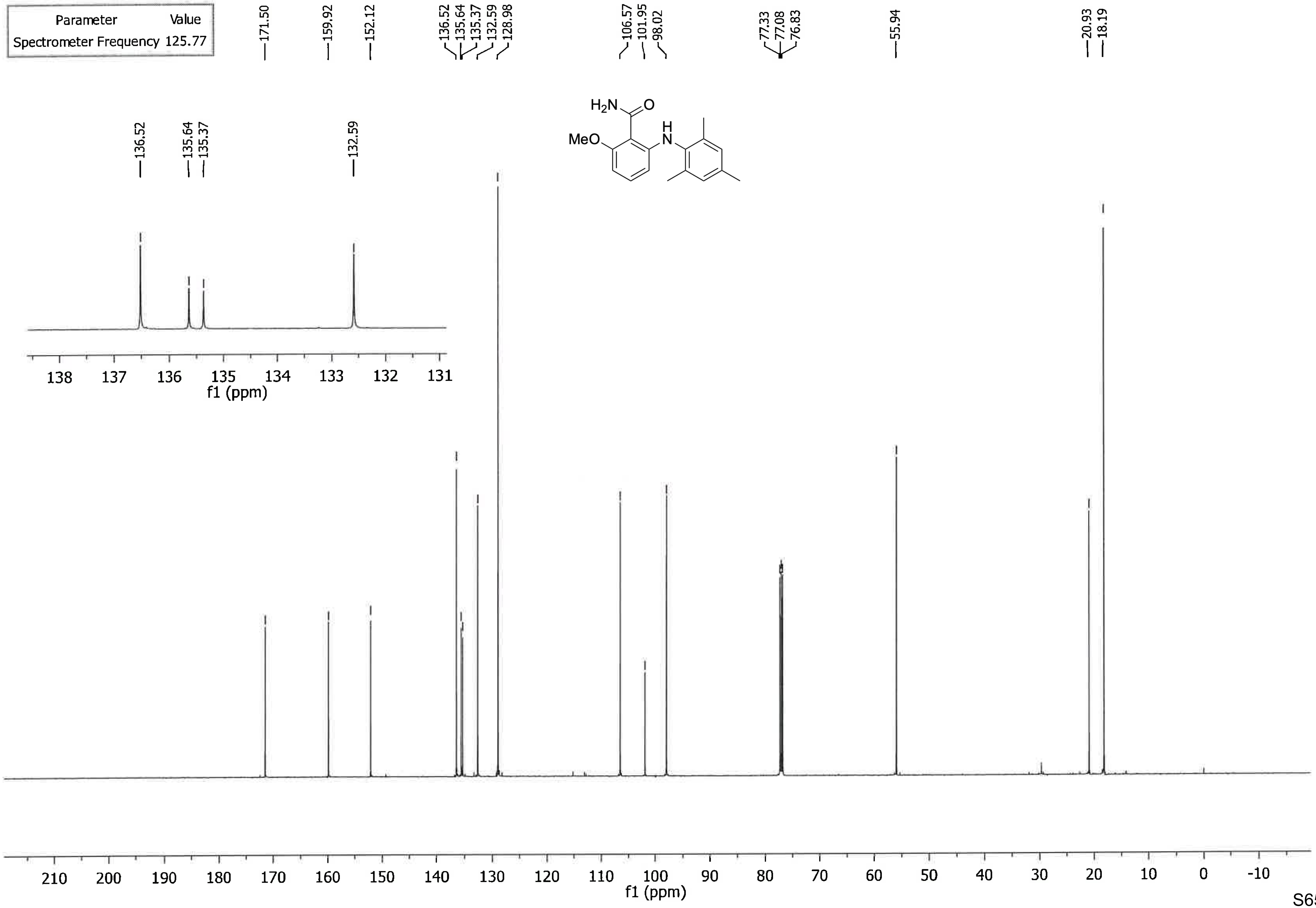

17: 1H NMR (500 MHz, CDCl3)

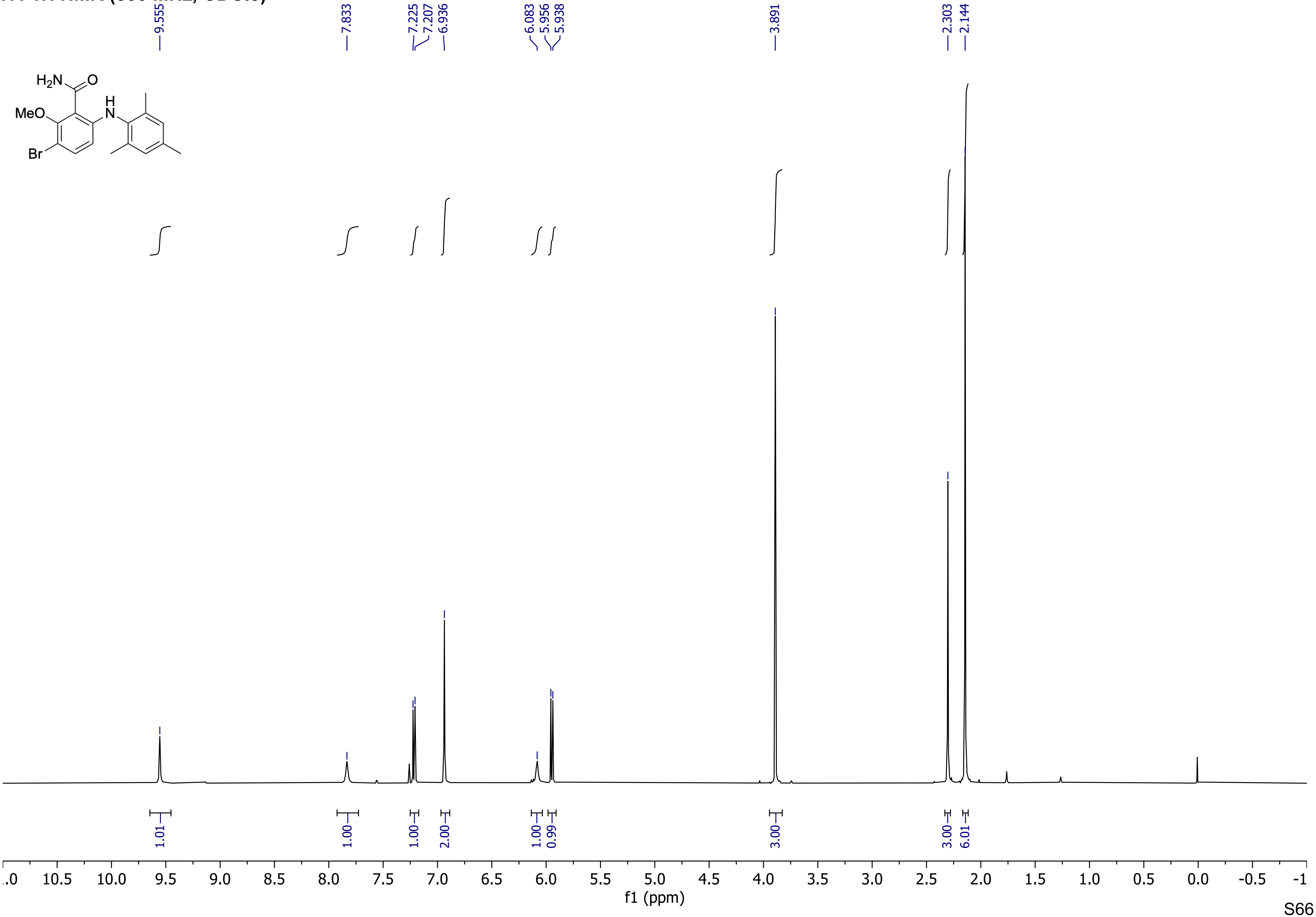

17: 13C{1H} NMR (126 MHz, CDCl3)

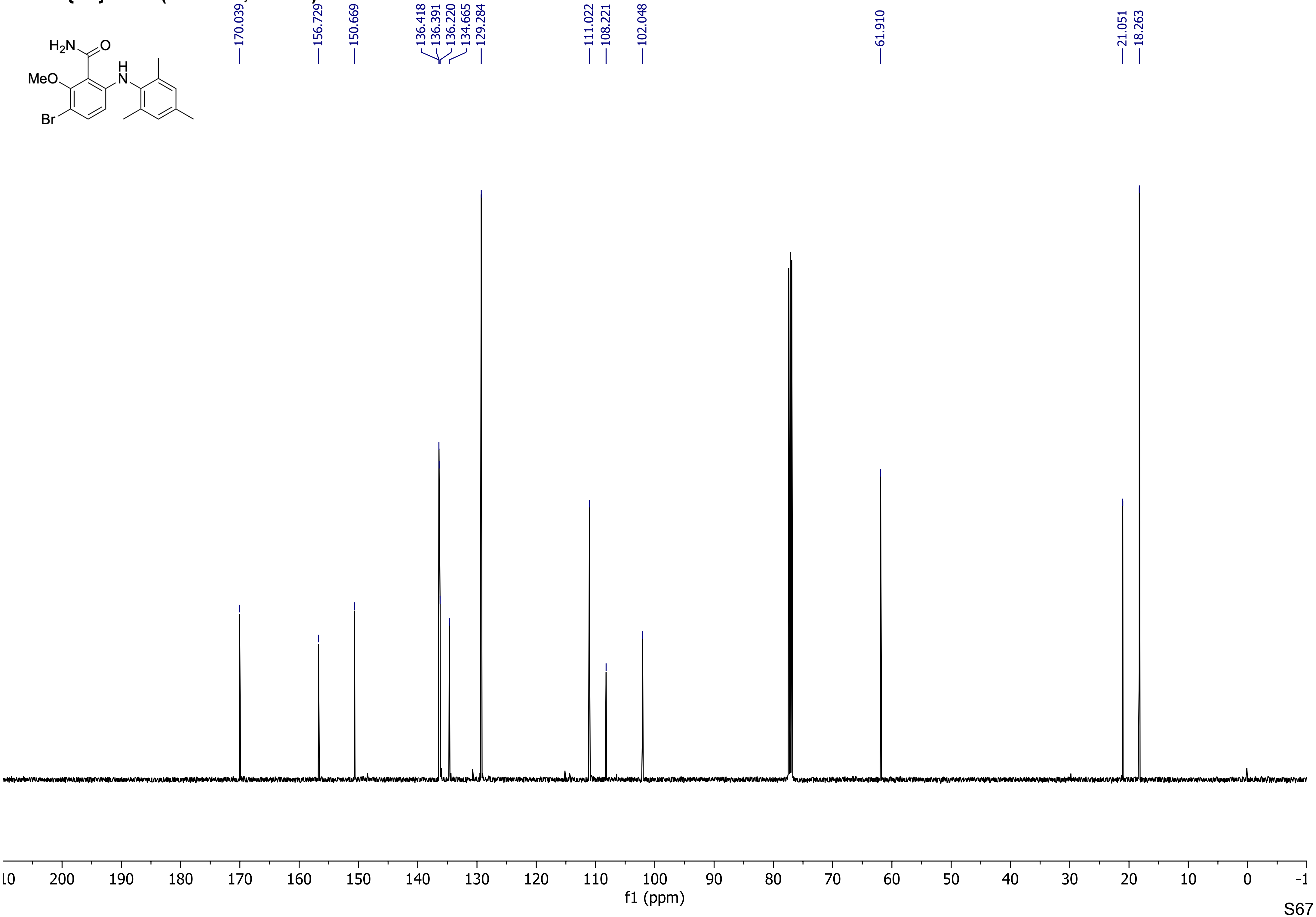

8a: 1H NMR (400 MHz, CDCl3)

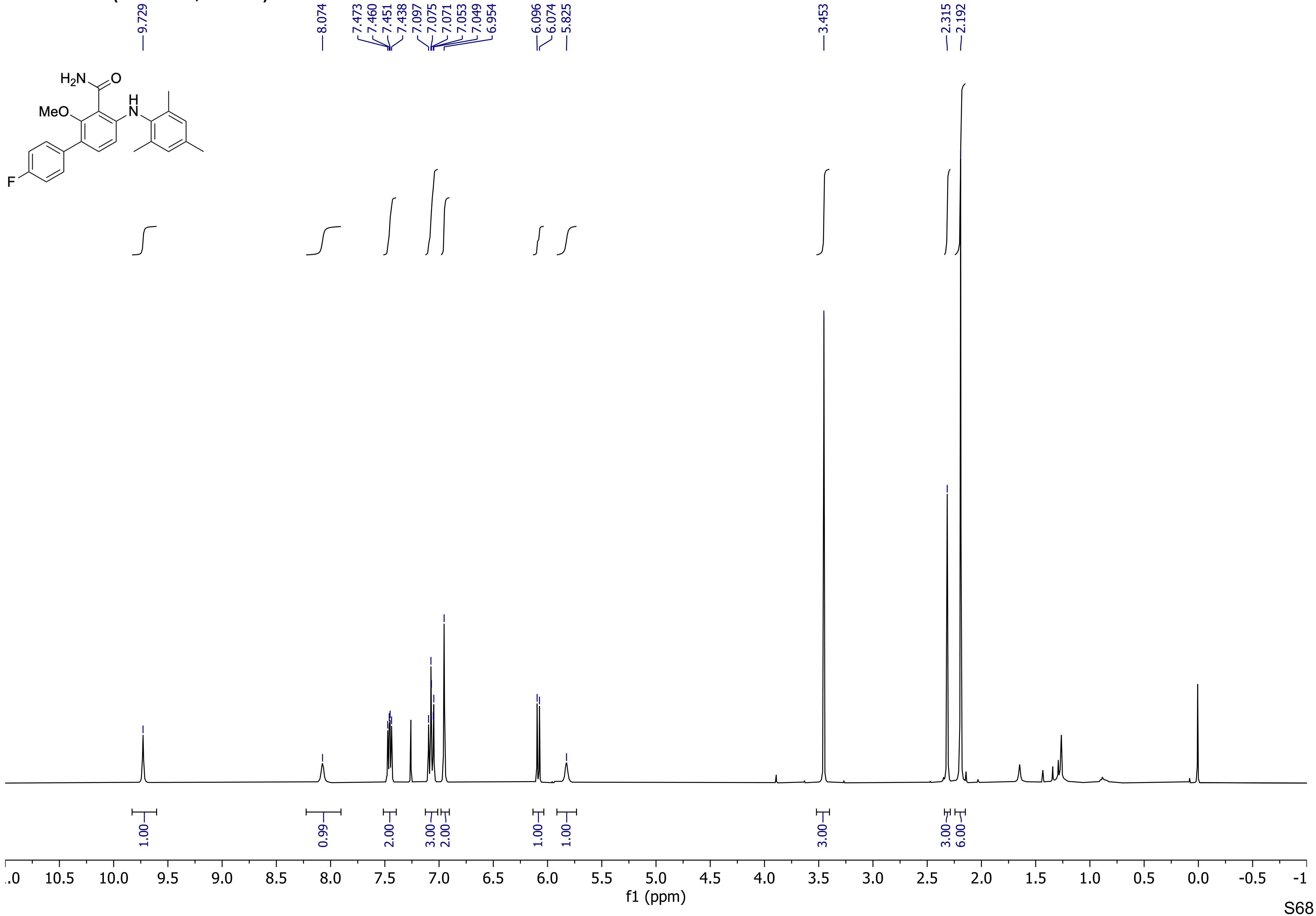

8a: 13C{1H} NMR (101 MHz, CDCl3)

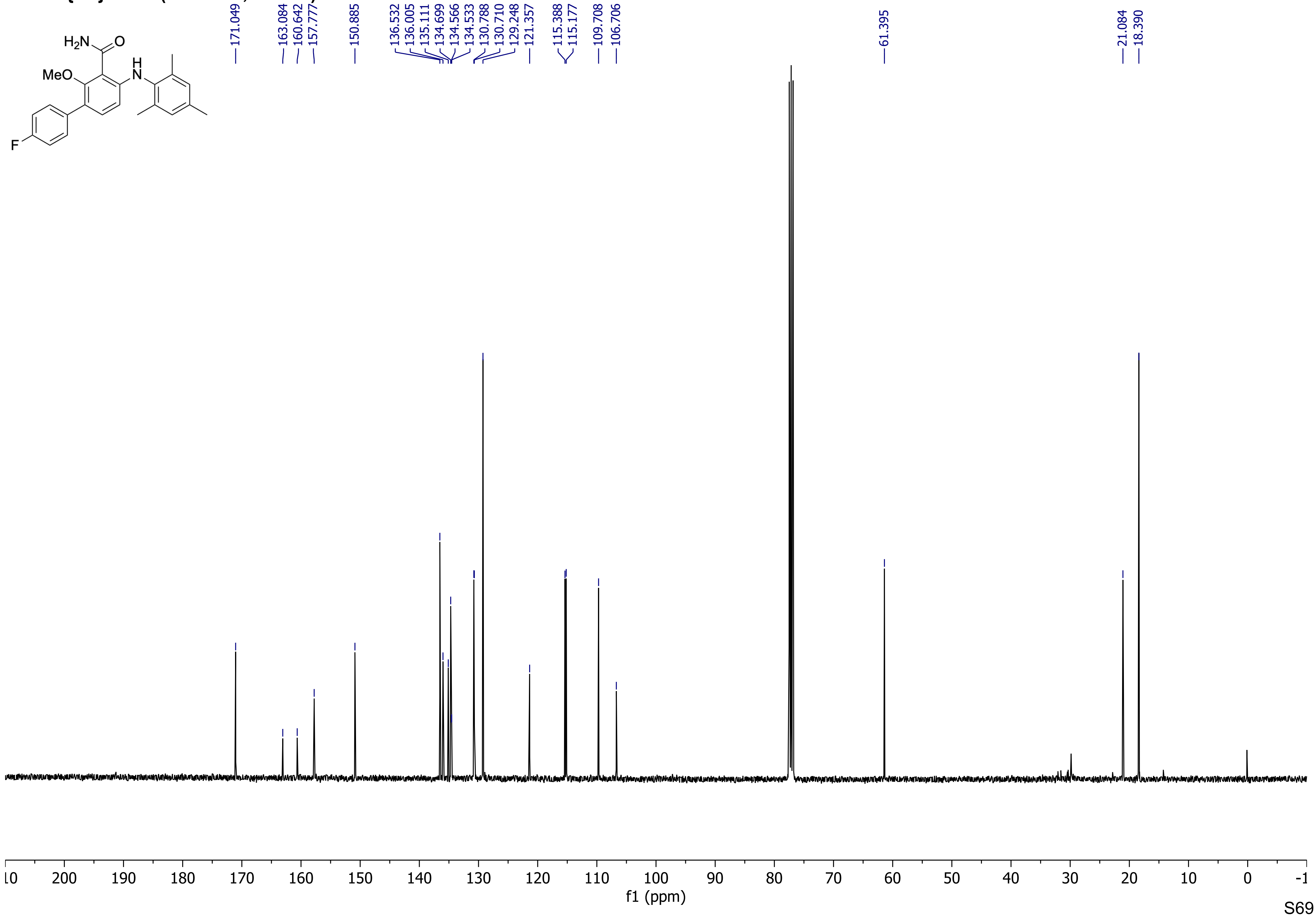

8b: 1H NMR (400 MHz, CDCl3)

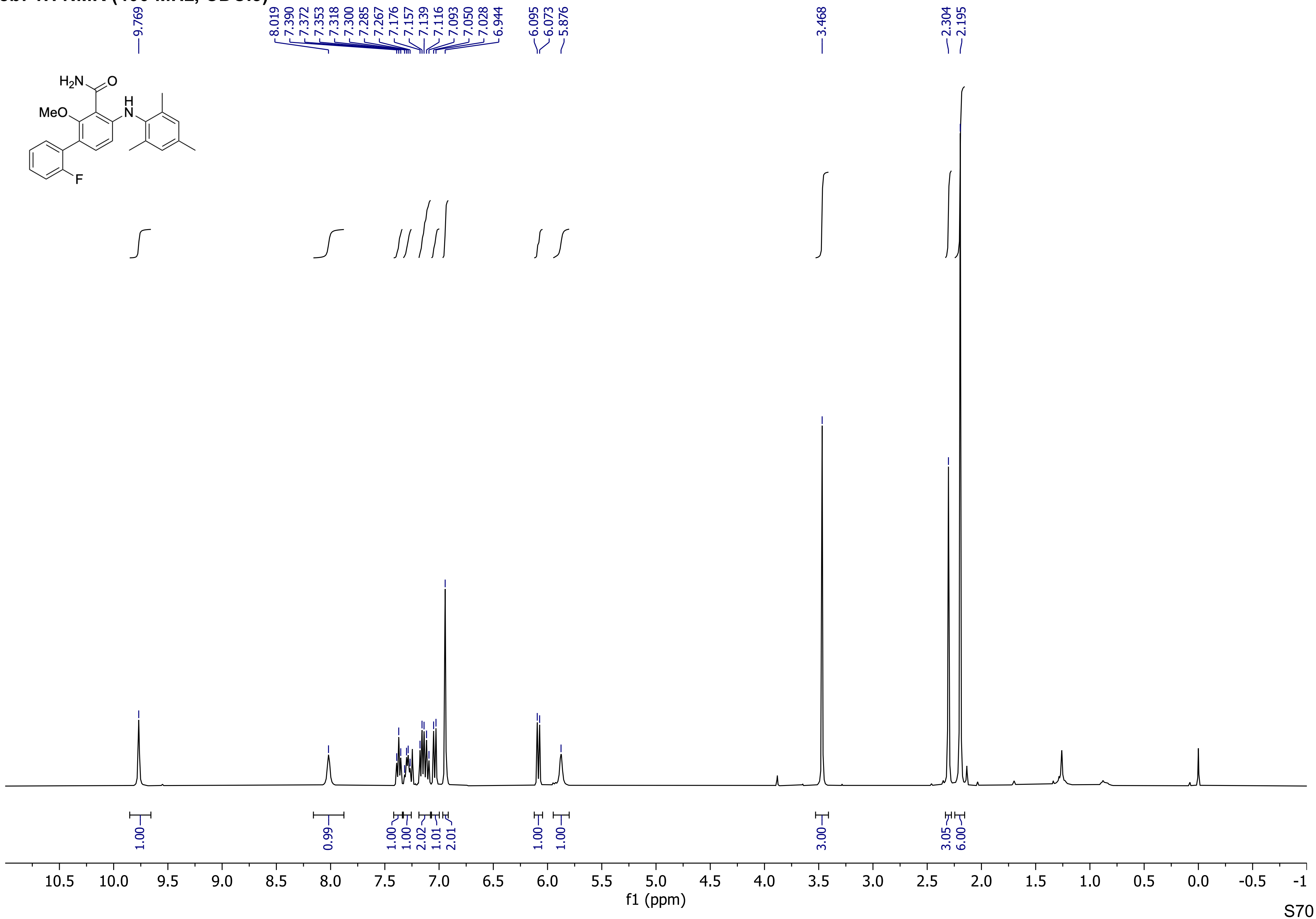

8b: 13C{1H} NMR (101 MHz, CDCl3)

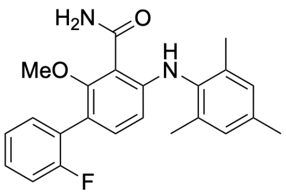

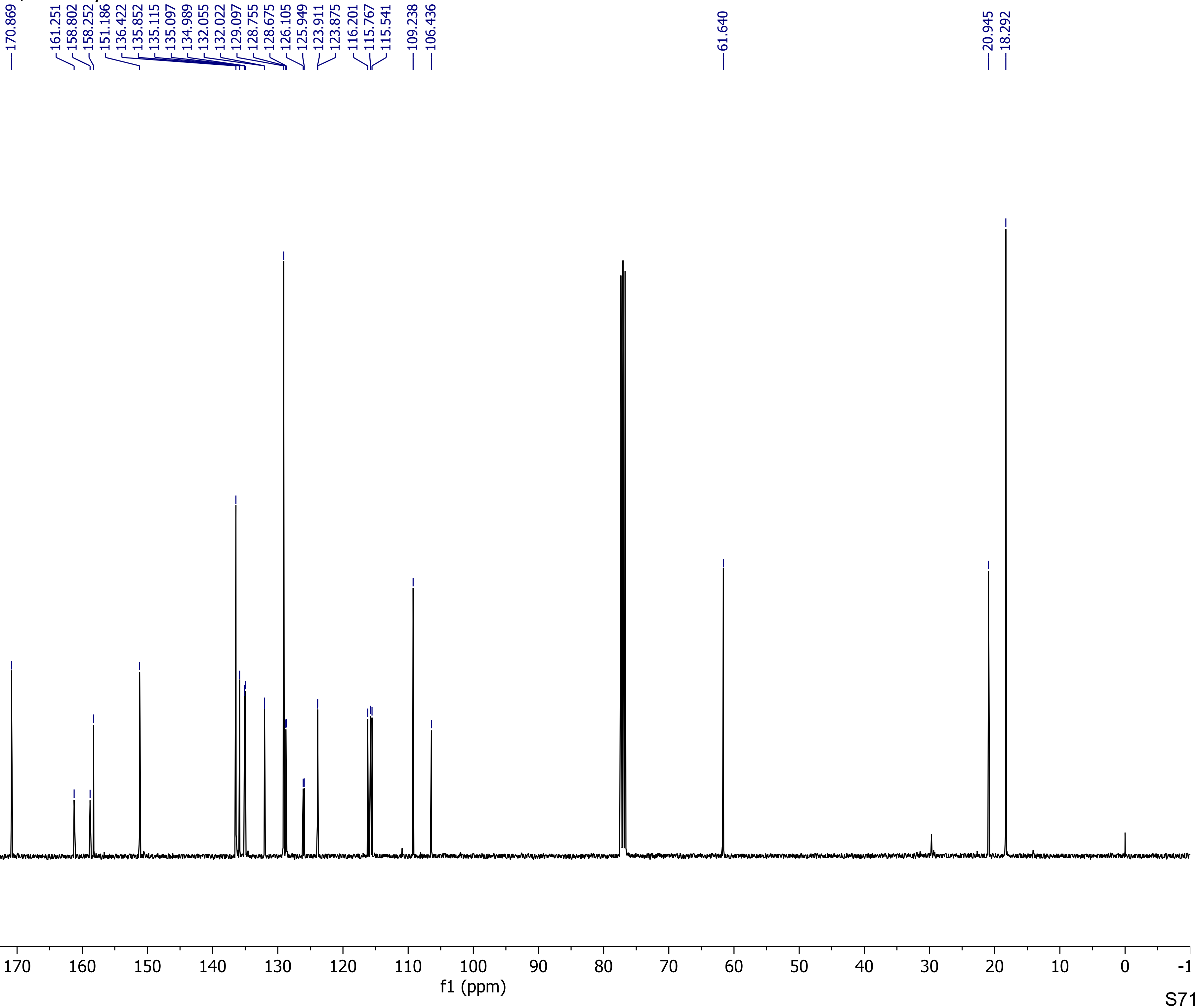

8c: 1H NMR (400 MHz, CDCl3)

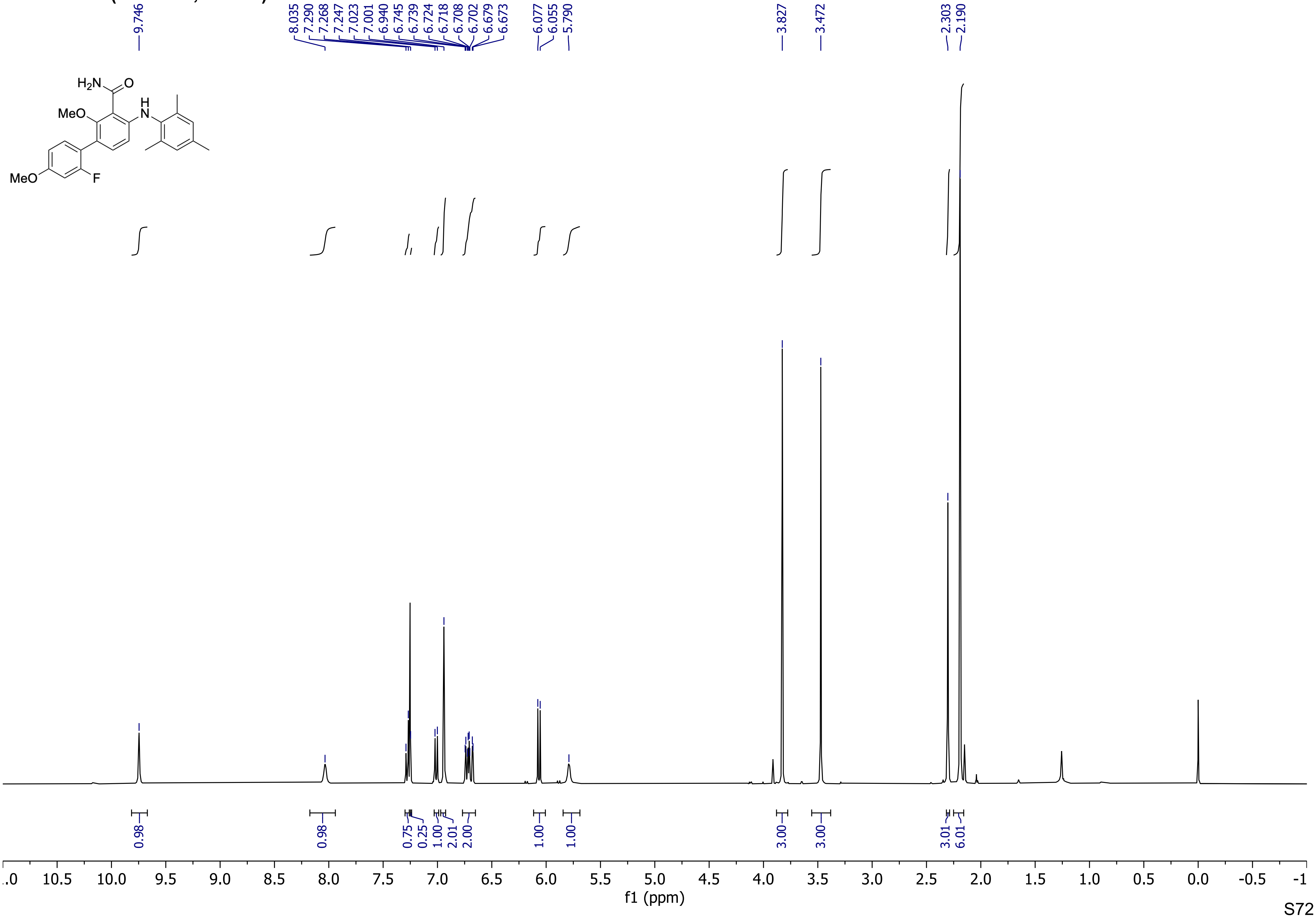

8c: 13C{1H} NMR (101 MHz, CDCl3)

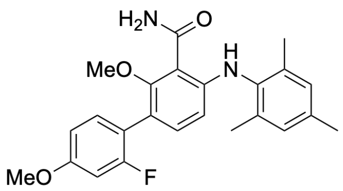

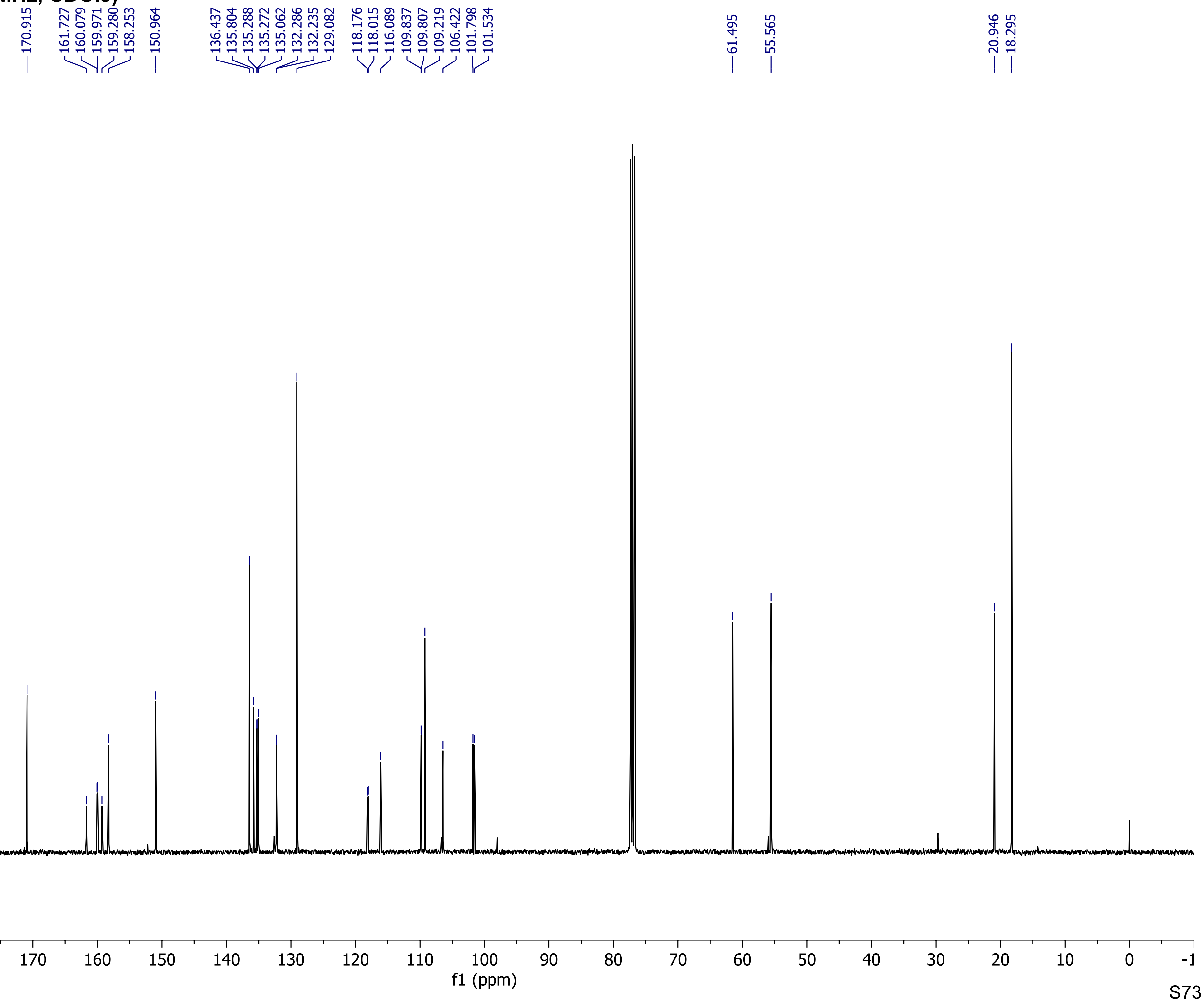

8d: 1H NMR (400 MHz, CDCl3)

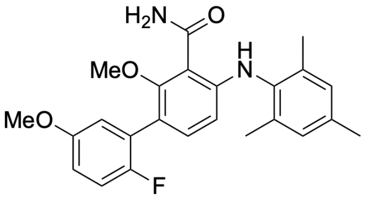

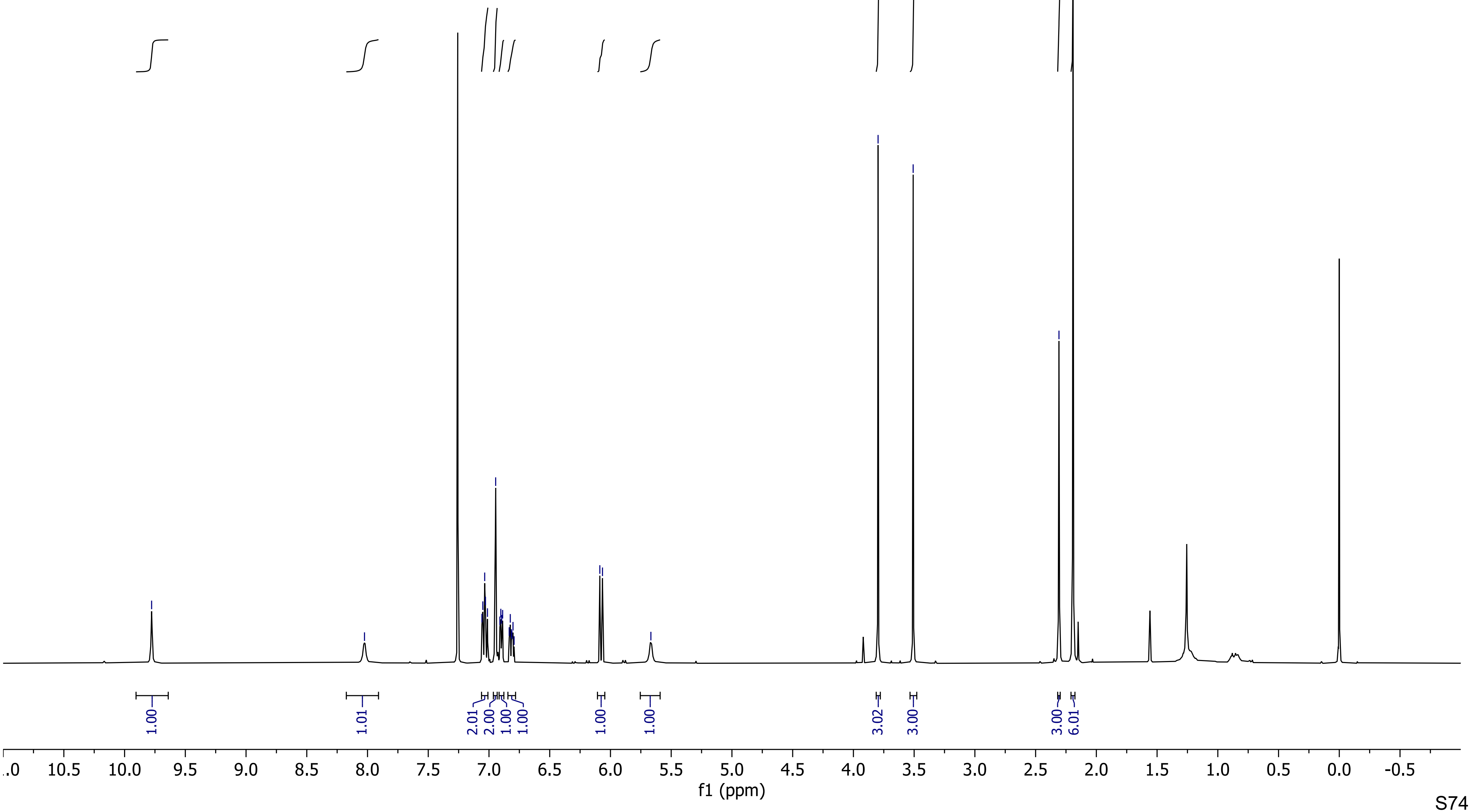

8d: 13C{1H} NMR (101 MHz, CDCl3)

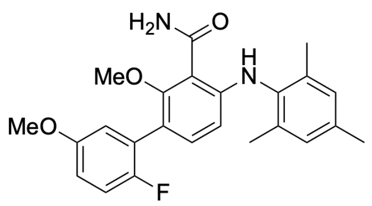

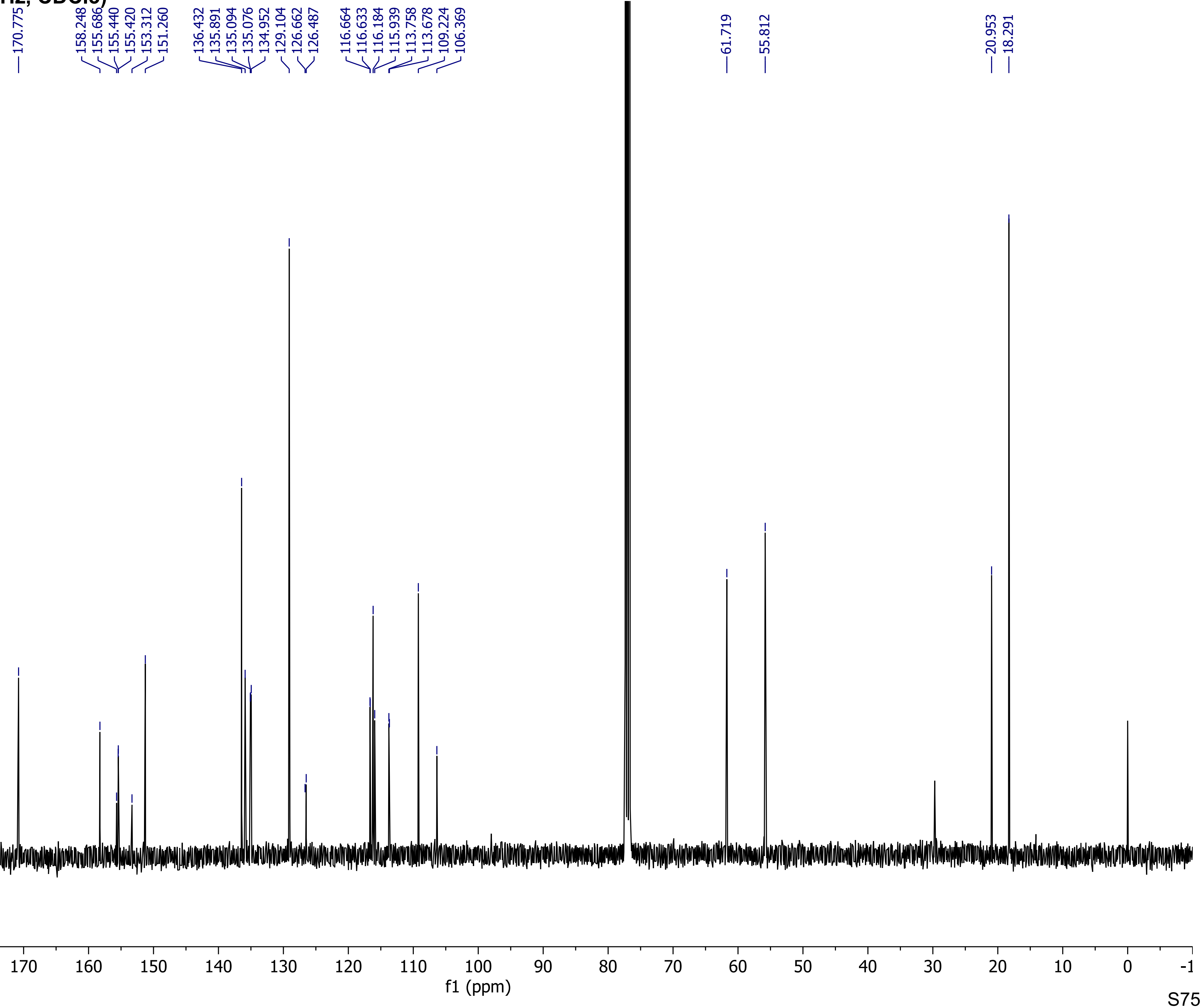

8e: 1H NMR (400 MHz, CDCl3)

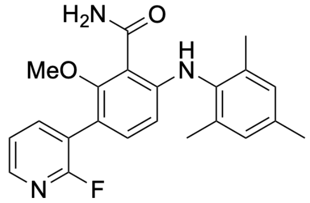

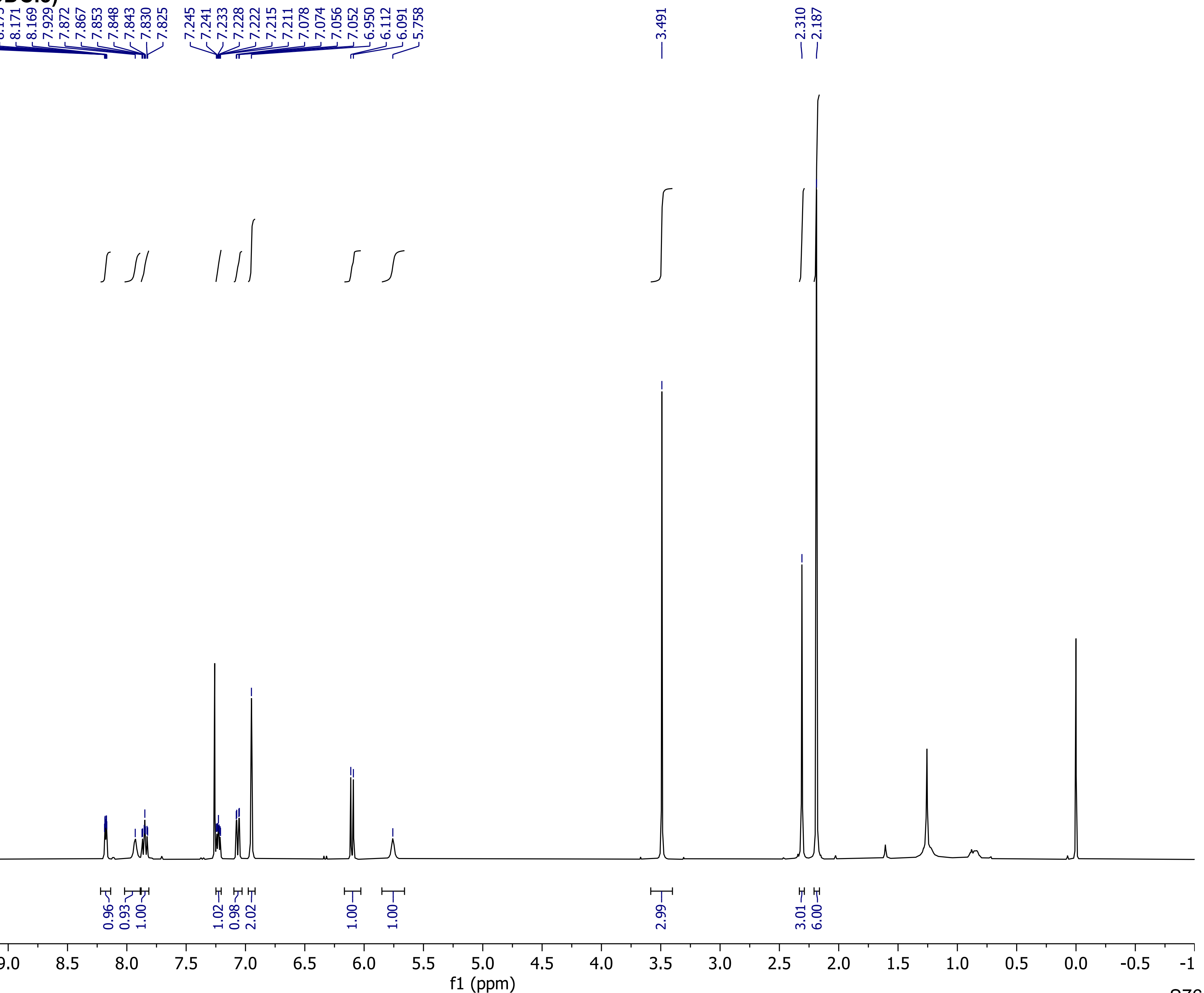

8e: 13C{1H} NMR (101 MHz, CDCl3)

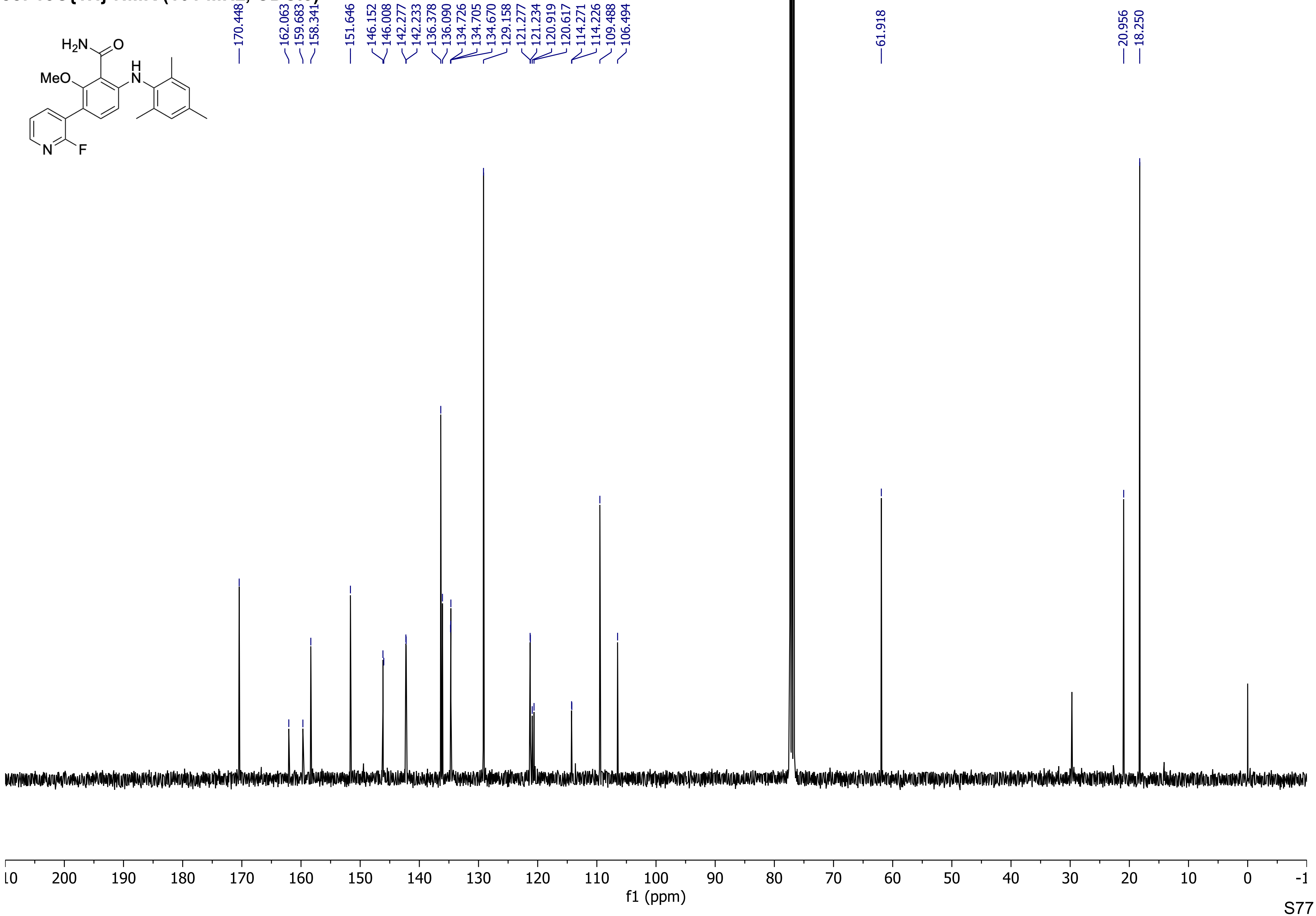

19: 1H NMR (500 MHz, CDCl3)

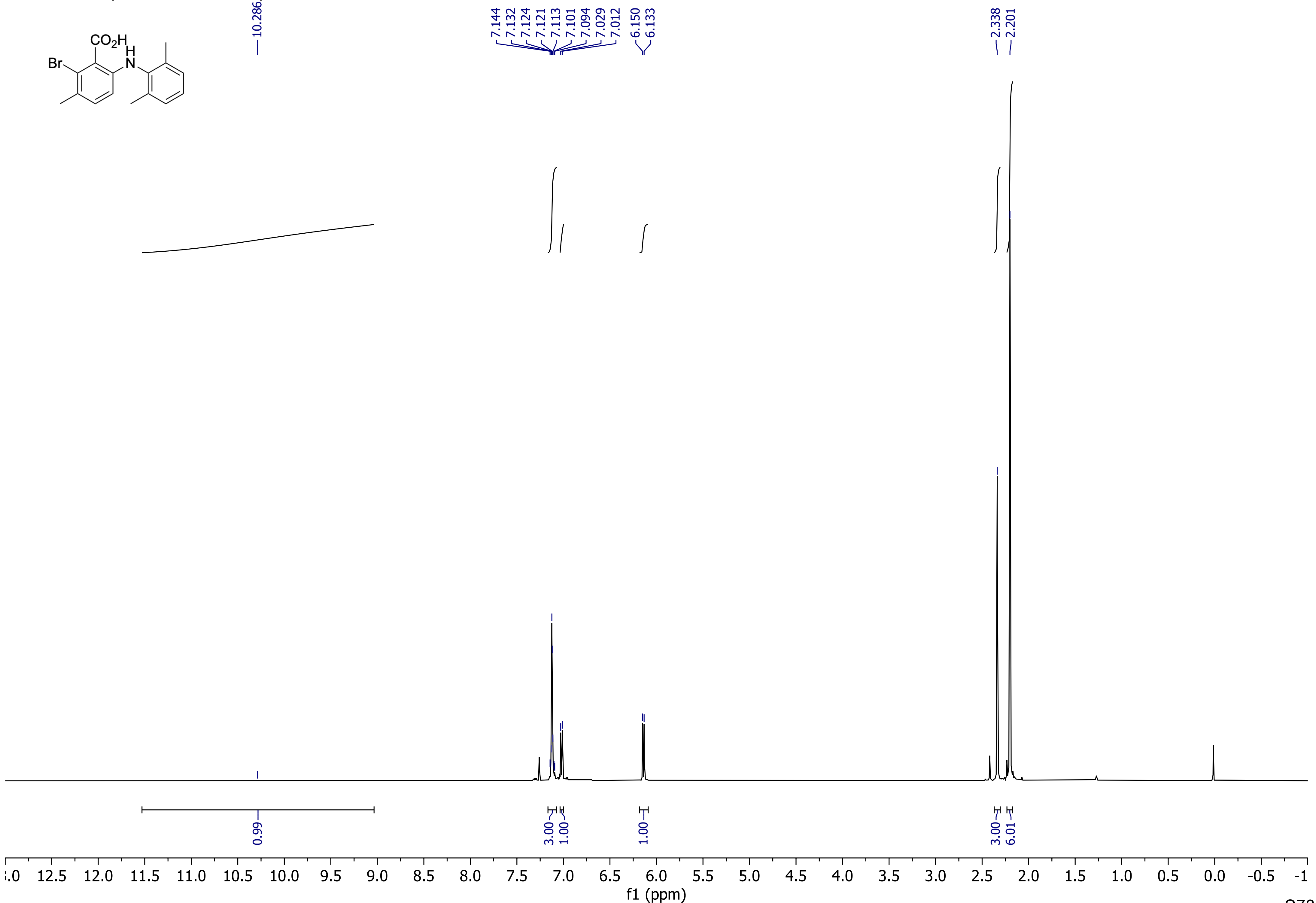

19: 13C{1H} NMR (126 MHz, CDCl3)

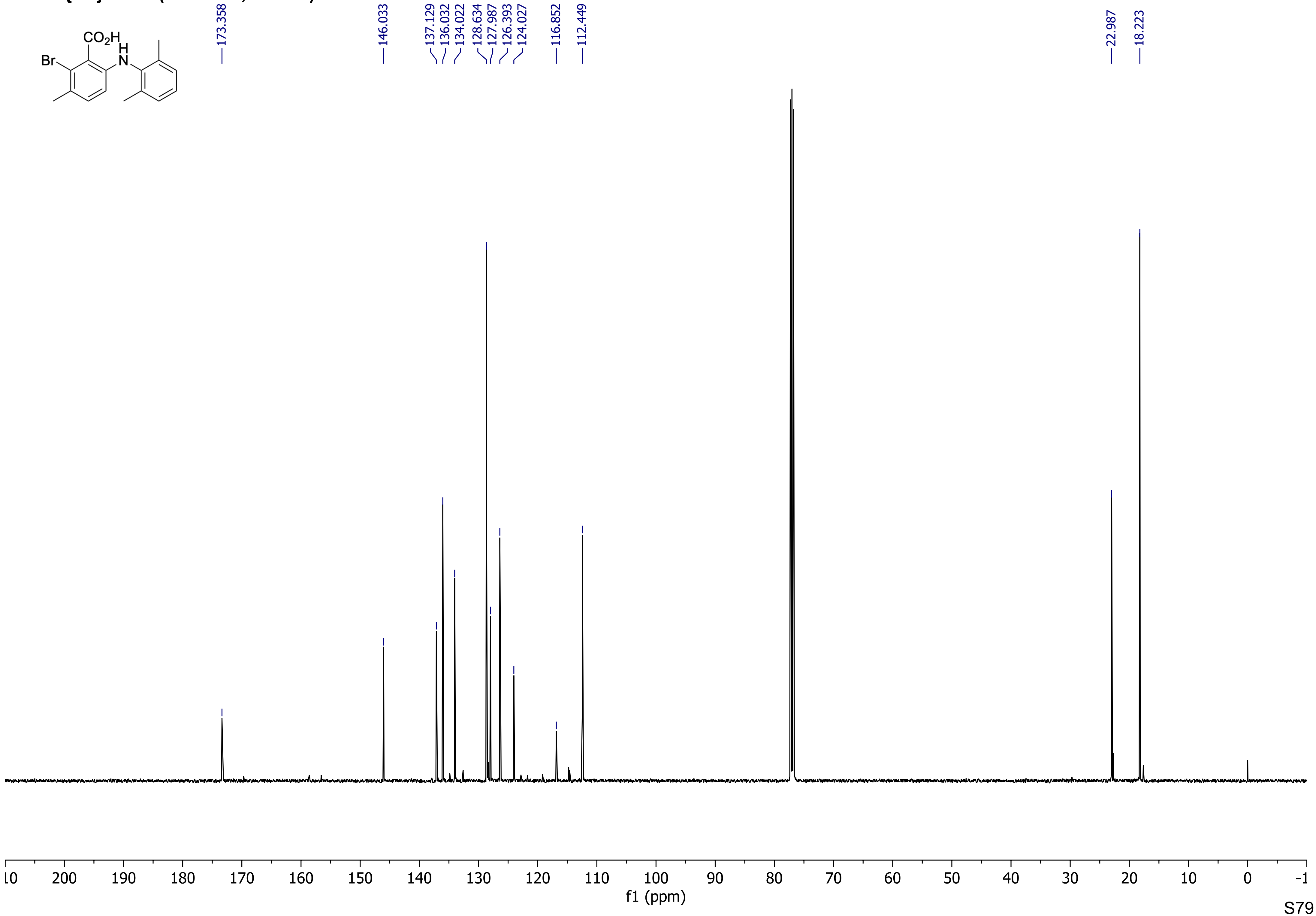

20: 1H NMR (500 MHz, CDCl3)

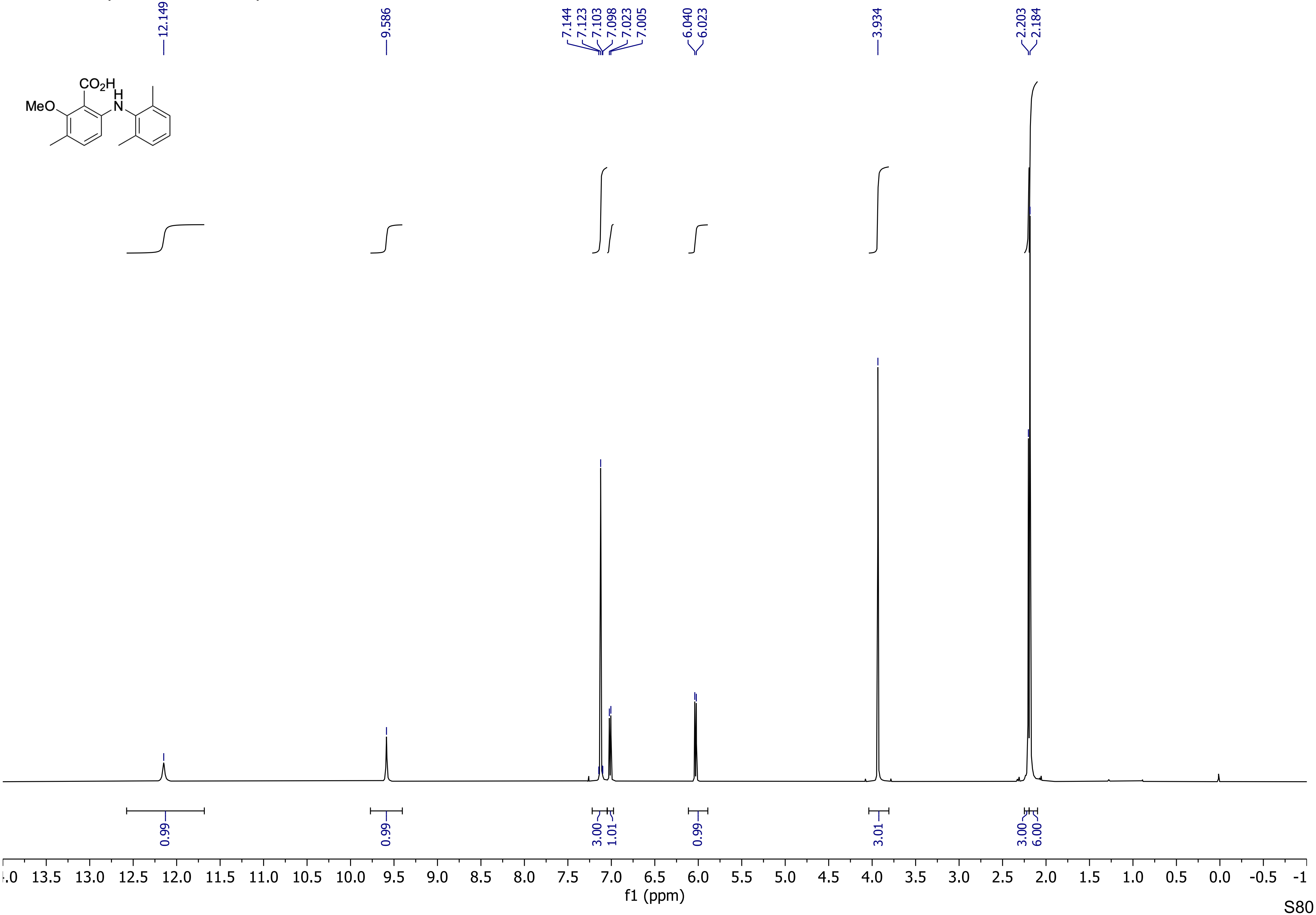

20: 13C{1H} NMR (126 MHz, CDCl3)

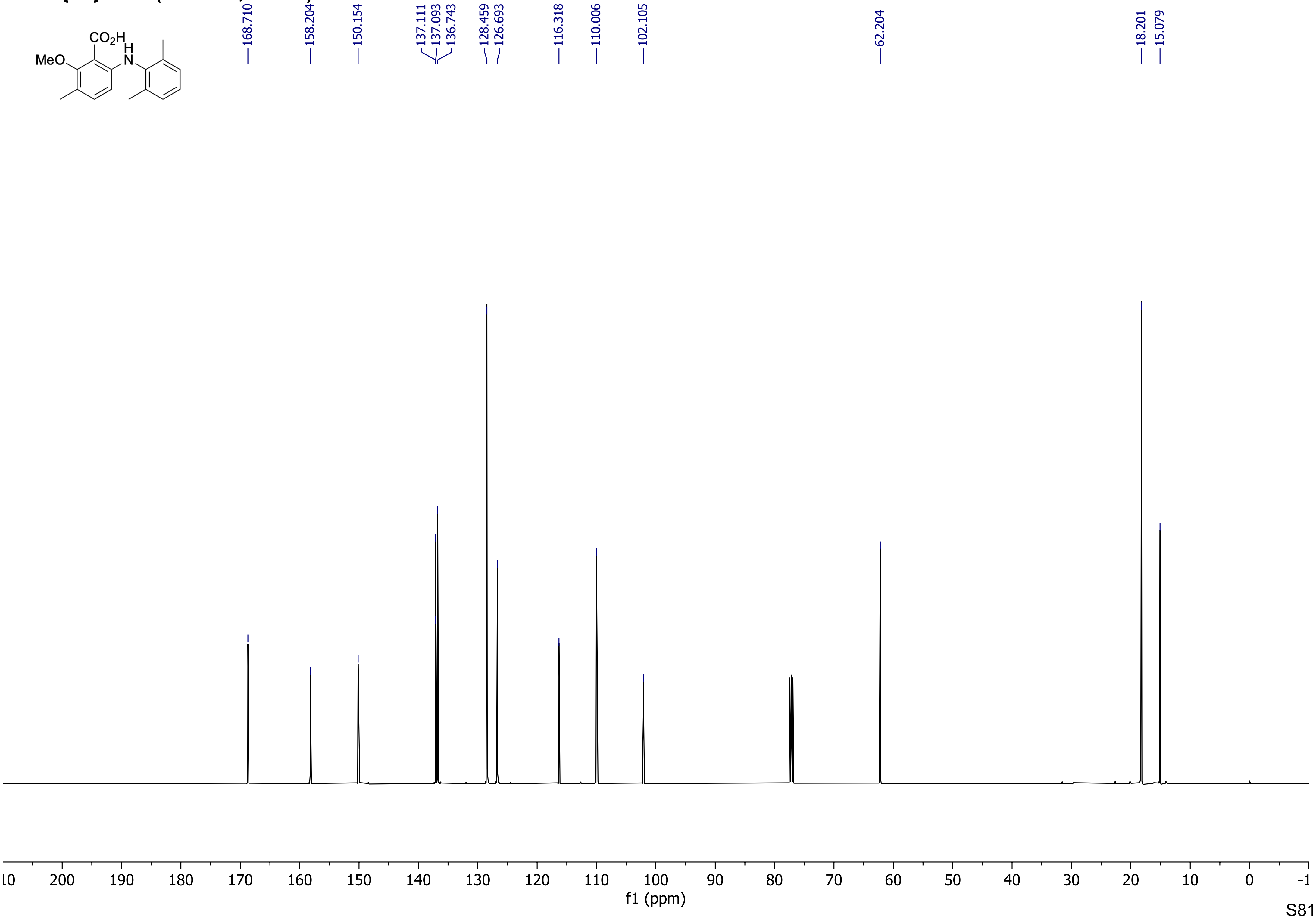

21: 1H NMR (400 MHz, CDCl3)

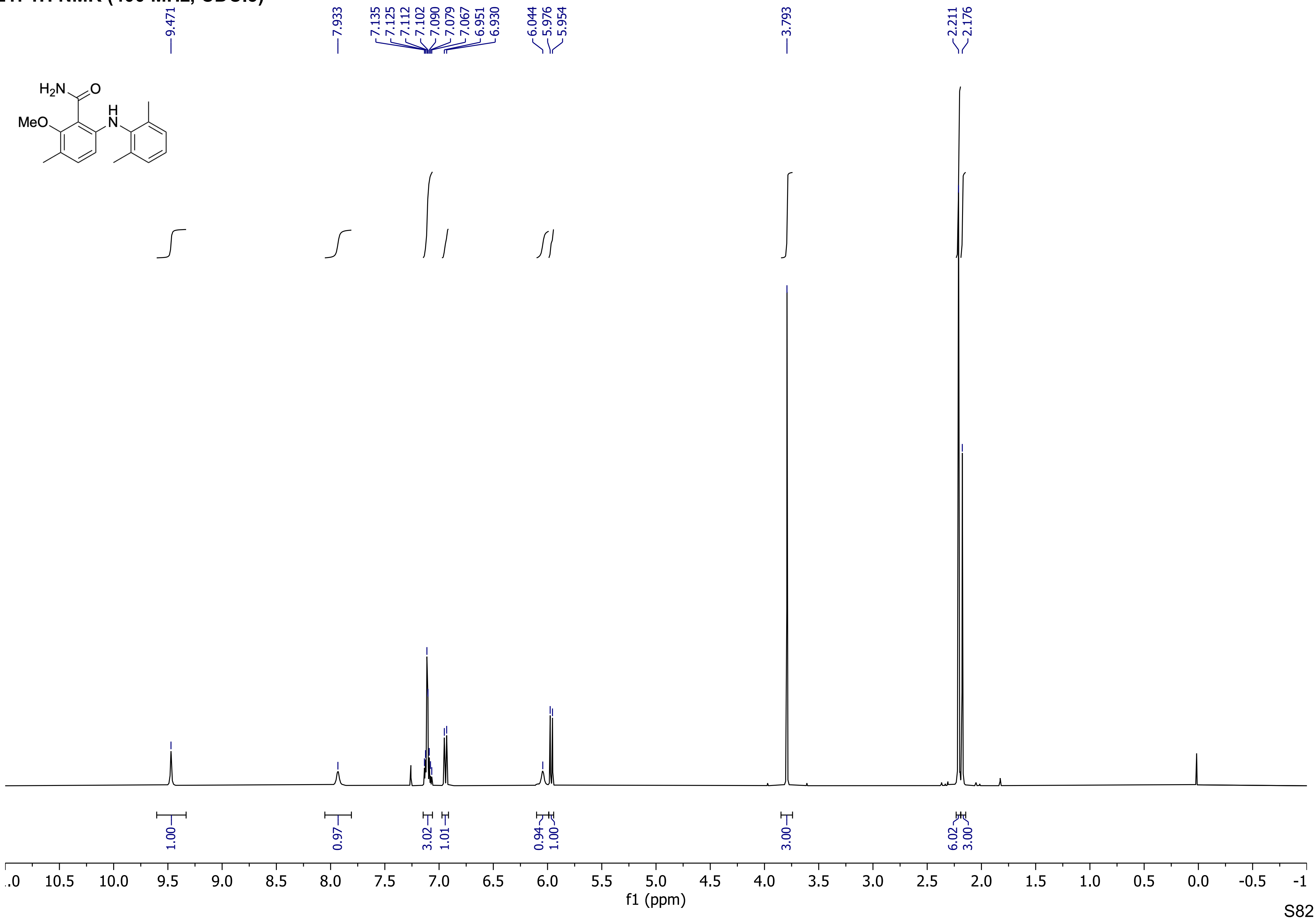

21: 13C{1H} NMR (101 MHz, CDCl3)

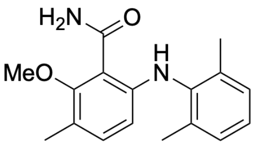

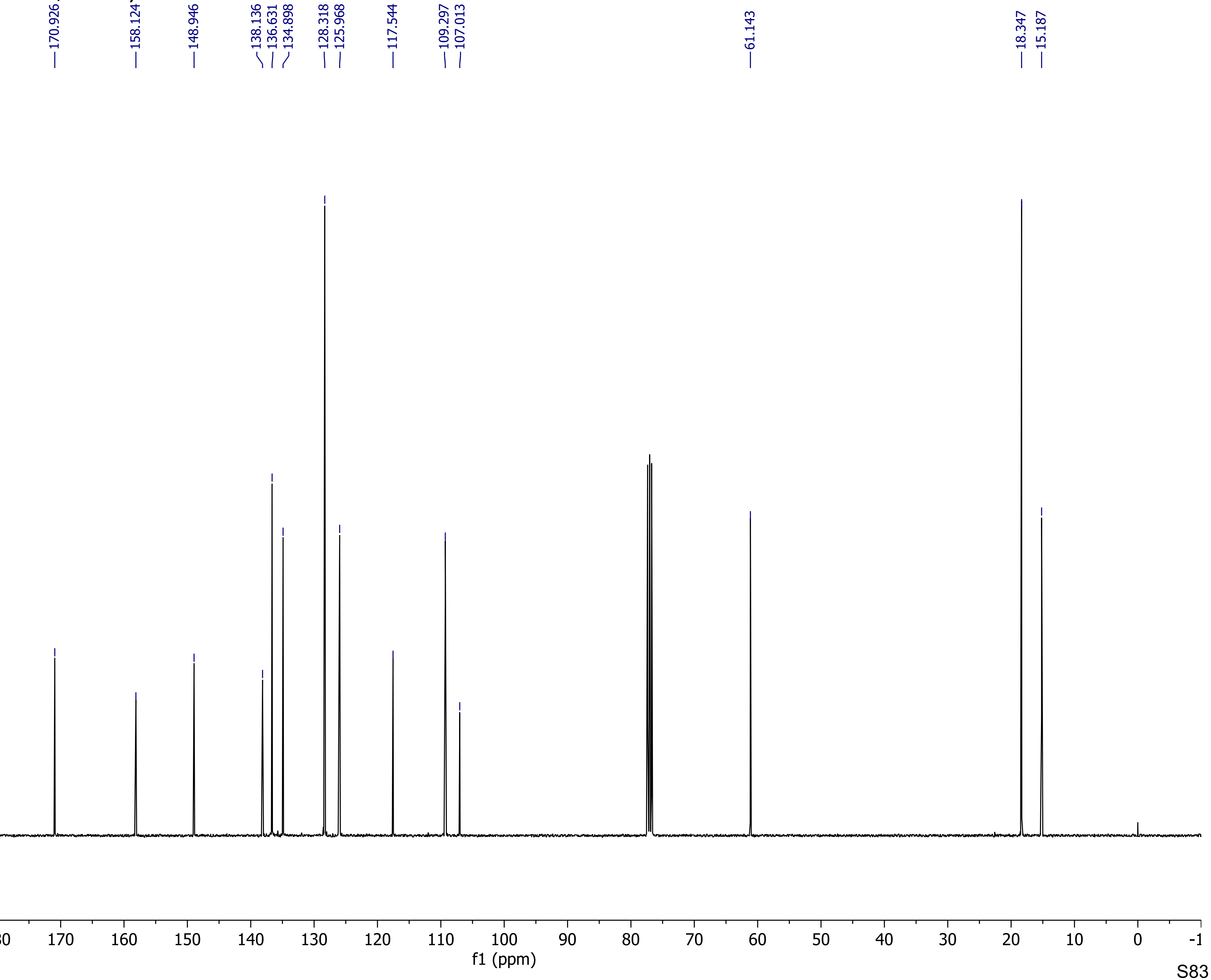

22: 1H NMR (500 MHz, CDCl3)

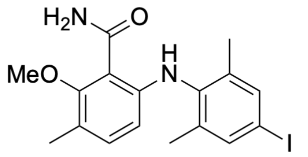

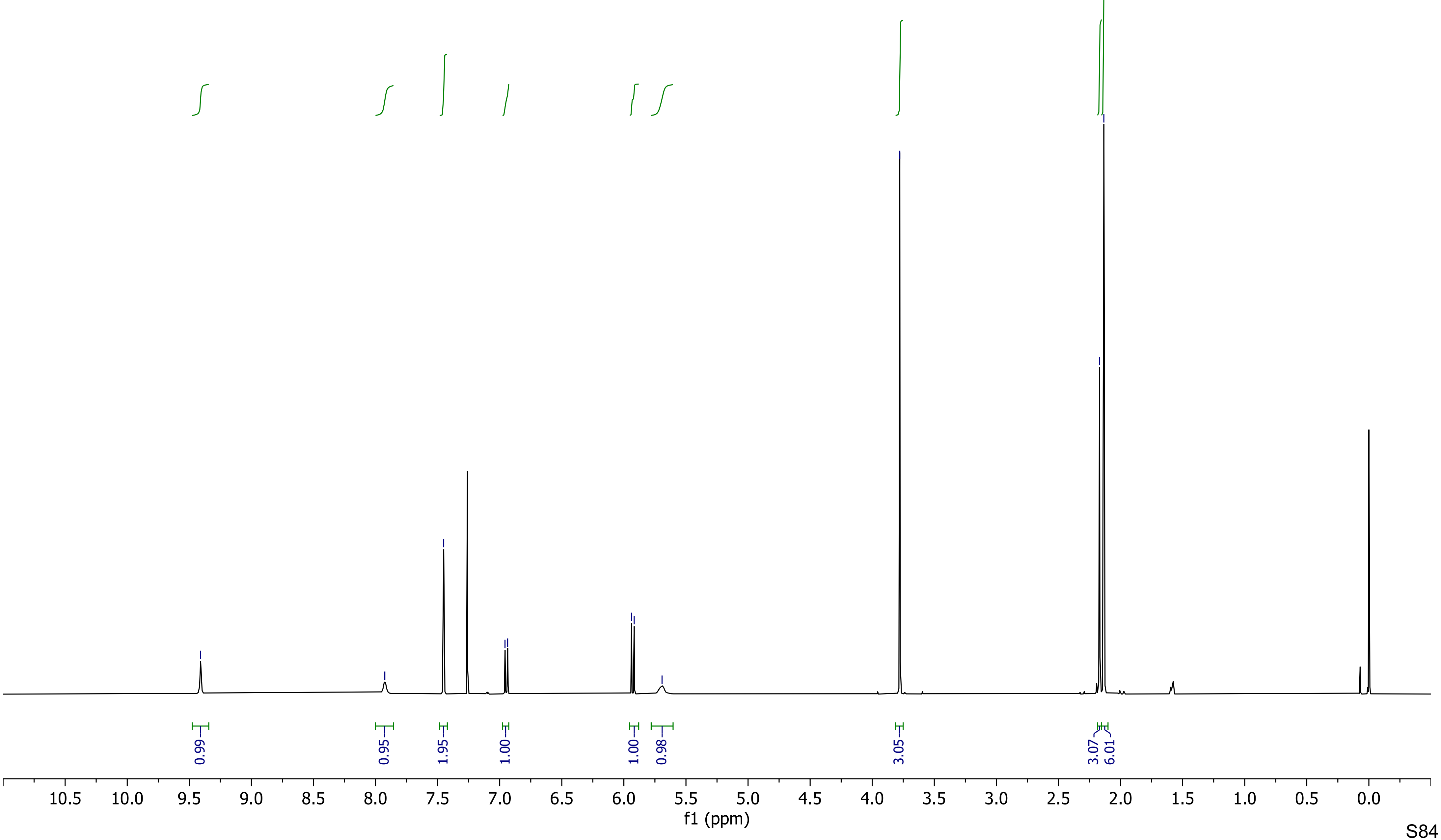

22: 13C{1H} NMR (126 MHz, CDCl3)

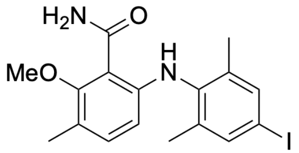

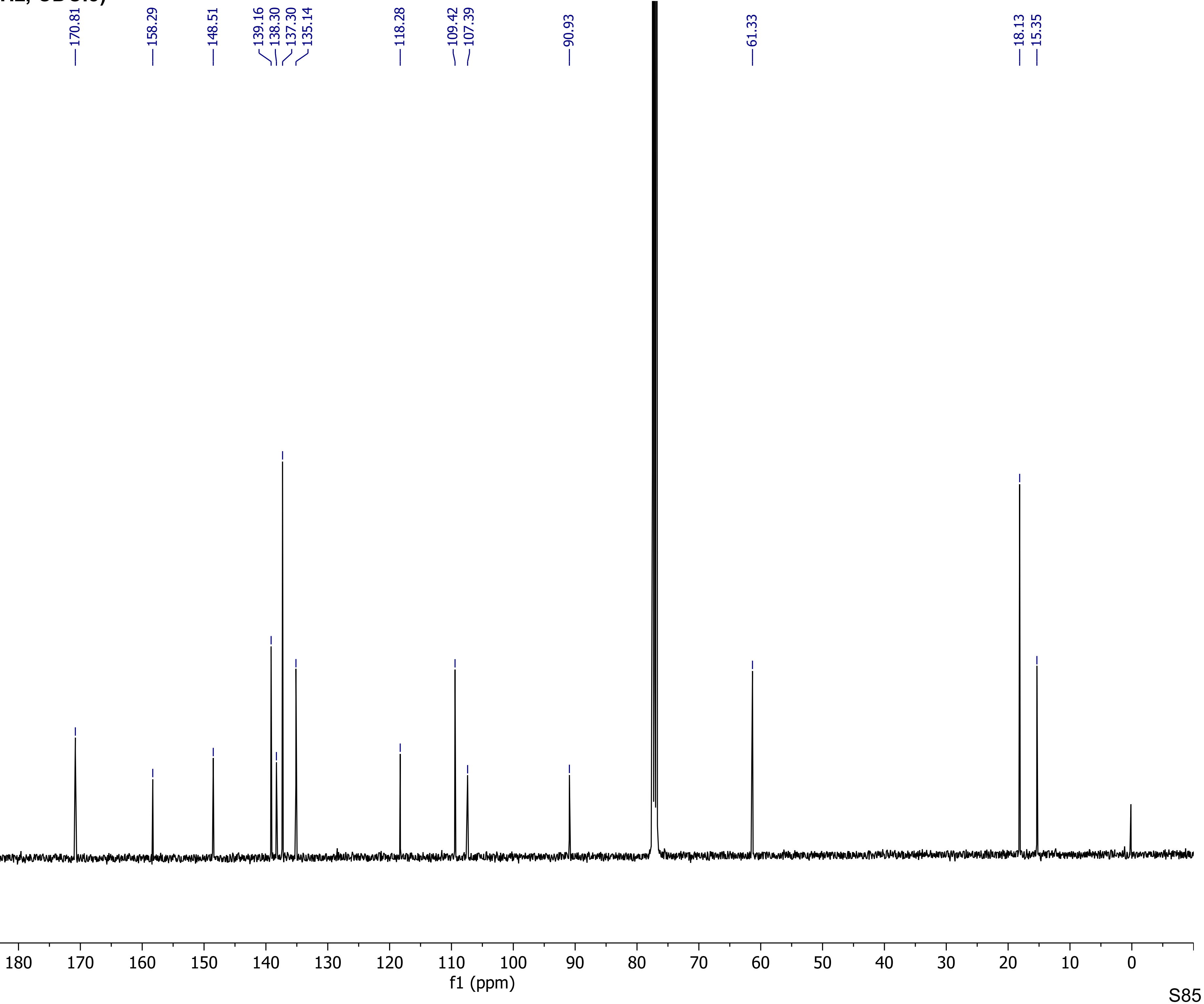

23: 1H NMR (500 MHz, CDCl3)

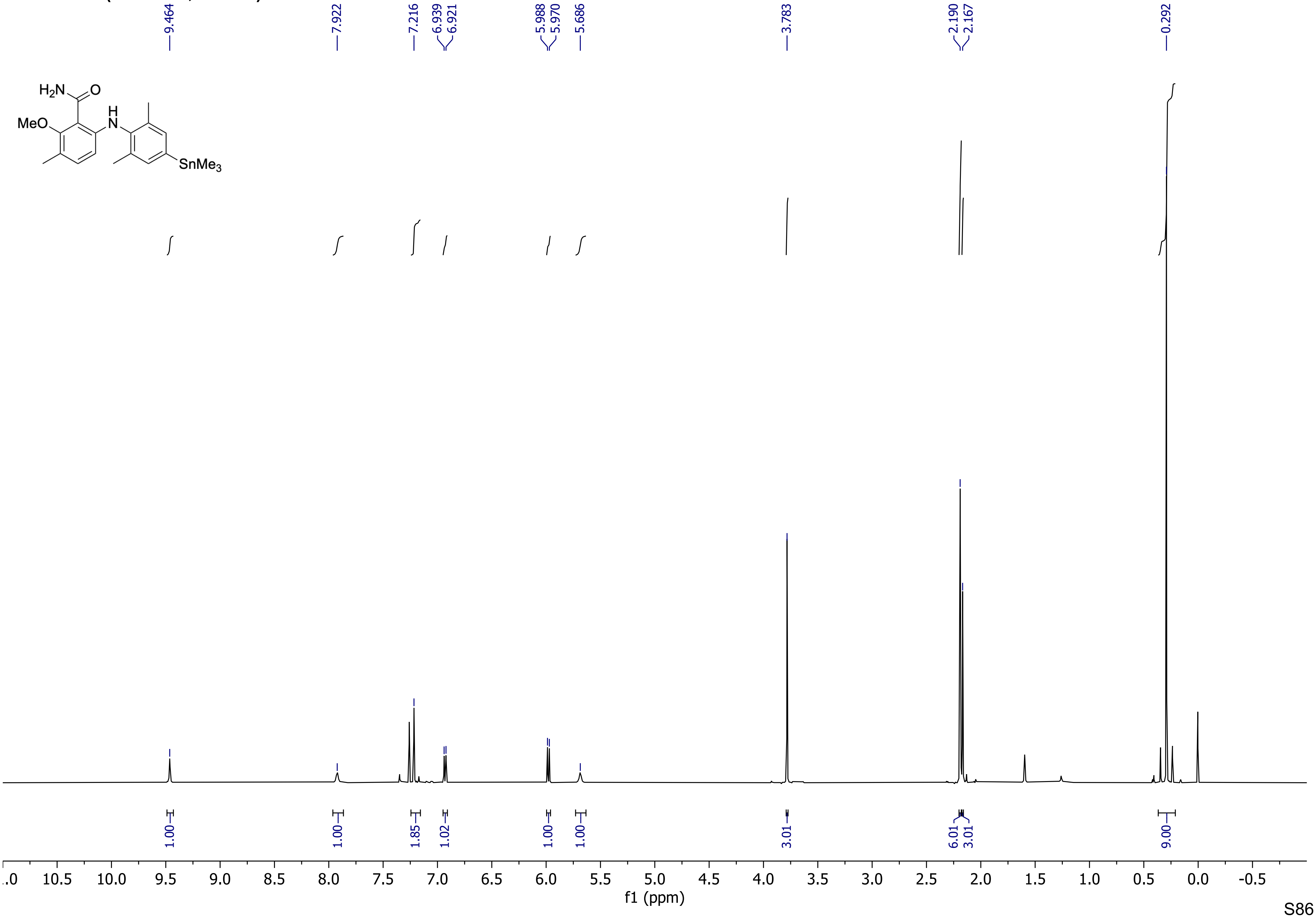

23: 13C{1H} NMR (126 MHz, CDCl3)

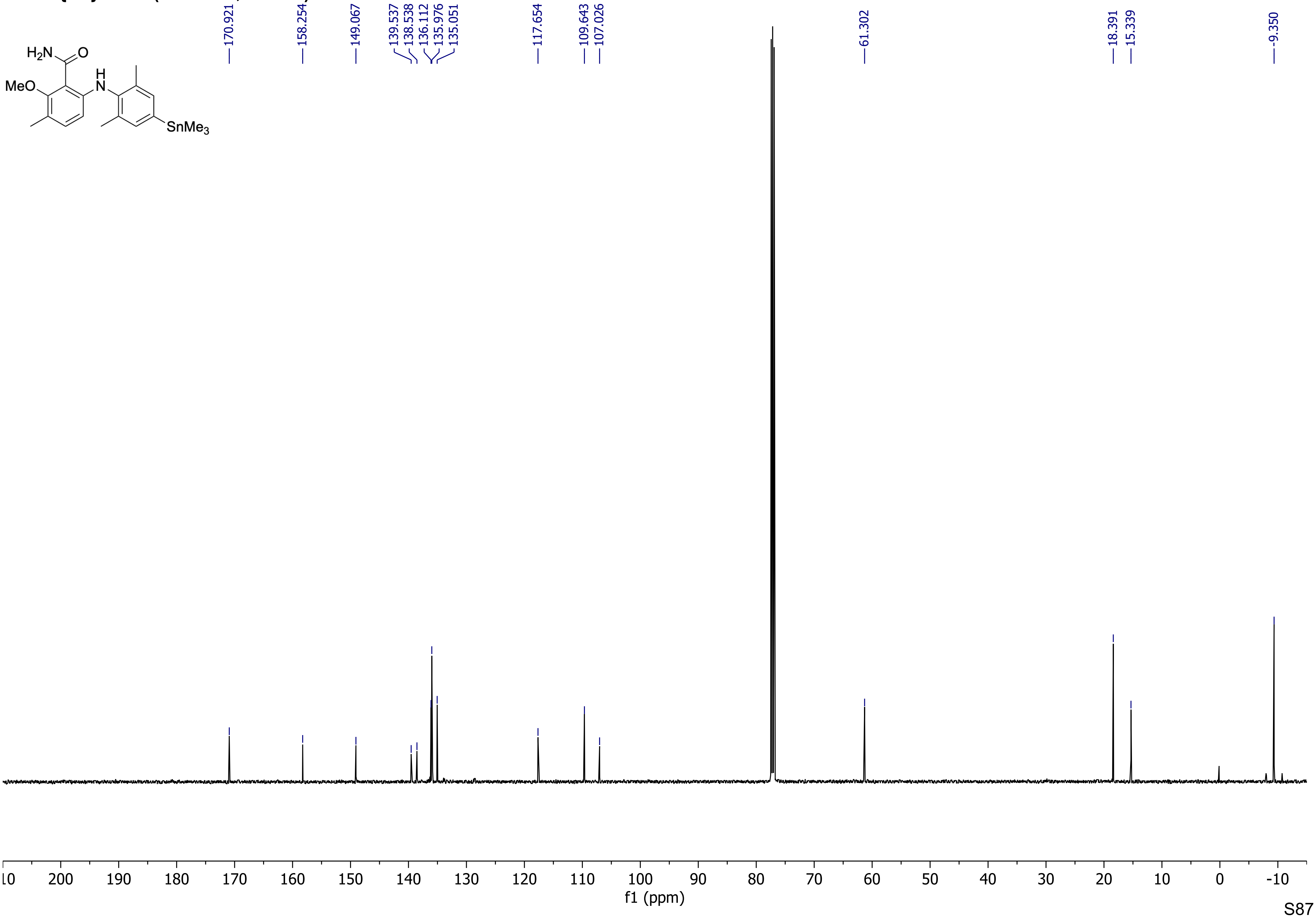

Supplement: MD-016-D4MD00929K-s001 [file MD-016-D4MD00929K-s001.pdf]
